# Supplementary material for: Urinary Metabolomic Profile in Children with Autism Spectrum Disorder
Source: Int J Mol Sci. 2025 Mar 3;26(5):2254. doi: 10.3390/ijms26052254 (PMC11900373; doi:10.3390/ijms26052254)
Supplement: Supplementary file 1 [file ijms-26-02254-s001.zip › ijms-3408884-supplementary.pdf]

Table S1: Comparation of the results of tryptophan and their metabolites in the whole group and separately in the moderate and severe group upon CARS score. Differences are presented as p values; all values are in nM/mmol of creatinine

Metabolite	Sibling (N=44)	ASD (N=44)	CARS<36 (N=28)	CARS>36.5 (N=15)	p value			
	1	2	**3**	4	1-2	1-3	1-4	3-4
TRP					0.04	0.06	0.09	0.74
Mean (SD)	18.20 (8.17)	22.30 (8.53)	22.96 (9.51)	21.74 (6.52)				
Median (Q1, Q3)	16.91 (11.97, 23.63)	19.94 (16.99, 28.36)	19.81 (16.16, 33.22)	21.37 (19.29, 25.67)				
Min - Max	6.03 - 38.83	7.38 - 39.65	9.07 - 39.65	7.38 - 31.30				
ATA					0.99	0.50	0.22	0.40
Mean (SD)	21.21 (9.43)	21.09 (10.78)	23.78 (13.10)	17.34 (7.61)				
Median (Q1, Q3)	21.44 (14.95, 25.43)	19.86 (13.90, 28.61)	20.55 (13.97, 32.92)	19.29 (10.24, 21.16)				
Min - Max	5.52 - 50.67	4.52 - 50.73	5.04 - 53.00	4.52 - 29.51				
IAA					0.78	0.75	0.74	0.83
Mean (SD)	1.79 (1.48)	1.86 (1.37)	1.97 (1.49)	1.57 (1.07)				
Median (Q1, Q3)	1.32 (0.70, 2.28)	1.67 (0.88, 2.61)	1.68 (0.83, 2.76)	1.18 (0.88, 2.37)				
Min - Max	0.11 - 5.84	0.20 - 5.80	0.20 - 5.80	0.22 - 3.53				
IALD					0.10	0.45	0.19	0.50
Mean (SD)	200.29 (119.30)	165.20 (83.91)	172.01 (88.14)	152.50 (76.64)				
Median (Q1, Q3)	174.38 (102.35, 287.53)	147.13 (104.24, 223.62)	147.13 (111.80, 226.26)	134.39 (91.37, 188.69)				
Min - Max	17.43 - 455.15	30.44 - 358.36	30.44 - 358.36	62.03 - 313.85				
IAM					1.00	0.91	0.96	0.78
Mean (SD)	61.77 (37.16)	61.78 (36.77)	65.64 (41.31)	57.77 (28.18)				
Median (Q1, Q3)	55.71 (31.43, 79.18)	57.14 (30.33, 79.70)	57.14 (32.71, 85.72)	58.03 (32.09, 79.70)				

Min - Max	10.43 - 160.04	12.85 - 160.99	18.11 - 160.99	12.85 - 100.63				
IBA					0.98	0.89	0.57	0.95
Mean (SD)	20.13 (17.40)	20.53 (18.82)	21.76 (20.33)	15.77 (13.97)				
Median (Q1, Q3)	17.20 (8.71, 23.79)	12.97 (8.10, 33.69)	12.51 (7.00, 37.91)	12.21 (8.17, 18.71)				
Min - Max	0.81 - 75.60	0.03 - 68.17	0.03 - 68.17	0.40 - 44.33				
ILA					0.53	0.19	0.47	0.96
Mean (SD)	1.08 (0.58)	0.99 (0.58)	0.94 (0.52)	0.94 (0.51)				
Median (Q1, Q3)	1.01 (0.61, 1.40)	0.75 (0.59, 1.13)	0.74 (0.67, 1.02)	0.83 (0.52, 1.40)				
Min - Max	0.18 - 2.41	0.27 - 2.67	0.28 - 2.39	0.27 - 1.80				
IPA					0.27	0.35	0.16	0.94
Mean (SD)	5.13 (3.04)	4.16 (3.11)	4.40 (3.60)	3.57 (1.28)				
Median (Q1, Q3)	4.53 (3.42, 6.86)	3.16 (2.08, 5.15)	3.64 (1.91, 5.54)	3.16 (2.75, 4.50)				
Min - Max	0.20 - 13.98	0.00 - 13.11	0.00 - 13.11	1.27 - 5.71				
KYN					0.86	1.00	0.28	0.62
Mean (SD)	92.94 (61.63)	99.77 (61.68)	80.67 (45.97)	99.77 (46.39)				
Median (Q1, Q3)	71.20 (48.30, 122.81)	82.34 (54.76, 128.66)	77.23 (47.82, 105.30)	97.70 (64.36, 134.02)				
Min - Max	25.41 - 241.15	11.62 - 240.85	11.62 - 205.89	29.30 - 184.66				
MIA					0.80	0.64	0.86	0.13
Mean (SD)	0.07 (0.04)	0.08 (0.06)	0.08 (0.05)	0.06 (0.04)				
Median (Q1, Q3)	0.06 (0.04, 0.10)	0.07 (0.04, 0.11)	0.07 (0.03, 0.11)	0.06 (0.04, 0.07)				
Min - Max	0.01 - 0.21	0.00 - 0.23	0.00 - 0.20	0.00 - 0.14				
NAcTRP					0.92	0.54	0.75	0.26
Mean (SD)	206.05 (118.34)	200.64 (83.98)	212.55 (87.76)	178.41 (74.11)				
Median (Q1, Q3)	185.08 (95.68, 279.38)	185.54 (149.26, 252.54)	225.92 (159.52, 265.84)	184.49 (112.53, 217.93)				
Min - Max	35.88 - 488.19	56.47 - 363.64	56.47 - 344.29	80.06 - 363.64				

TRPN					0.10	0.13	0.72	0.11
Mean (SD)	389.89 (209.89)	456.33 (257.72)	518.74 (278.95)	365.38 (173.84)				
Median (Q1, Q3)	389.13 (246.22, 483.64)	441.10 (230.08, 649.44)	528.14 (281.69, 763.43)	391.67 (238.62, 451.97)				
Min - Max	56.29 - 1041.14	63.19 - 1075.56	63.19 - 1075.56	125.02 - 653.10				
5OHIAA					0.61	0.28	0.63	0.17
Mean (SD)	48.77 (27.63)	50.76 (25.04)	53.62 (23.03)	44.82 (28.83)				
Median (Q1, Q3)	39.12 (31.35, 61.84)	48.64 (31.03, 61.78)	48.64 (33.60, 63.86)	42.98 (23.75, 49.14)				
Min - Max	17.96 - 116.40	9.74 - 116.26	24.96 - 101.83	9.74 - 116.26				
5OHTRP					0.15	0.40	0.01	0.14
Mean (SD)	22.79 (12.56)	19.13 (12.76)	21.98 (14.30)	14.24 (7.92)				
Median (Q1, Q3)	22.21 (11.71, 30.28)	18.39 (10.09, 24.31)	21.52 (12.10, 26.27)	12.46 (9.79, 19.67)				
Min - Max	0.71 - 49.81	0.79 - 54.70	3.48 - 54.70	0.79 - 28.73				
5MIAA					0.89	0.13	0.37	0.19
Mean (SD)	15.12 (9.89)	16.40 (13.23)	11.43 (9.37)	19.68 (13.52)				
Median (Q1, Q3)	15.11 (6.53, 22.07)	10.93 (7.69, 22.06)	9.05 (6.86, 11.35)	16.73 (7.84, 31.74)				
Min - Max	0.79 - 45.27	1.12 - 52.02	0.90 - 38.93	4.06 - 44.47				
U-Creatinine					0.43	0.68	0.50	0.99
Mean (SD)	7.58 (4.27)	8.36 (4.76)	8.40 (5.06)	8.29 (4.33)				
Median (Q1, Q3)	6.55 (4.62, 9.93)	7.70 (4.55, 10.88)	7.10 (4.40, 10.65)	8.40 (5.25, 11.00)				
Min - Max	0.10 - 18.30	0.70 - 18.20	2.30 - 18.20	0.70 - 15.90				

1 – 2 ASSOCIATION

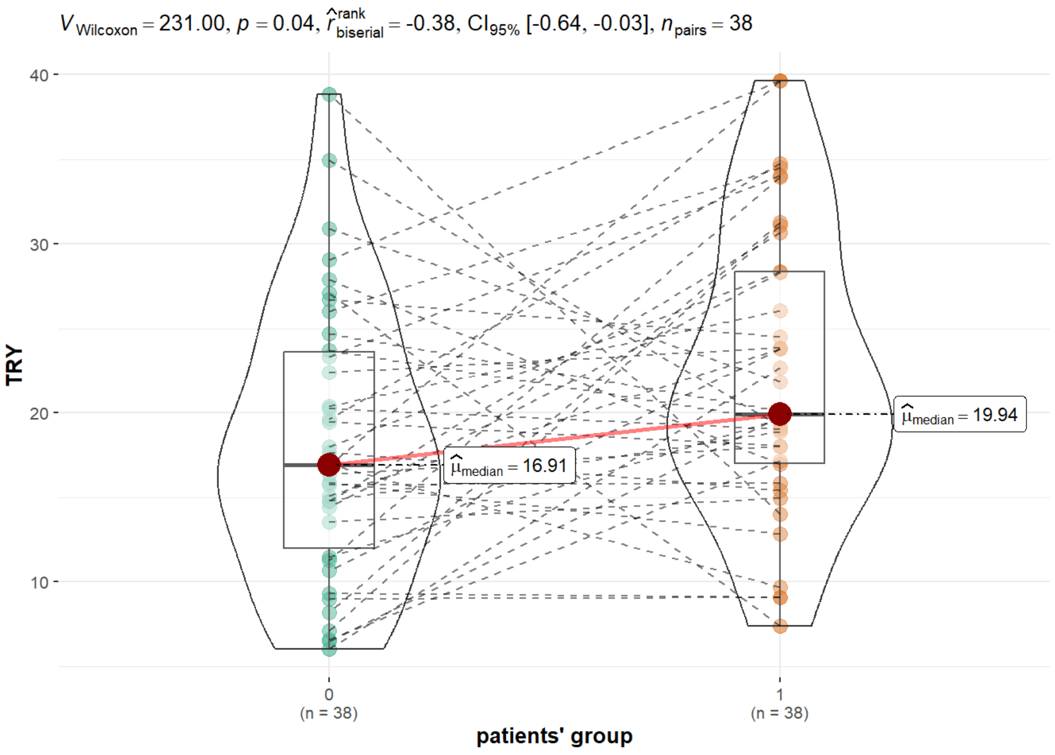
$V_{\text{Wilcoxon}} = 231.00$, $p = 0.04$, $\widehat{r}_{\text{biserial}}^{\text{rank}} = -0.38$, $\text{CI}_{95\%}$ [-0.64, -0.03], $n_{\text{pairs}} = 38$
TRY
40
30
20
10
$\widehat{\mu}_{\text{median}} = 16.91$
$\widehat{\mu}_{\text{median}} = 19.94$
0
(n = 38)
1
(n = 38)
patients' group

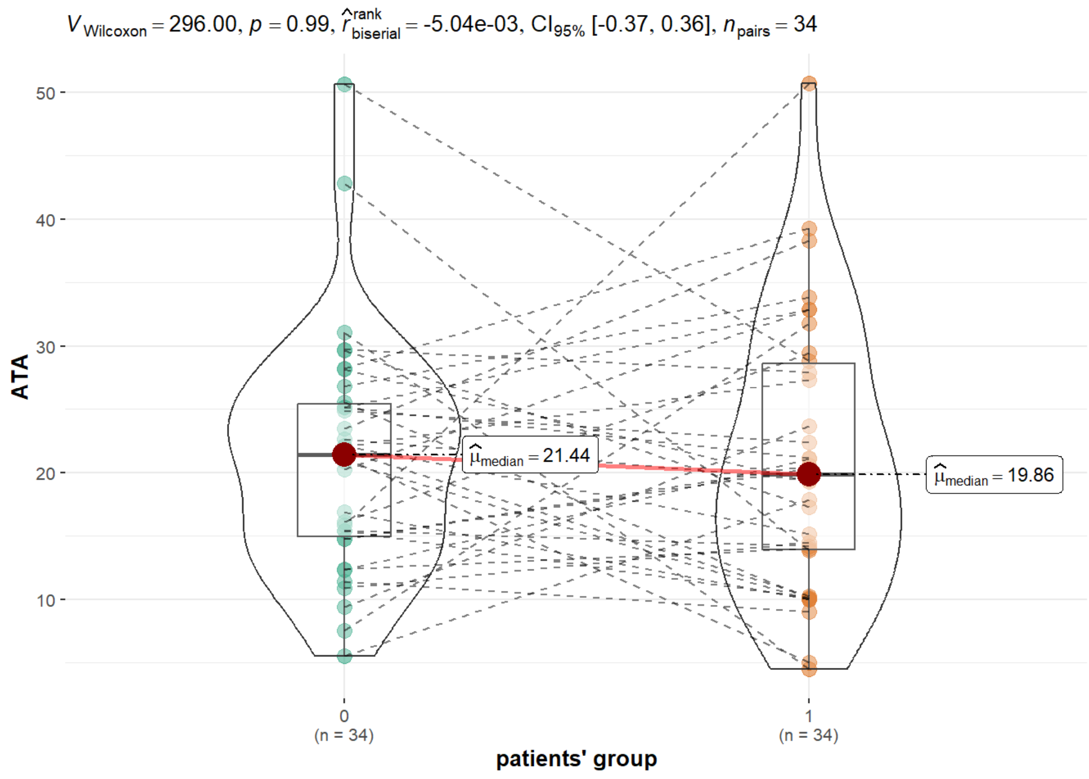

$V_{Wilcoxon} = 296.00$, $p = 0.99$, $\hat{r}^{rank}_{biserial} = -5.04e-03$, $CI_{95\%}$ [-0.37, 0.36], $n_{pairs} = 34$
ATA
50
40
30
20
10
$\hat{\mu}_{median} = 21.44$
$\hat{\mu}_{median} = 19.86$
0
(n = 34)
1
(n = 34)
patients' group

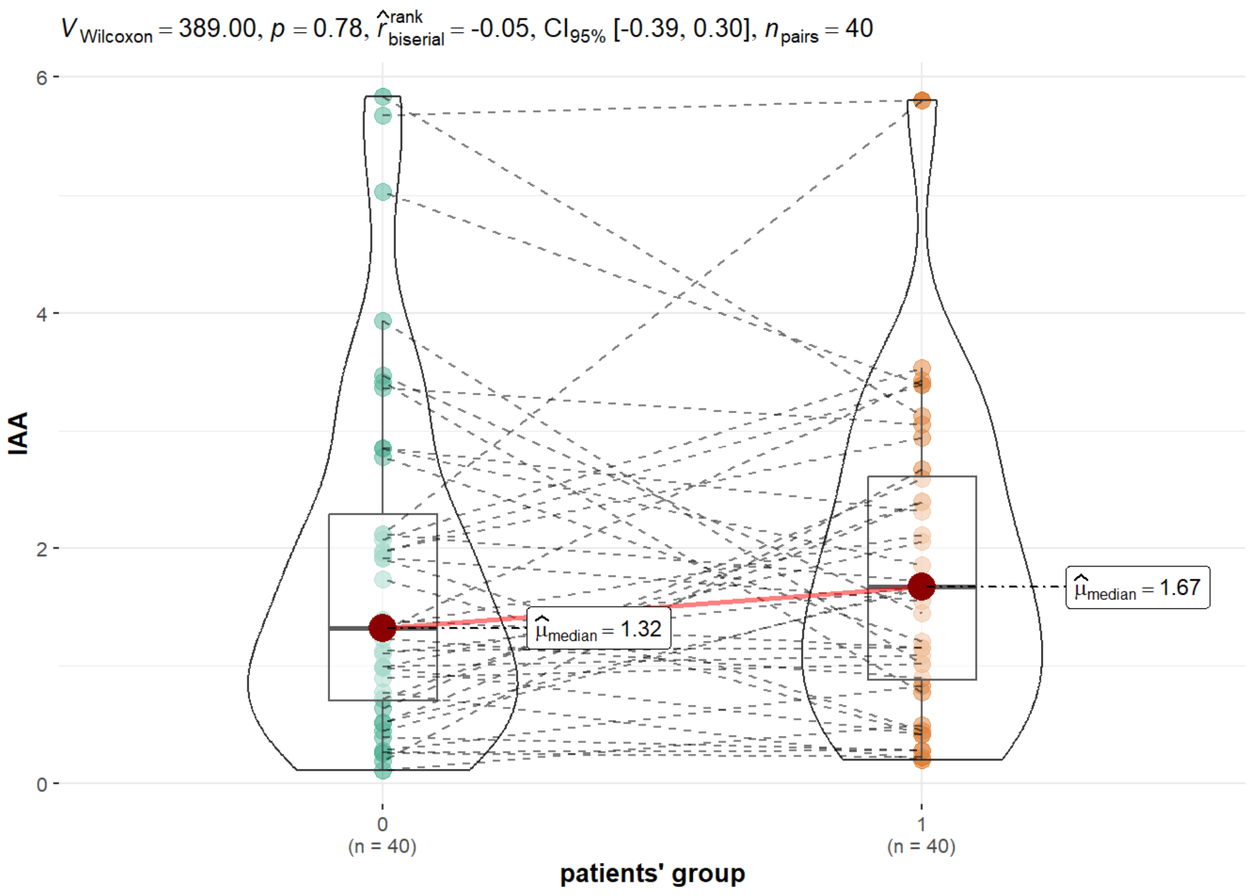

$V_{Wilcoxon} = 389.00$, $p = 0.78$, $\hat{r}^{rank}_{biserial} = -0.05$, $CI_{95\%}$ [-0.39, 0.30], $n_{pairs} = 40$
6
4
2
0
IAA
$\hat{\mu}_{median} = 1.32$
$\hat{\mu}_{median} = 1.67$
0
(n = 40)
1
(n = 40)
patients' group

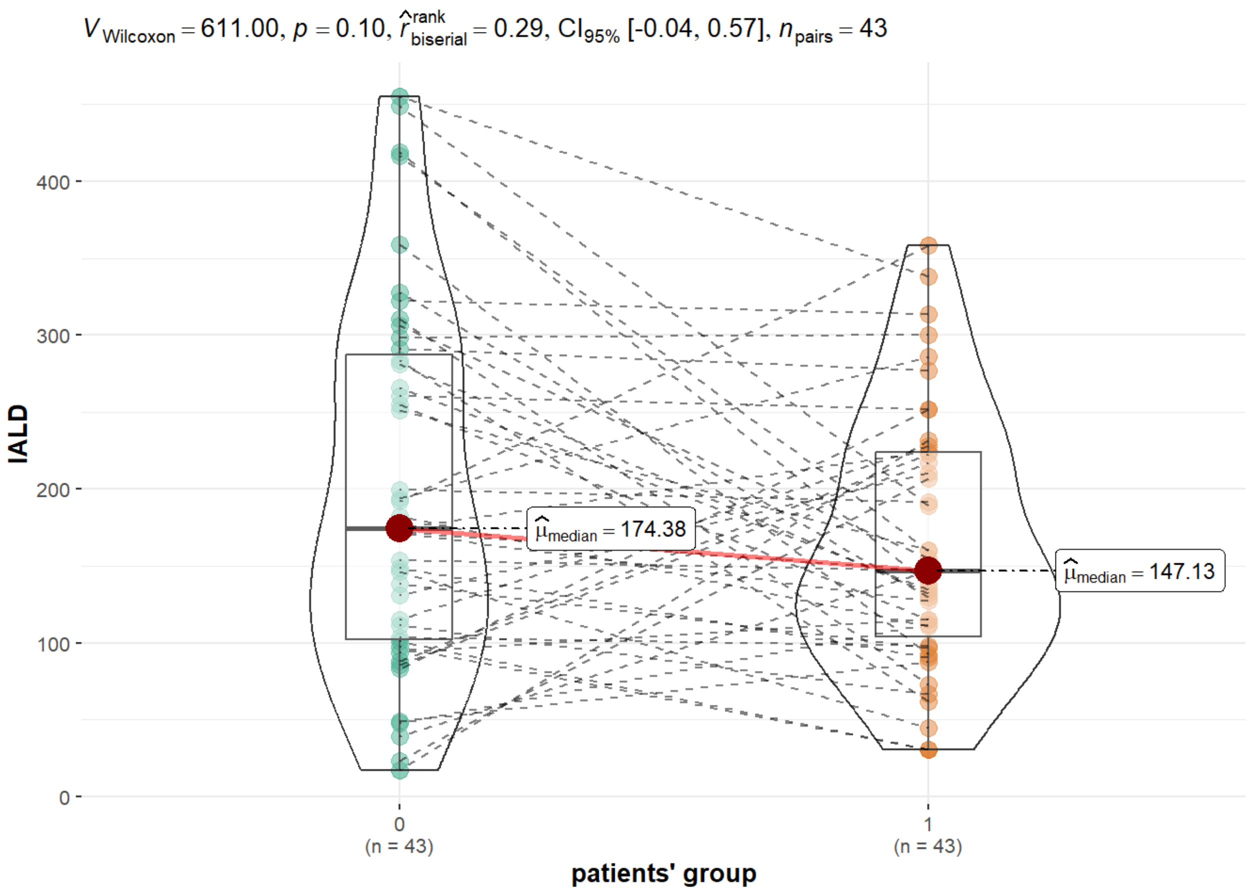
$V_{Wilcoxon} = 611.00$, $p = 0.10$, $\hat{r}^{rank}_{biserial} = 0.29$, $CI_{95\%}$ [-0.04, 0.57], $n_{pairs} = 43$
IALD
400
300
200
100
0
$\hat{\mu}_{median} = 174.38$
$\hat{\mu}_{median} = 147.13$
0
(n = 43)
1
(n = 43)
patients' group

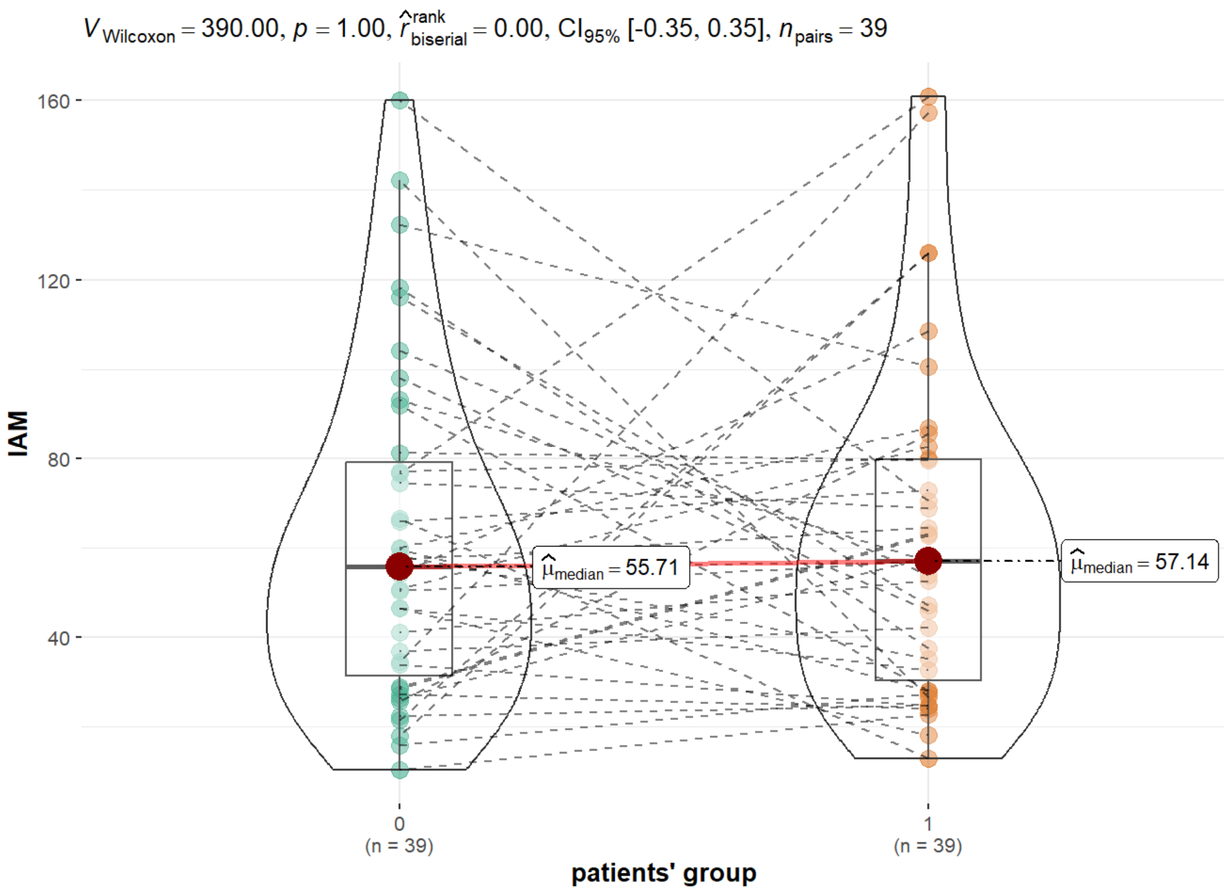
$V_{Wilcoxon} = 390.00$, $p = 1.00$, $\hat{r}^{rank}_{biserial} = 0.00$, $CI_{95\%}$ [-0.35, 0.35], $n_{pairs} = 39$
160
120
80
40
IAM
$\hat{\mu}_{median} = 55.71$
$\hat{\mu}_{median} = 57.14$
0
(n = 39)
1
(n = 39)
patients' group

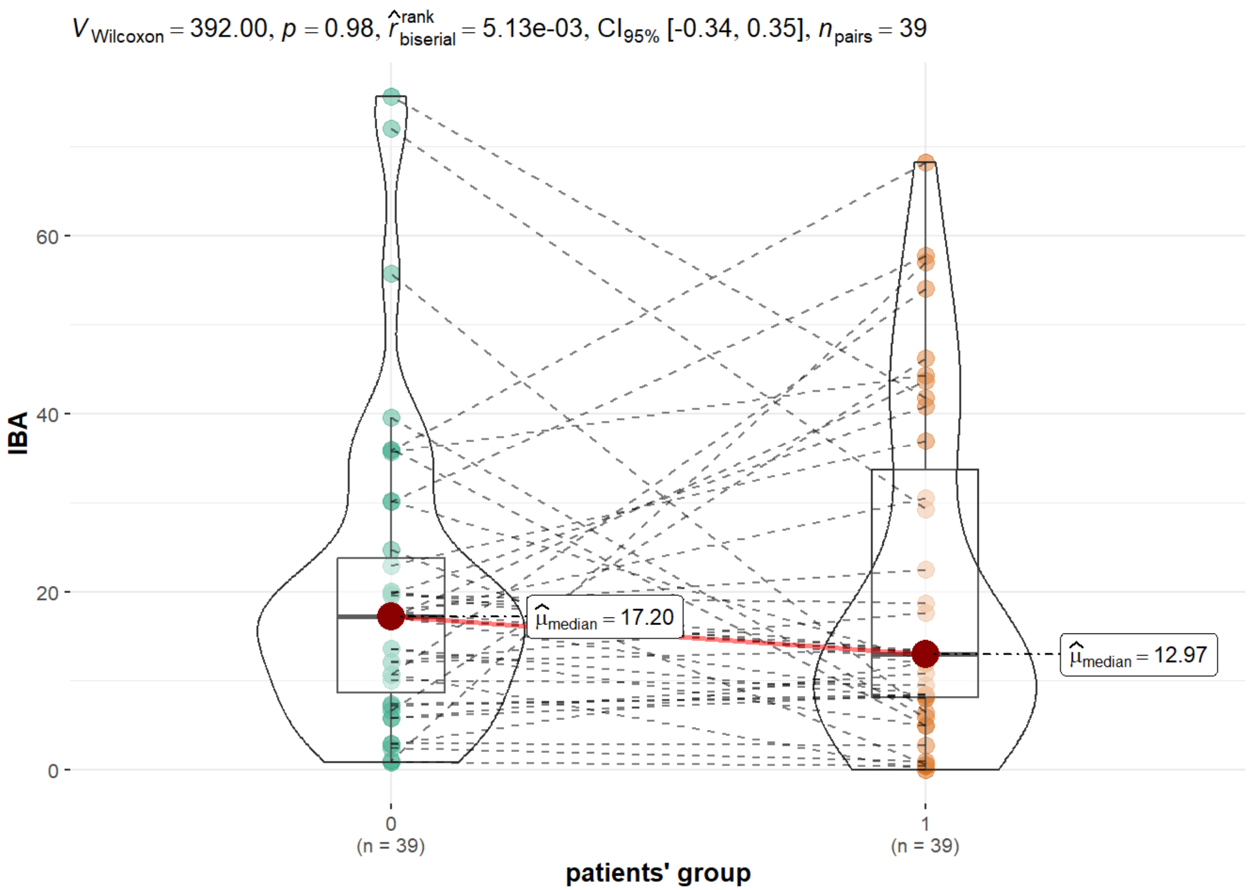

$V_{Wilcoxon} = 392.00$, $p = 0.98$, $\hat{r}^{rank}_{biserial} = 5.13e-03$, $CI_{95\%}$ [-0.34, 0.35], $n_{pairs} = 39$
IBA
60
40
20
0
$\hat{\mu}_{median} = 17.20$
$\hat{\mu}_{median} = 12.97$
0
(n = 39)
1
(n = 39)
patients' group

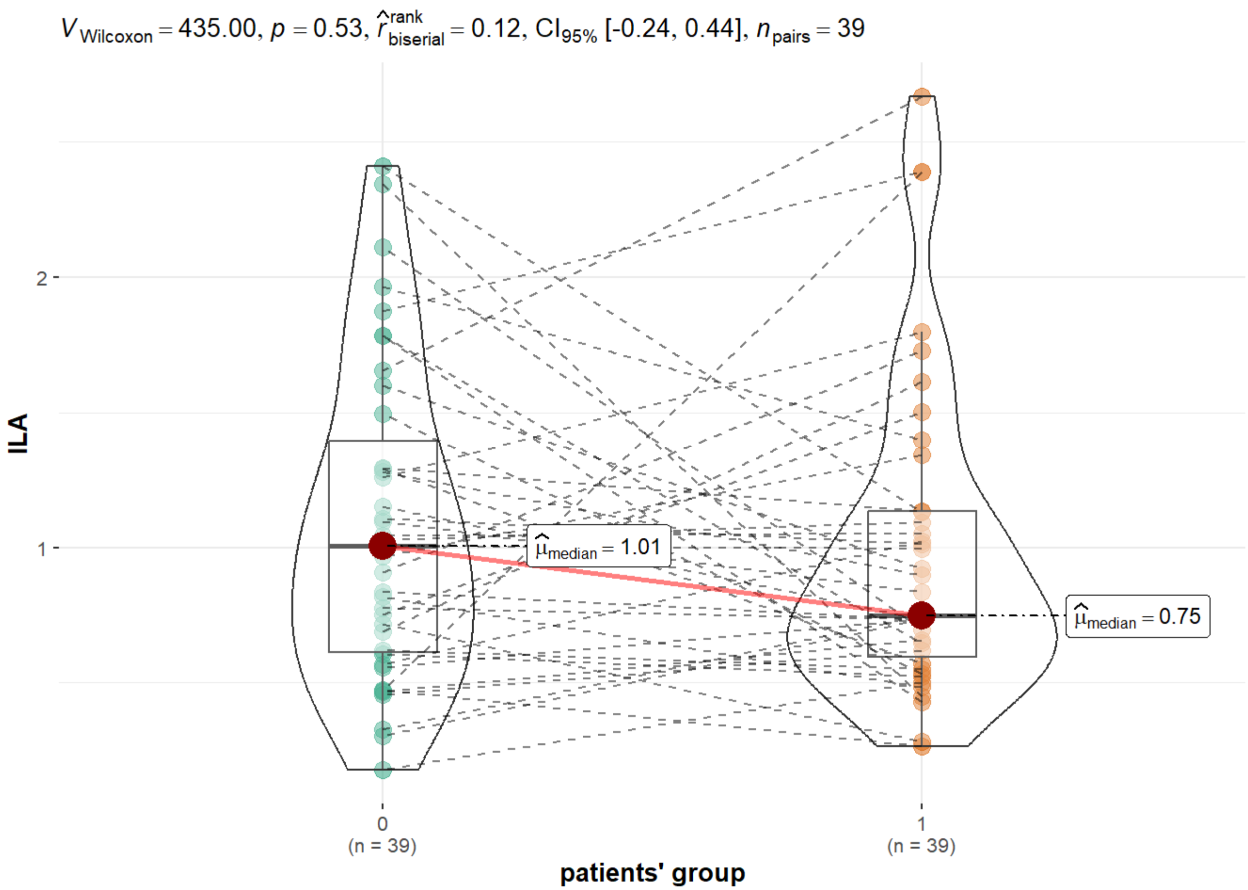

$V_{\text{Wilcoxon}} = 435.00$, $p = 0.53$, $\hat{r}^{\text{rank}}_{\text{biserial}} = 0.12$, $\text{CI}_{95\%}$ [-0.24, 0.44], $n_{\text{pairs}} = 39$
ILA
2
1
$\hat{\mu}_{\text{median}} = 1.01$
$\hat{\mu}_{\text{median}} = 0.75$
0
(n = 39)
1
(n = 39)
patients' group

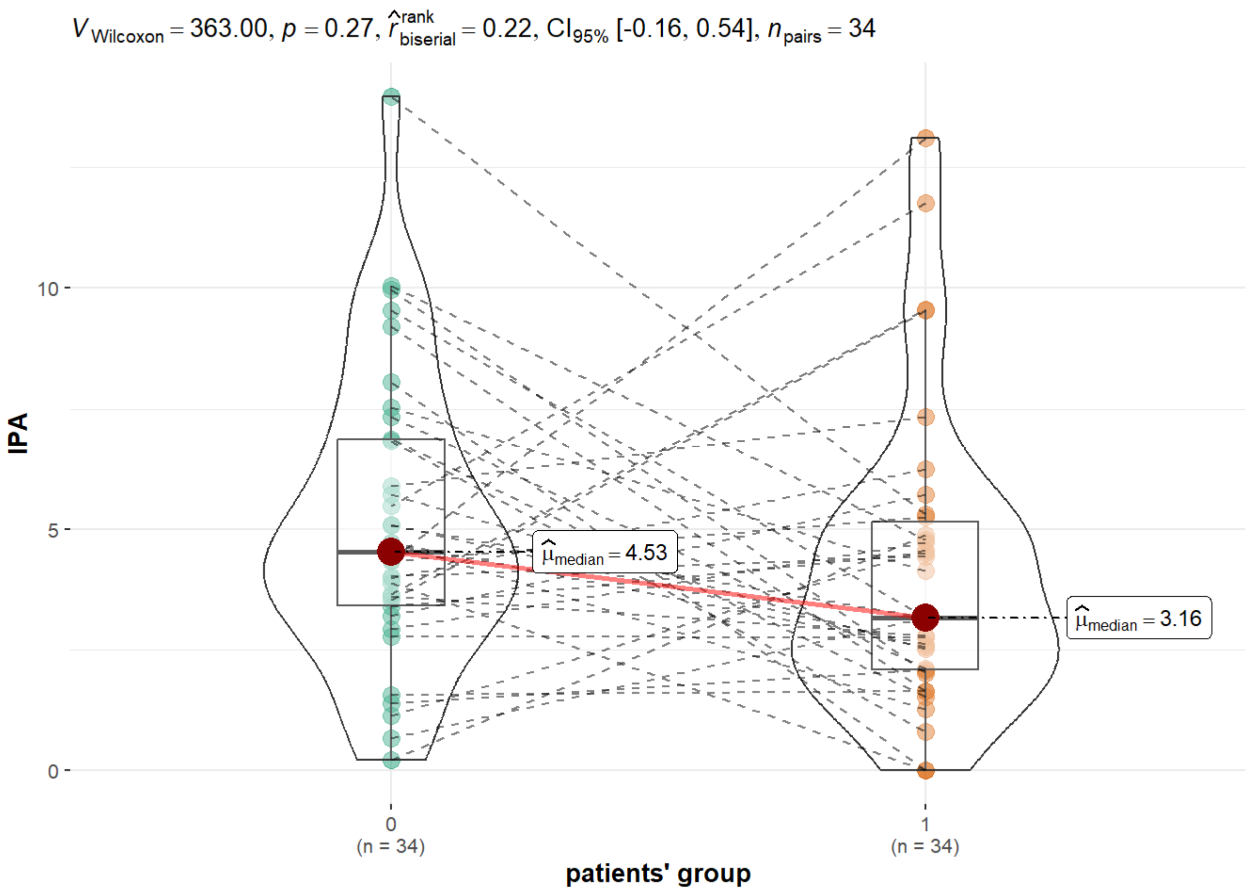

$V_{Wilcoxon} = 363.00$, $p = 0.27$, $\hat{r}^{rank}_{biserial} = 0.22$, $CI_{95\%}$ [-0.16, 0.54], $n_{pairs} = 34$
IPA
10
5
0
$\hat{\mu}_{median} = 4.53$
$\hat{\mu}_{median} = 3.16$
0
(n = 34)
1
(n = 34)
patients' group

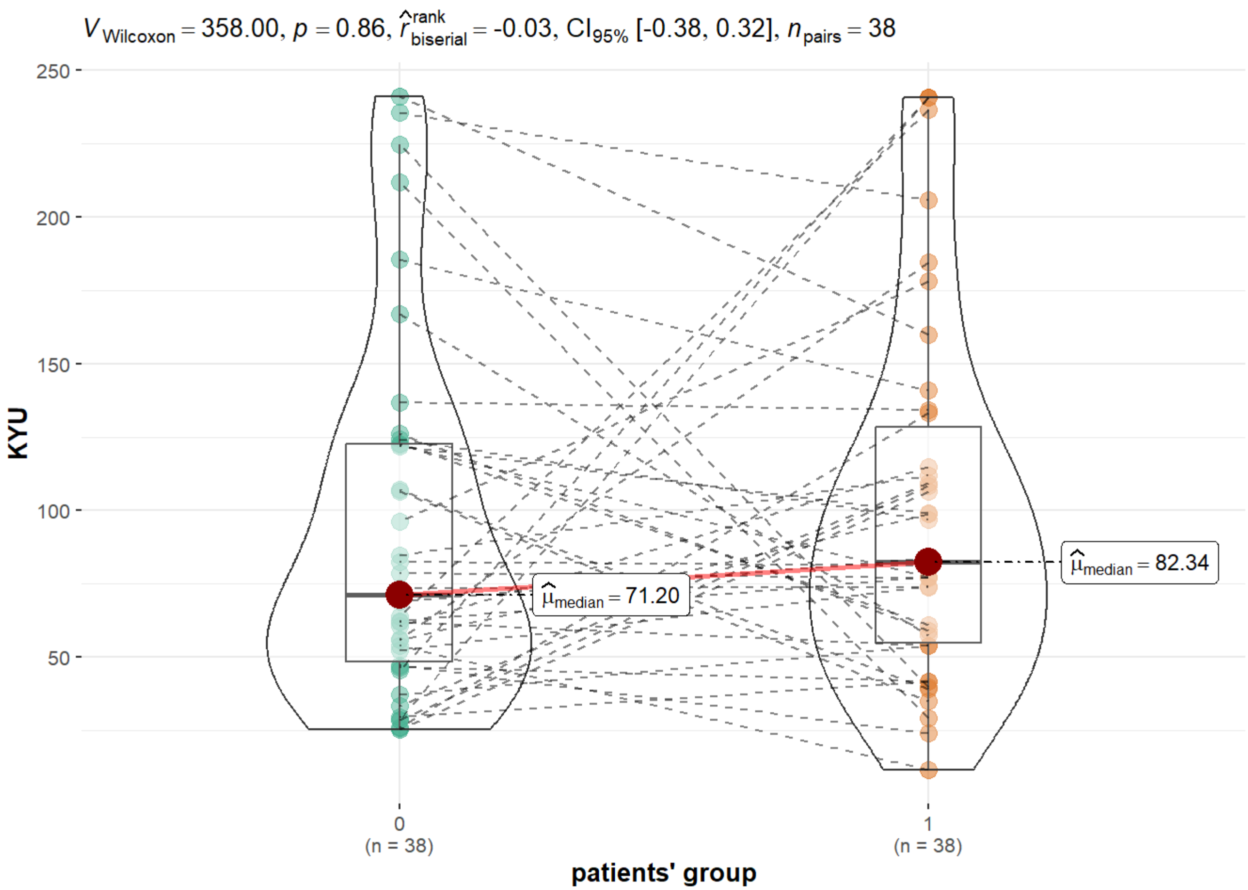

$V_{Wilcoxon} = 358.00$, $p = 0.86$, $\hat{r}^{rank}_{biserial} = -0.03$, $CI_{95\%}$ [-0.38, 0.32], $n_{pairs} = 38$
250
200
150
100
50
KYU
$\hat{\mu}_{median} = 71.20$
$\hat{\mu}_{median} = 82.34$
0
(n = 38)
1
(n = 38)
patients' group

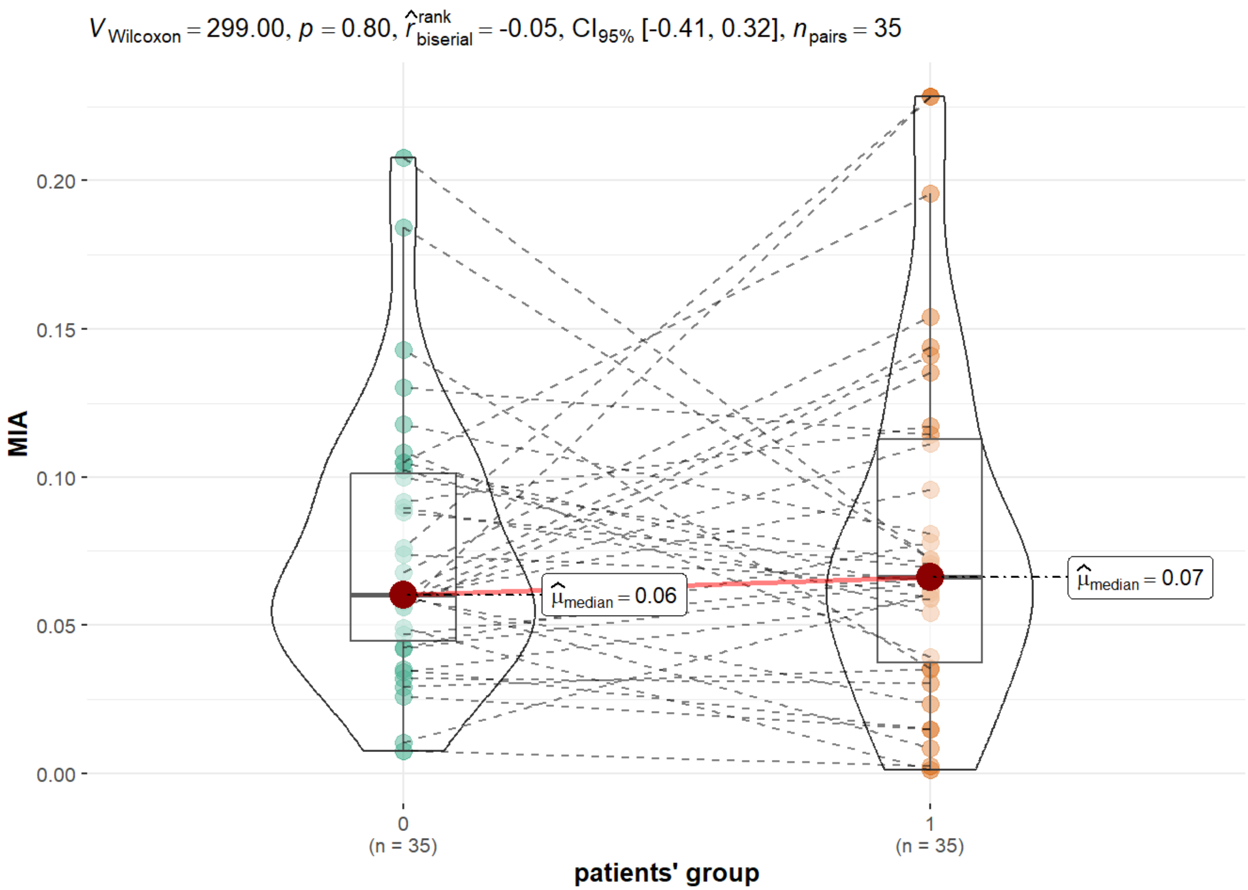

$V_{Wilcoxon} = 299.00$, $p = 0.80$, $\hat{r}^{rank}_{biserial} = -0.05$, $CI_{95\%}$ [-0.41, 0.32], $n_{pairs} = 35$
MIA
0.20
0.15
0.10
0.05
0.00
$\hat{\mu}_{median} = 0.06$
$\hat{\mu}_{median} = 0.07$
0
(n = 35)
1
(n = 35)
patients' group

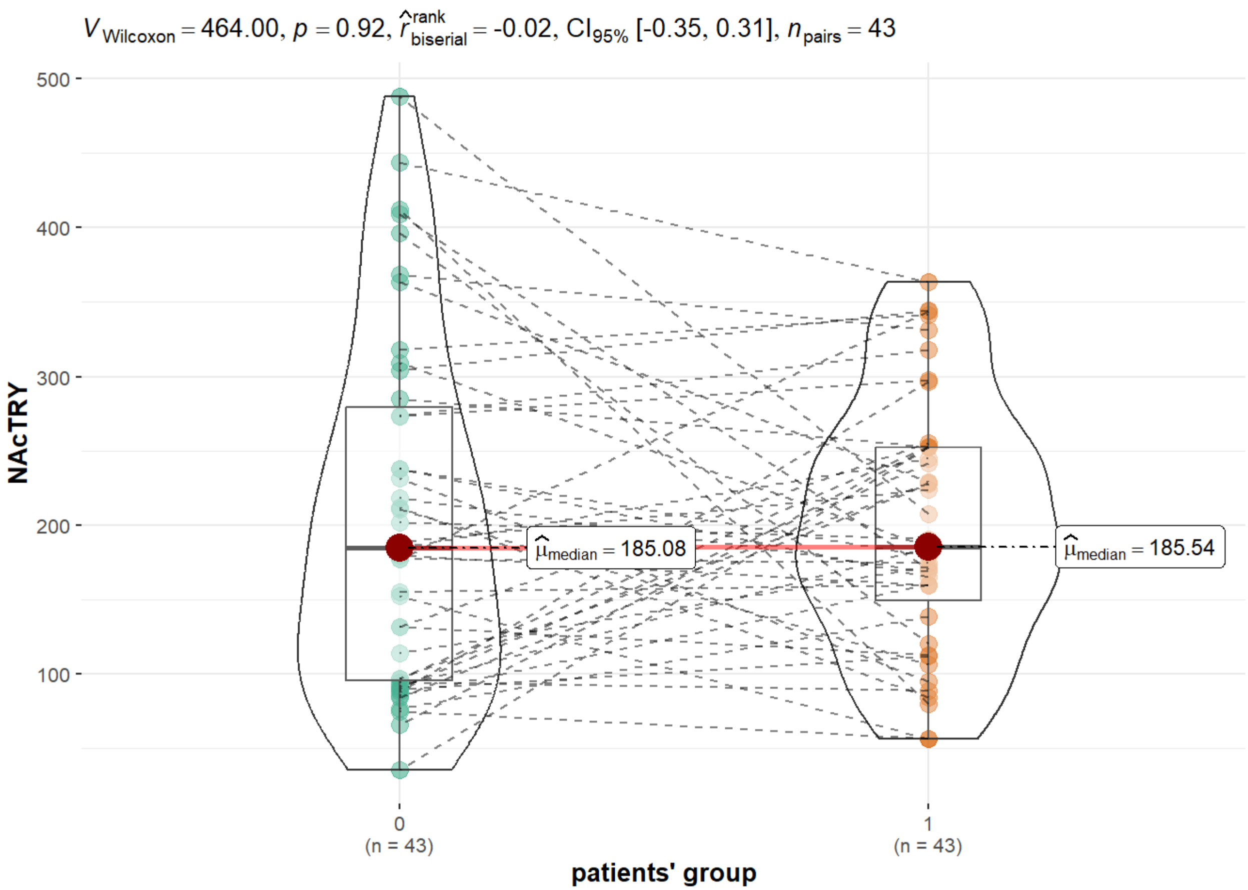
$V_{Wilcoxon} = 464.00$, $p = 0.92$, $\hat{r}^{rank}_{biserial} = -0.02$, $CI_{95\%}$ [-0.35, 0.31], $n_{pairs} = 43$
500
400
300
200
100
NAcTRY
$\hat{\mu}_{median} = 185.08$
$\hat{\mu}_{median} = 185.54$
0
(n = 43)
1
(n = 43)
patients' group

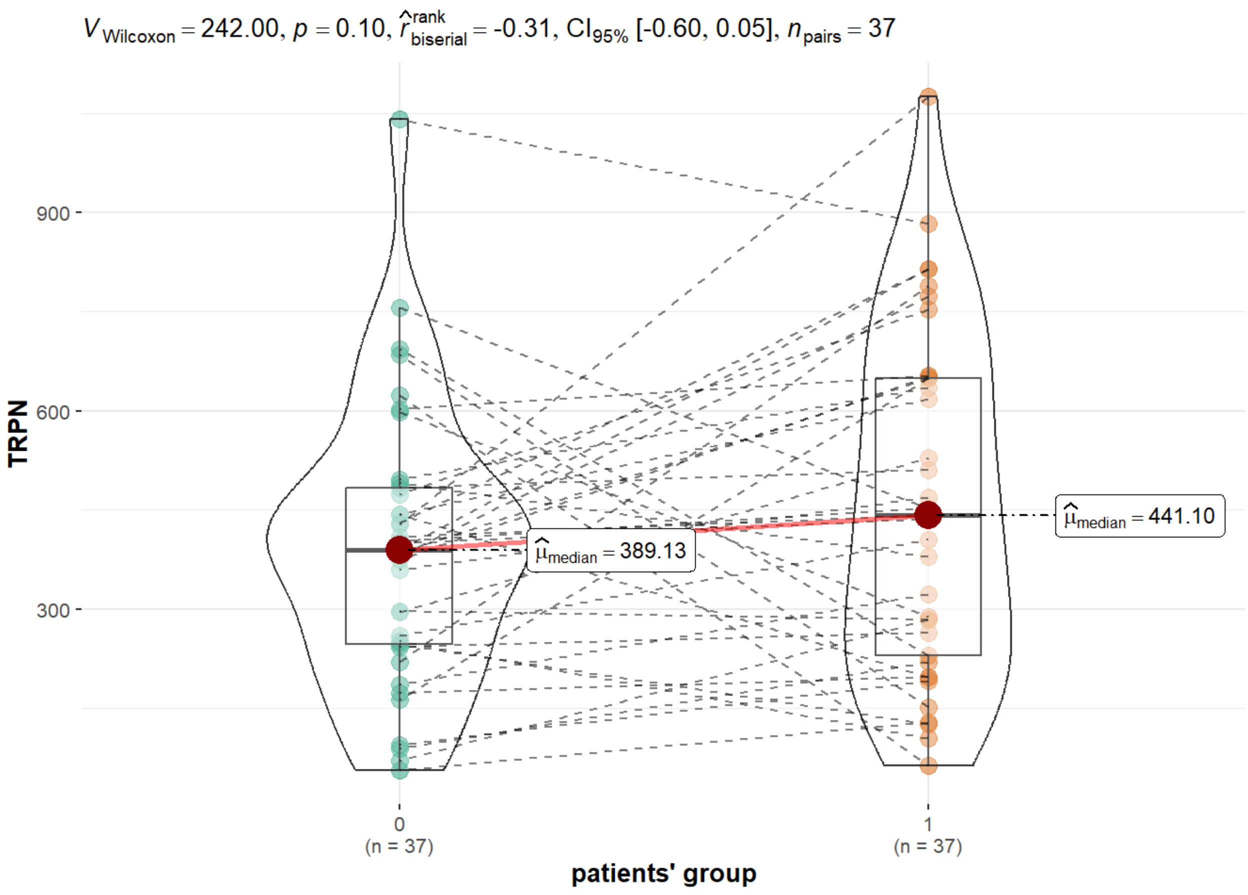

$V_{Wilcoxon} = 242.00$, $p = 0.10$, $\hat{r}^{rank}_{biserial} = -0.31$, $CI_{95\%}$ [-0.60, 0.05], $n_{pairs} = 37$
TRPN
900
600
300
$\hat{\mu}_{median} = 389.13$
$\hat{\mu}_{median} = 441.10$
0
(n = 37)
1
(n = 37)
patients' group

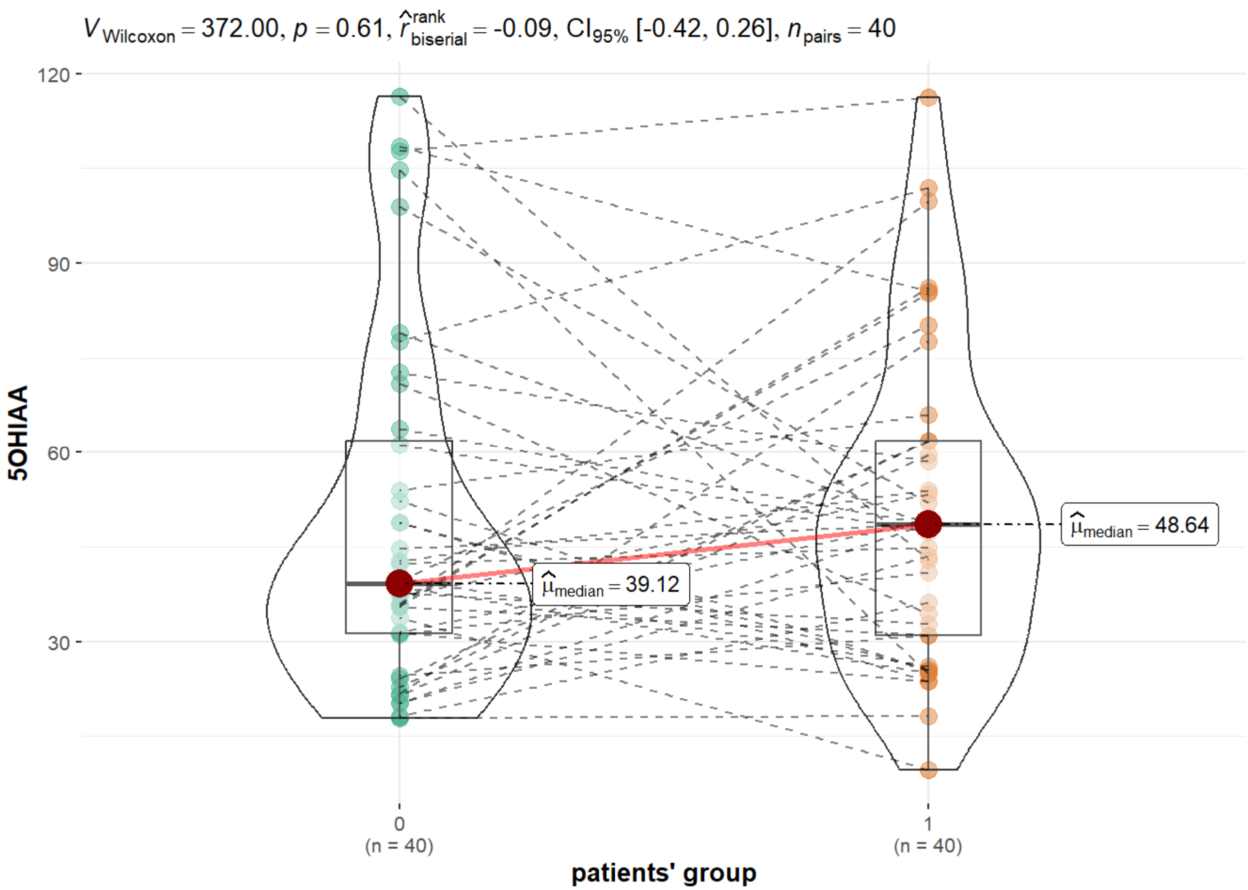

$V_{Wilcoxon} = 372.00$, $p = 0.61$, $\hat{r}^{rank}_{biserial} = -0.09$, $CI_{95\%}$ [-0.42, 0.26], $n_{pairs} = 40$
5OHIAA
120
90
60
30
$\hat{\mu}_{median} = 39.12$
$\hat{\mu}_{median} = 48.64$
0
(n = 40)
1
(n = 40)
patients' group

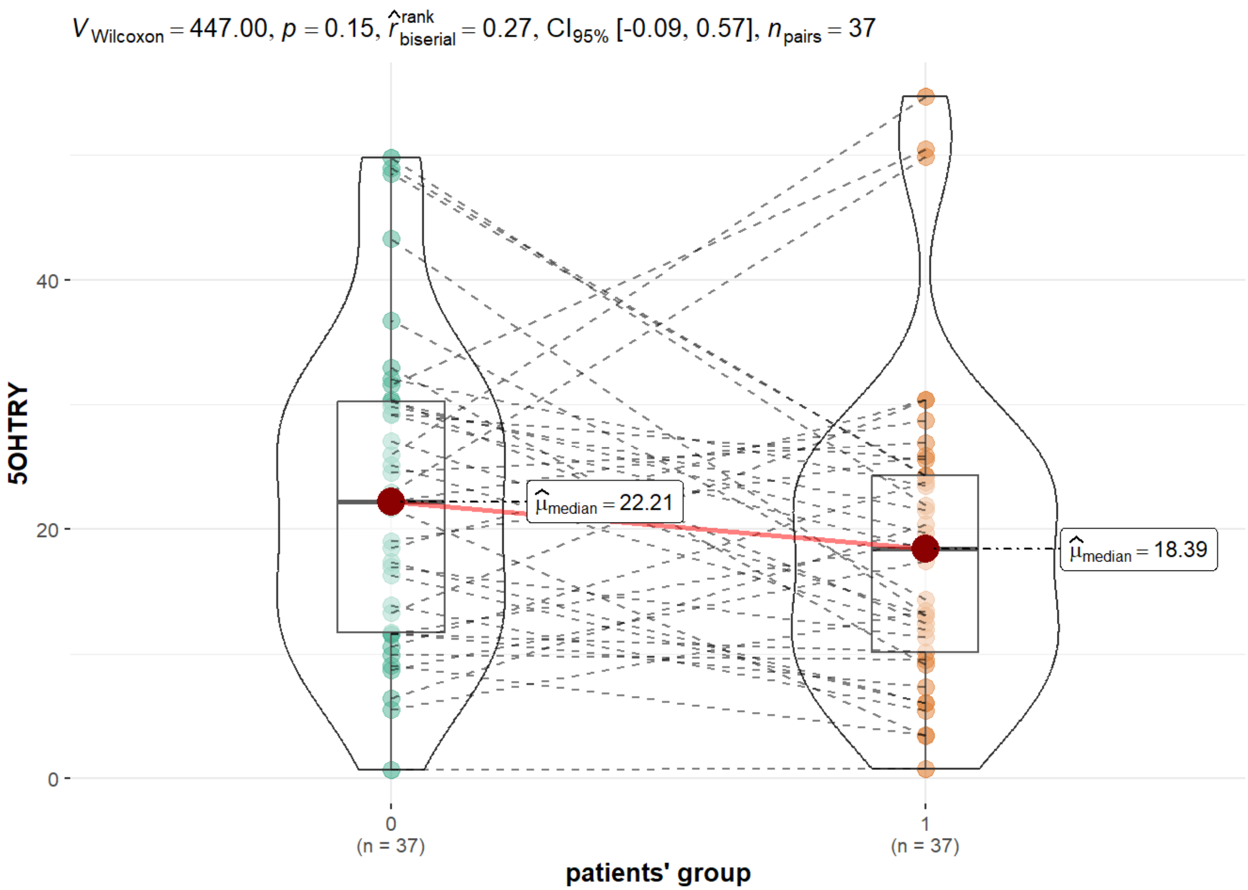

$V_{\text{Wilcoxon}} = 447.00$, $p = 0.15$, $\hat{r}^{\text{rank}}_{\text{biserial}} = 0.27$, $\text{CI}_{95\%}$ [-0.09, 0.57], $n_{\text{pairs}} = 37$
5OHTRY
40
20
0
$\hat{\mu}_{\text{median}} = 22.21$
$\hat{\mu}_{\text{median}} = 18.39$
0
(n = 37)
1
(n = 37)
patients' group

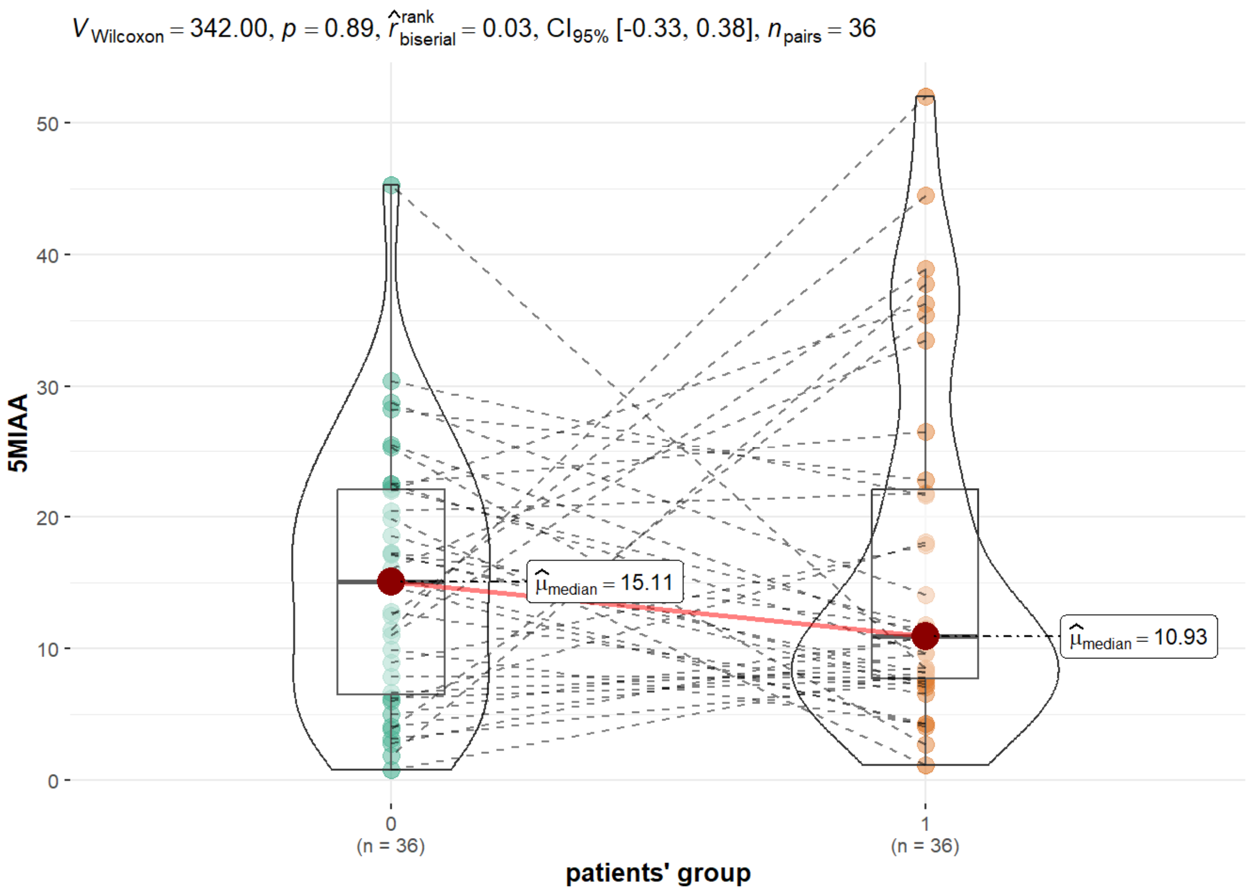

V_Wilcoxon = 342.00, p = 0.89, r̂_biserial^rank = 0.03, CI_95% [-0.33, 0.38], n_pairs = 36
5MIAA
50
40
30
20
10
0
μ̂_median = 15.11
μ̂_median = 10.93
0
(n = 36)
1
(n = 36)
patients' group

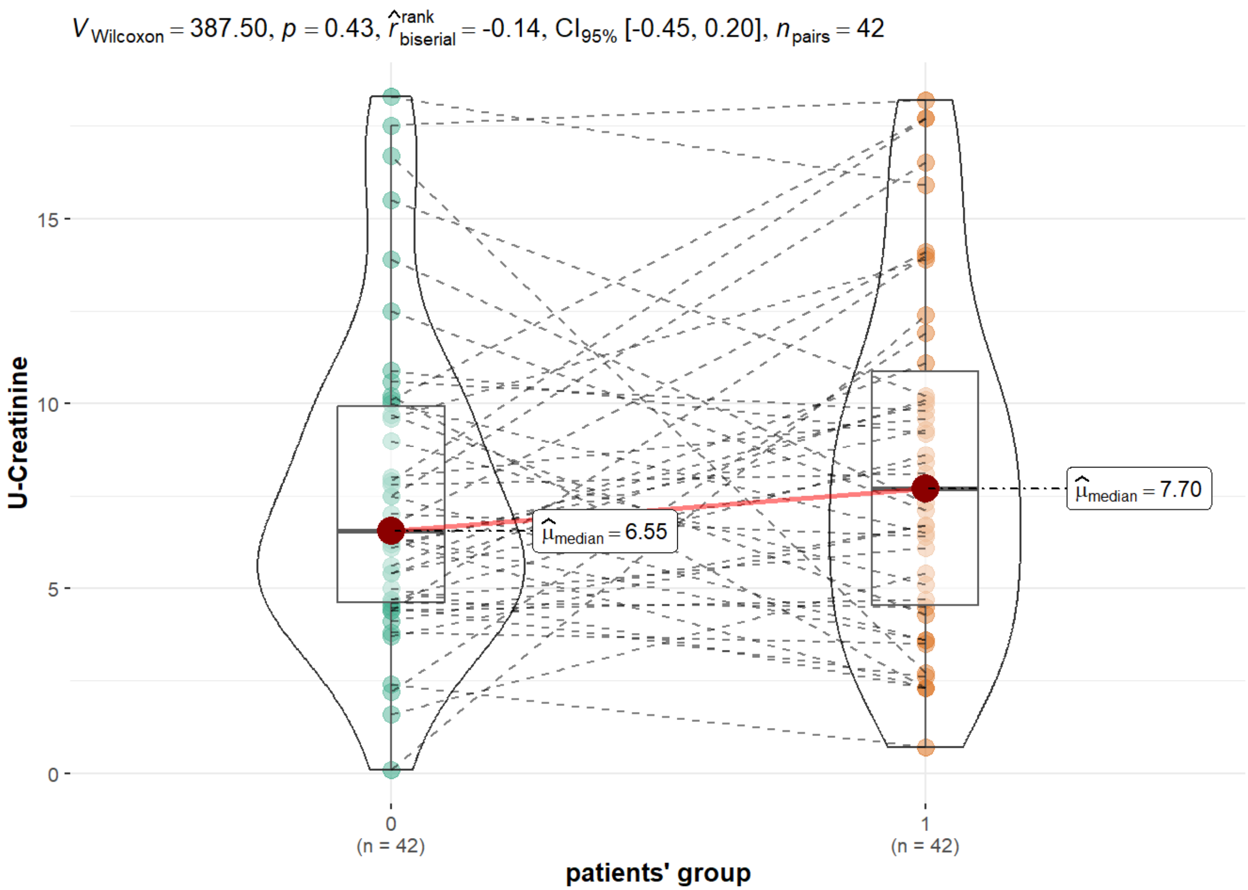

$V_{\text{Wilcoxon}} = 387.50$, $p = 0.43$, $\hat{r}^{\text{rank}}_{\text{biserial}} = -0.14$, $\text{CI}_{95\%}$ [-0.45, 0.20], $n_{\text{pairs}} = 42$
U-Creatinine
0
5
10
15
$\hat{\mu}_{\text{median}} = 6.55$
$\hat{\mu}_{\text{median}} = 7.70$
0
(n = 42)
1
(n = 42)
patients' group

1 – 3 ASSOCIATION

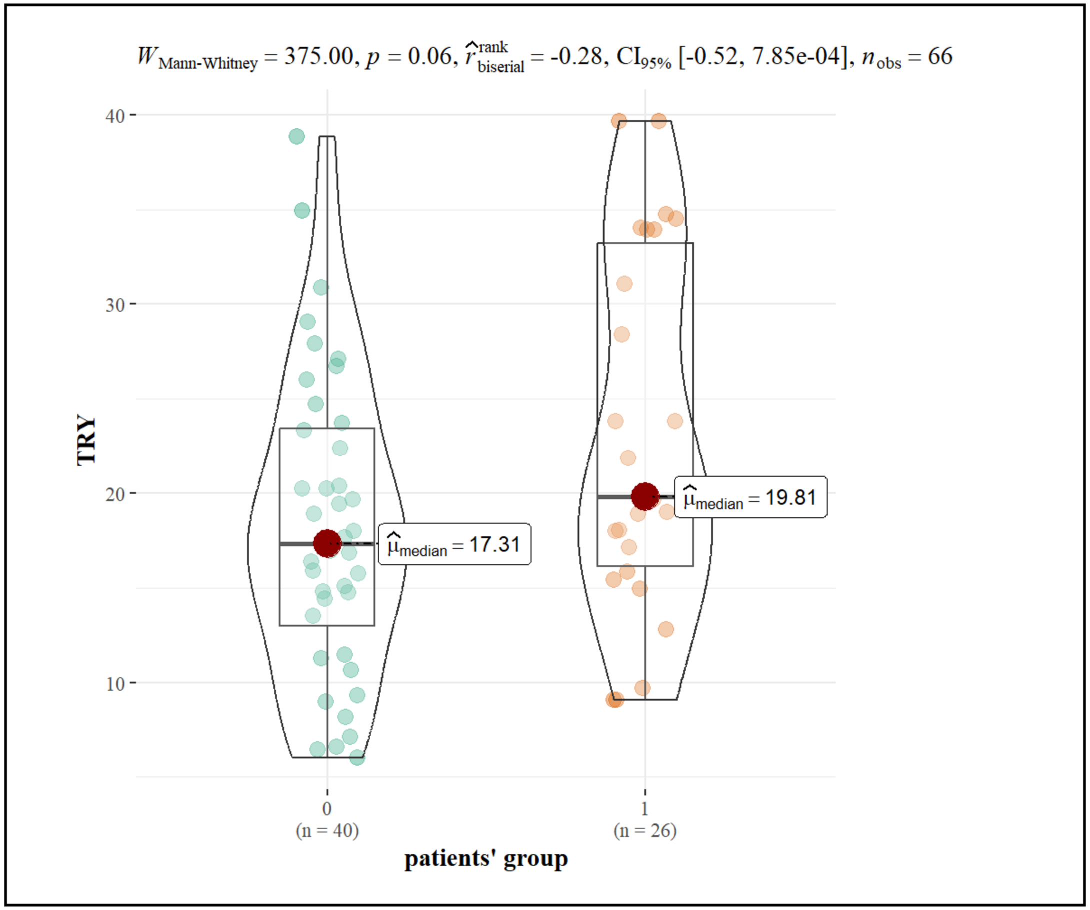
$W_{\text{Mann-Whitney}} = 375.00$, $p = 0.06$, $\hat{r}_{\text{biserial}}^{\text{rank}} = -0.28$, $\text{CI}_{95\%}$ [-0.52, 7.85e-04], $n_{\text{obs}} = 66$
TRY
40
30
20
10
$\hat{\mu}_{\text{median}} = 17.31$
$\hat{\mu}_{\text{median}} = 19.81$
0
(n = 40)
1
(n = 26)
patients' group

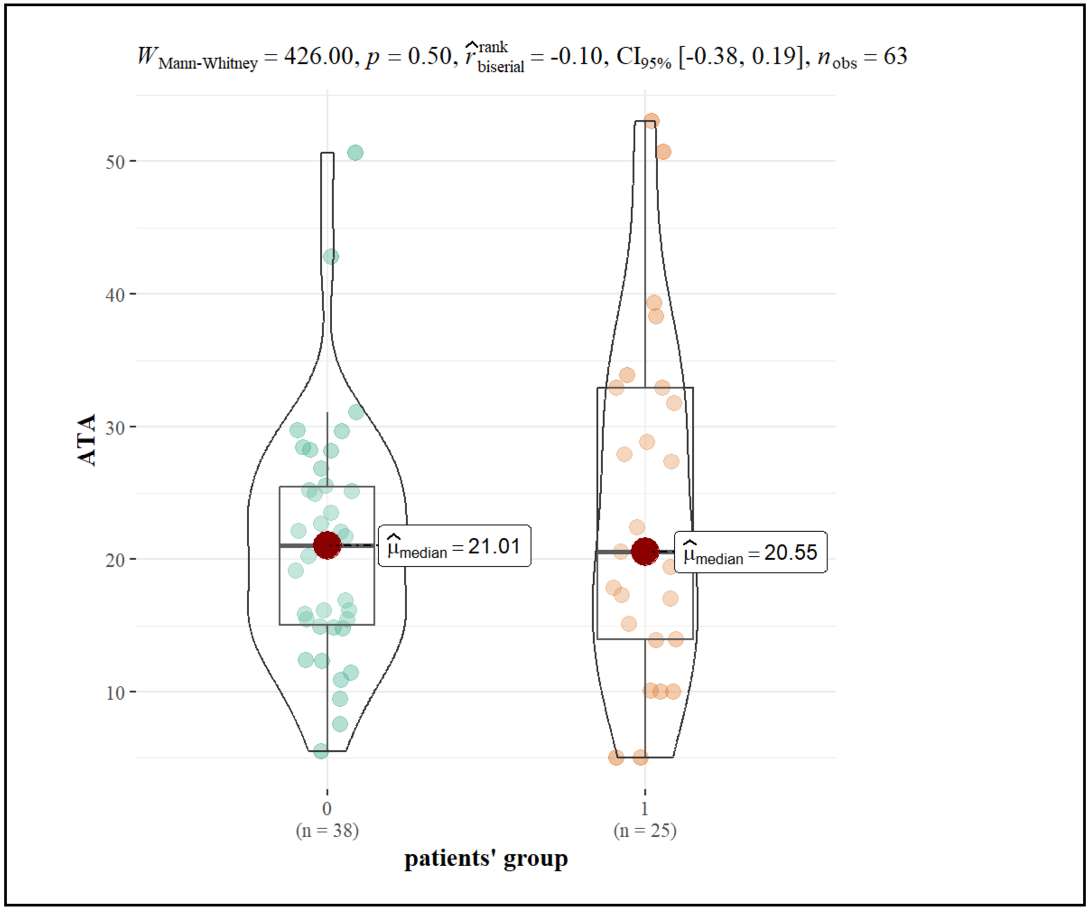

$W_{\text{Mann-Whitney}}$ = 426.00, p = 0.50, $\hat{r}^{\text{rank}}_{\text{biserial}}$ = -0.10, $\text{CI}_{95\%}$ [-0.38, 0.19], n_{obs} = 63
ATA
50
40
30
20
10
$\hat{\mu}_{\text{median}}$ = 21.01
$\hat{\mu}_{\text{median}}$ = 20.55
0
(n = 38)
1
(n = 25)
patients' group

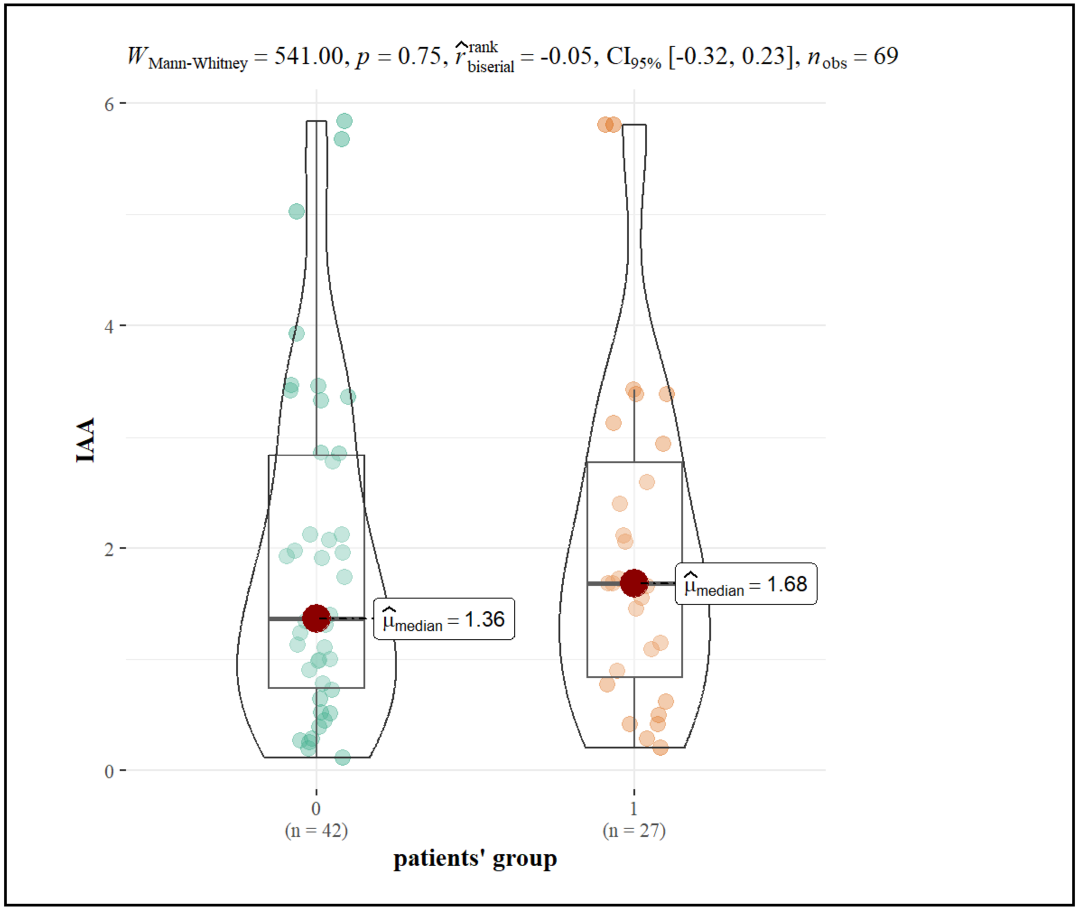

$W_{\text{Mann-Whitney}}$ = 541.00, p = 0.75, $\hat{r}_{\text{biserial}}^{\text{rank}}$ = -0.05, $\text{CI}_{95\%}$ [-0.32, 0.23], n_{obs} = 69
IAA
6
4
2
0
$\hat{\mu}_{\text{median}}$ = 1.36
$\hat{\mu}_{\text{median}}$ = 1.68
0
(n = 42)
1
(n = 27)
patients' group

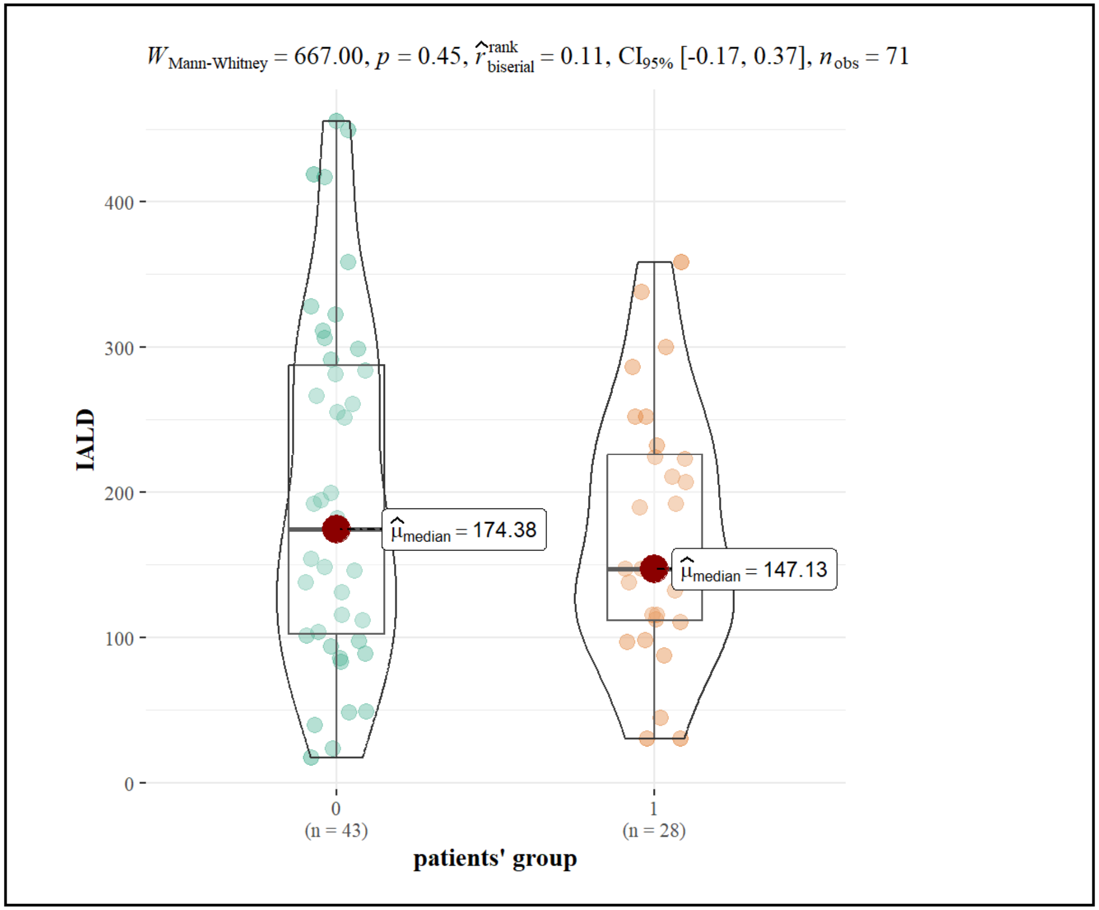

$W_{\text{Mann-Whitney}} = 667.00$, $p = 0.45$, $\hat{r}_{\text{biserial}}^{\text{rank}} = 0.11$, $\text{CI}_{95\%}$ [-0.17, 0.37], $n_{\text{obs}} = 71$
IALD
400
300
200
100
0
$\hat{\mu}_{\text{median}} = 174.38$
$\hat{\mu}_{\text{median}} = 147.13$
0
(n = 43)
1
(n = 28)
patients' group

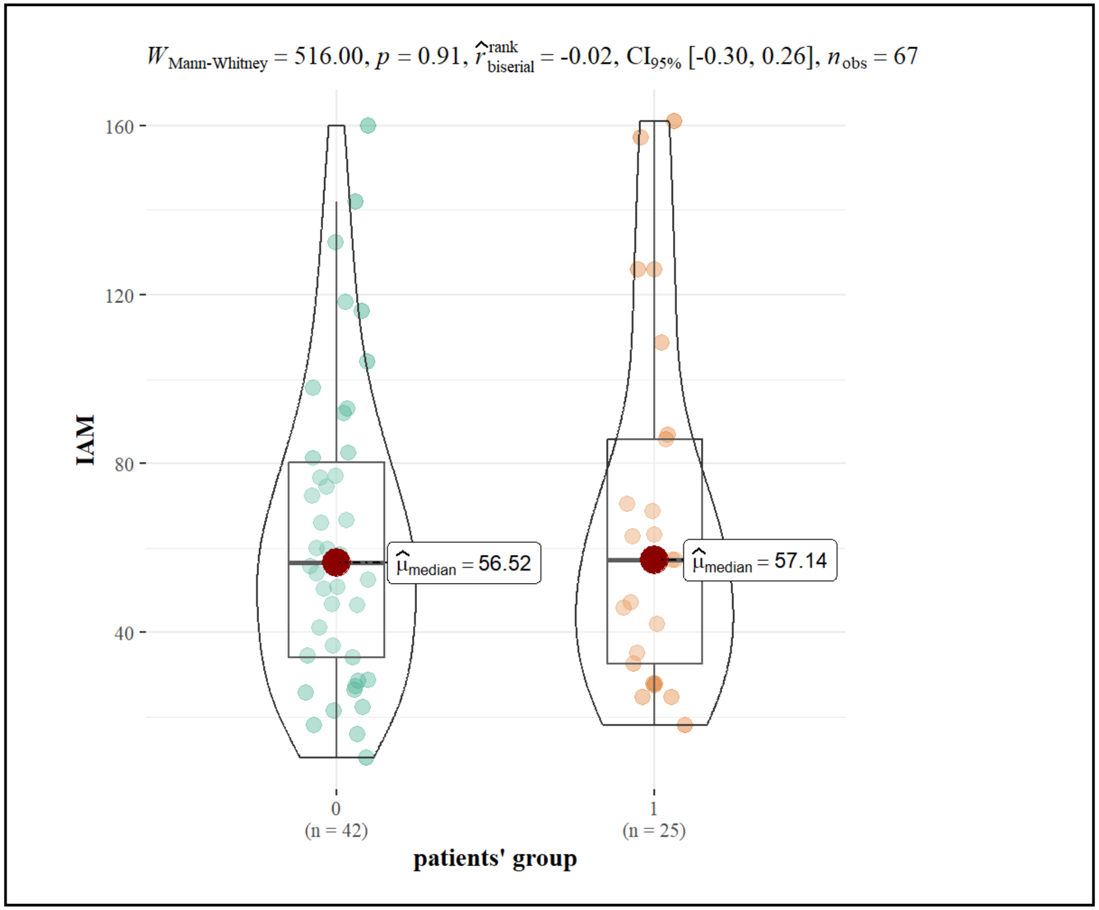

$W_{\text{Mann-Whitney}} = 516.00$, $p = 0.91$, $\hat{r}_{\text{biserial}}^{\text{rank}} = -0.02$, $\text{CI}_{95\%}$ [-0.30, 0.26], $n_{\text{obs}} = 67$
IAM
160
120
80
40
$\hat{\mu}_{\text{median}} = 56.52$
$\hat{\mu}_{\text{median}} = 57.14$
0
(n = 42)
1
(n = 25)
patients' group

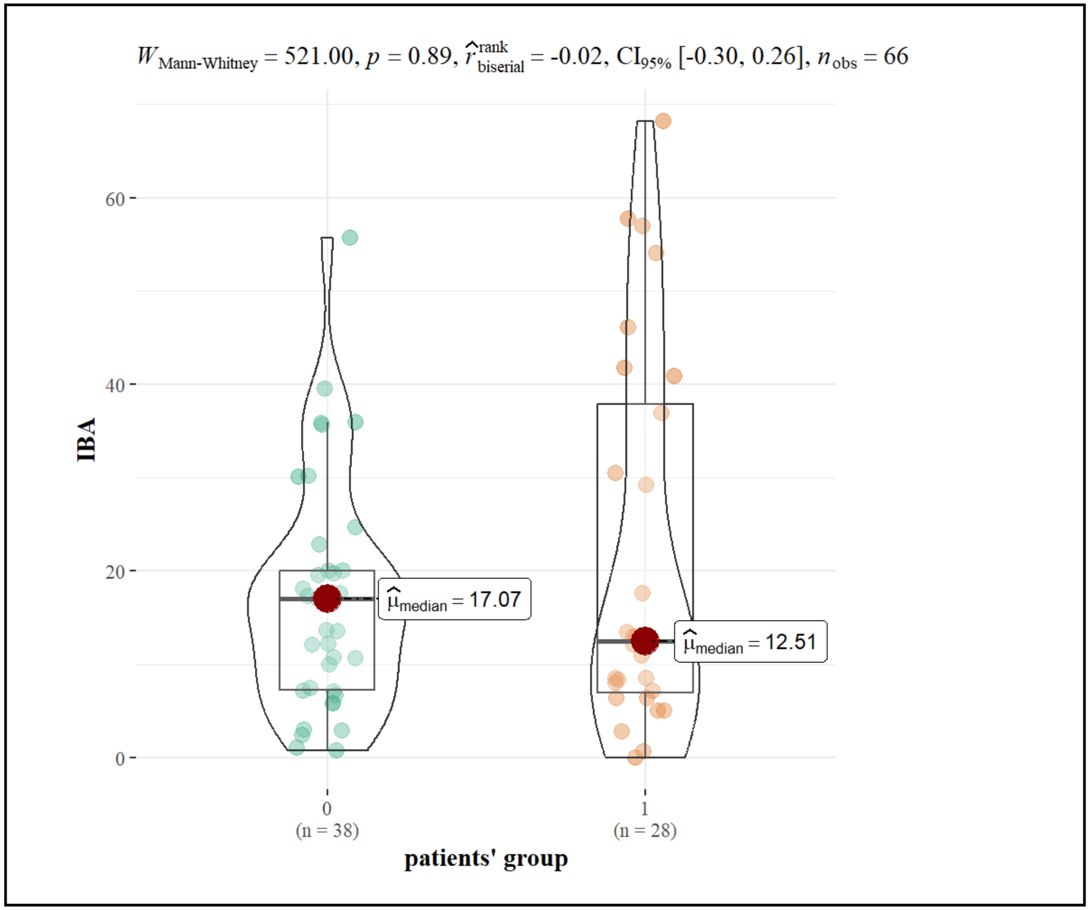
$W_{\text{Mann-Whitney}} = 521.00$, $p = 0.89$, $\hat{r}^{\text{rank}}_{\text{biserial}} = -0.02$, $\text{CI}_{95\%}$ [-0.30, 0.26], $n_{\text{obs}} = 66$
IBA
60
40
20
0
$\hat{\mu}_{\text{median}} = 17.07$
$\hat{\mu}_{\text{median}} = 12.51$
0
(n = 38)
1
(n = 28)
patients' group

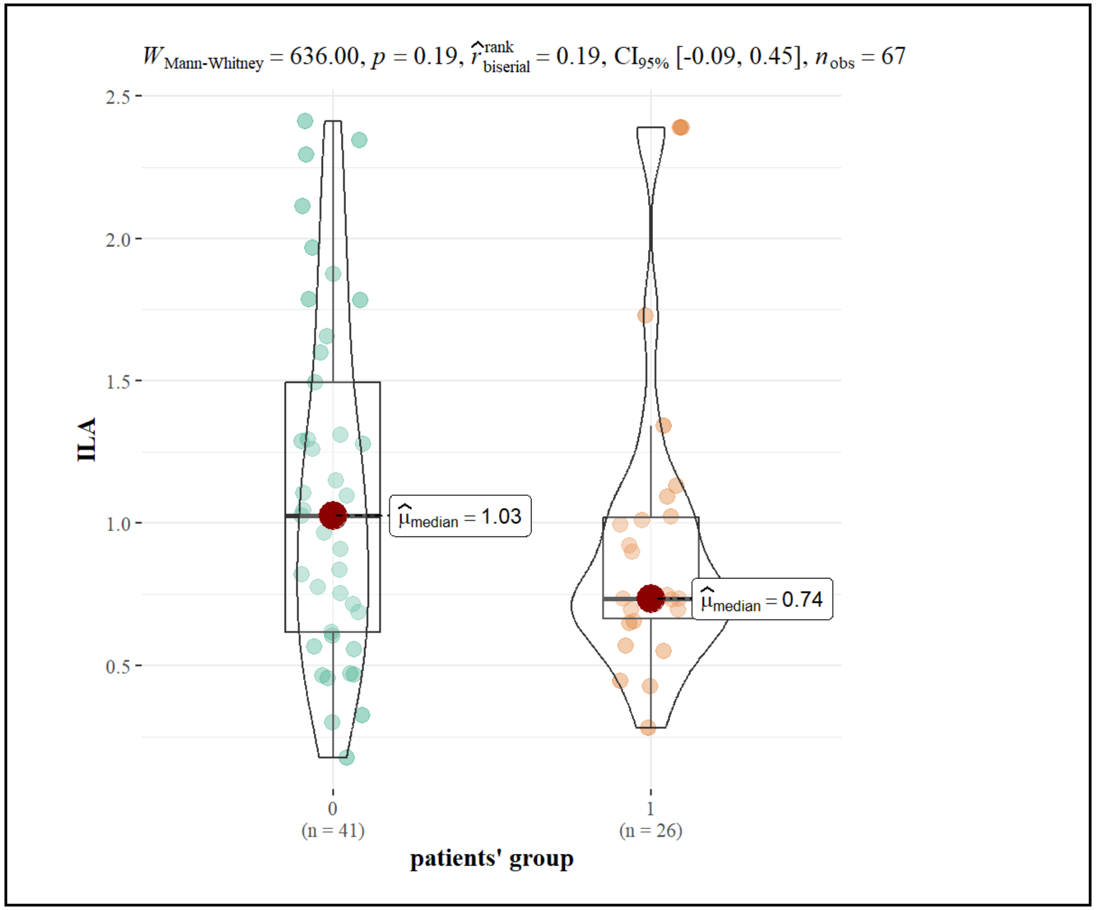

$W_{\text{Mann-Whitney}} = 636.00$, $p = 0.19$, $\hat{r}^{\text{rank}}_{\text{biserial}} = 0.19$, $\text{CI}_{95\%}$ [-0.09, 0.45], $n_{\text{obs}} = 67$
2.5
2.0
1.5
1.0
0.5
ILA
$\hat{\mu}_{\text{median}} = 1.03$
$\hat{\mu}_{\text{median}} = 0.74$
0
(n = 41)
1
(n = 26)
patients' group

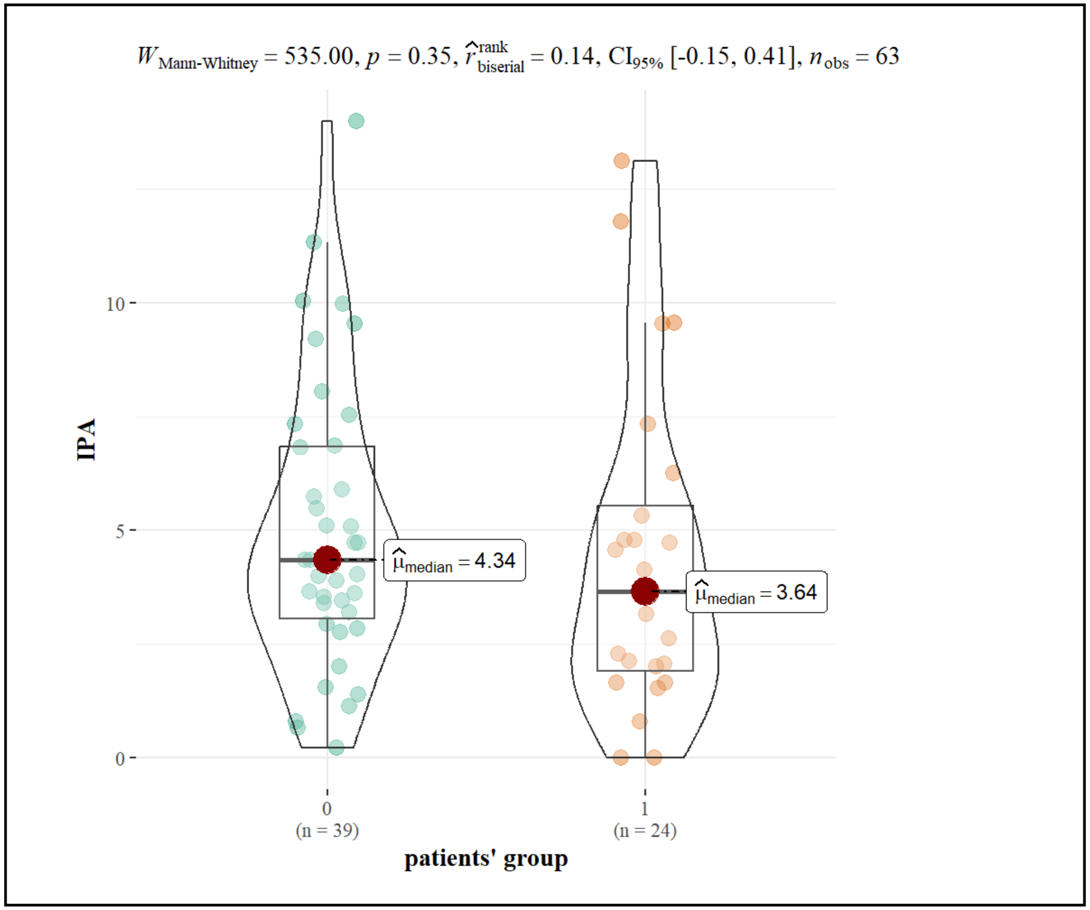

$W_{\text{Mann-Whitney}} = 535.00$, $p = 0.35$, $\hat{r}^{\text{rank}}_{\text{biserial}} = 0.14$, $\text{CI}_{95\%}$ [-0.15, 0.41], $n_{\text{obs}} = 63$
IPA
10
5
0
$\hat{\mu}_{\text{median}} = 4.34$
$\hat{\mu}_{\text{median}} = 3.64$
0
(n = 39)
1
(n = 24)
patients' group

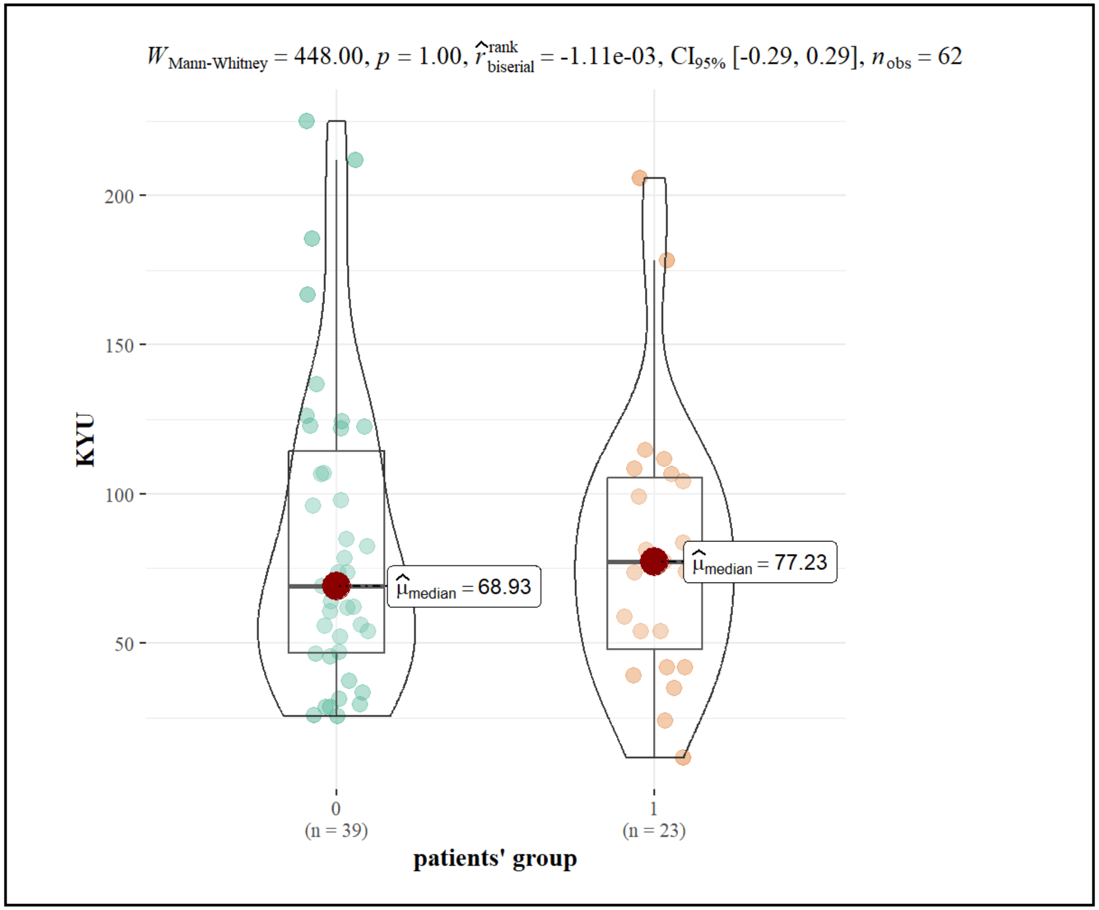

$W_{\text{Mann-Whitney}}$ = 448.00, p = 1.00, $\hat{r}_{\text{biserial}}^{\text{rank}}$ = -1.11e-03, $\text{CI}_{95\%}$ [-0.29, 0.29], n_{obs} = 62
KYU
200
150
100
50
$\hat{\mu}_{\text{median}}$ = 68.93
$\hat{\mu}_{\text{median}}$ = 77.23
0
(n = 39)
1
(n = 23)
patients' group

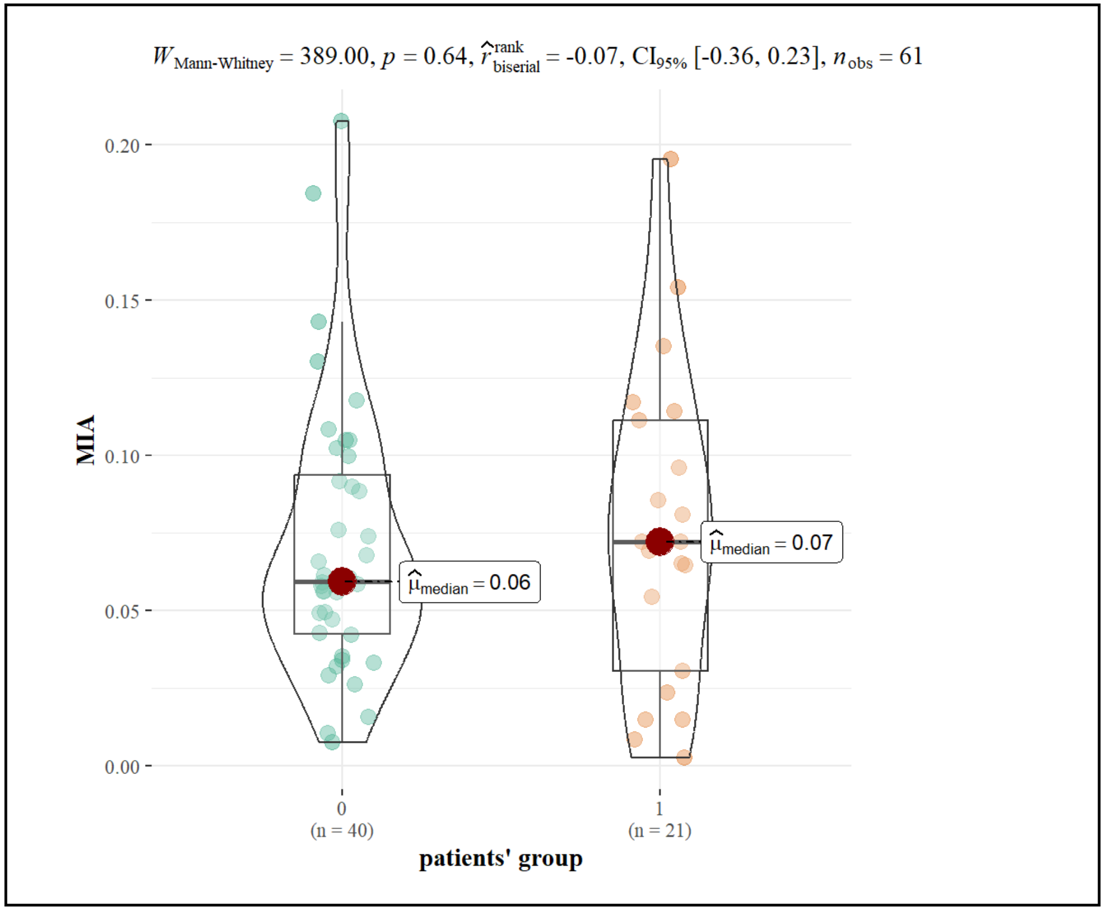

$W_{\text{Mann-Whitney}} = 389.00$, $p = 0.64$, $\hat{r}_{\text{biserial}}^{\text{rank}} = -0.07$, $\text{CI}_{95\%}$ [-0.36, 0.23], $n_{\text{obs}} = 61$
MIA
0.20
0.15
0.10
0.05
0.00
$\hat{\mu}_{\text{median}} = 0.06$
$\hat{\mu}_{\text{median}} = 0.07$
0
(n = 40)
1
(n = 21)
patients' group

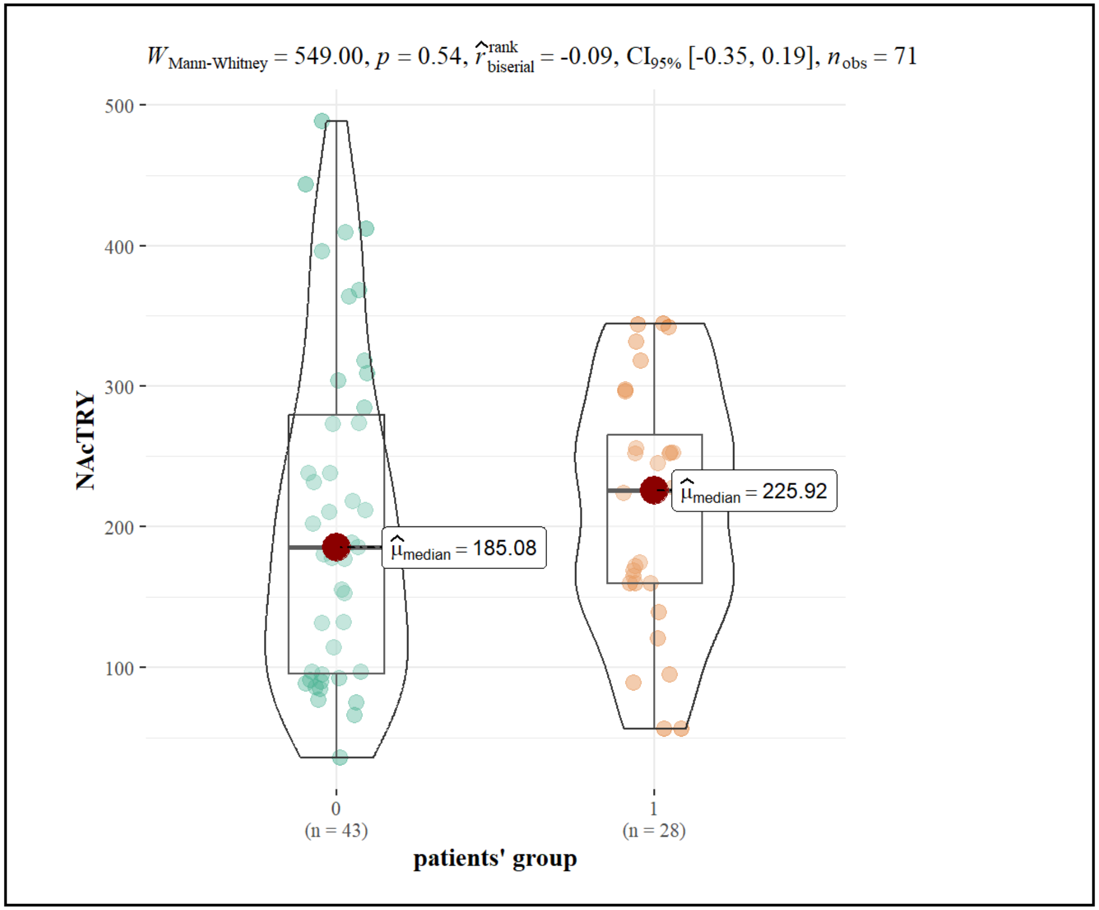

$W_{\text{Mann-Whitney}} = 549.00$, $p = 0.54$, $\hat{r}^{\text{rank}}_{\text{biserial}} = -0.09$, $\text{CI}_{95\%}$ [-0.35, 0.19], $n_{\text{obs}} = 71$
NAcTRY
500
400
300
200
100
$\hat{\mu}_{\text{median}} = 185.08$
$\hat{\mu}_{\text{median}} = 225.92$
0
(n = 43)
1
(n = 28)
patients' group

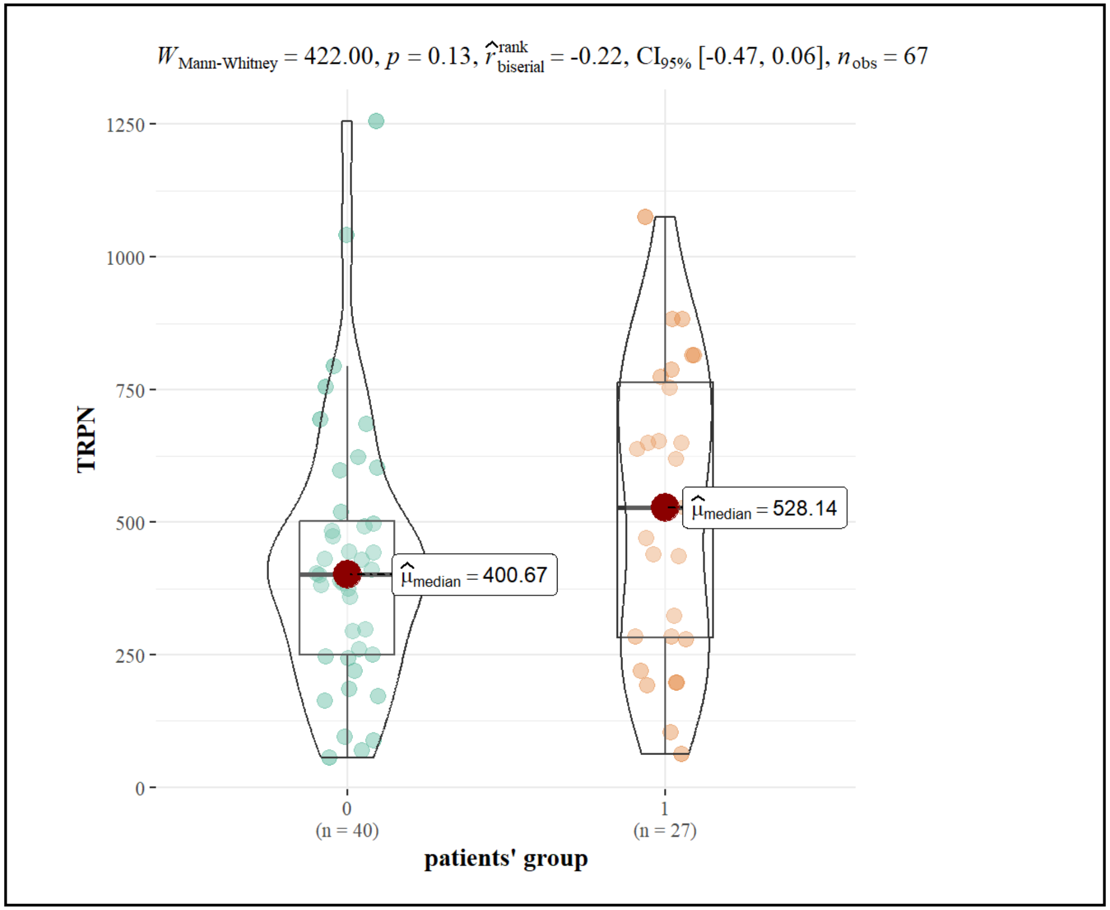
$W_{\text{Mann-Whitney}} = 422.00$, $p = 0.13$, $\hat{r}_{\text{biserial}}^{\text{rank}} = -0.22$, $\text{CI}_{95\%}$ [-0.47, 0.06], $n_{\text{obs}} = 67$
TRPN
1250
1000
750
500
250
0
$\hat{\mu}_{\text{median}} = 400.67$
$\hat{\mu}_{\text{median}} = 528.14$
0
(n = 40)
1
(n = 27)
patients' group

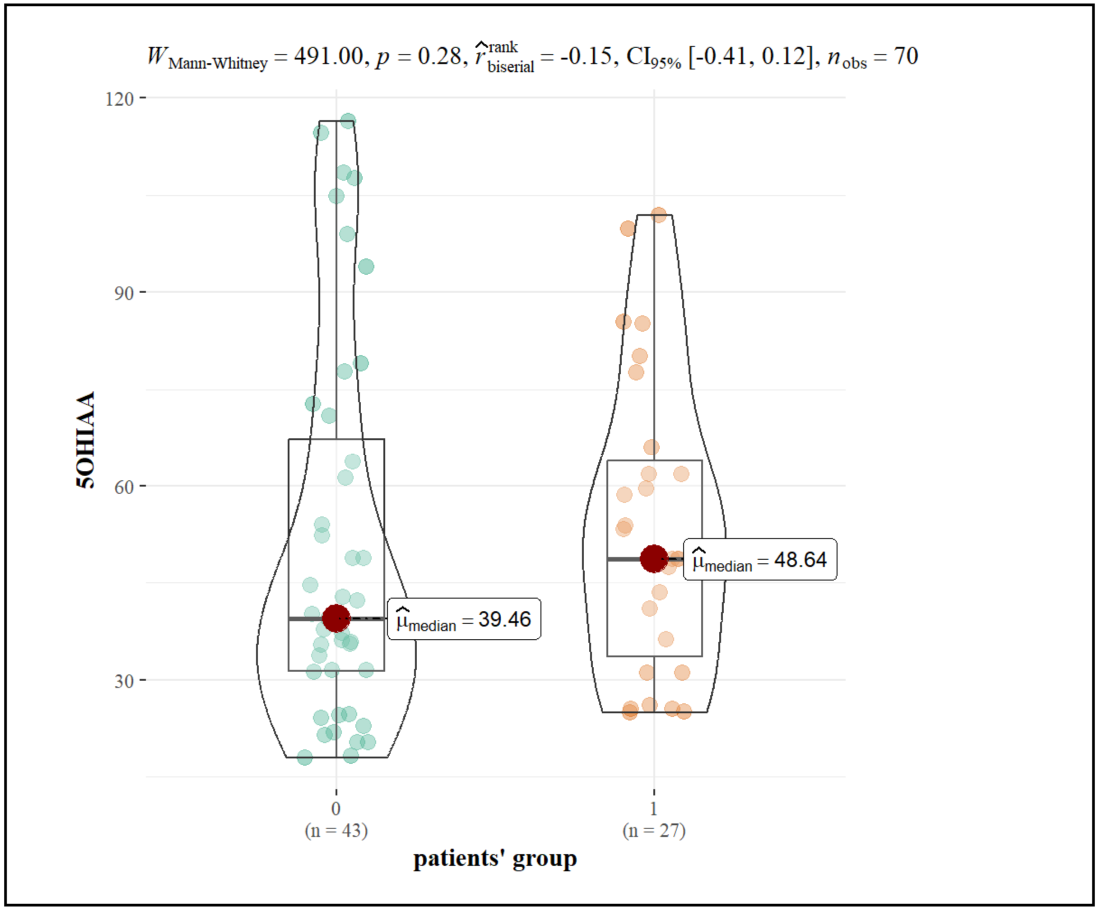

$W_{\text{Mann-Whitney}} = 491.00$, $p = 0.28$, $\hat{r}_{\text{biserial}}^{\text{rank}} = -0.15$, $\text{CI}_{95\%}$ [-0.41, 0.12], $n_{\text{obs}} = 70$
120
90
60
30
5OHIAA
$\hat{\mu}_{\text{median}} = 39.46$
$\hat{\mu}_{\text{median}} = 48.64$
0
(n = 43)
1
(n = 27)
patients' group

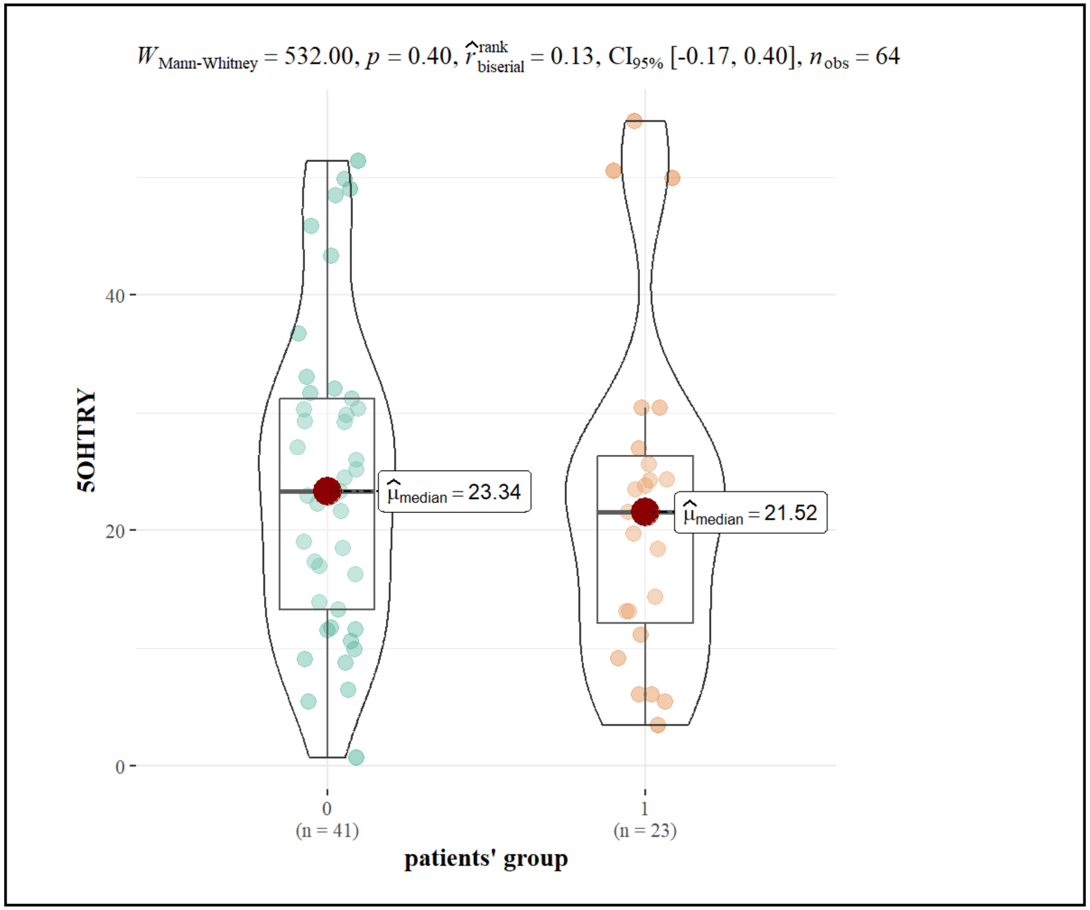

$W_{\text{Mann-Whitney}} = 532.00$, $p = 0.40$, $\hat{r}_{\text{biserial}}^{\text{rank}} = 0.13$, $CI_{95\%}$ [-0.17, 0.40], $n_{\text{obs}} = 64$
5OHTRY
40
20
0
$\hat{\mu}_{\text{median}} = 23.34$
$\hat{\mu}_{\text{median}} = 21.52$
0
(n = 41)
1
(n = 23)
patients' group

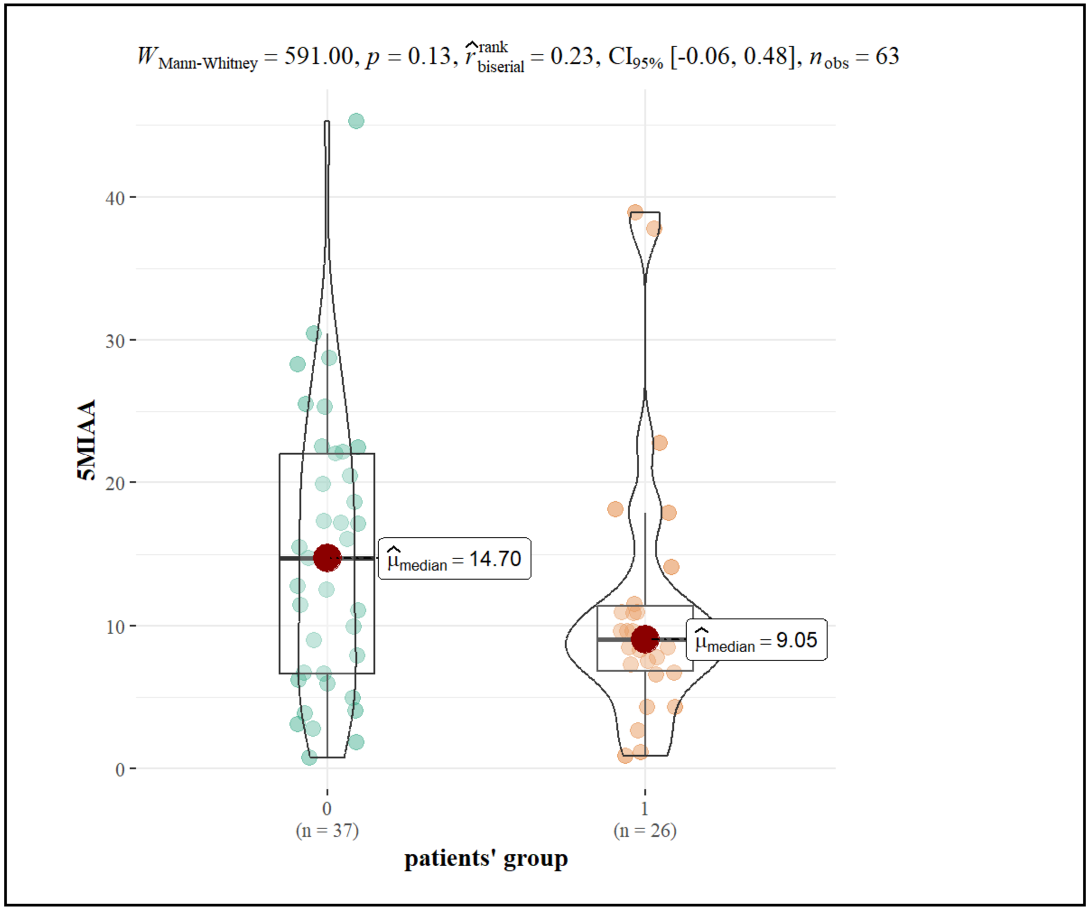

$W_{\text{Mann-Whitney}} = 591.00$, $p = 0.13$, $\hat{r}_{\text{biserial}}^{\text{rank}} = 0.23$, $\text{CI}_{95\%}$ [-0.06, 0.48], $n_{\text{obs}} = 63$
5MIAA
0
10
20
30
40
$\hat{\mu}_{\text{median}} = 14.70$
$\hat{\mu}_{\text{median}} = 9.05$
0
(n = 37)
1
(n = 26)
patients' group

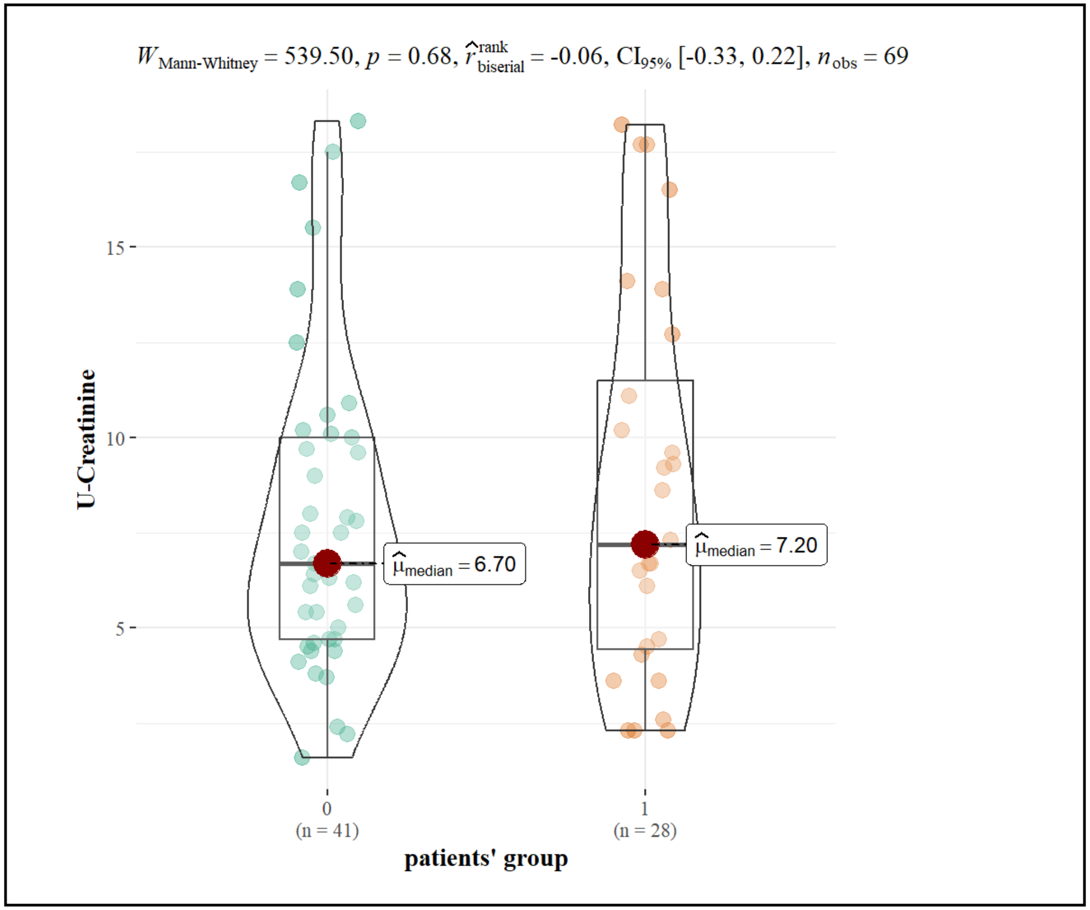

$W_{\text{Mann-Whitney}} = 539.50, p = 0.68, \hat{r}^{\text{rank}}_{\text{biserial}} = -0.06, \text{CI}_{95\%} [-0.33, 0.22], n_{\text{obs}} = 69$
U-Creatinine
15
10
5
$\hat{\mu}_{\text{median}} = 6.70$
$\hat{\mu}_{\text{median}} = 7.20$
0
(n = 41)
1
(n = 28)
patients' group

1-4 ASSOCIATION

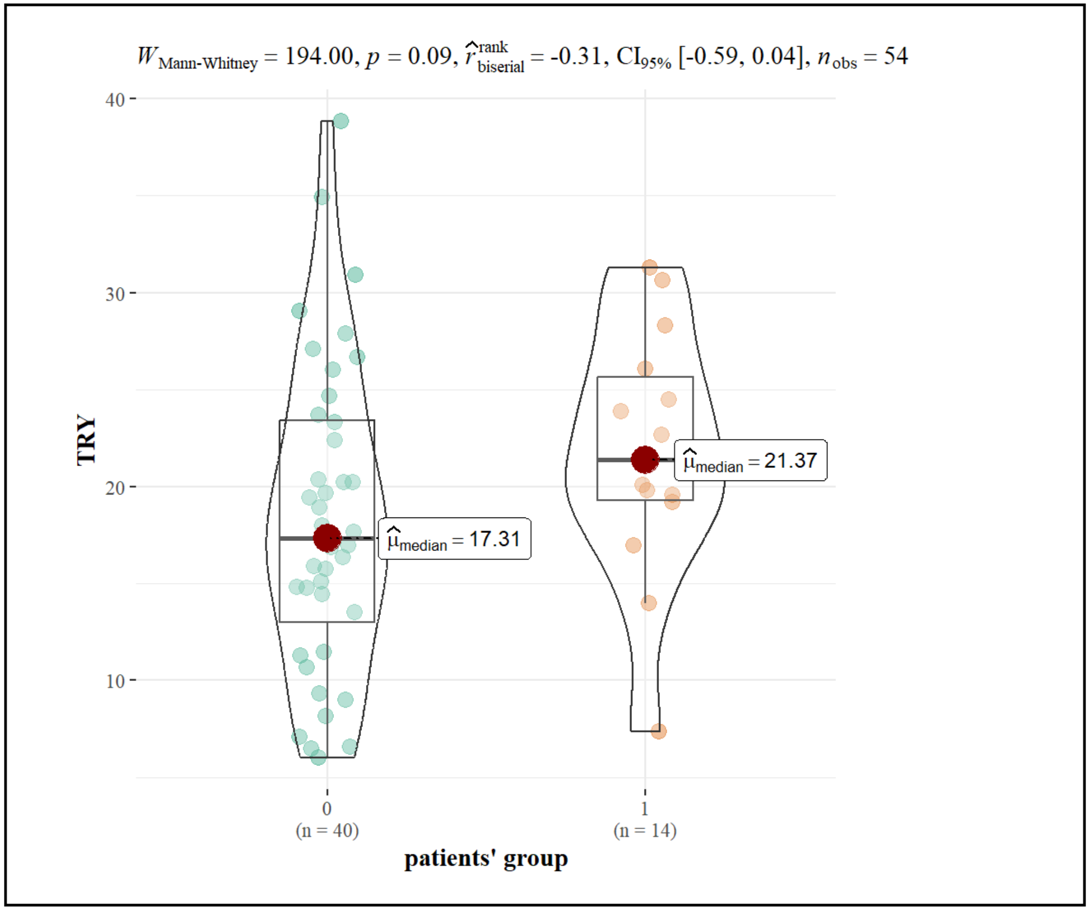

$W_{\text{Mann-Whitney}} = 194.00$, $p = 0.09$, $\hat{r}^{\text{rank}}_{\text{biserial}} = -0.31$, $\text{CI}_{95\%}$ [-0.59, 0.04], $n_{\text{obs}} = 54$
TRY
40
30
20
10
$\hat{\mu}_{\text{median}} = 17.31$
$\hat{\mu}_{\text{median}} = 21.37$
0
(n = 40)
1
(n = 14)
patients' group

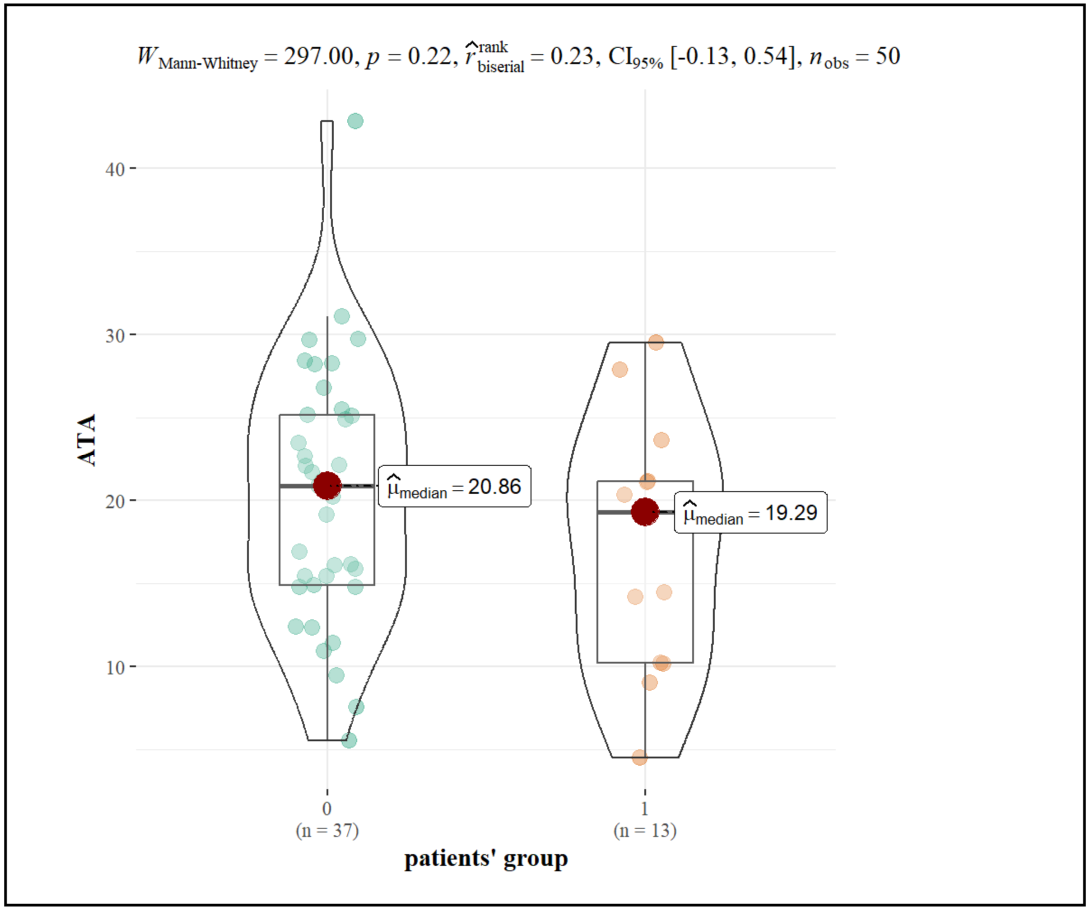

$W_{\text{Mann-Whitney}} = 297.00$, $p = 0.22$, $\hat{r}_{\text{biserial}}^{\text{rank}} = 0.23$, $\text{CI}_{95\%}$ [-0.13, 0.54], $n_{\text{obs}} = 50$
ATA
40
30
20
10
$\hat{\mu}_{\text{median}} = 20.86$
$\hat{\mu}_{\text{median}} = 19.29$
0
(n = 37)
1
(n = 13)
patients' group

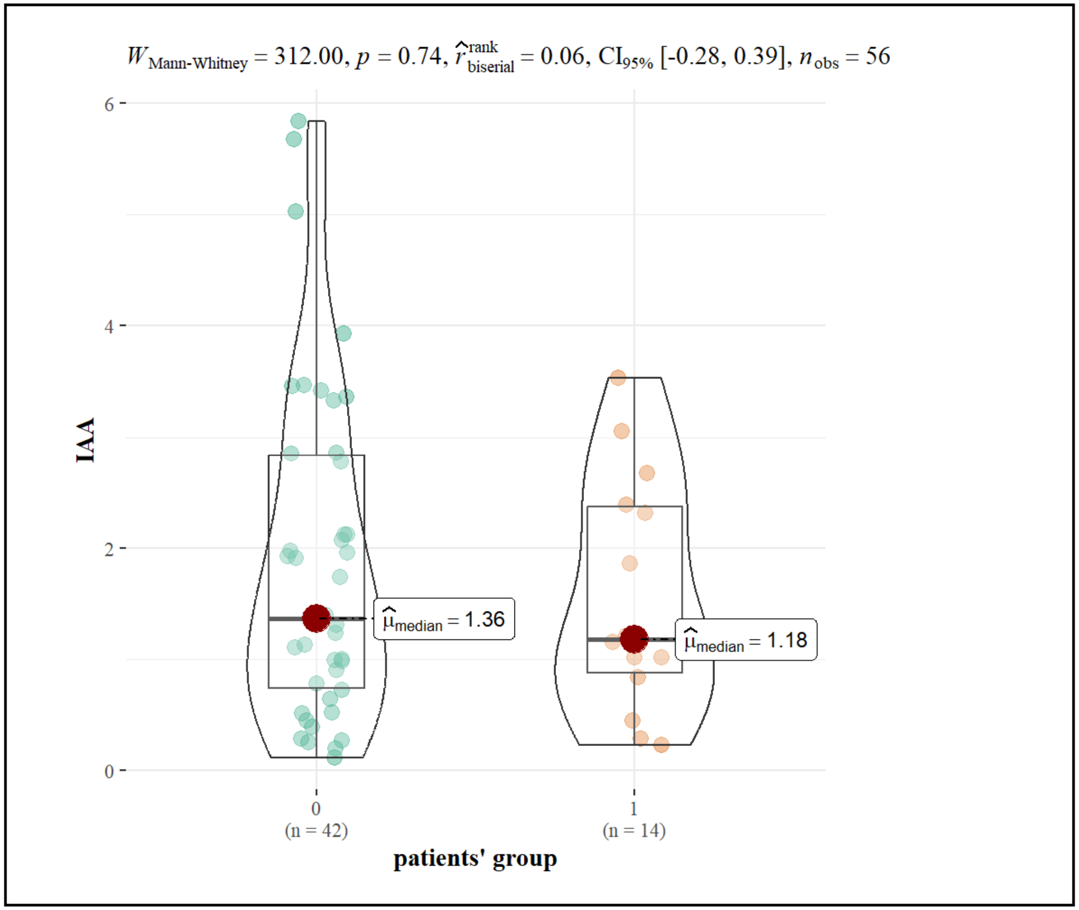

$W_{\text{Mann-Whitney}} = 312.00$, $p = 0.74$, $\hat{r}_{\text{biserial}}^{\text{rank}} = 0.06$, $\text{CI}_{95\%}$ [-0.28, 0.39], $n_{\text{obs}} = 56$
IAA
6
4
2
0
$\hat{\mu}_{\text{median}} = 1.36$
$\hat{\mu}_{\text{median}} = 1.18$
0
(n = 42)
1
(n = 14)
patients' group

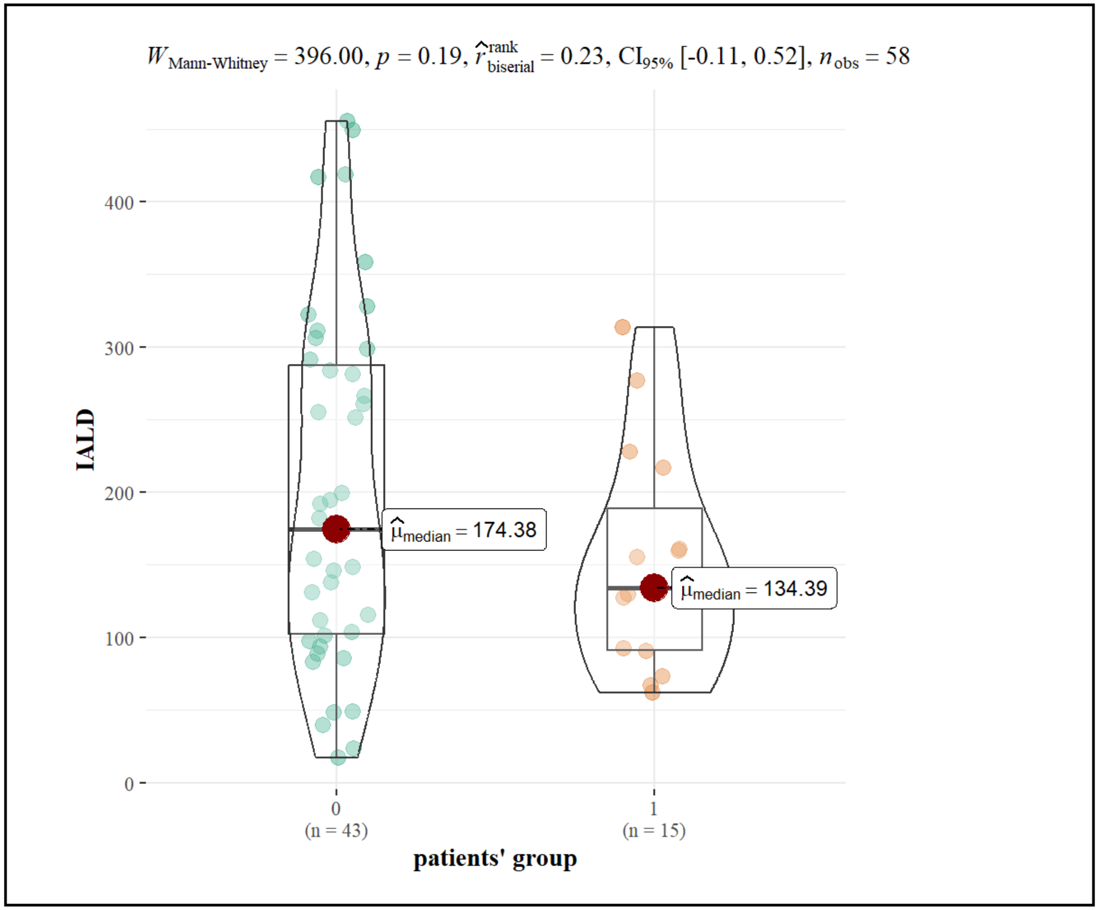

$W_{\text{Mann-Whitney}} = 396.00$, $p = 0.19$, $\hat{r}_{\text{biserial}}^{\text{rank}} = 0.23$, $\text{CI}_{95\%}$ [-0.11, 0.52], $n_{\text{obs}} = 58$
IALD
400
300
200
100
0
$\hat{\mu}_{\text{median}} = 174.38$
$\hat{\mu}_{\text{median}} = 134.39$
0
(n = 43)
1
(n = 15)
patients' group

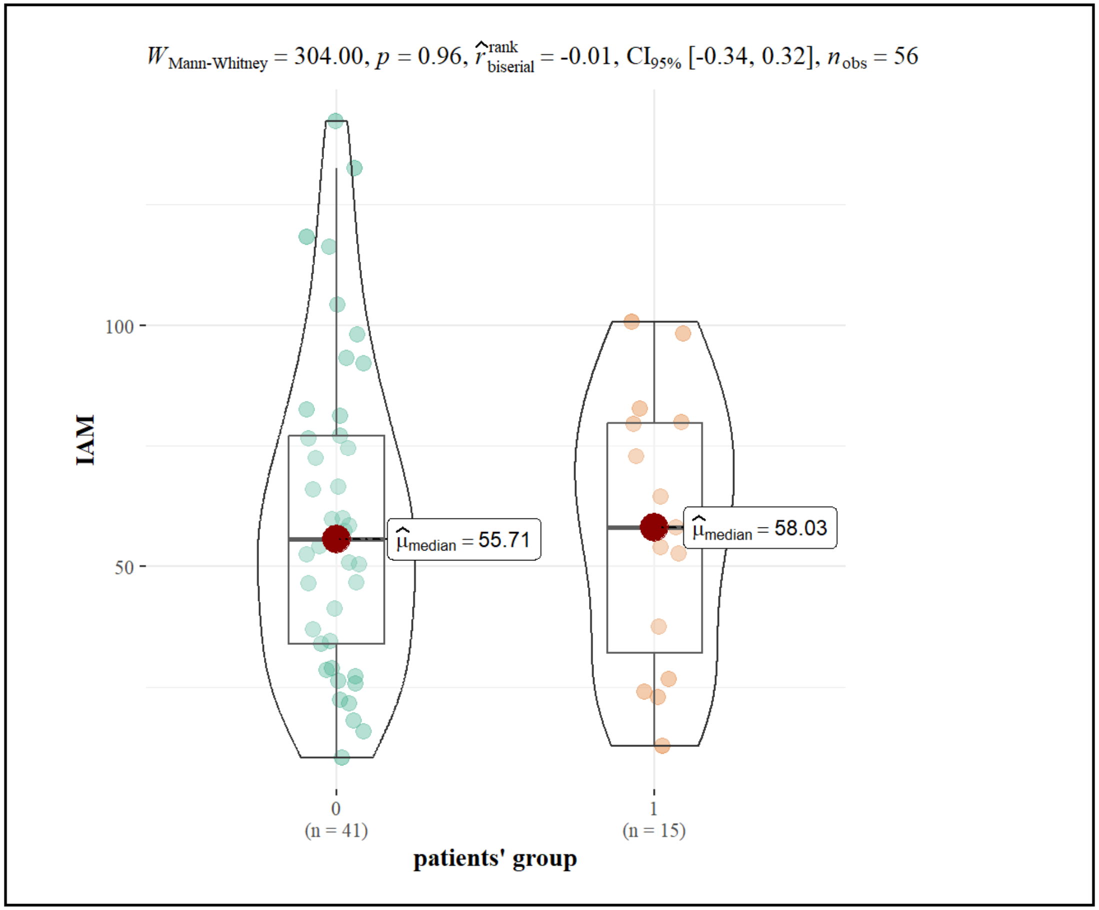

$W_{\text{Mann-Whitney}} = 304.00$, $p = 0.96$, $\hat{r}_{\text{biserial}}^{\text{rank}} = -0.01$, $\text{CI}_{95\%}$ [-0.34, 0.32], $n_{\text{obs}} = 56$
IAM
100
50
$\hat{\mu}_{\text{median}} = 55.71$
$\hat{\mu}_{\text{median}} = 58.03$
0
(n = 41)
1
(n = 15)
patients' group

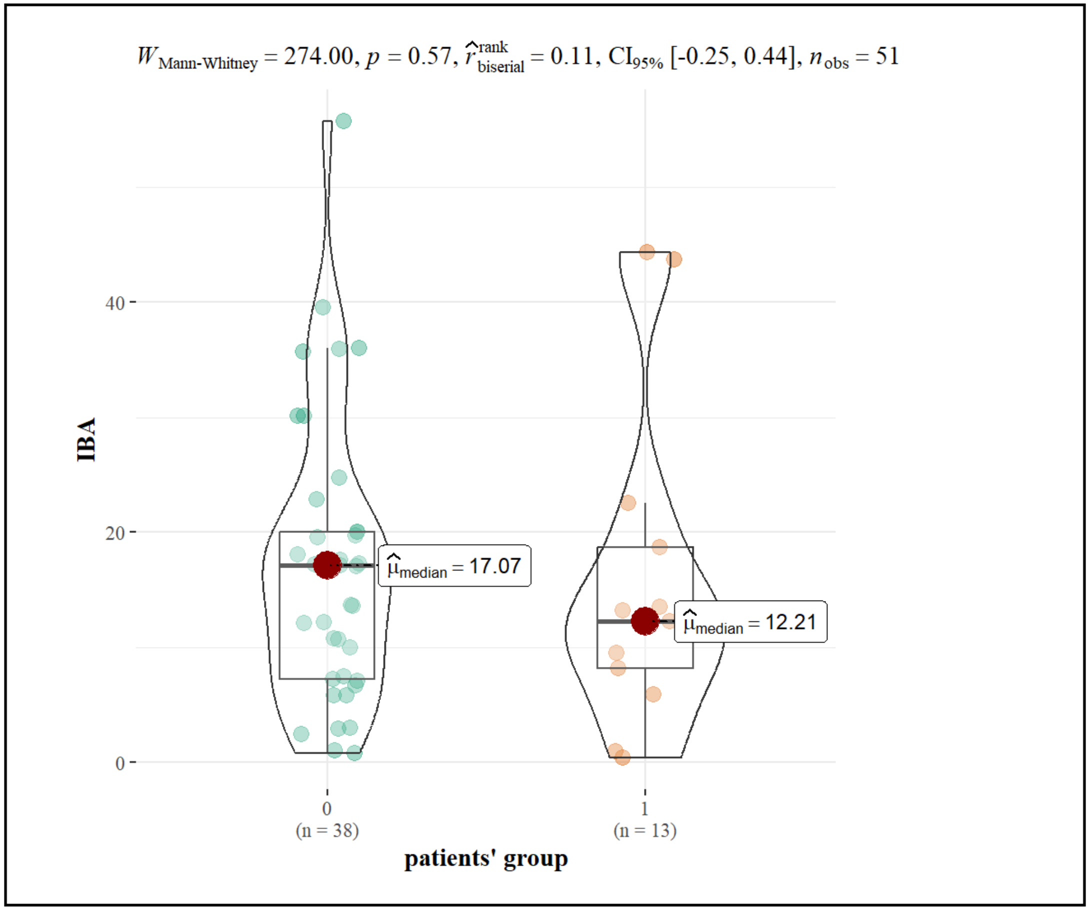
$W_{Mann-Whitney} = 274.00, p = 0.57, \hat{r}^{rank}_{biserial} = 0.11, CI_{95\%}$ [-0.25, 0.44], $n_{obs} = 51$
IBA
40
20
0
$\hat{\mu}_{median} = 17.07$
$\hat{\mu}_{median} = 12.21$
0
(n = 38)
1
(n = 13)
patients' group

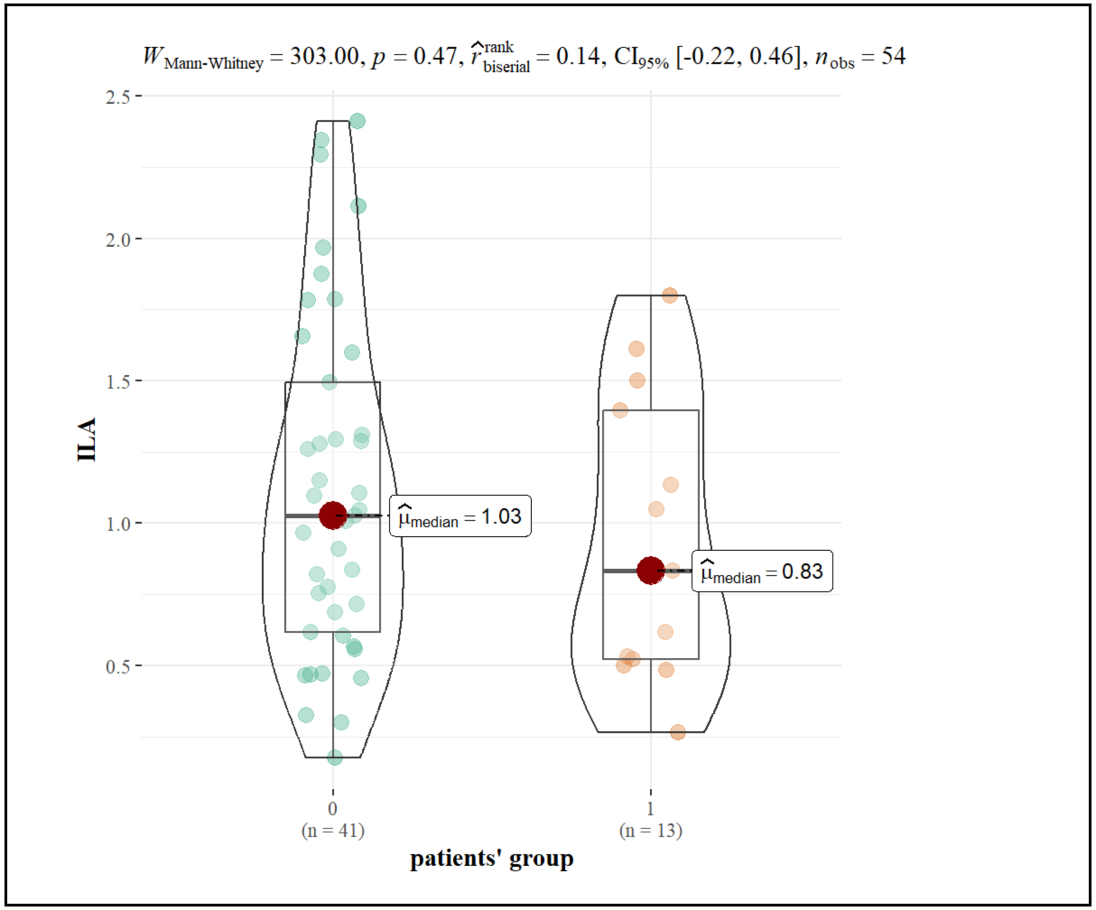

$W_{\text{Mann-Whitney}} = 303.00$, $p = 0.47$, $\hat{r}^{\text{rank}}_{\text{biserial}} = 0.14$, $\text{CI}_{95\%}$ [-0.22, 0.46], $n_{\text{obs}} = 54$
2.5
2.0
1.5
1.0
0.5
ILA
$\hat{\mu}_{\text{median}} = 1.03$
$\hat{\mu}_{\text{median}} = 0.83$
0
(n = 41)
1
(n = 13)
patients' group

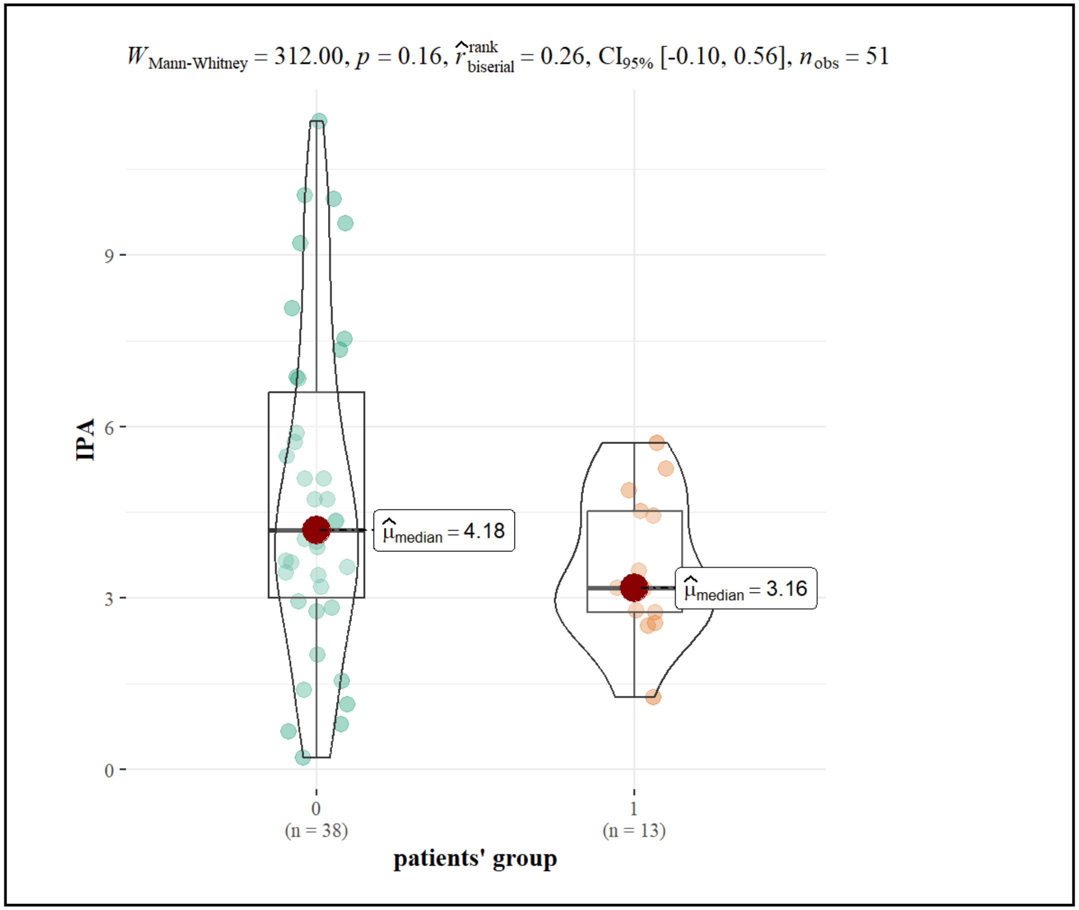

$W_{\text{Mann-Whitney}} = 312.00$, $p = 0.16$, $\hat{r}_{\text{biserial}}^{\text{rank}} = 0.26$, $\text{CI}_{95\%}$ [-0.10, 0.56], $n_{\text{obs}} = 51$
IPA
$\hat{\mu}_{\text{median}} = 4.18$
$\hat{\mu}_{\text{median}} = 3.16$
0
(n = 38)
1
(n = 13)
patients' group

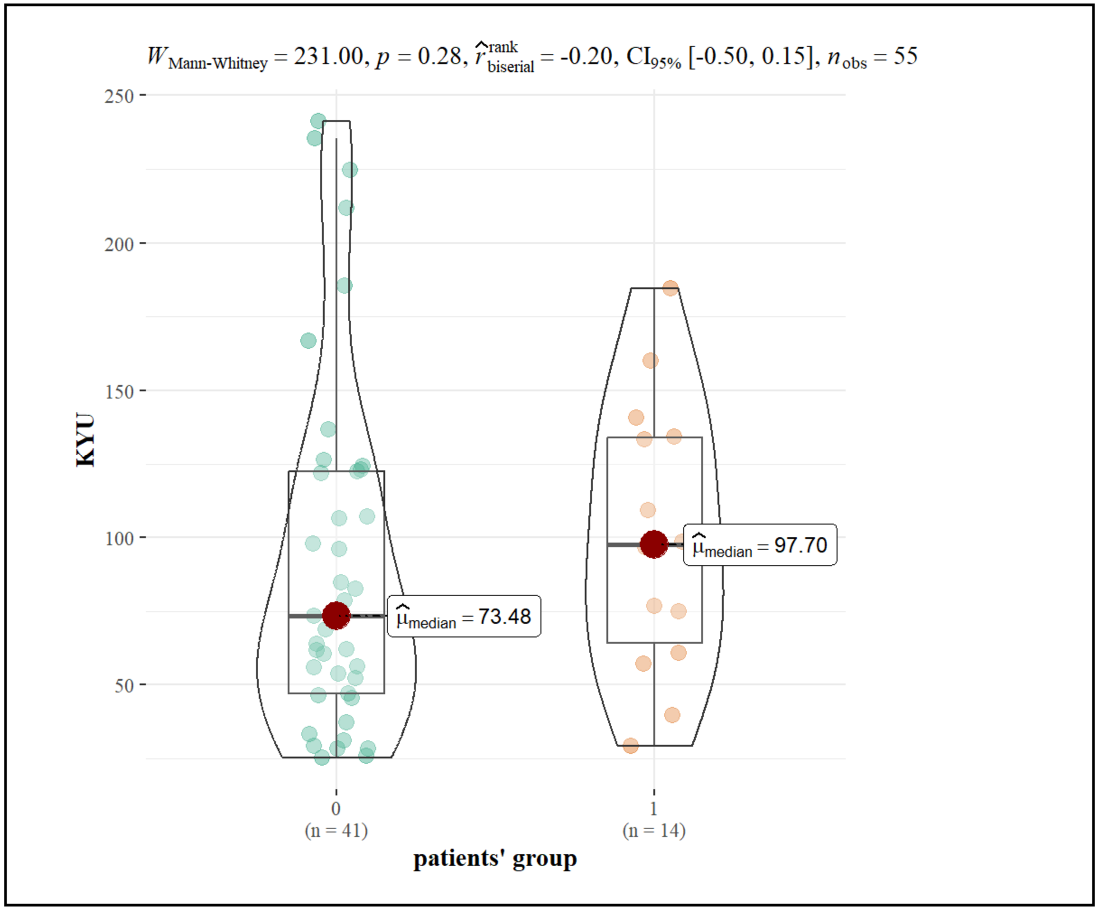

$W_{\text{Mann-Whitney}} = 231.00$, $p = 0.28$, $\hat{r}^{\text{rank}}_{\text{biserial}} = -0.20$, $\text{CI}_{95\%}$ [-0.50, 0.15], $n_{\text{obs}} = 55$
KYU
250
200
150
100
50
$\hat{\mu}_{\text{median}} = 73.48$
$\hat{\mu}_{\text{median}} = 97.70$
0
(n = 41)
1
(n = 14)
patients' group

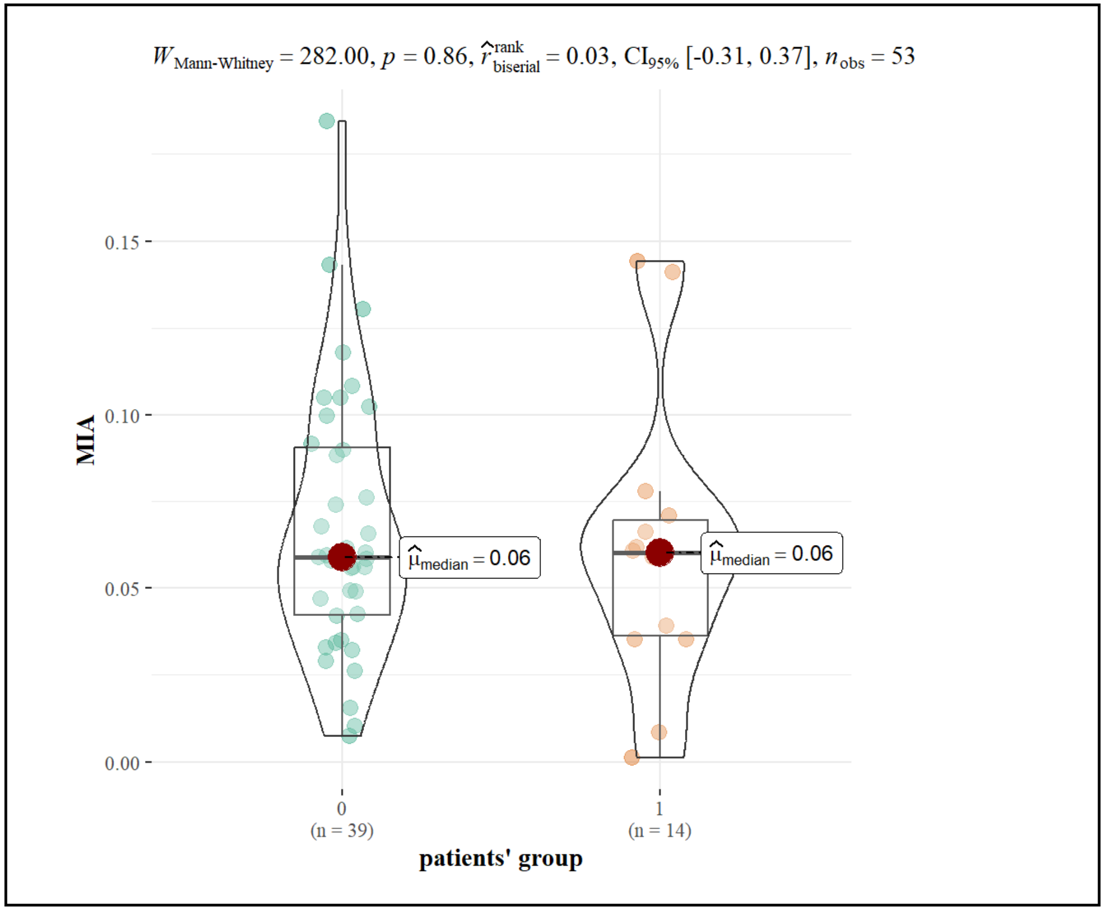
$W_{\text{Mann-Whitney}} = 282.00$, $p = 0.86$, $\hat{r}_{\text{biserial}}^{\text{rank}} = 0.03$, $\text{CI}_{95\%}$ [-0.31, 0.37], $n_{\text{obs}} = 53$
MIA
0.15
0.10
0.05
0.00
$\hat{\mu}_{\text{median}} = 0.06$
$\hat{\mu}_{\text{median}} = 0.06$
0
(n = 39)
1
(n = 14)
patients' group

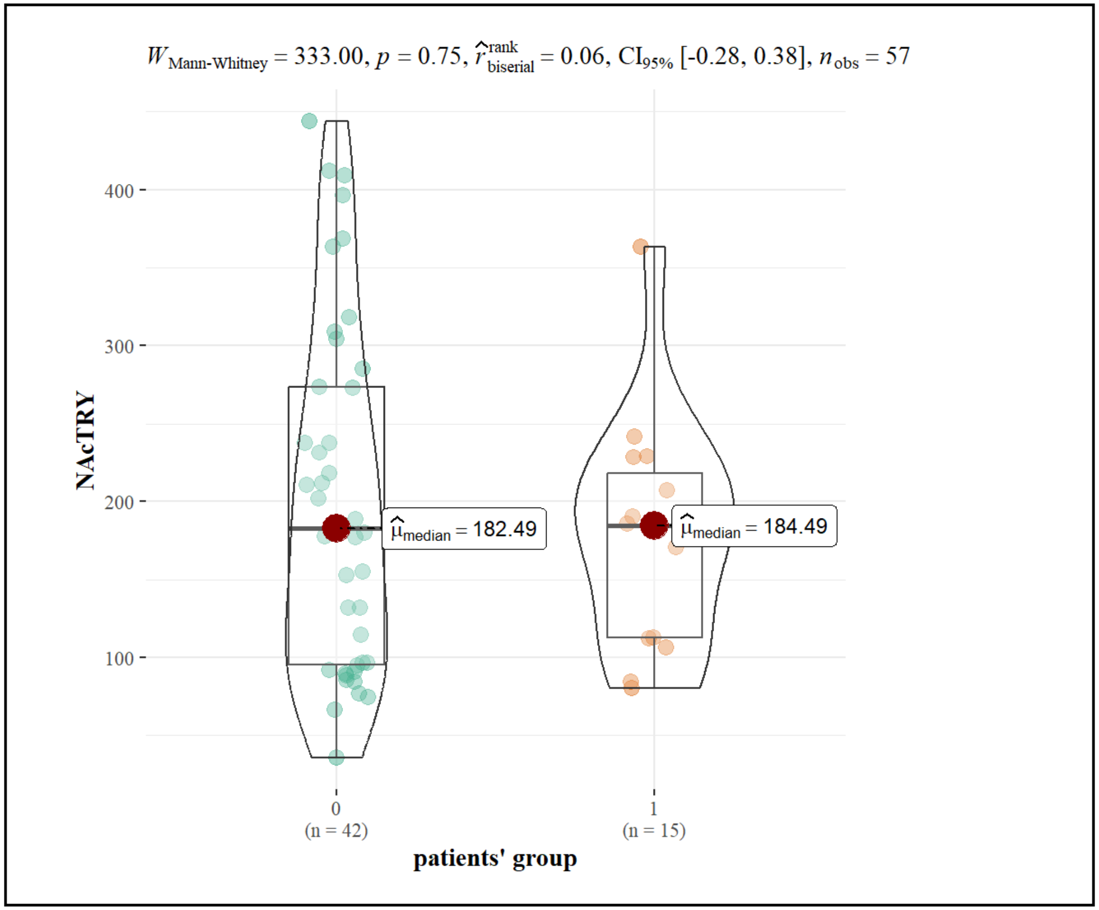

$W_{\text{Mann-Whitney}} = 333.00$, $p = 0.75$, $\hat{r}^{\text{rank}}_{\text{biserial}} = 0.06$, $\text{CI}_{95\%}$ [-0.28, 0.38], $n_{\text{obs}} = 57$
NAcTRY
400
300
200
100
$\hat{\mu}_{\text{median}} = 182.49$
$\hat{\mu}_{\text{median}} = 184.49$
0
(n = 42)
1
(n = 15)
patients' group

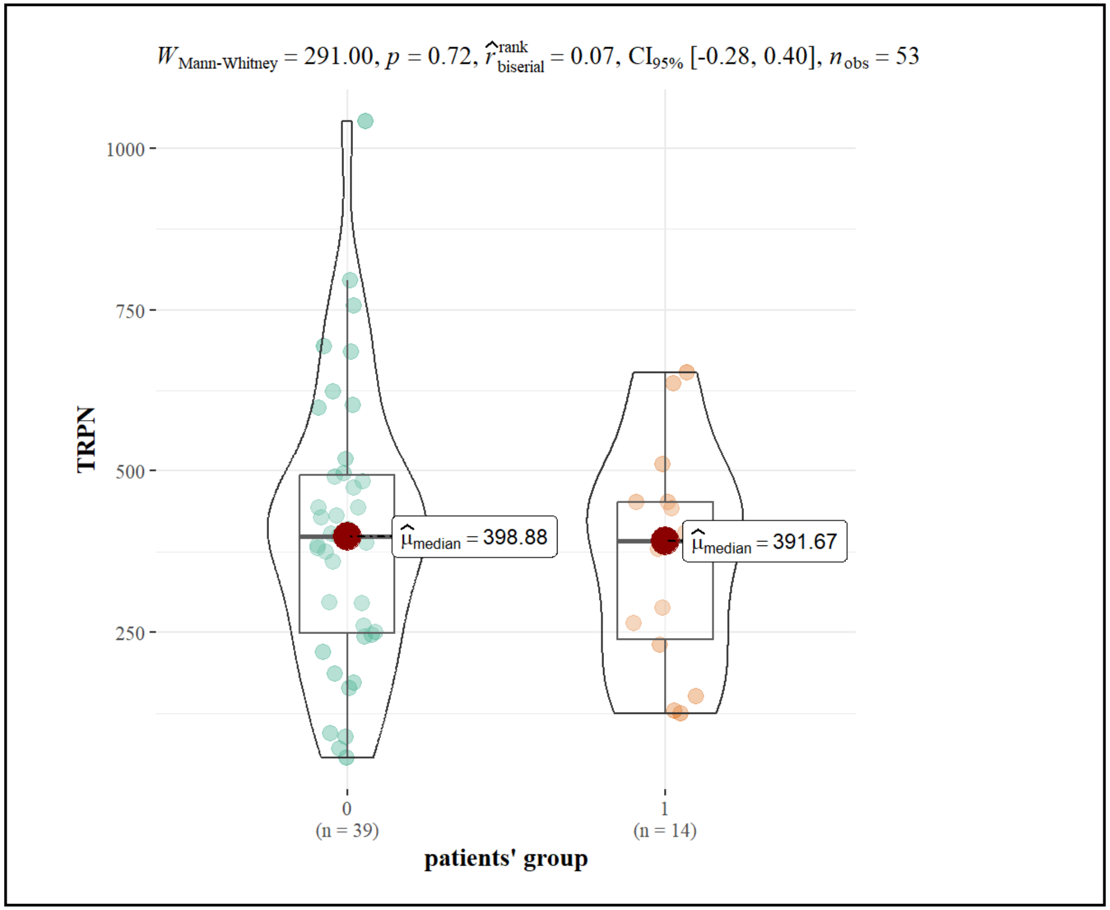

$W_{\text{Mann-Whitney}} = 291.00$, $p = 0.72$, $\widehat{r}_{\text{biserial}}^{\text{rank}} = 0.07$, $\text{CI}_{95\%}$ [-0.28, 0.40], $n_{\text{obs}} = 53$
1000
750
500
250
TRPN
$\widehat{\mu}_{\text{median}} = 398.88$
$\widehat{\mu}_{\text{median}} = 391.67$
0
(n = 39)
1
(n = 14)
patients' group

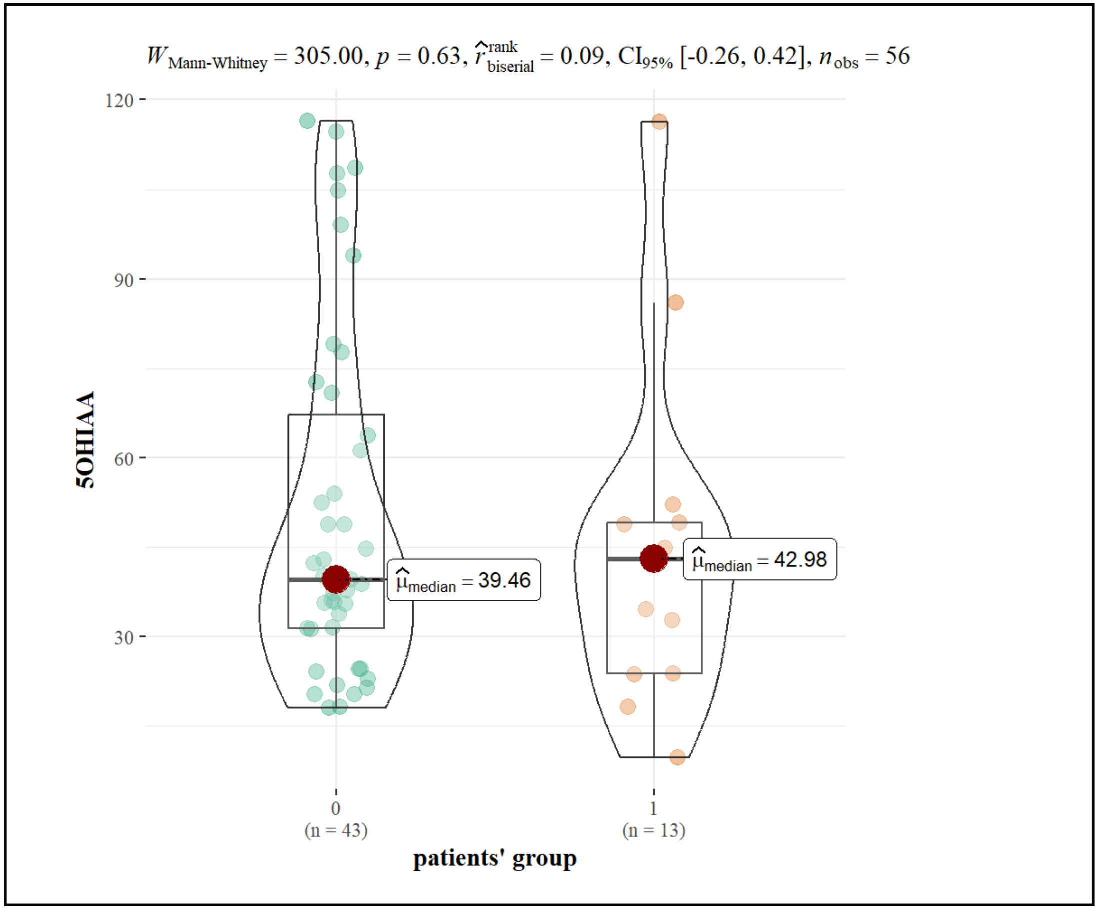

$W_{\text{Mann-Whitney}} = 305.00$, $p = 0.63$, $\hat{r}_{\text{biserial}}^{\text{rank}} = 0.09$, $\text{CI}_{95\%}$ [-0.26, 0.42], $n_{\text{obs}} = 56$
120
90
60
30
5OHIAA
$\hat{\mu}_{\text{median}} = 39.46$
$\hat{\mu}_{\text{median}} = 42.98$
0
(n = 43)
1
(n = 13)
patients' group

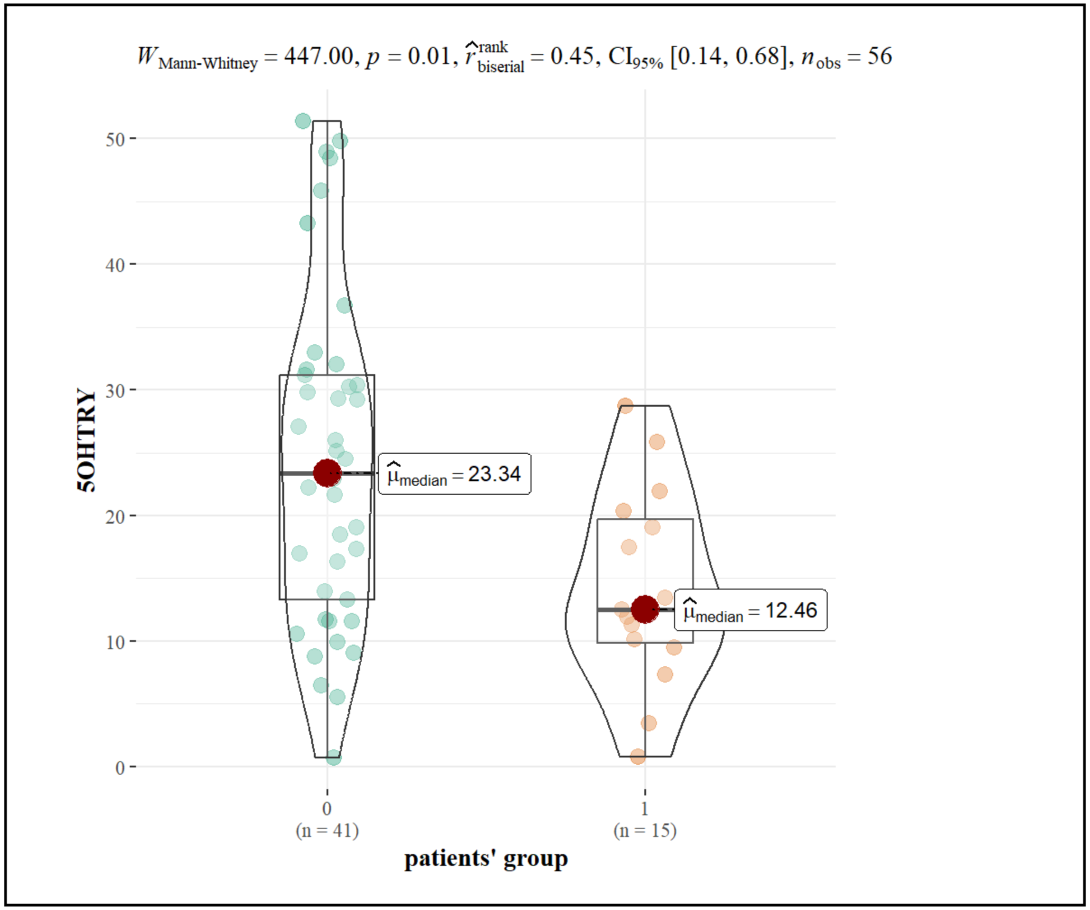

$W_{\text{Mann-Whitney}} = 447.00$, $p = 0.01$, $\widehat{r}_{\text{biserial}}^{\text{rank}} = 0.45$, $\text{CI}_{95\%}$ [0.14, 0.68], $n_{\text{obs}} = 56$
5OHTRY
50
40
30
20
10
0
$\widehat{\mu}_{\text{median}} = 23.34$
$\widehat{\mu}_{\text{median}} = 12.46$
0
(n = 41)
1
(n = 15)
patients' group

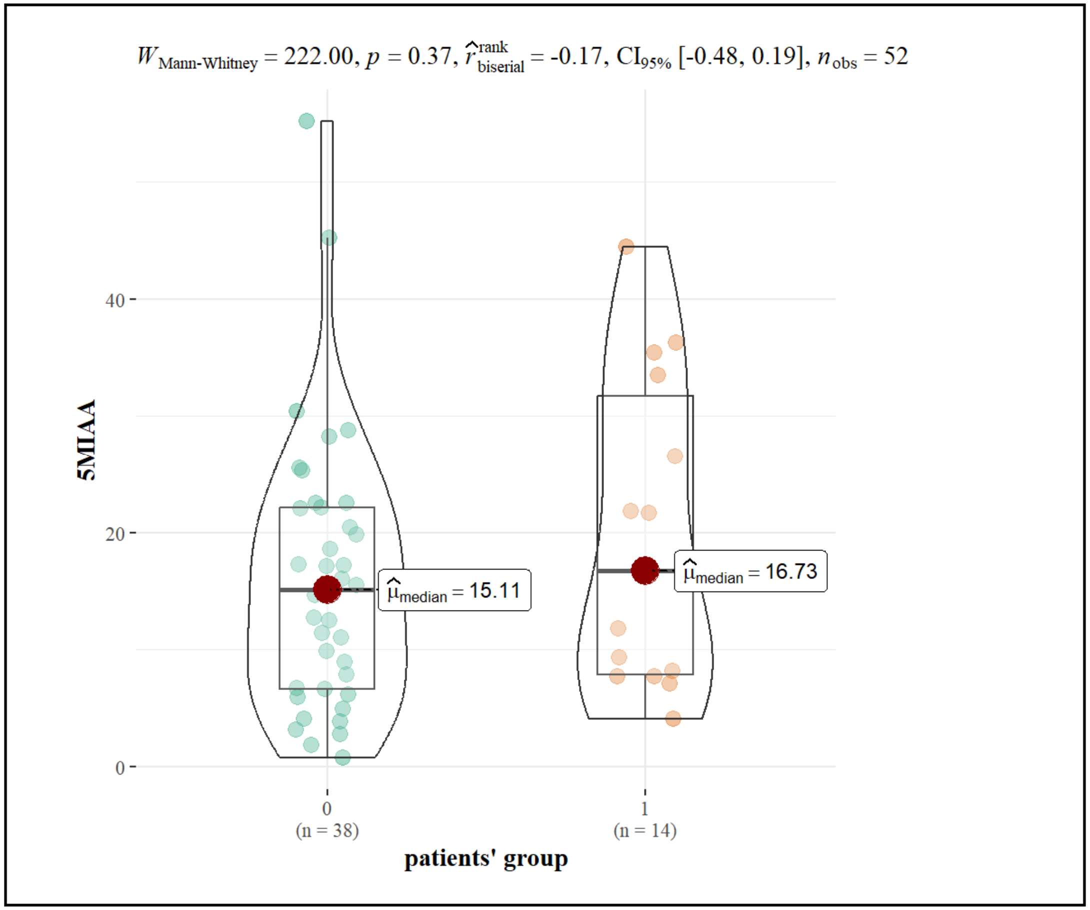

$W_{\text{Mann-Whitney}} = 222.00$, $p = 0.37$, $\hat{r}^{\text{rank}}_{\text{biserial}} = -0.17$, $\text{CI}_{95\%}$ [-0.48, 0.19], $n_{\text{obs}} = 52$
5MIAA
40
20
0
$\hat{\mu}_{\text{median}} = 15.11$
$\hat{\mu}_{\text{median}} = 16.73$
0
(n = 38)
1
(n = 14)
patients' group

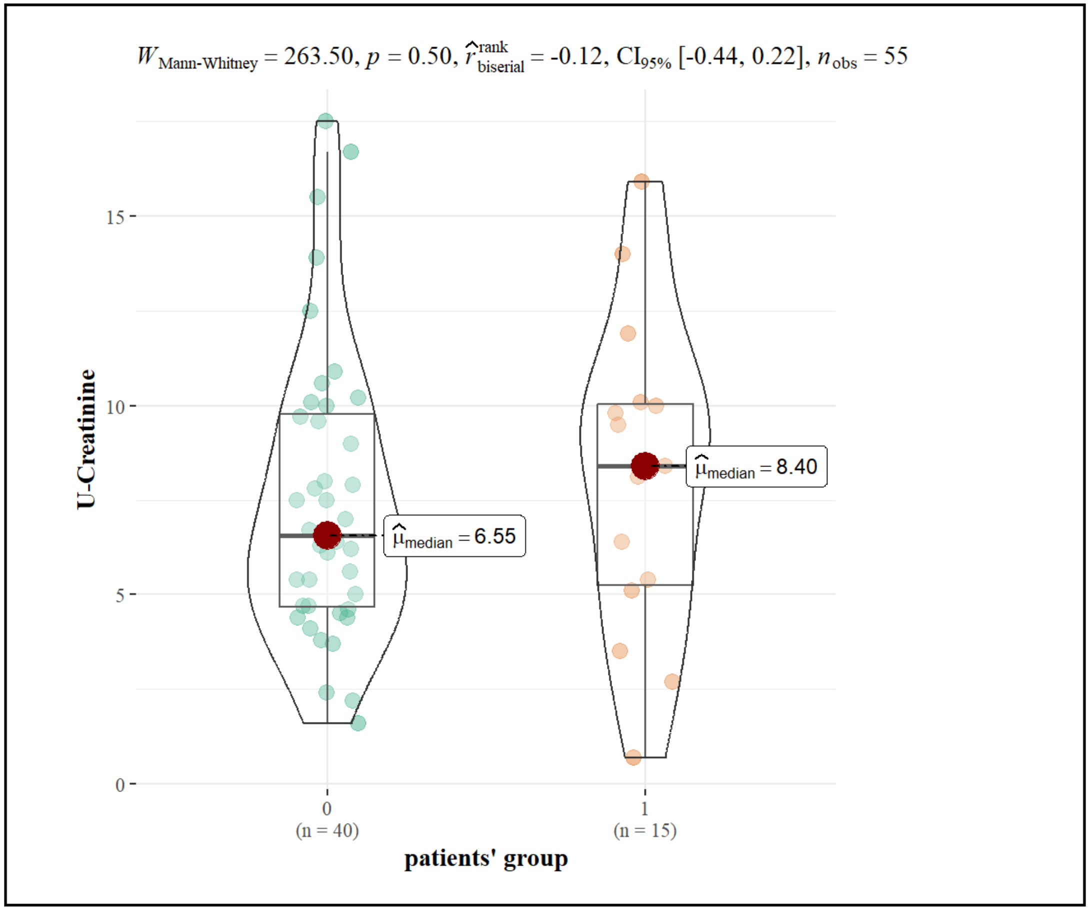

$W_{\text{Mann-Whitney}}$ = 263.50, p = 0.50, $\hat{r}_{\text{biserial}}^{\text{rank}}$ = -0.12, $\text{CI}_{95\%}$ [-0.44, 0.22], n_{obs} = 55
U-Creatinine
15
10
5
0
$\hat{\mu}_{\text{median}}$ = 6.55
$\hat{\mu}_{\text{median}}$ = 8.40
0
(n = 40)
1
(n = 15)
patients' group

3 – 4 ASSOCIATION

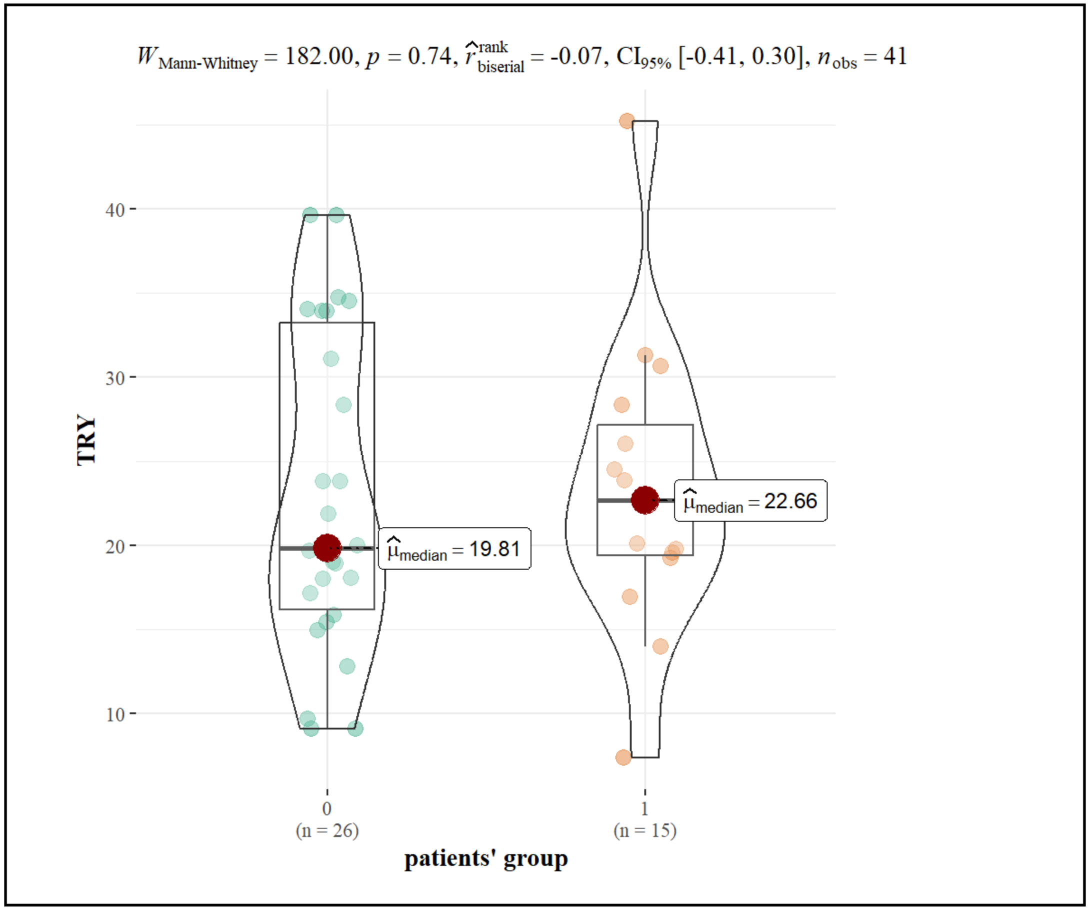

$W_{\text{Mann-Whitney}} = 182.00$, $p = 0.74$, $\hat{r}^{\text{rank}}_{\text{biserial}} = -0.07$, $\text{CI}_{95\%}$ [-0.41, 0.30], $n_{\text{obs}} = 41$
TRY
40
30
20
10
$\hat{\mu}_{\text{median}} = 19.81$
$\hat{\mu}_{\text{median}} = 22.66$
0
(n = 26)
1
(n = 15)
patients' group

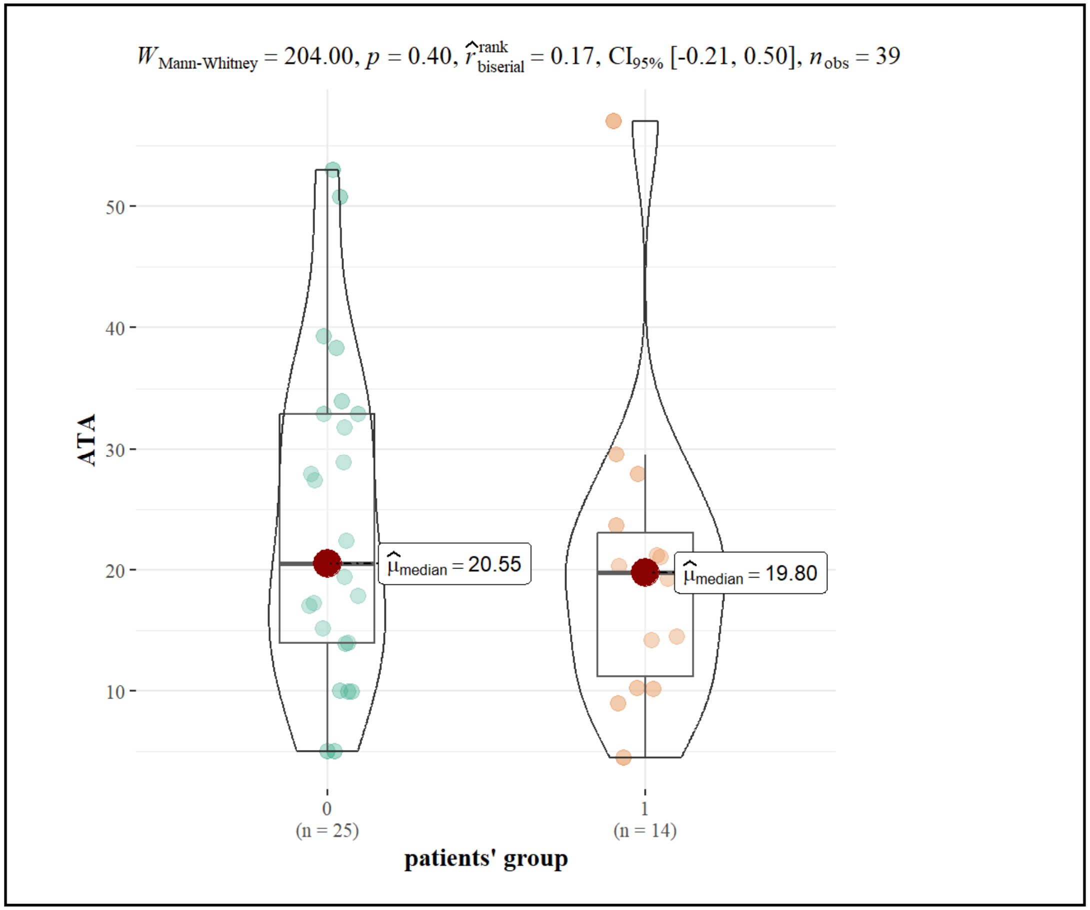

$W_{\text{Mann-Whitney}} = 204.00$, $p = 0.40$, $\widehat{r}_{\text{biserial}}^{\text{rank}} = 0.17$, $\text{CI}_{95\%}$ [-0.21, 0.50], $n_{\text{obs}} = 39$
ATA
50
40
30
20
10
$\widehat{\mu}_{\text{median}} = 20.55$
$\widehat{\mu}_{\text{median}} = 19.80$
0
(n = 25)
1
(n = 14)
patients' group

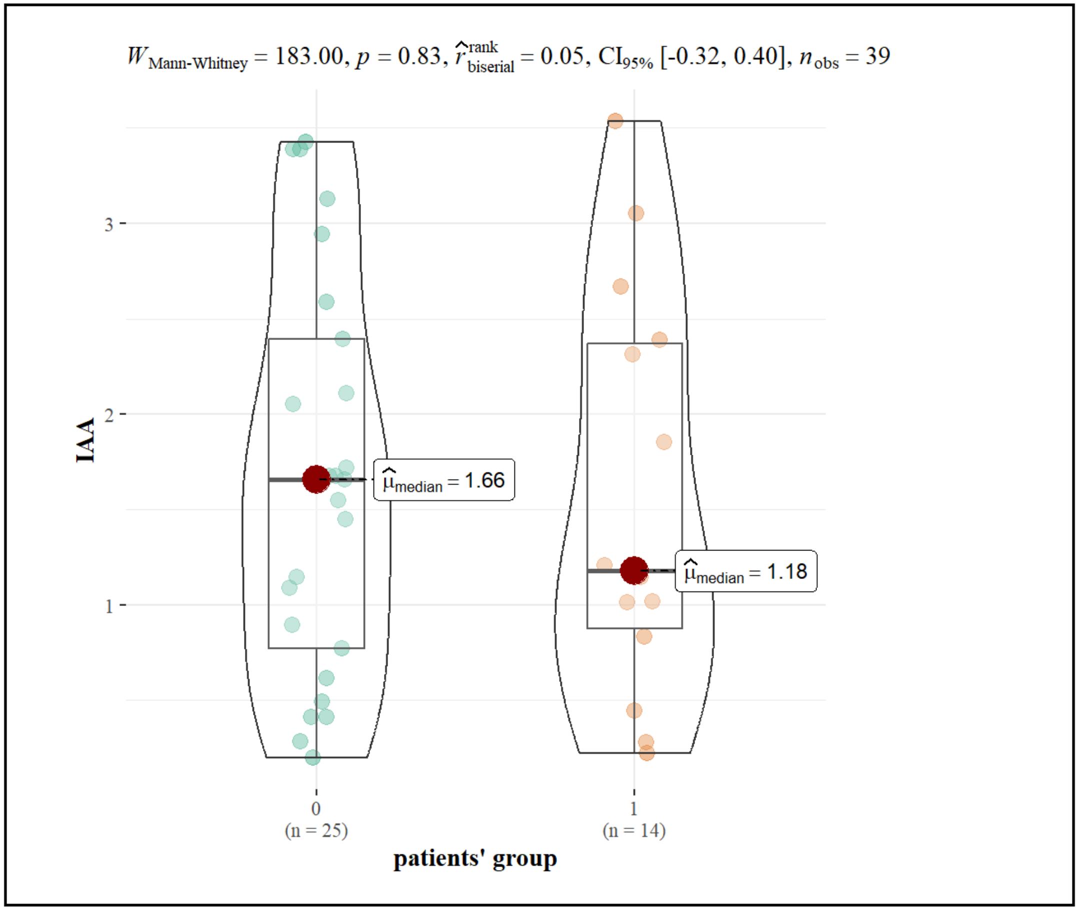
$W_{\text{Mann-Whitney}} = 183.00$, $p = 0.83$, $\hat{r}_{\text{biserial}}^{\text{rank}} = 0.05$, $\text{CI}_{95\%}$ [-0.32, 0.40], $n_{\text{obs}} = 39$
$\hat{\mu}_{\text{median}} = 1.66$
$\hat{\mu}_{\text{median}} = 1.18$
IAA
3
2
1
0
(n = 25)
1
(n = 14)
patients' group

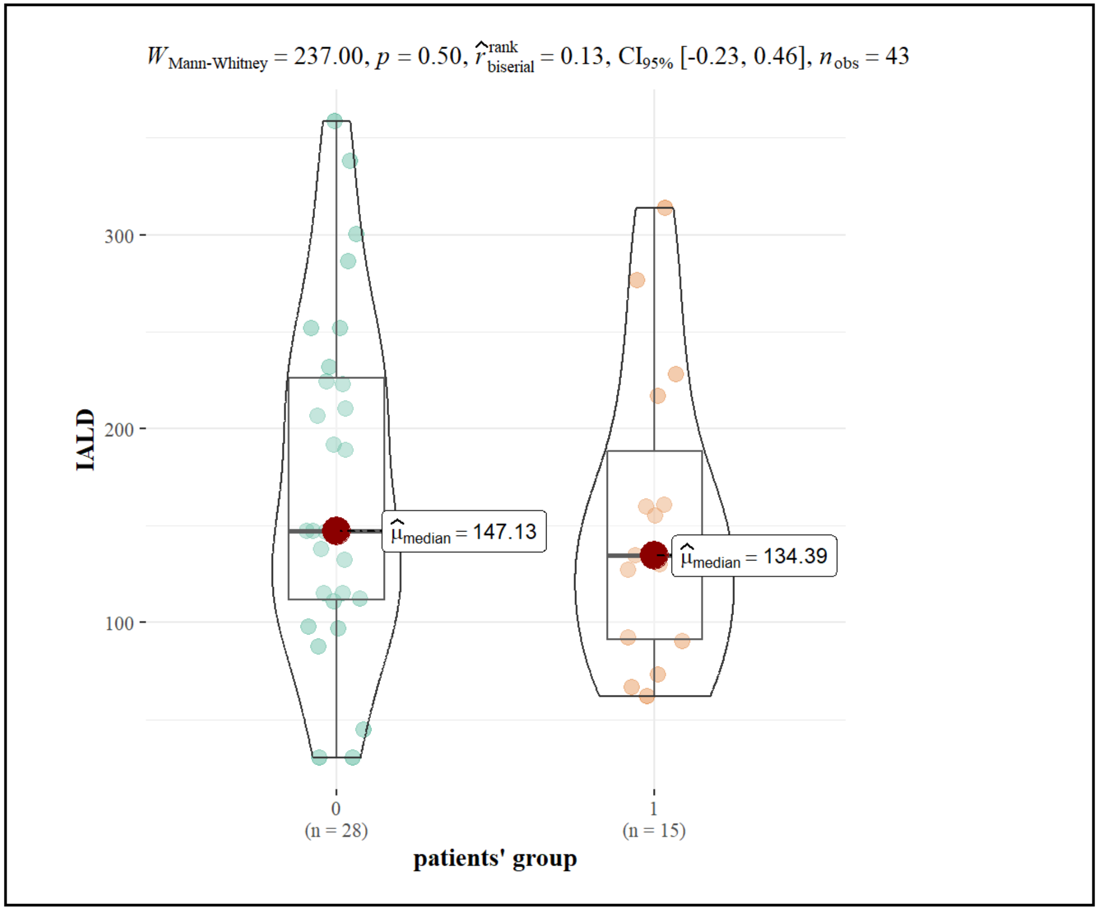
$W_{\text{Mann-Whitney}} = 237.00$, $p = 0.50$, $\hat{r}_{\text{biserial}}^{\text{rank}} = 0.13$, $CI_{95\%}$ [-0.23, 0.46], $n_{\text{obs}} = 43$
IALD
300
200
100
$\hat{\mu}_{\text{median}} = 147.13$
$\hat{\mu}_{\text{median}} = 134.39$
0
(n = 28)
1
(n = 15)
patients' group

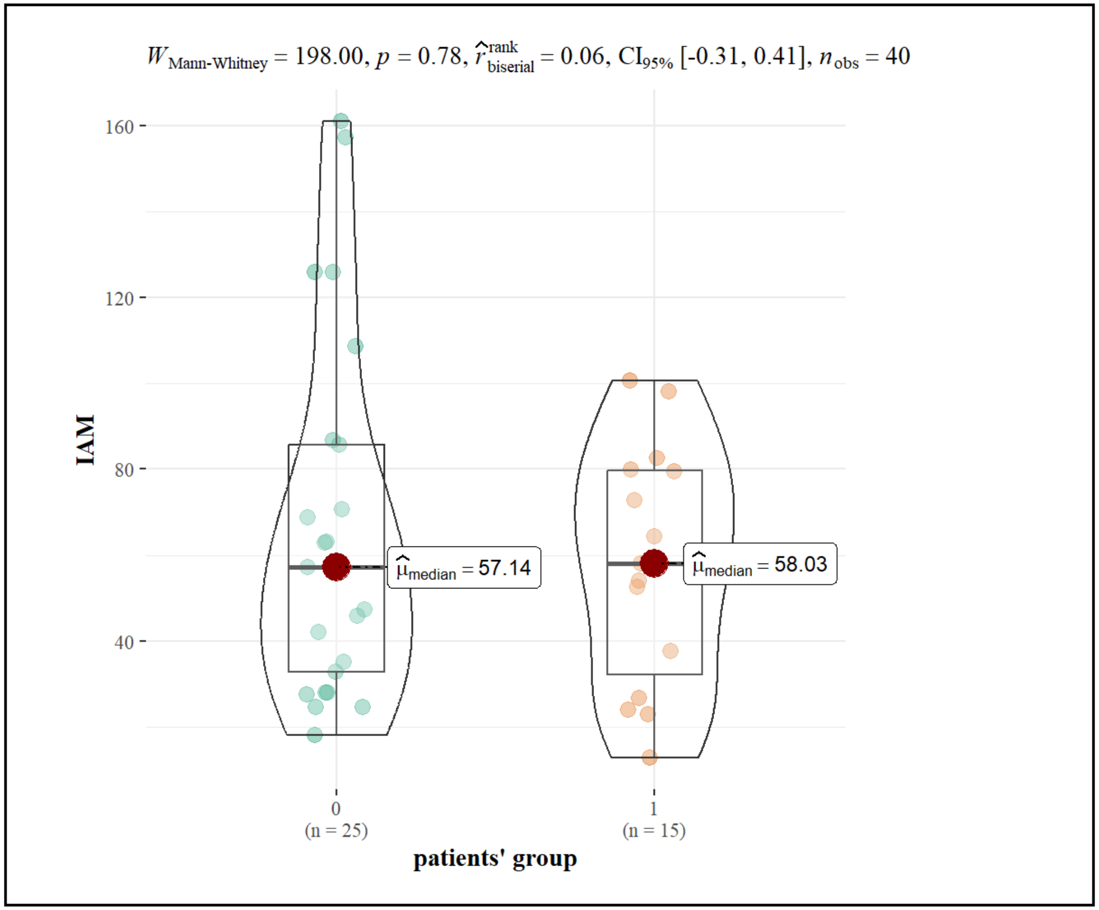

$W_{Mann-Whitney} = 198.00$, $p = 0.78$, $\hat{r}^{rank}_{biserial} = 0.06$, $CI_{95\%}$ [-0.31, 0.41], $n_{obs} = 40$
160
120
80
40
IAM
$\hat{\mu}_{median} = 57.14$
$\hat{\mu}_{median} = 58.03$
0
(n = 25)
1
(n = 15)
patients' group

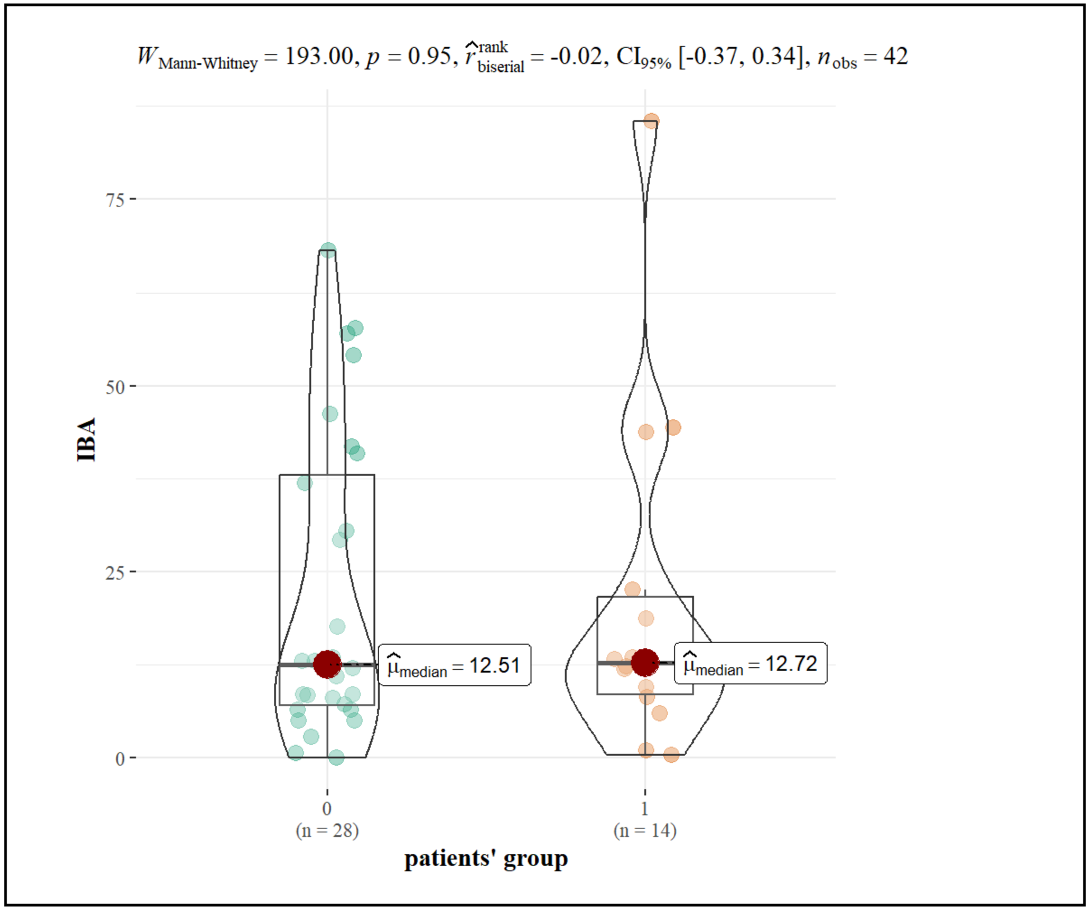

$W_{\text{Mann-Whitney}} = 193.00$, $p = 0.95$, $\hat{r}_{\text{biserial}}^{\text{rank}} = -0.02$, $\text{CI}_{95\%}$ [-0.37, 0.34], $n_{\text{obs}} = 42$
IBA
75
50
25
0
$\hat{\mu}_{\text{median}} = 12.51$
$\hat{\mu}_{\text{median}} = 12.72$
0
(n = 28)
1
(n = 14)
patients' group

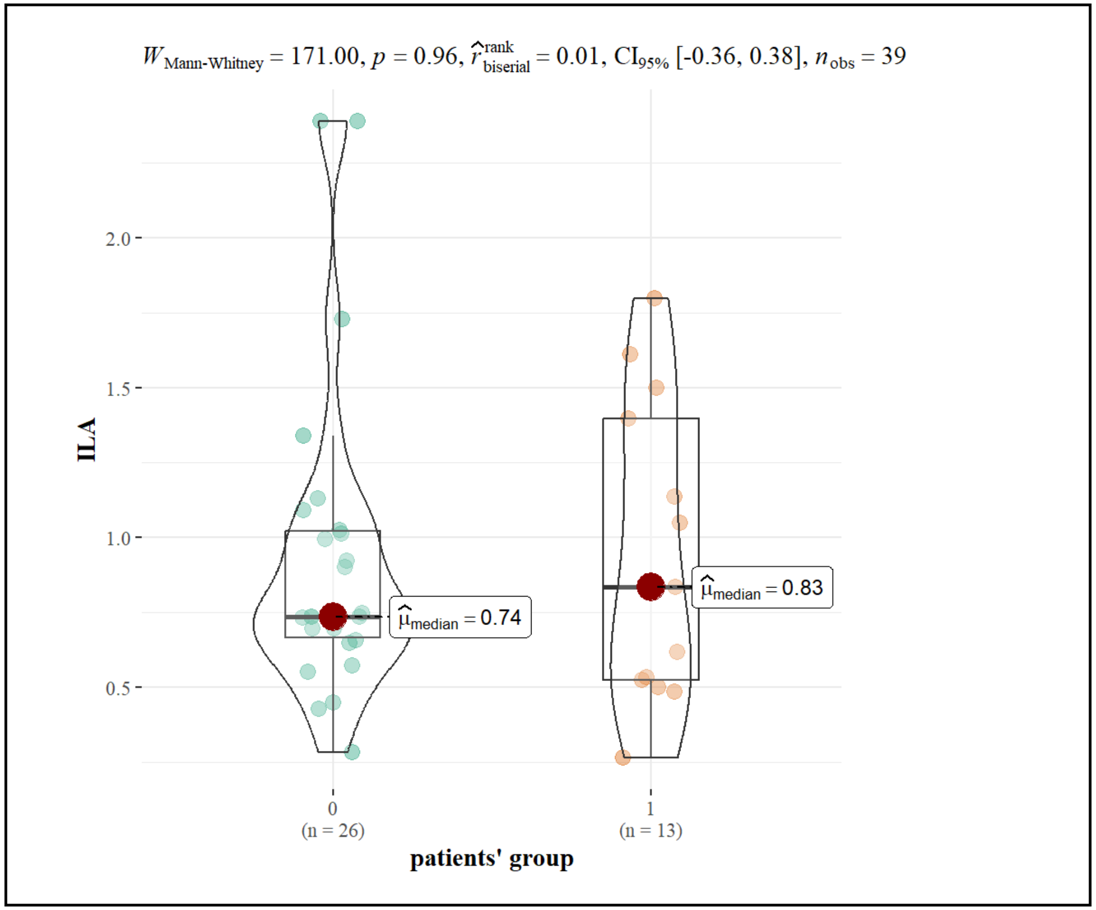

$W_{\text{Mann-Whitney}} = 171.00$, $p = 0.96$, $\hat{r}_{\text{biserial}}^{\text{rank}} = 0.01$, $\text{CI}_{95\%}$ [-0.36, 0.38], $n_{\text{obs}} = 39$
ILA
2.0
1.5
1.0
0.5
$\hat{\mu}_{\text{median}} = 0.74$
$\hat{\mu}_{\text{median}} = 0.83$
0
(n = 26)
1
(n = 13)
patients' group

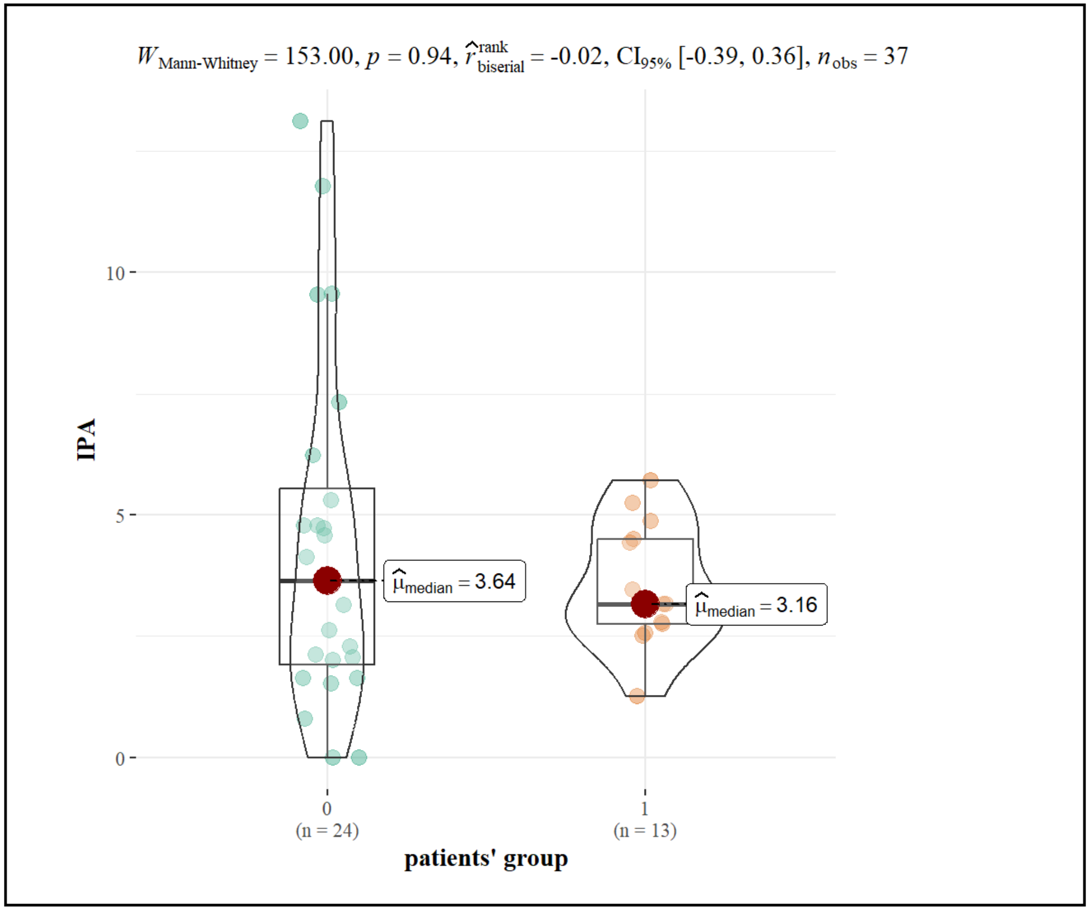

$W_{Mann\text{-}Whitney} = 153.00$, $p = 0.94$, $\hat{r}^{rank}_{biserial} = -0.02$, $CI_{95\%}$ [-0.39, 0.36], $n_{obs} = 37$
IPA
10
5
0
$\hat{\mu}_{median} = 3.64$
$\hat{\mu}_{median} = 3.16$
0
(n = 24)
1
(n = 13)
patients' group

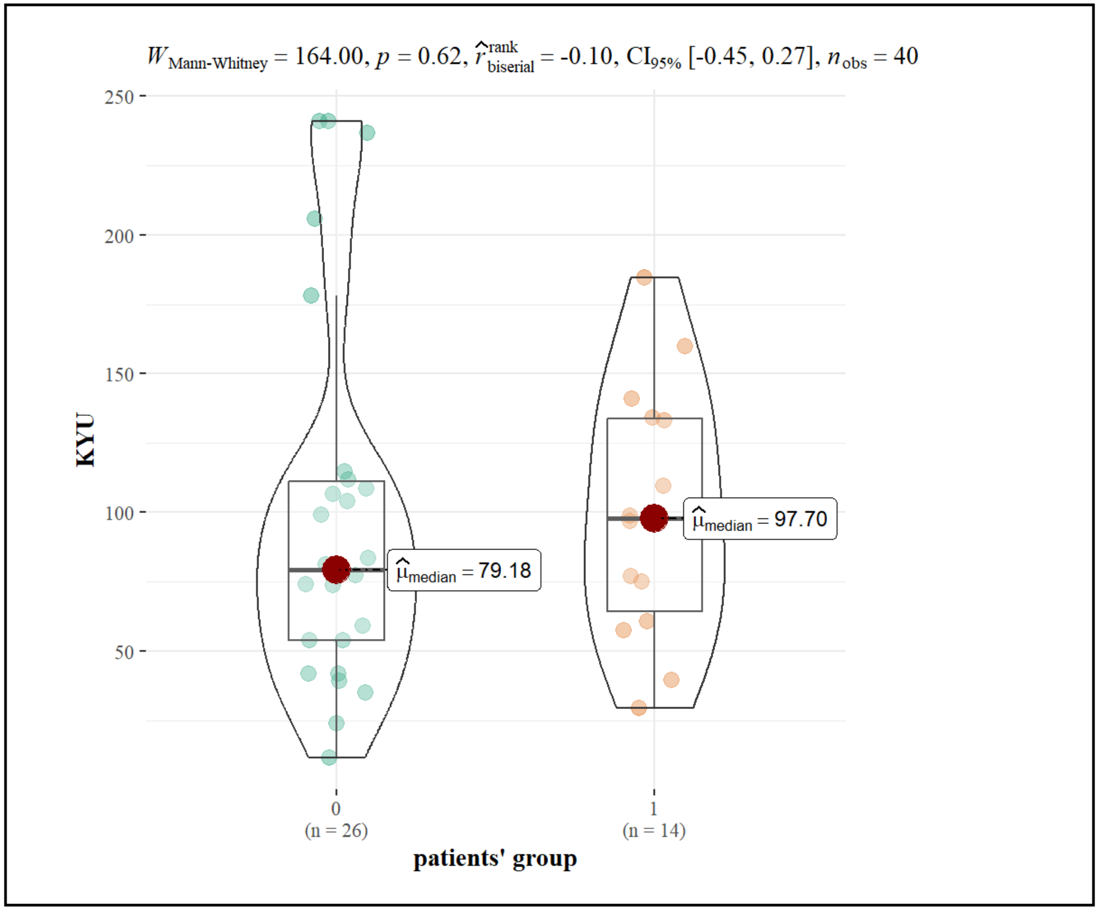

$W_{\text{Mann-Whitney}} = 164.00$, $p = 0.62$, $\hat{r}_{\text{biserial}}^{\text{rank}} = -0.10$, $\text{CI}_{95\%}$ [-0.45, 0.27], $n_{\text{obs}} = 40$
KYU
250
200
150
100
50
$\hat{\mu}_{\text{median}} = 79.18$
$\hat{\mu}_{\text{median}} = 97.70$
0
(n = 26)
1
(n = 14)
patients' group

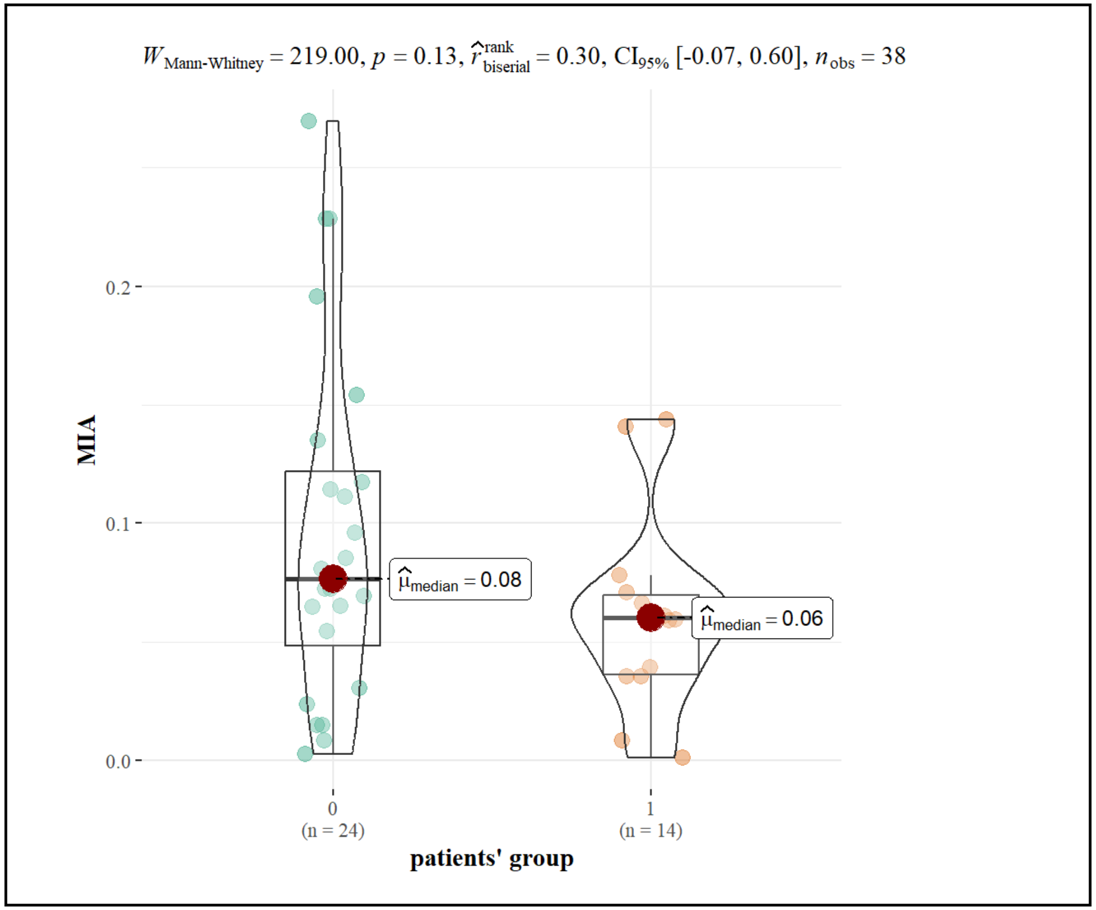

$W_{Mann-Whitney} = 219.00$, $p = 0.13$, $\hat{r}^{rank}_{biserial} = 0.30$, $CI_{95\%}$ [-0.07, 0.60], $n_{obs} = 38$
0.2
0.1
0.0
MIA
$\hat{\mu}_{median} = 0.08$
$\hat{\mu}_{median} = 0.06$
0
(n = 24)
1
(n = 14)
patients' group

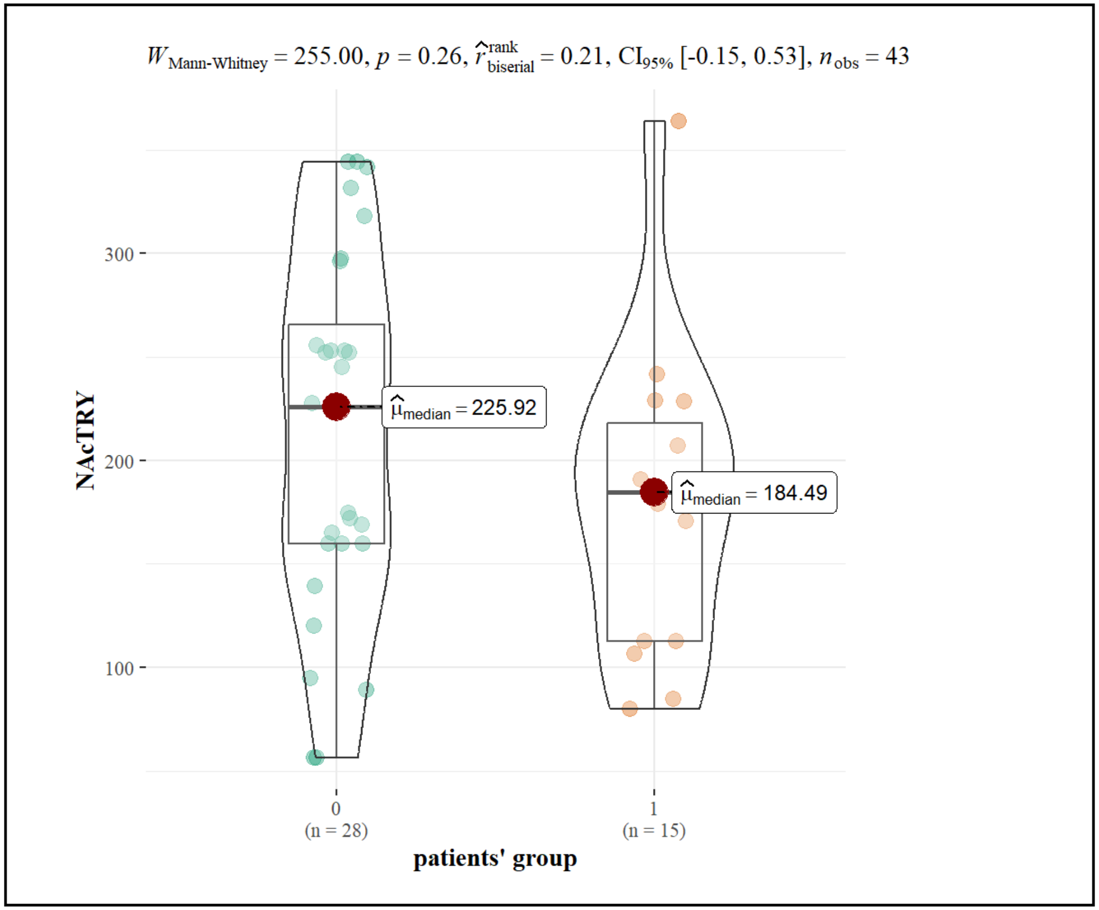

$W_{\text{Mann-Whitney}} = 255.00$, $p = 0.26$, $\hat{r}_{\text{biserial}}^{\text{rank}} = 0.21$, $\text{CI}_{95\%}$ [-0.15, 0.53], $n_{\text{obs}} = 43$
$\hat{\mu}_{\text{median}} = 225.92$
$\hat{\mu}_{\text{median}} = 184.49$
300
200
100
NAcTRY
0
(n = 28)
1
(n = 15)
patients' group

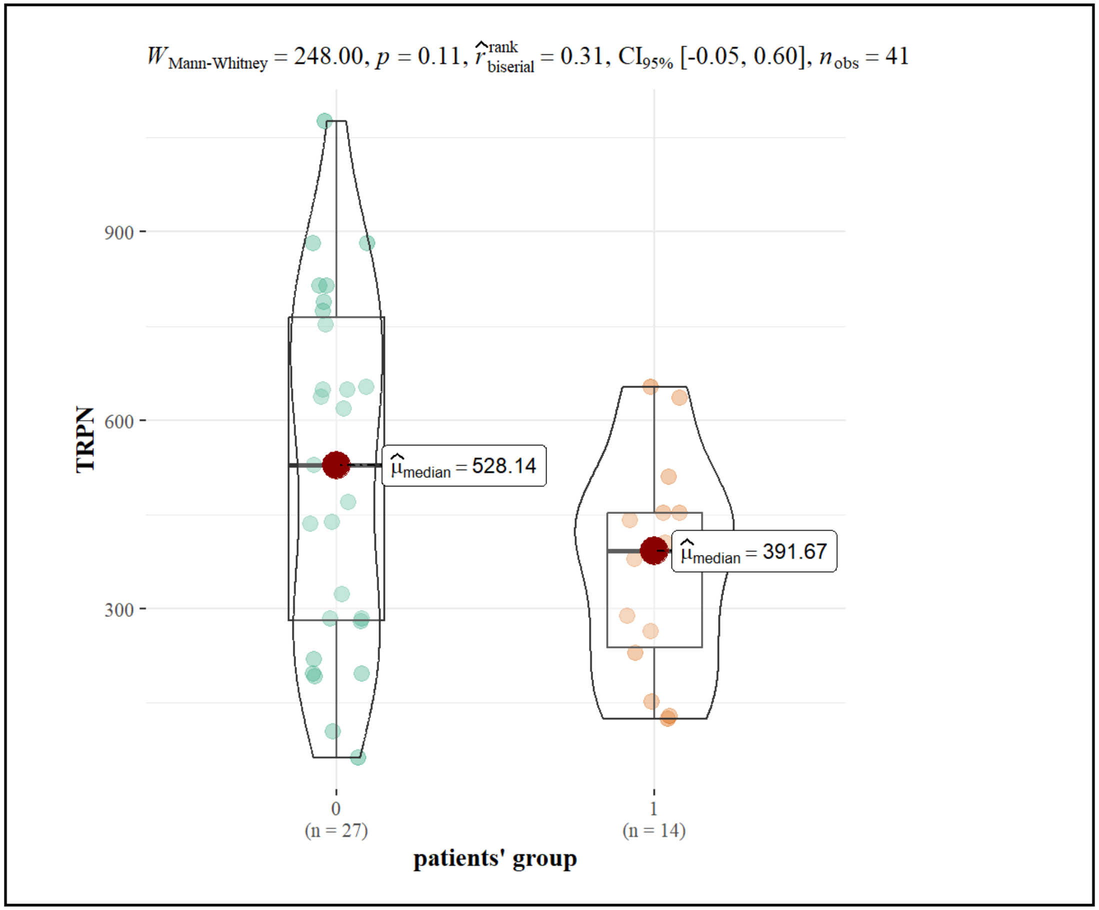
$W_{\text{Mann-Whitney}} = 248.00$, $p = 0.11$, $\hat{r}_{\text{biserial}}^{\text{rank}} = 0.31$, $\text{CI}_{95\%}$ [-0.05, 0.60], $n_{\text{obs}} = 41$
900
600
300
TRPN
$\hat{\mu}_{\text{median}} = 528.14$
$\hat{\mu}_{\text{median}} = 391.67$
0
(n = 27)
1
(n = 14)
patients' group

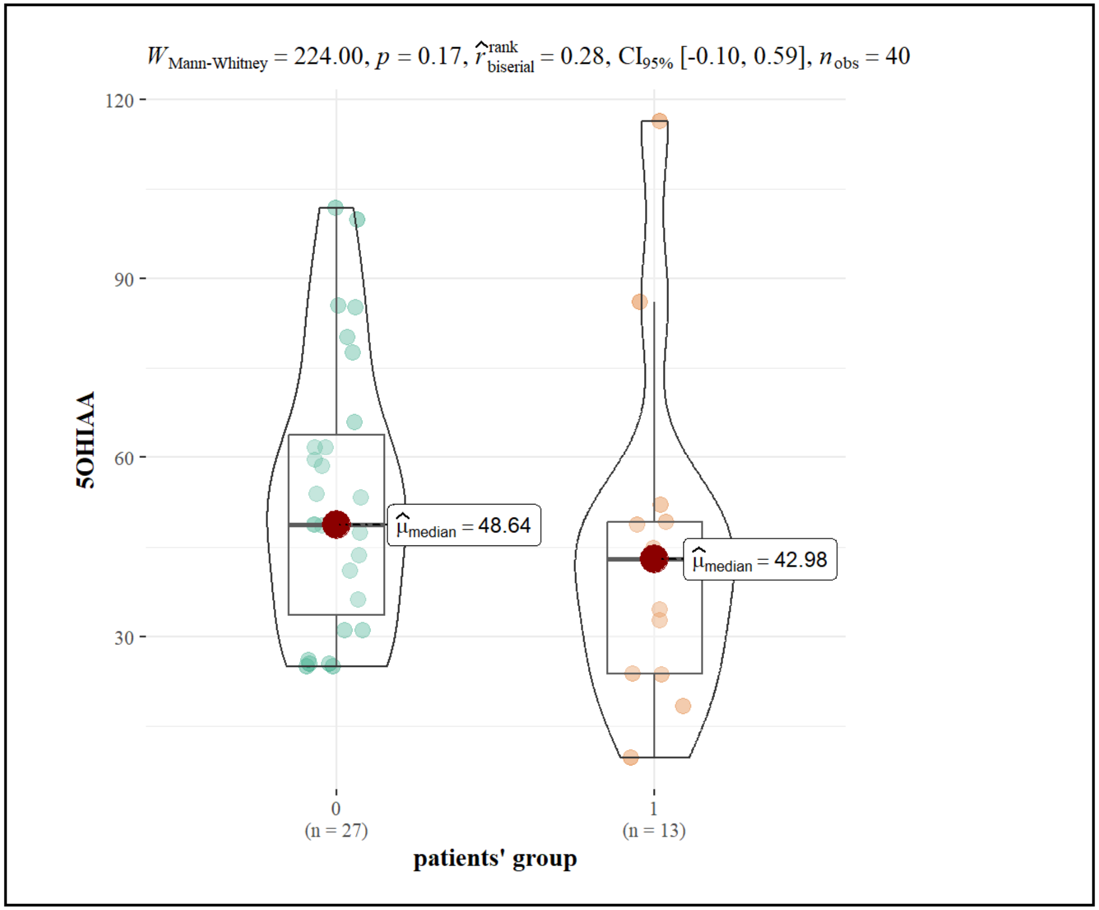

$W_{\text{Mann-Whitney}} = 224.00$, $p = 0.17$, $\hat{r}_{\text{biserial}}^{\text{rank}} = 0.28$, $CI_{95\%}$ [-0.10, 0.59], $n_{\text{obs}} = 40$
120
90
60
30
5OHIAA
$\hat{\mu}_{\text{median}} = 48.64$
$\hat{\mu}_{\text{median}} = 42.98$
0
(n = 27)
1
(n = 13)
patients' group

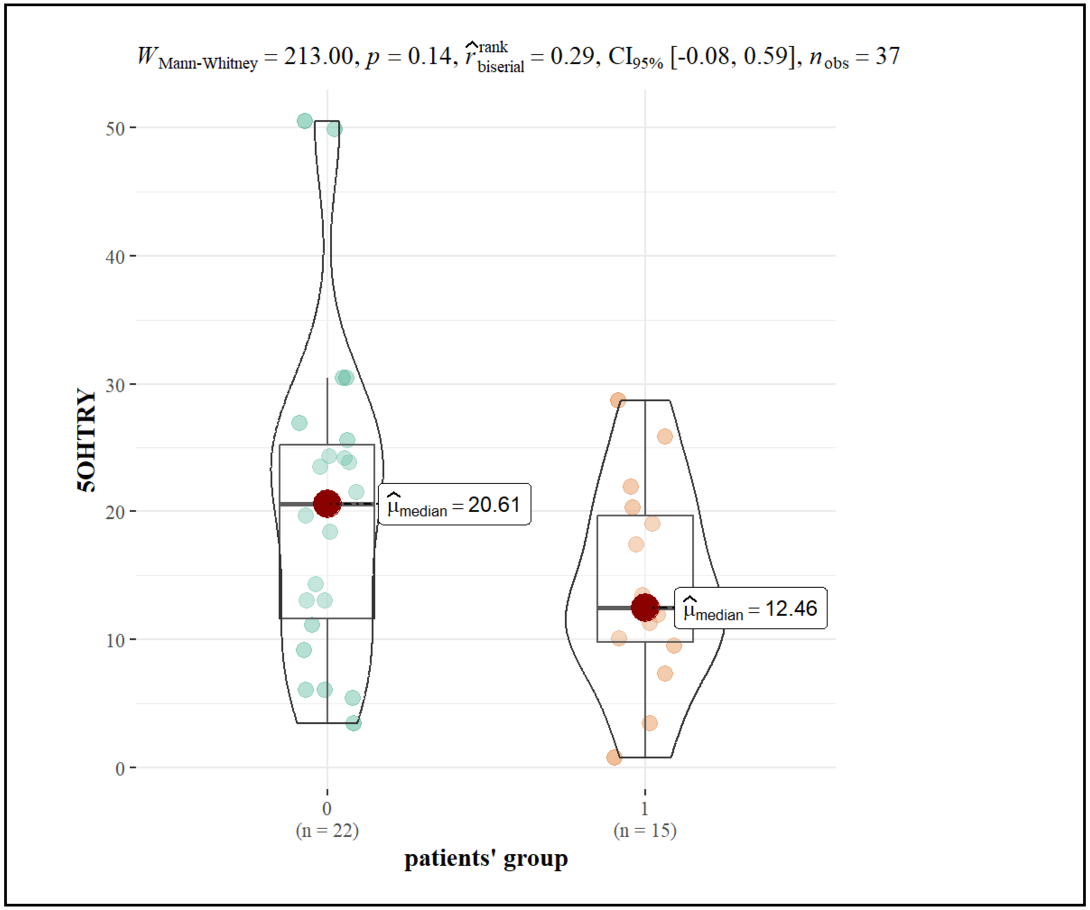

$W_{\text{Mann-Whitney}} = 213.00$, $p = 0.14$, $\hat{r}_{\text{biserial}}^{\text{rank}} = 0.29$, $CI_{95\%}$ [-0.08, 0.59], $n_{\text{obs}} = 37$
5OHTRY
50
40
30
20
10
0
$\hat{\mu}_{\text{median}} = 20.61$
$\hat{\mu}_{\text{median}} = 12.46$
0
(n = 22)
1
(n = 15)
patients' group

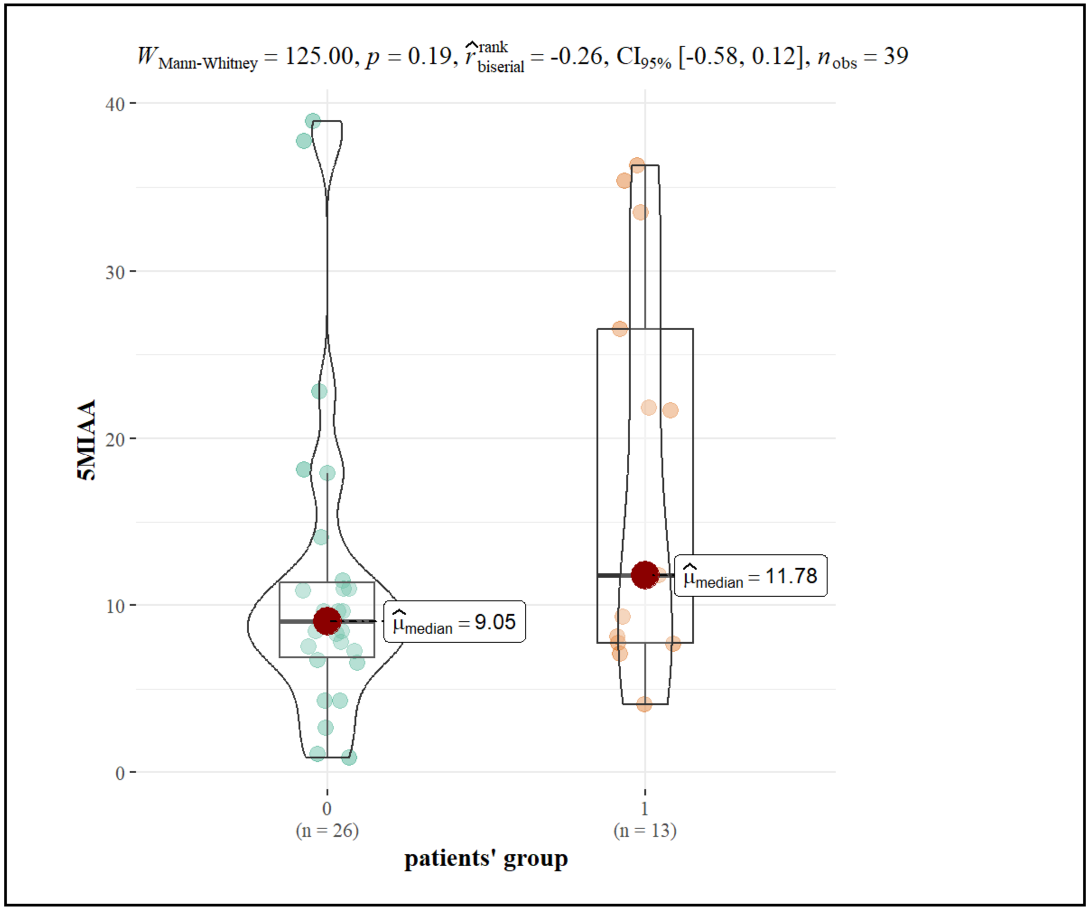
$W_{\text{Mann-Whitney}} = 125.00$, $p = 0.19$, $\hat{r}^{\text{rank}}_{\text{biserial}} = -0.26$, $\text{CI}_{95\%}$ [-0.58, 0.12], $n_{\text{obs}} = 39$
40
30
20
10
0
5MIAA
$\hat{\mu}_{\text{median}} = 9.05$
$\hat{\mu}_{\text{median}} = 11.78$
0
(n = 26)
1
(n = 13)
patients' group

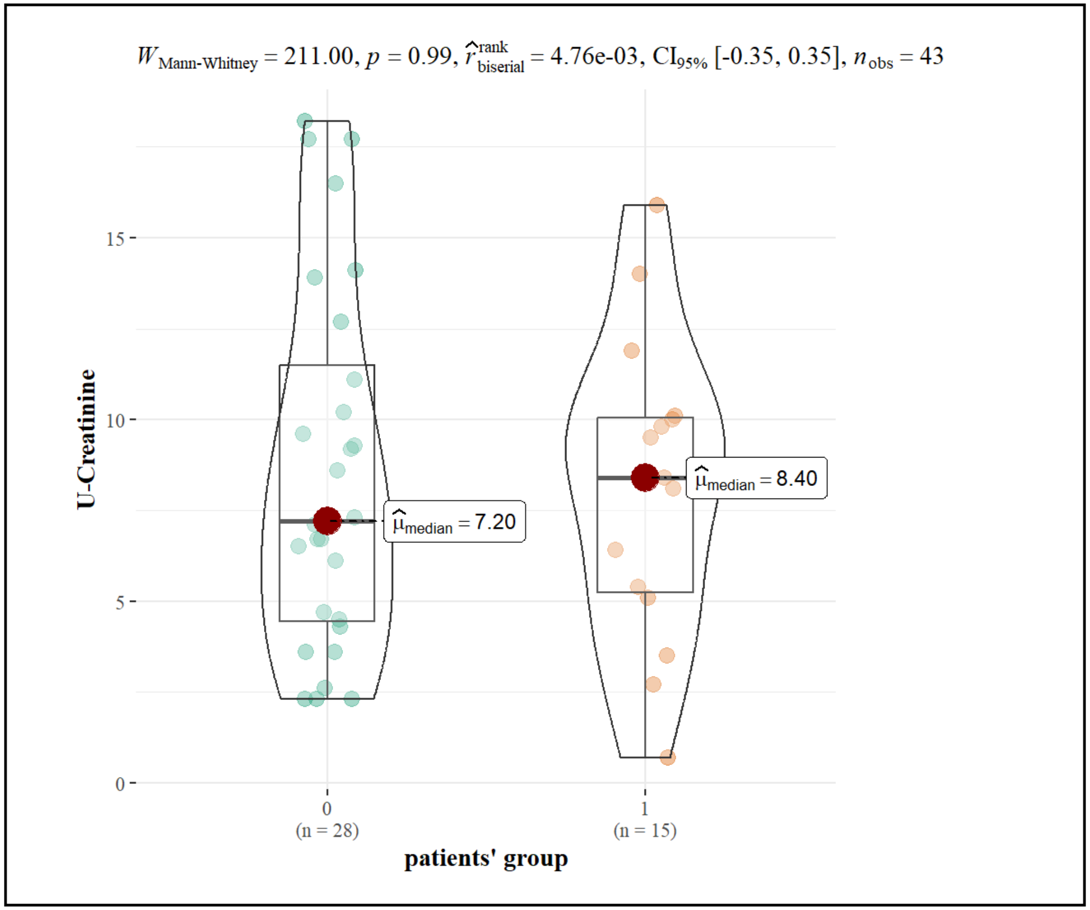

$W_{\text{Mann-Whitney}} = 211.00$, $p = 0.99$, $\widehat{r}_{\text{biserial}}^{\text{rank}} = 4.76\text{e-}03$, $\text{CI}_{95\%}$ [-0.35, 0.35], $n_{\text{obs}} = 43$
U-Creatinine
15
10
5
0
$\widehat{\mu}_{\text{median}} = 7.20$
$\widehat{\mu}_{\text{median}} = 8.40$
0
(n = 28)
1
(n = 15)
patients' group

Table S2: Comparation of the results of tryptophan and their metabolites ratios in the whole group and separately in the moderate and severe group upon CARS score. Differences are presented as p values

Metabolite ratios	Control (N=43)	ASD (N=43)	CARS<36 (N=28)	CARS>36.5 (N=16)	p value			
	1	2	3	4	1-2	1-3	1-4	3-4
KYN_TRP					0.96	0.47	0.27	0.17
Mean (SD)	4.47 (1.76)	4.48 (2.20)	4.20 (1.93)	5.39 (2.81)				
Median (Q1, Q3)	4.21 (3.32, 5.28)	4.67 (2.27, 5.84)	4.60 (2.53, 5.42)	5.53 (3.57, 6.93)				
Min - Max	0.82 - 9.03	1.20 - 9.56	1.20 - 8.34	1.27 - 10.41				
KYN_ATA					0.22	0.54	0.14	0.82
Mean (SD)	4.07 (3.14)	4.72 (2.69)	4.34 (2.46)	6.93 (4.63)				
Median (Q1, Q3)	2.71 (1.98, 5.61)	4.53 (2.83, 5.59)	4.46 (2.83, 5.41)	5.30 (3.41, 8.66)				
Min - Max	0.30 - 12.54	0.57 - 12.81	0.57 - 9.85	2.34 - 17.01				
TRP_IAA					0.34	0.86	0.50	0.29
Mean (SD)	15.32 (10.77)	13.71 (8.80)	13.14 (5.82)	20.60 (19.27)				
Median (Q1, Q3)	11.63 (6.73, 22.28)	11.85 (7.14, 17.96)	11.85 (9.19, 18.12)	13.19 (6.16, 32.42)				
Min - Max	2.12 - 40.36	3.09 - 44.43	3.09 - 22.02	3.44 - 63.91				
IAA_IALD					0.36	0.60	0.70	1.00
Mean (SD)	0.01 (0.01)	0.01 (0.01)	0.01 (0.01)	0.01 (0.00)				
Median (Q1, Q3)	0.01 (0.00, 0.01)	0.01 (0.01, 0.01)	0.01 (0.01, 0.01)	0.01 (0.01, 0.01)				
Min - Max	0.00 - 0.03	0.00 - 0.02	0.00 - 0.03	0.00 - 0.02				
TRP_IAM					0.42	0.86	0.28	0.27
Mean (SD)	0.39 (0.22)	0.45 (0.32)	0.42 (0.33)	0.53 (0.30)				
Median (Q1, Q3)	0.34 (0.23, 0.50)	0.31 (0.20, 0.76)	0.27 (0.17, 0.73)	0.44 (0.29, 0.79)				
Min - Max	0.10 - 1.03	0.02 - 1.08	0.02 - 1.05	0.19 - 1.08				

TRP_ILA					0.24	0.06	0.56	0.81
Mean (SD)	21.16 (13.25)	25.38 (14.16)	26.08 (14.03)	23.81 (14.36)				
Median (Q1, Q3)	17.41 (13.06, 27.07)	22.58 (14.49, 32.35)	25.71 (14.21, 32.35)	19.01 (15.41, 29.69)				
Min - Max	4.32 - 57.27	0.79 - 56.88	0.79 - 56.88	5.88 - 54.20				
TRP_NAcTRP					0.31	0.07	0.04	0.43
Mean (SD)	0.11 (0.07)	0.12 (0.04)	0.12 (0.04)	0.13 (0.07)				
Median (Q1, Q3)	0.09 (0.07, 0.15)	0.12 (0.10, 0.15)	0.13 (0.10, 0.15)	0.11 (0.09, 0.16)				
Min - Max	0.02 - 0.26	0.05 - 0.25	0.05 - 0.22	0.06 - 0.31				
TRP_TRPN					0.46	0.39	0.37	0.03
Mean (SD)	0.05 (0.03)	0.05 (0.02)	0.04 (0.02)	0.05 (0.02)				
Median (Q1, Q3)	0.04 (0.03, 0.07)	0.05 (0.03, 0.06)	0.04 (0.03, 0.05)	0.06 (0.05, 0.06)				
Min - Max	0.01 - 0.13	0.01 - 0.09	0.02 - 0.09	0.01 - 0.09				
TRP_5OHIAA					0.98	0.44	0.92	0.82
Mean (SD)	0.46 (0.27)	0.48 (0.33)	0.39 (0.21)	0.62 (0.48)				
Median (Q1, Q3)	0.37 (0.27, 0.63)	0.33 (0.22, 0.57)	0.33 (0.23, 0.54)	0.41 (0.21, 1.01)				
Min - Max	0.06 - 1.17	0.13 - 1.32	0.13 - 0.98	0.17 - 1.72				
TRP_5OHTRP					0.01	0.68	0.09	0.08
Mean (SD)	0.83 (0.55)	1.28 (0.89)	0.84 (0.54)	1.69 (0.83)				
Median (Q1, Q3)	0.62 (0.44, 1.07)	1.06 (0.59, 1.83)	0.69 (0.40, 1.12)	1.83 (1.06, 2.39)				
Min - Max	0.16 - 2.14	0.26 - 3.03	0.26 - 2.41	0.54 - 2.81				
TRP_5MIAA					0.04	0.04	0.52	0.34
Mean (SD)	1.36 (0.96)	2.25 (1.79)	2.32 (1.76)	1.45 (1.12)				
Median (Q1, Q3)	1.10 (0.56, 2.11)	1.57 (0.83, 3.84)	1.69 (0.85, 4.01)	1.09 (0.69, 1.95)				
Min - Max	0.11 - 3.52	0.27 - 6.42	0.23 - 5.54	0.27 - 3.76				
TRP_IPA					0.41	0.74	0.43	0.67
Mean (SD)	4.20 (2.86)	4.86 (3.38)	4.81 (3.50)	1.41 (1.09)				

Median (Q1, Q3)	3.83 (2.06, 5.91)	3.81 (1.99, 6.89)	3.73 (1.89, 6.86)	1.15 (0.64, 1.82)				
Min - Max	0.09 - 10.29	0.64 - 13.18	0.72 - 13.18	0.27 - 4.05				
TRP_IBA					0.49	0.30	0.63	0.62
Mean (SD)	1.40 (1.22)	1.53 (1.08)	2.00 (1.67)	4.96 (3.28)				
Median (Q1, Q3)	0.98 (0.44, 2.08)	1.40 (0.57, 2.32)	1.78 (0.65, 2.71)	4.41 (3.09, 6.48)				
Min - Max	0.09 - 4.62	0.27 - 4.08	0.29 - 6.17	0.64 - 11.28				
TRY_MIA					0.29	0.50	0.10	0.19
Mean (SD)	258.65 (128.65)	307.21 (219.11)	248.60 (165.80)	404.21 (252.53)				
Median (Q1, Q3)	229.38 (173.32, 344.60)	235.91 (140.82, 456.88)	221.01 (140.82, 329.70)	456.88 (178.25, 604.11)				
Min - Max	50.51 - 518.99	25.69 - 763.72	25.69 - 614.63	49.48 - 723.93				
IAA_KYN					0.81	0.61	0.67	0.67
Mean (SD)	0.02 (0.01)	0.02 (0.02)	0.02 (0.02)	0.02 (0.01)				
Median (Q1, Q3)	0.02 (0.01, 0.03)	0.02 (0.01, 0.03)	0.02 (0.01, 0.03)	0.02 (0.00, 0.02)				
Min - Max	0.00 - 0.06	0.00 - 0.06	0.00 - 0.06	0.00 - 0.05				
KYN_IALD					0.19	0.12	0.44	0.93
Mean (SD)	0.55 (0.44)	0.68 (0.45)	0.74 (0.49)	0.68 (0.51)				
Median (Q1, Q3)	0.38 (0.28, 0.72)	0.60 (0.40, 0.77)	0.61 (0.40, 0.93)	0.60 (0.45, 0.65)				
Min - Max	0.06 - 1.73	0.05 - 1.66	0.05 - 1.66	0.13 - 1.93				

1 –2 ASSOCIATION

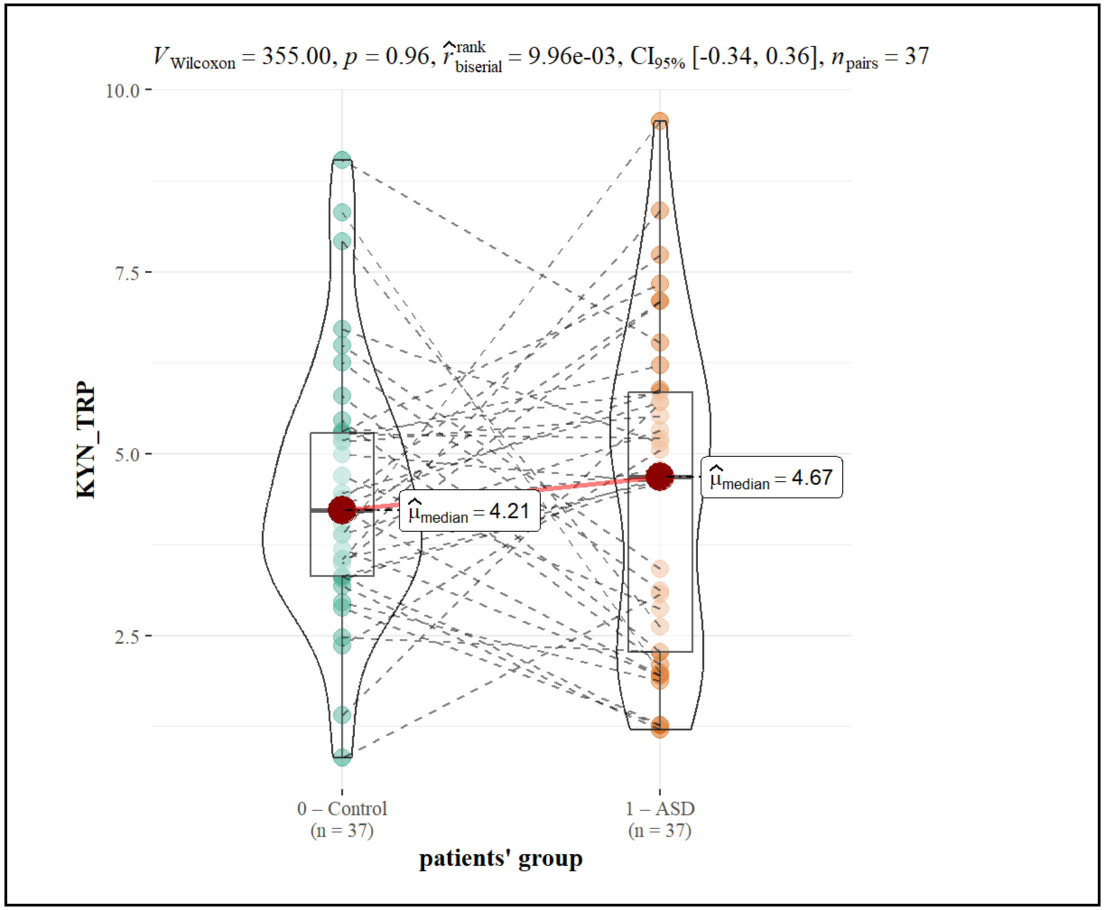
$V_{Wilcoxon} = 355.00$, $p = 0.96$, $\hat{r}^{rank}_{biserial} = 9.96e\text{-}03$, $CI_{95\%}$ [-0.34, 0.36], $n_{pairs} = 37$
10.0
7.5
5.0
2.5
KYN_TRP
$\hat{\mu}_{median} = 4.21$
$\hat{\mu}_{median} = 4.67$
0 – Control
(n = 37)
1 – ASD
(n = 37)
patients' group

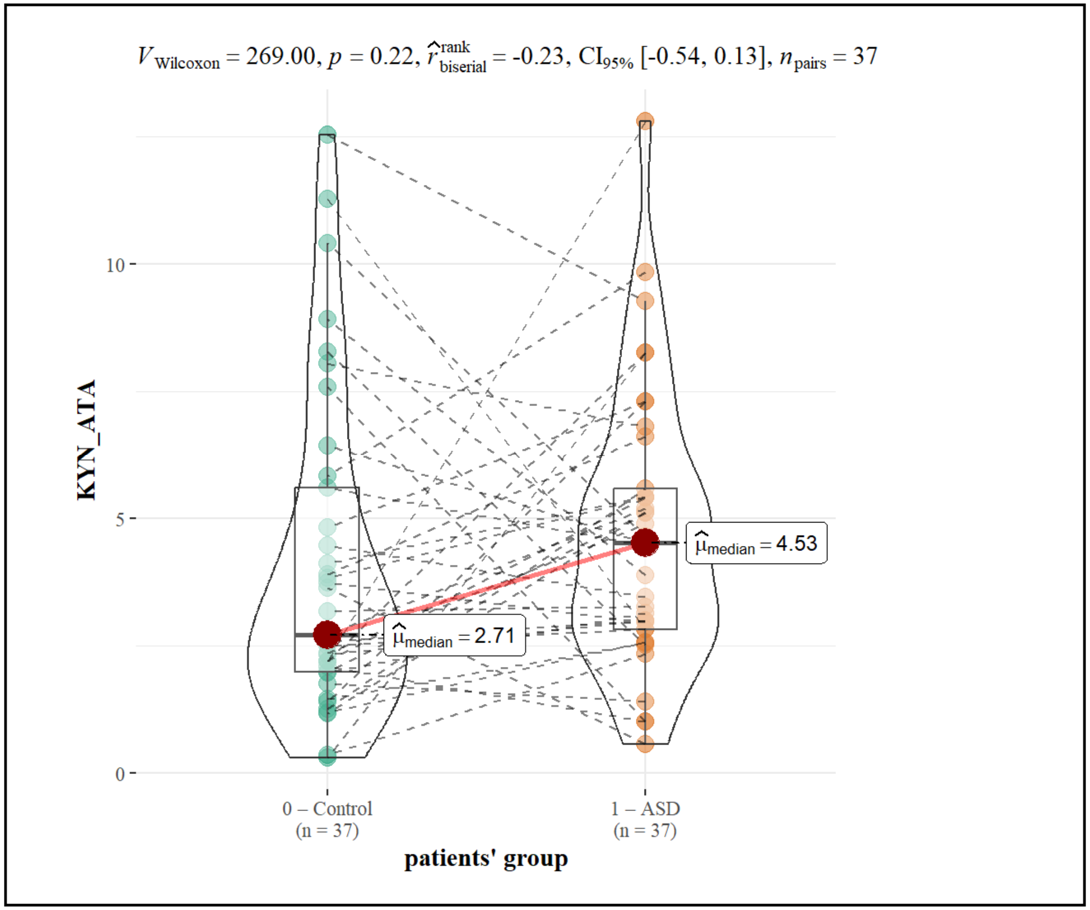

$V_{\text{Wilcoxon}} = 269.00$, $p = 0.22$, $\hat{r}_{\text{biserial}}^{\text{rank}} = -0.23$, $\text{CI}_{95\%}$ [-0.54, 0.13], $n_{\text{pairs}} = 37$
KYN_ATA
10
5
0
$\hat{\mu}_{\text{median}} = 4.53$
$\hat{\mu}_{\text{median}} = 2.71$
0 – Control
(n = 37)
1 – ASD
(n = 37)
patients' group

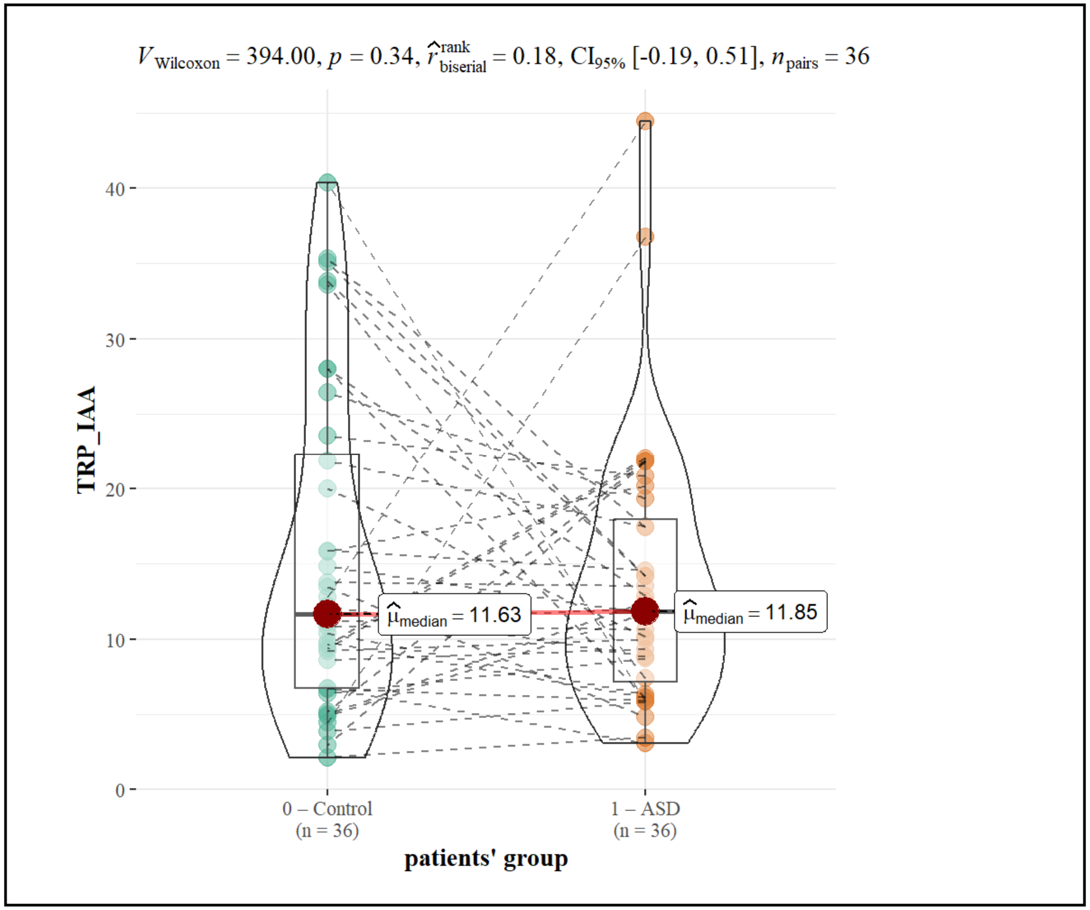

$V_{Wilcoxon}$ = 394.00, p = 0.34, $\hat{r}^{rank}_{biserial}$ = 0.18, $CI_{95\%}$ [-0.19, 0.51], n_{pairs} = 36
TRP_IAA
40
30
20
10
0
$\hat{\mu}_{median}$ = 11.63
$\hat{\mu}_{median}$ = 11.85
0 – Control
(n = 36)
1 – ASD
(n = 36)
patients' group

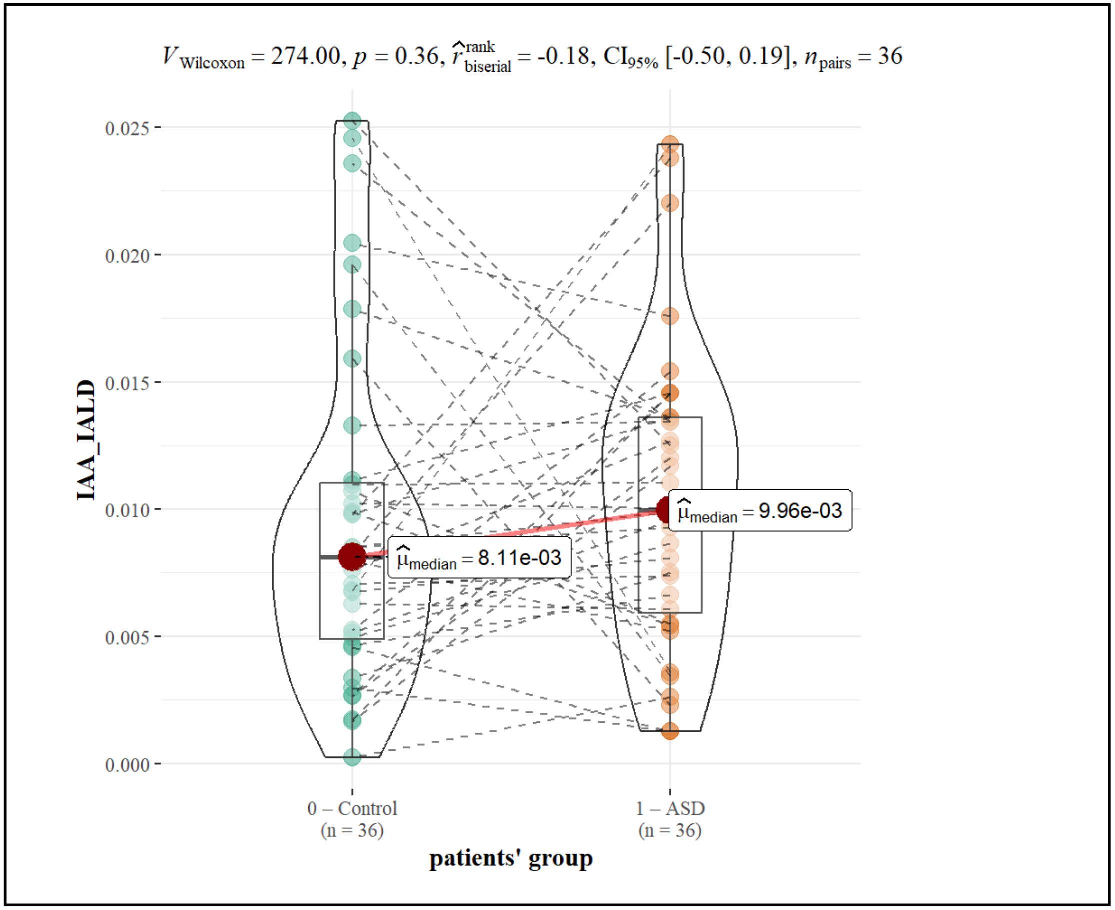

$V_{Wilcoxon} = 274.00$, $p = 0.36$, $\hat{r}^{rank}_{biserial} = -0.18$, $CI_{95\%}$ [-0.50, 0.19], $n_{pairs} = 36$
IAA_IALD
0.025
0.020
0.015
0.010
0.005
0.000
$\hat{\mu}_{median} = 8.11e-03$
$\hat{\mu}_{median} = 9.96e-03$
0 – Control
(n = 36)
1 – ASD
(n = 36)
patients' group

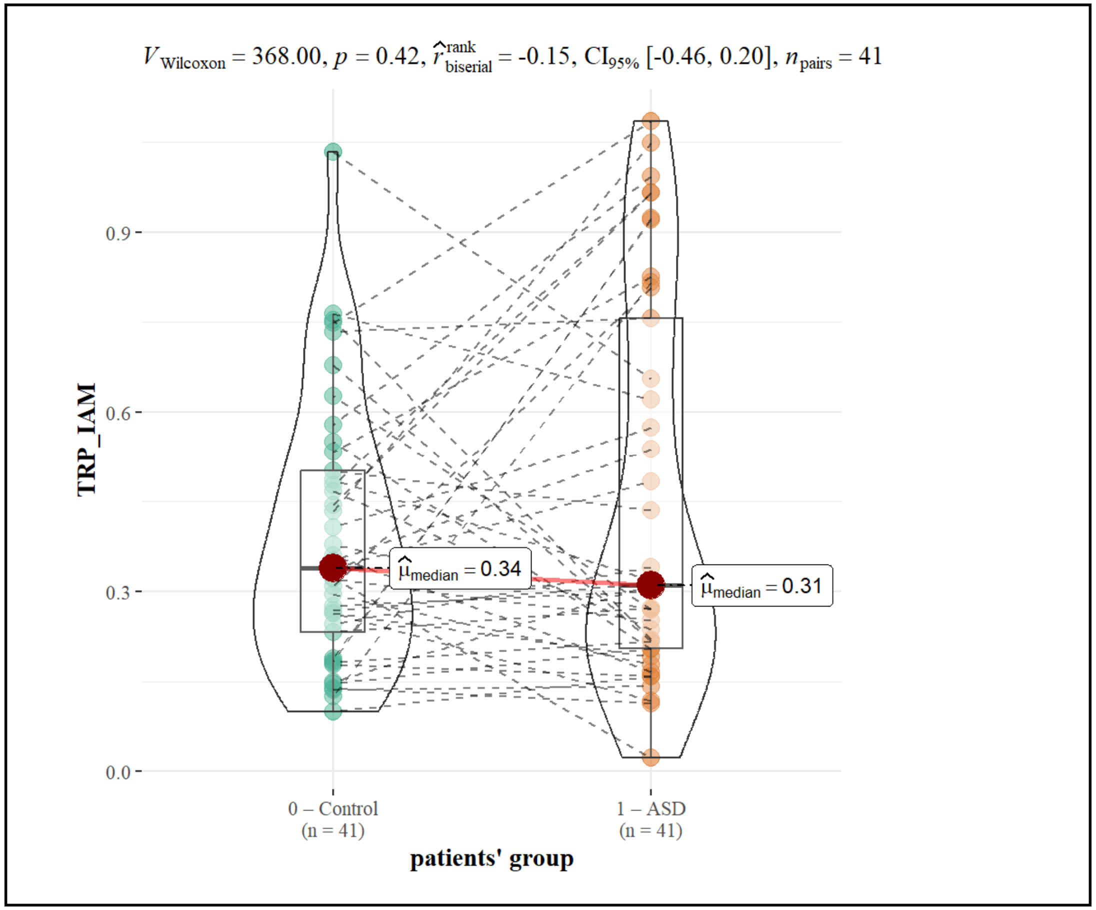

V Wilcoxon = 368.00, p = 0.42, r̂ rank biserial = -0.15, CI95% [-0.46, 0.20], n pairs = 41
TRP_IAM
0.9
0.6
0.3
0.0
μ̂ median = 0.34
μ̂ median = 0.31
0 – Control
(n = 41)
1 – ASD
(n = 41)
patients' group

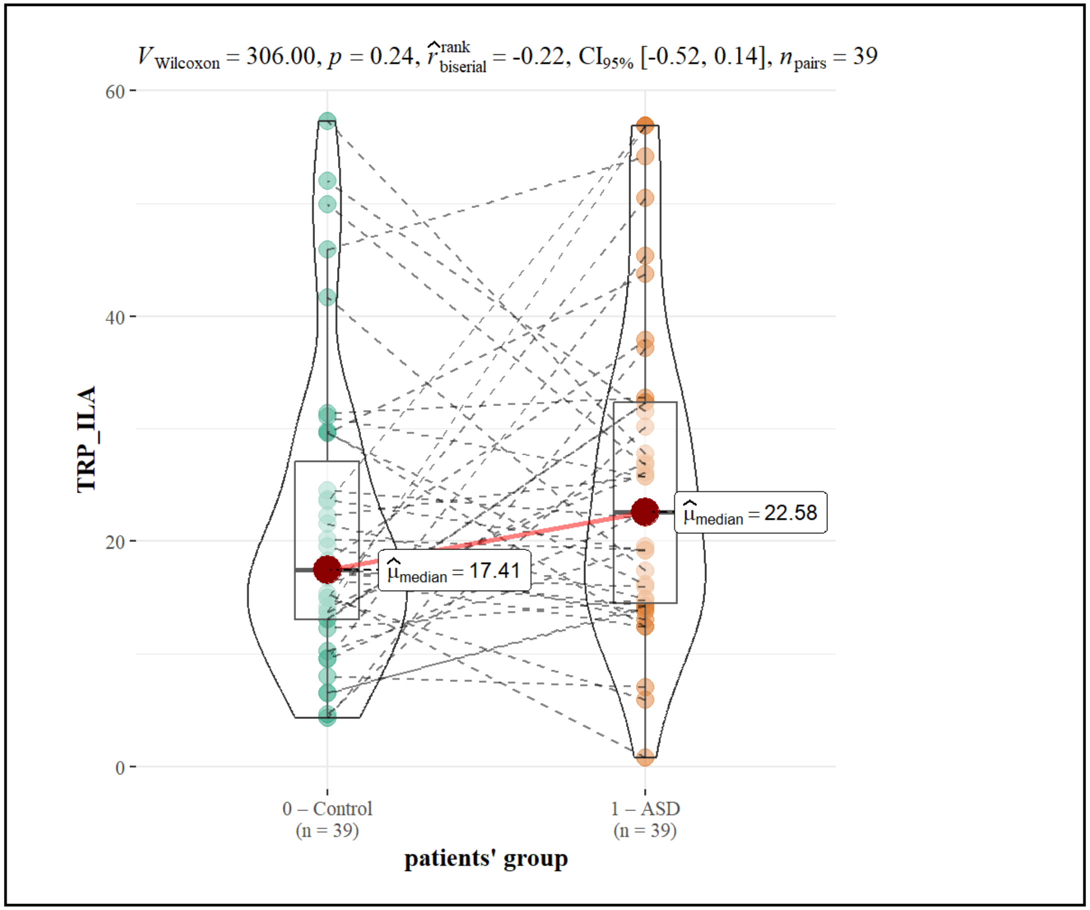
$V_{Wilcoxon} = 306.00$, $p = 0.24$, $\hat{r}^{rank}_{biserial} = -0.22$, $CI_{95\%}$ [-0.52, 0.14], $n_{pairs} = 39$
60
40
20
0
TRP_ILA
$\hat{\mu}_{median} = 17.41$
$\hat{\mu}_{median} = 22.58$
0 – Control
(n = 39)
1 – ASD
(n = 39)
patients' group

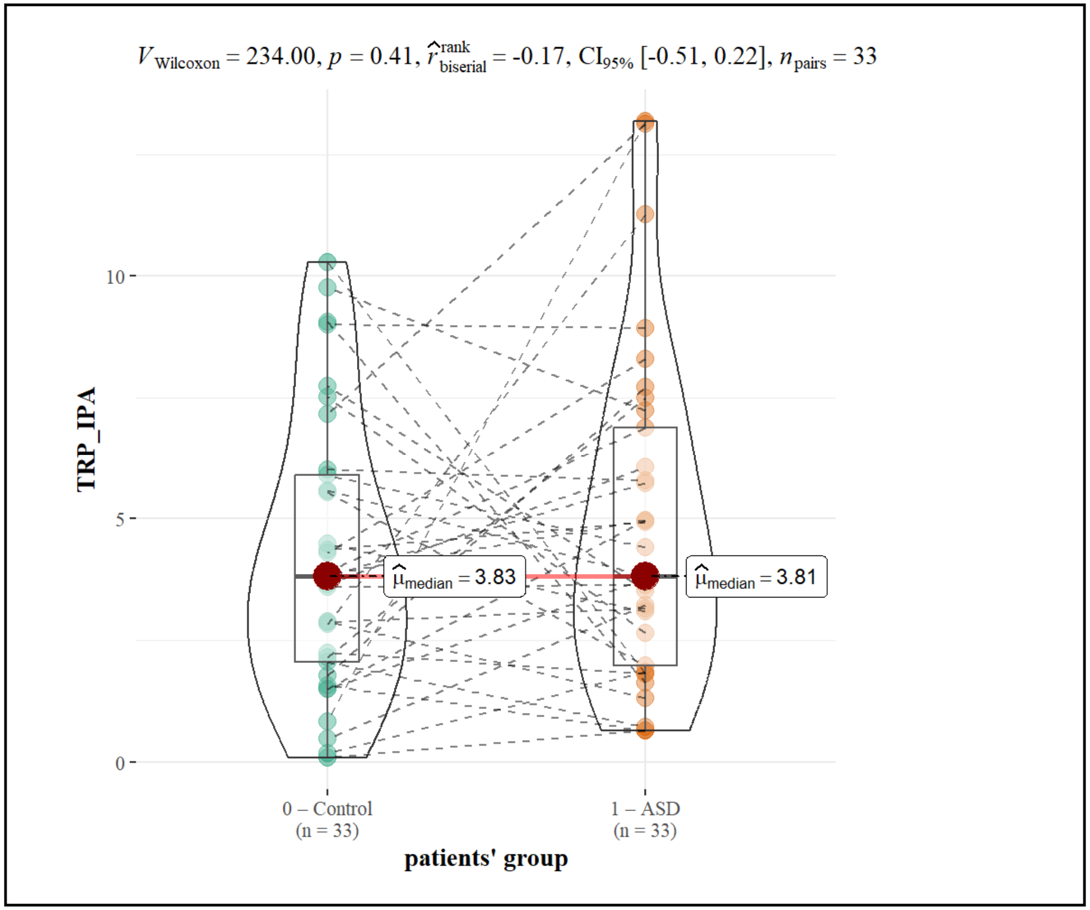

$V_{\text{Wilcoxon}} = 234.00$, $p = 0.41$, $\hat{r}_{\text{biserial}}^{\text{rank}} = -0.17$, $CI_{95\%}$ [-0.51, 0.22], $n_{\text{pairs}} = 33$
TRP_IPA
10
5
0
$\hat{\mu}_{\text{median}} = 3.83$
$\hat{\mu}_{\text{median}} = 3.81$
0 – Control
(n = 33)
1 – ASD
(n = 33)
patients' group

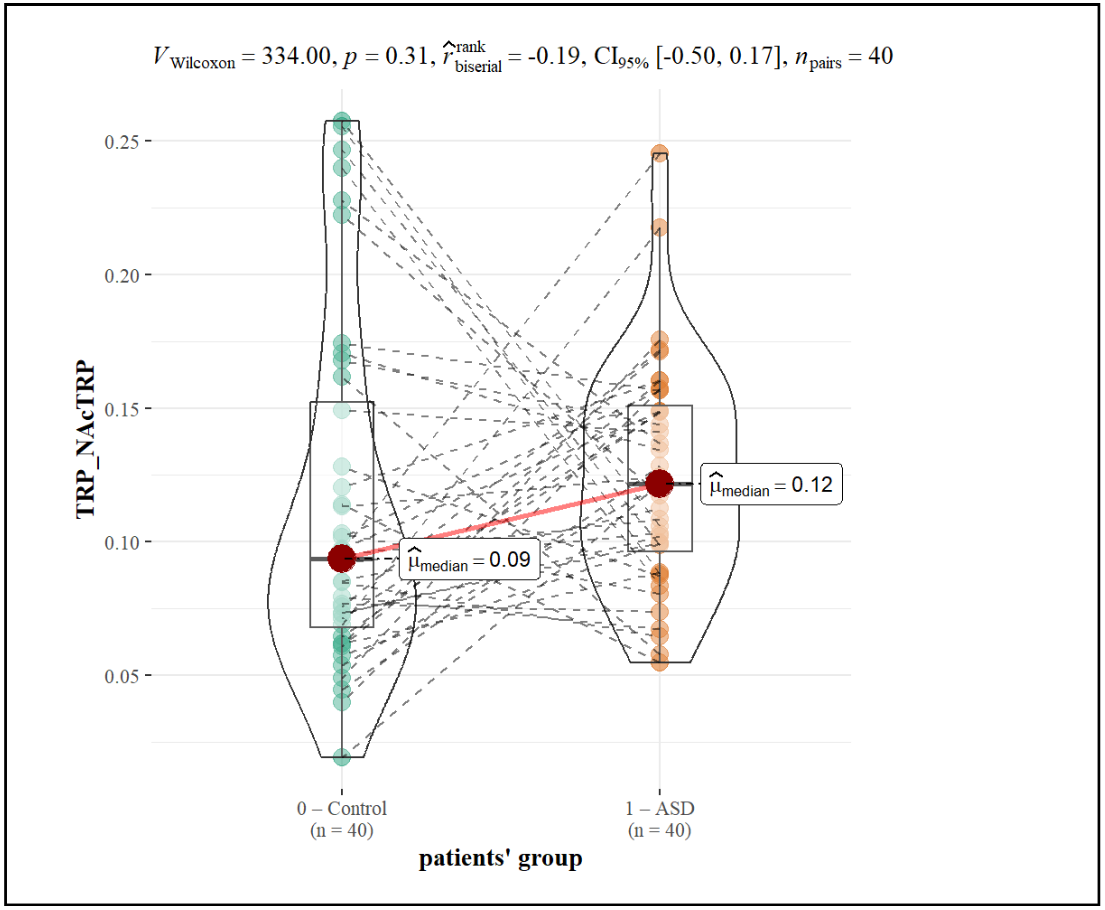

$V_{Wilcoxon}$ = 334.00, p = 0.31, $\hat{r}^{rank}_{biserial}$ = -0.19, $CI_{95\%}$ [-0.50, 0.17], n_{pairs} = 40
TRP_NAcTRP
0.25
0.20
0.15
0.10
0.05
$\hat{\mu}_{median}$ = 0.09
$\hat{\mu}_{median}$ = 0.12
0 – Control
(n = 40)
1 – ASD
(n = 40)
patients' group

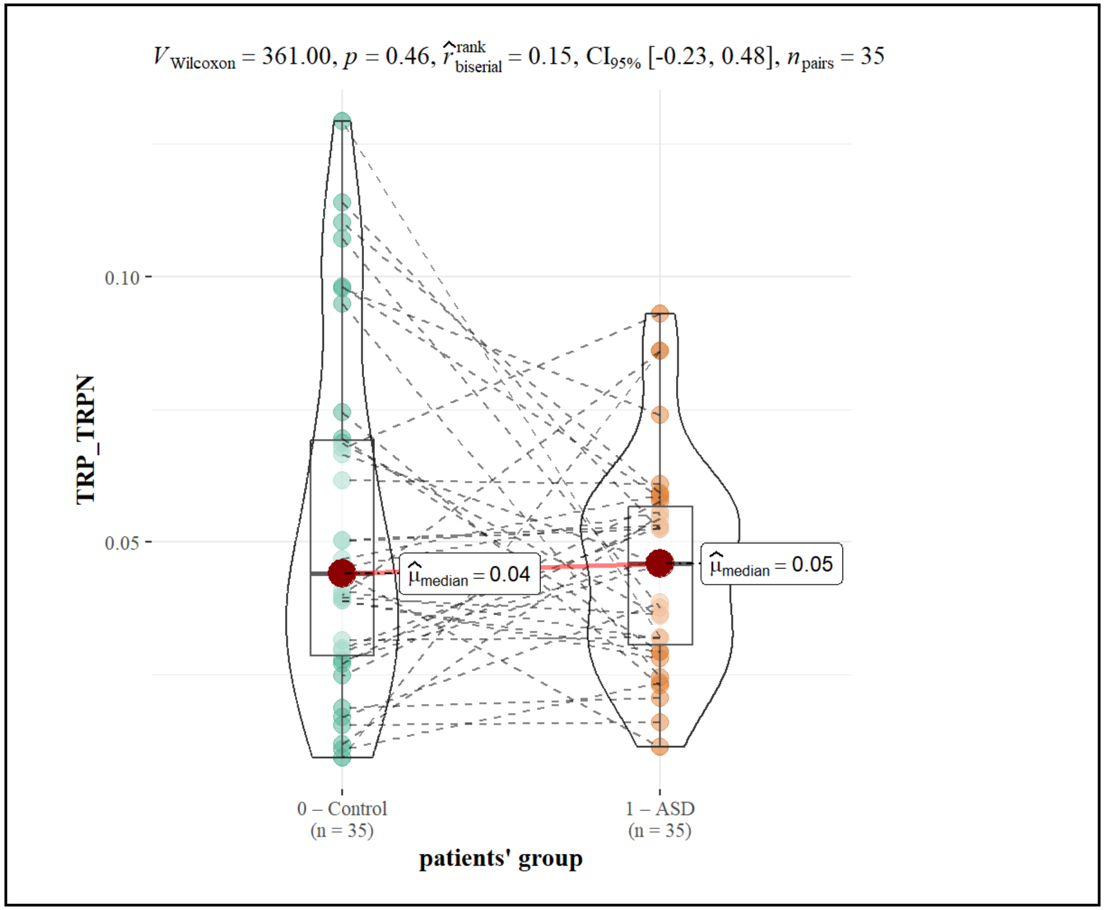

$V_{\text{Wilcoxon}} = 361.00$, $p = 0.46$, $\hat{r}^{\text{rank}}_{\text{biserial}} = 0.15$, $\text{CI}_{95\%}$ [-0.23, 0.48], $n_{\text{pairs}} = 35$
TRP_TRPN
0.10
0.05
$\hat{\mu}_{\text{median}} = 0.04$
$\hat{\mu}_{\text{median}} = 0.05$
0 – Control
(n = 35)
1 – ASD
(n = 35)
patients' group

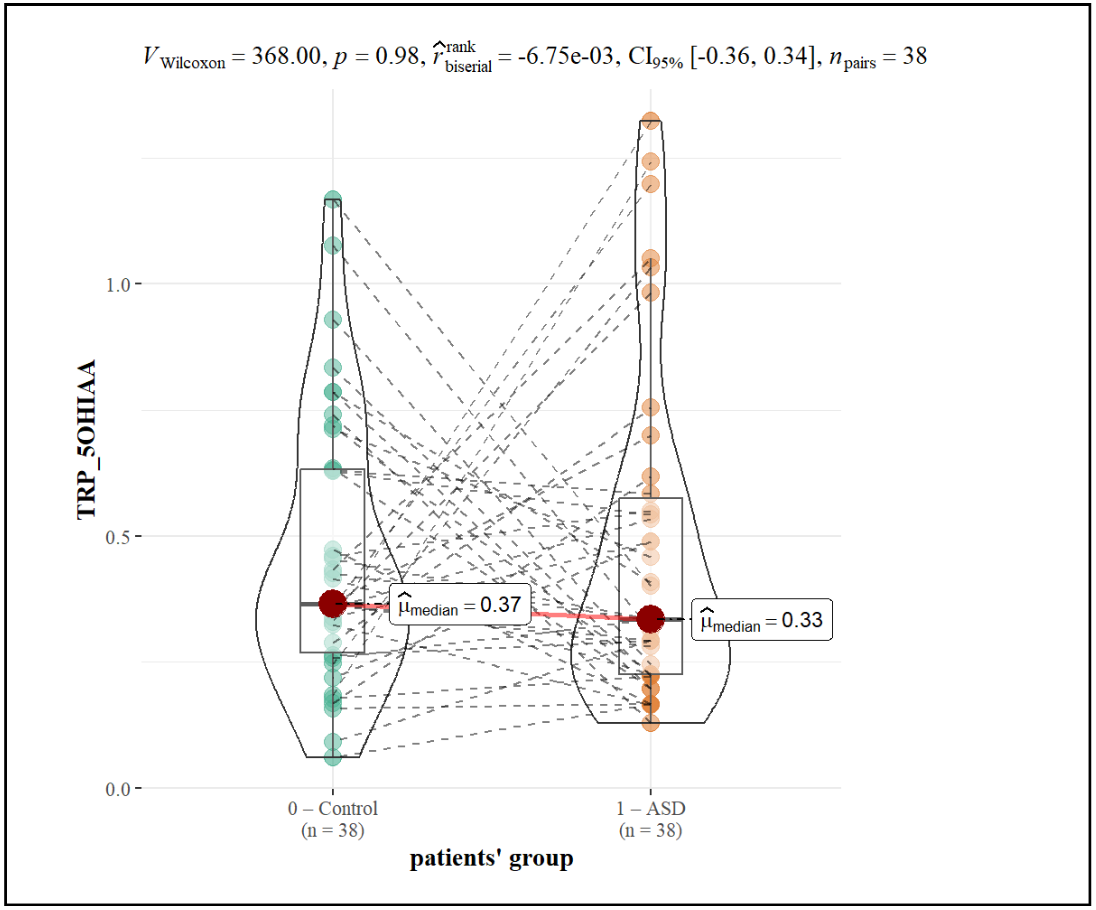

V Wilcoxon = 368.00, p = 0.98, r̂ rank biserial = -6.75e-03, CI95% [-0.36, 0.34], n pairs = 38
TRP_5OHIAA
1.0
0.5
0.0
μ̂ median = 0.37
μ̂ median = 0.33
0 – Control
(n = 38)
1 – ASD
(n = 38)
patients' group

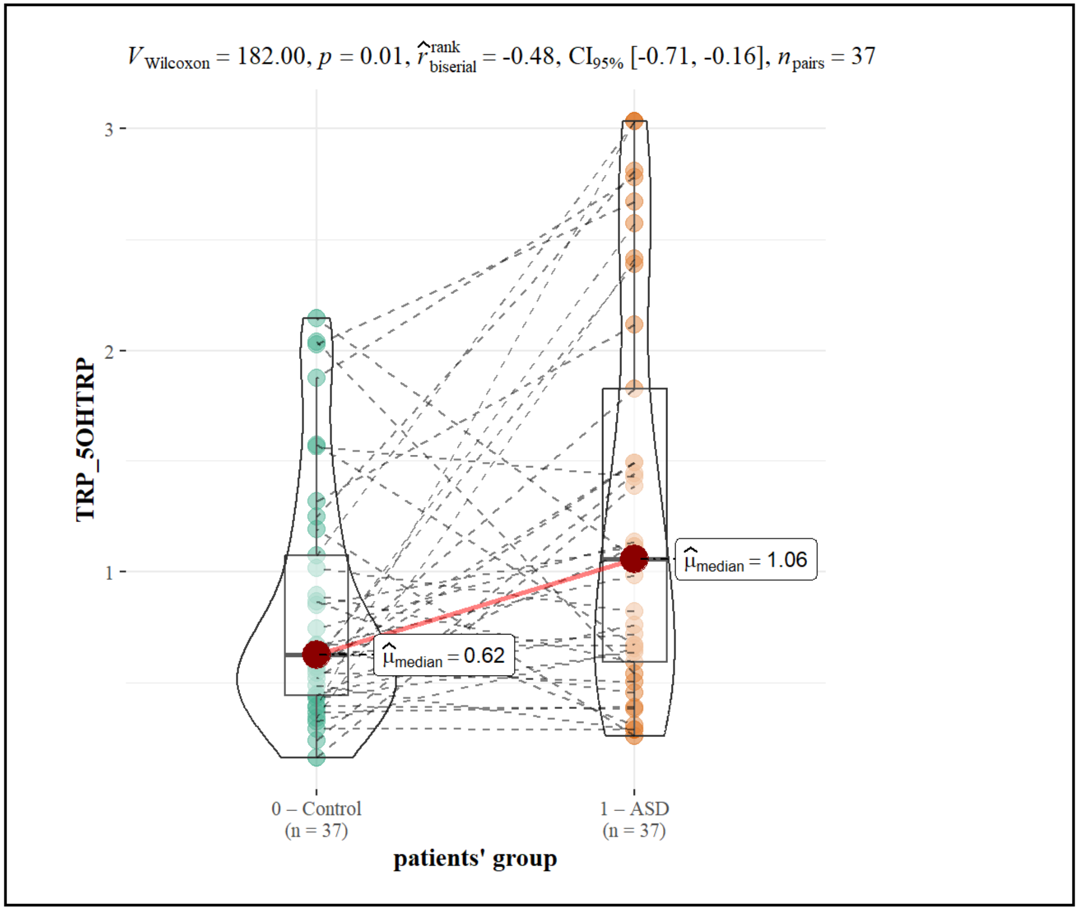
$V_{Wilcoxon}$ = 182.00, p = 0.01, $\hat{r}^{rank}_{biserial}$ = -0.48, $CI_{95\%}$ [-0.71, -0.16], n_{pairs} = 37
TRP_5OHTRP
3
2
1
$\hat{\mu}_{median}$ = 1.06
$\hat{\mu}_{median}$ = 0.62
0 – Control
(n = 37)
1 – ASD
(n = 37)
patients' group

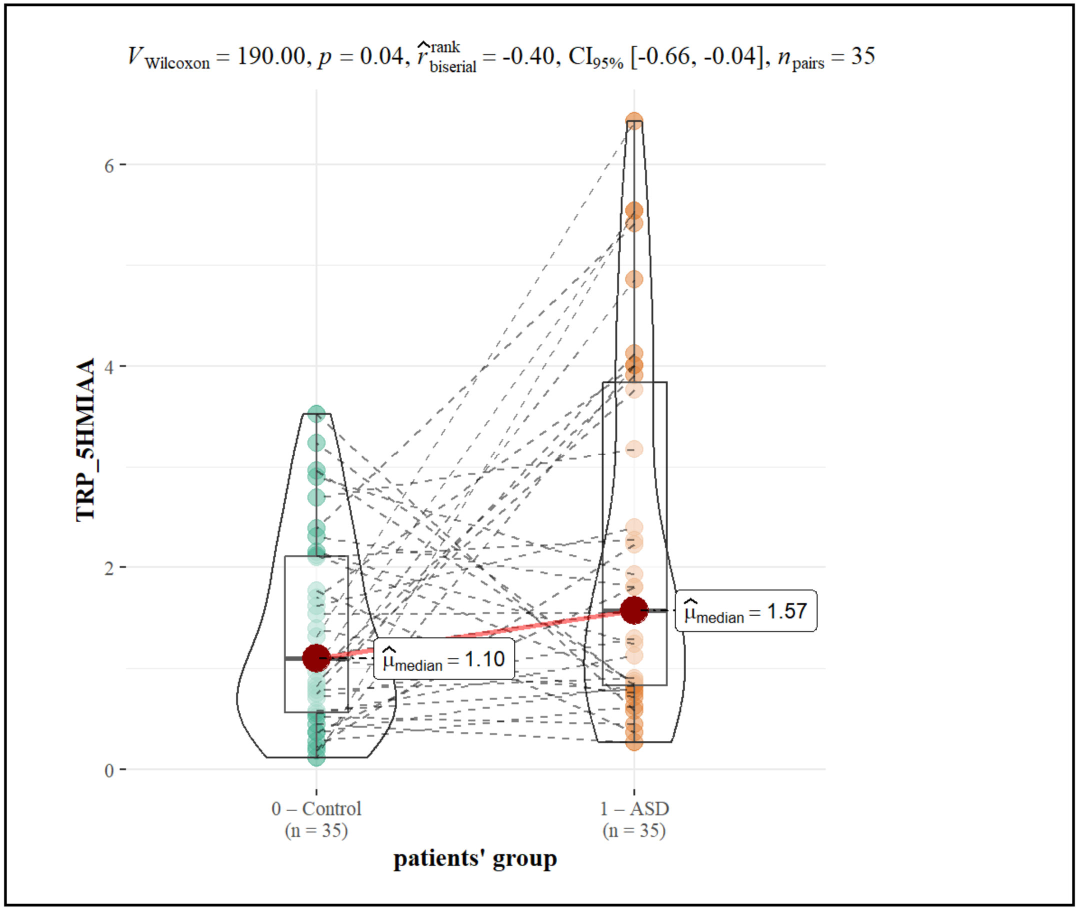
$V_{Wilcoxon}$ = 190.00, p = 0.04, $\hat{r}^{rank}_{biserial}$ = -0.40, $CI_{95\%}$ [-0.66, -0.04], n_{pairs} = 35
TRP_5HMIAA
0
2
4
6
$\hat{\mu}_{median}$ = 1.10
$\hat{\mu}_{median}$ = 1.57
0 – Control
(n = 35)
1 – ASD
(n = 35)
patients' group

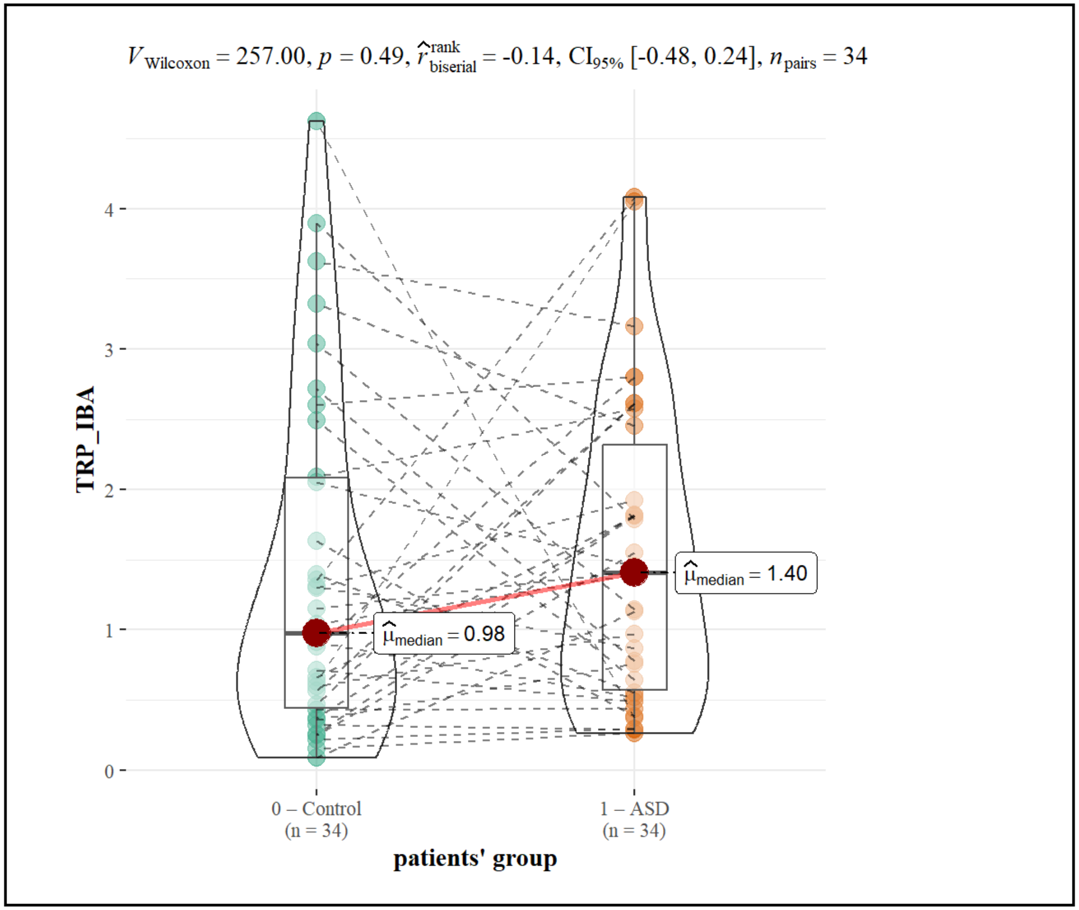
V_{Wilcoxon} = 257.00, p = 0.49, $\hat{r}_{\text{biserial}}^{\text{rank}}$ = -0.14, $CI_{95\%}$ [-0.48, 0.24], n_{pairs} = 34
TRP_IBA
0
1
2
3
4
$\hat{\mu}_{\text{median}}$ = 0.98
$\hat{\mu}_{\text{median}}$ = 1.40
0 – Control
(n = 34)
1 – ASD
(n = 34)
patients' group

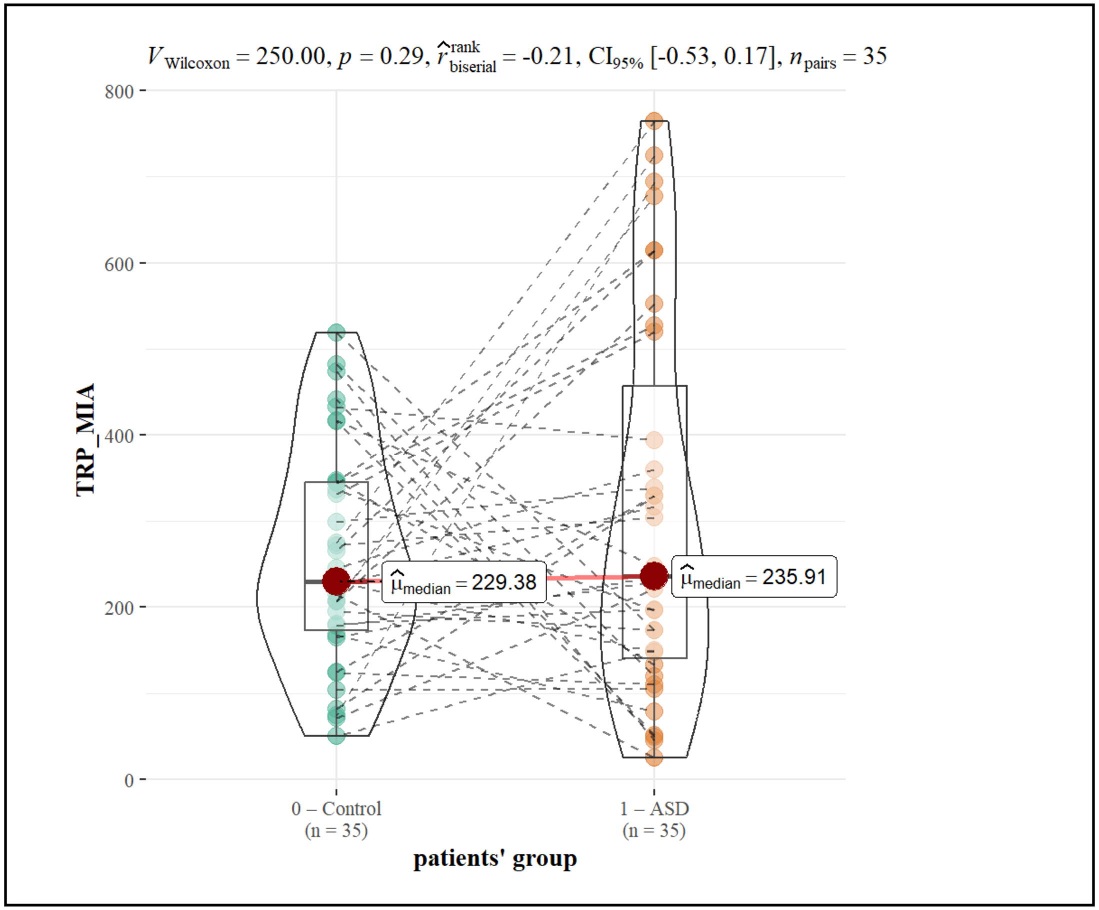

$V_{Wilcoxon} = 250.00$, $p = 0.29$, $\hat{r}_{biserial}^{rank} = -0.21$, $CI_{95\%}$ [-0.53, 0.17], $n_{pairs} = 35$
TRP_MIA
800
600
400
200
0
$\hat{\mu}_{median} = 229.38$
$\hat{\mu}_{median} = 235.91$
0 – Control
(n = 35)
1 – ASD
(n = 35)
patients' group

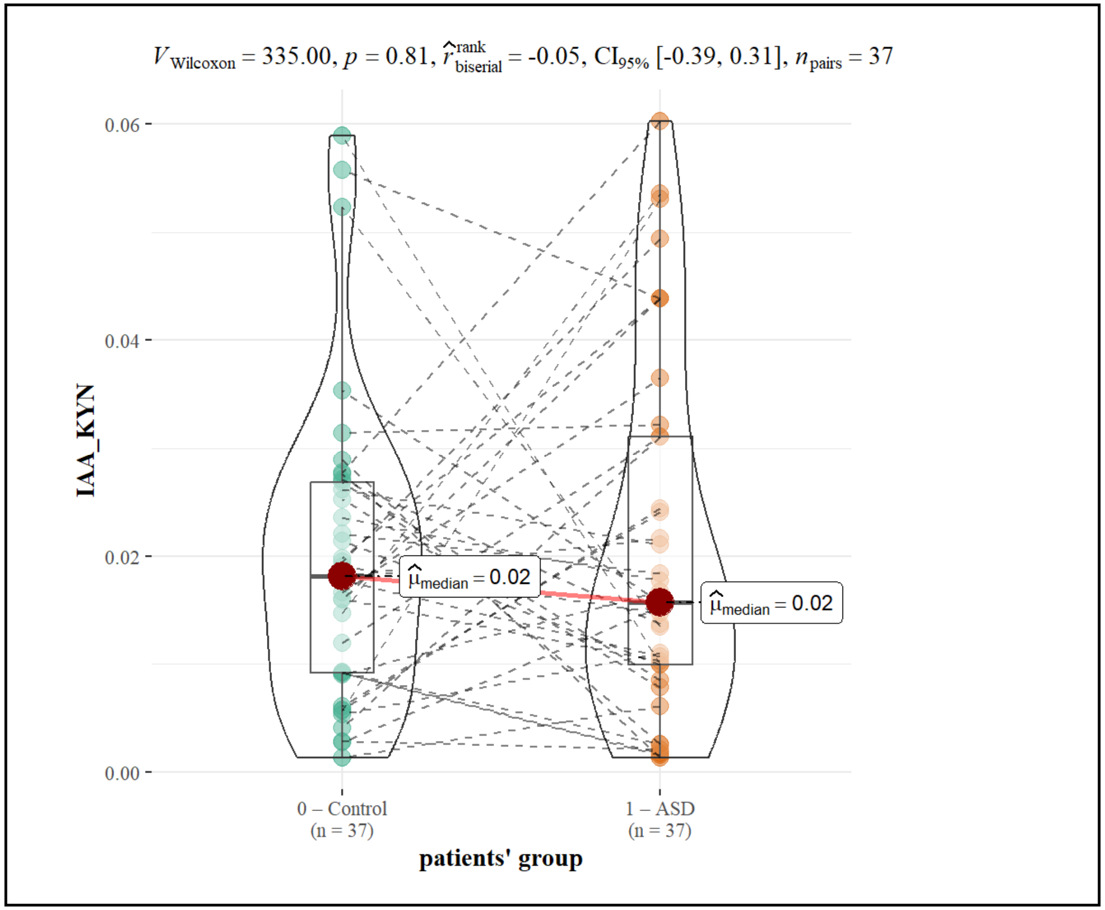

$V_{\text{Wilcoxon}} = 335.00$, $p = 0.81$, $\hat{r}^{\text{rank}}_{\text{biserial}} = -0.05$, $\text{CI}_{95\%}$ [-0.39, 0.31], $n_{\text{pairs}} = 37$
0.06
0.04
0.02
0.00
IAA_KYN
$\hat{\mu}_{\text{median}} = 0.02$
$\hat{\mu}_{\text{median}} = 0.02$
0 – Control
(n = 37)
1 – ASD
(n = 37)
patients' group

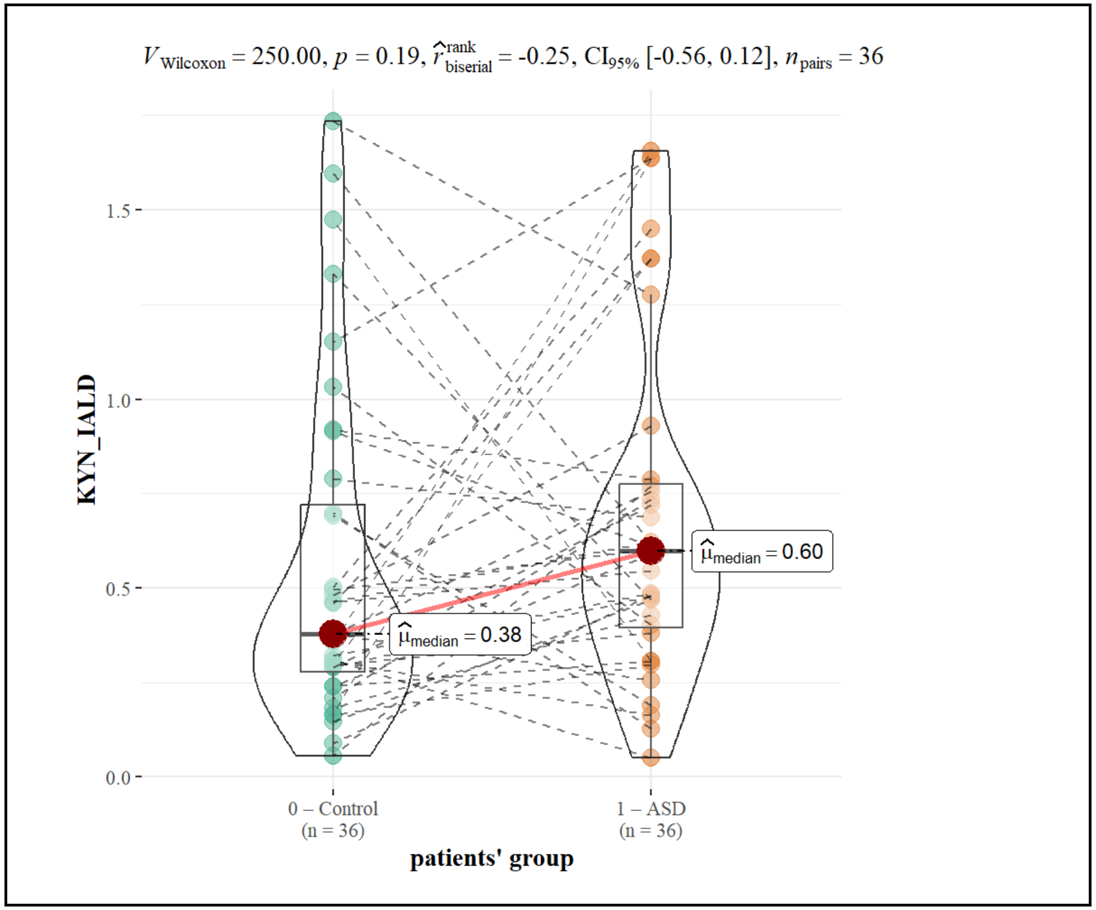

$V_{Wilcoxon}$ = 250.00, p = 0.19, $\hat{r}^{rank}_{biserial}$ = -0.25, $CI_{95\%}$ [-0.56, 0.12], n_{pairs} = 36
KYN_IALD
1.5
1.0
0.5
0.0
$\hat{\mu}_{median}$ = 0.38
$\hat{\mu}_{median}$ = 0.60
0 – Control
(n = 36)
1 – ASD
(n = 36)
patients' group

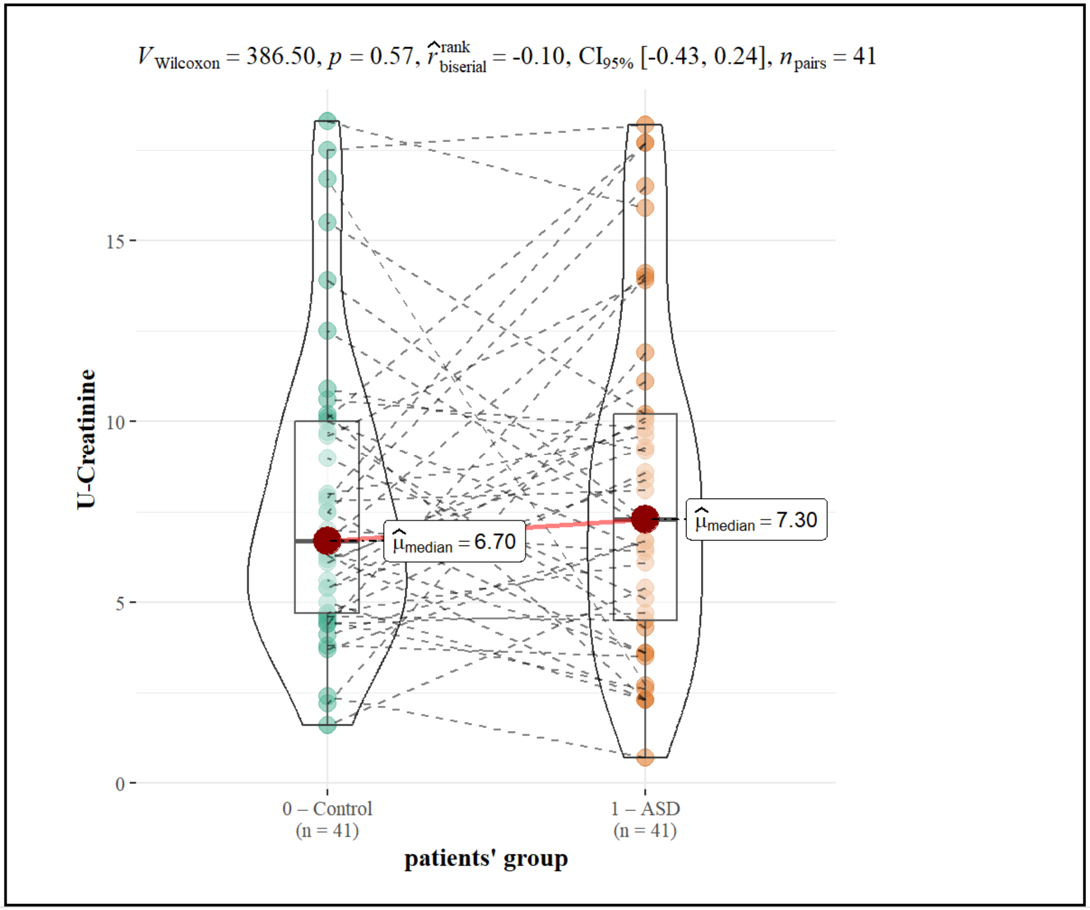
$V_{Wilcoxon}$ = 386.50, p = 0.57, $\hat{r}^{rank}_{biserial}$ = -0.10, $CI_{95\%}$ [-0.43, 0.24], n_{pairs} = 41
U-Creatinine
0
5
10
15
$\hat{\mu}_{median}$ = 6.70
$\hat{\mu}_{median}$ = 7.30
0 – Control
(n = 41)
1 – ASD
(n = 41)
patients' group

1 – 3 ASSOCIATION

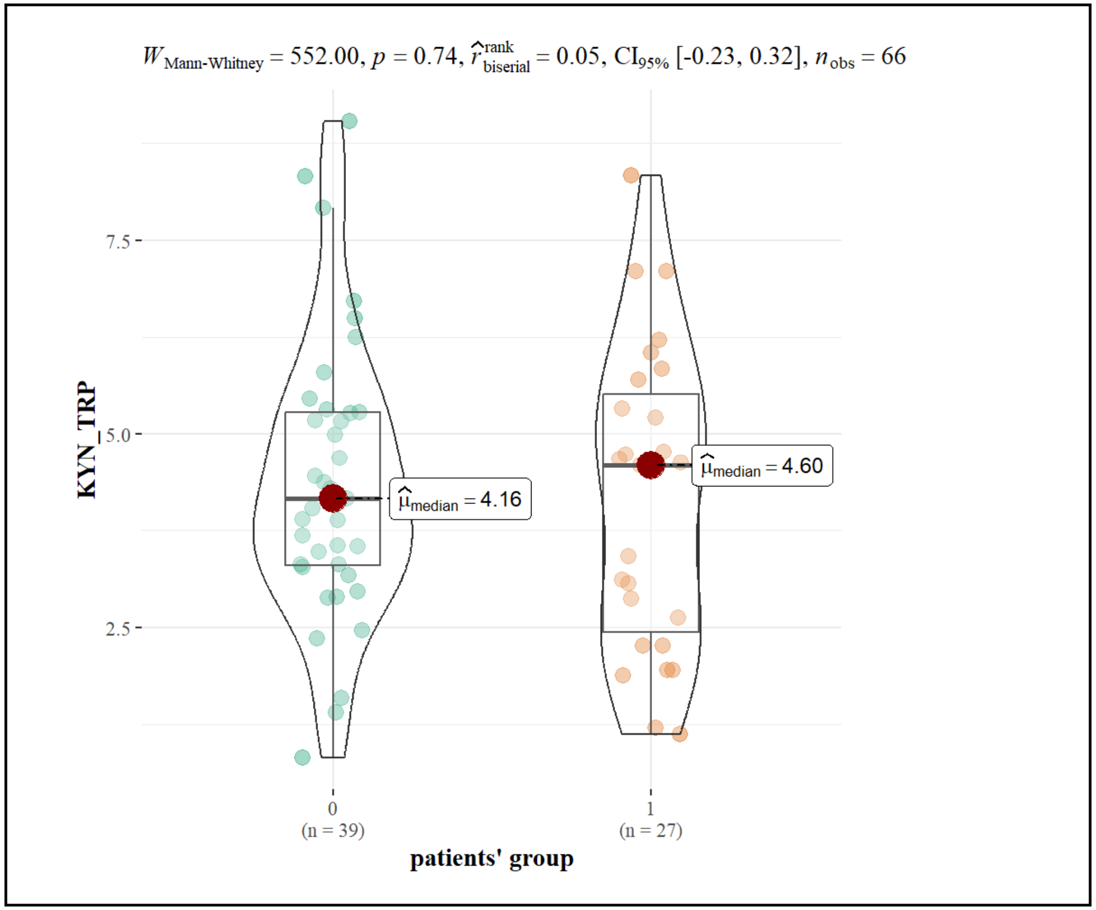

$W_{\text{Mann-Whitney}} = 552.00$, $p = 0.74$, $\hat{r}_{\text{biserial}}^{\text{rank}} = 0.05$, $\text{CI}_{95\%}$ [-0.23, 0.32], $n_{\text{obs}} = 66$
KYN_TRP
7.5
5.0
2.5
$\hat{\mu}_{\text{median}} = 4.16$
$\hat{\mu}_{\text{median}} = 4.60$
0
(n = 39)
1
(n = 27)
patients' group

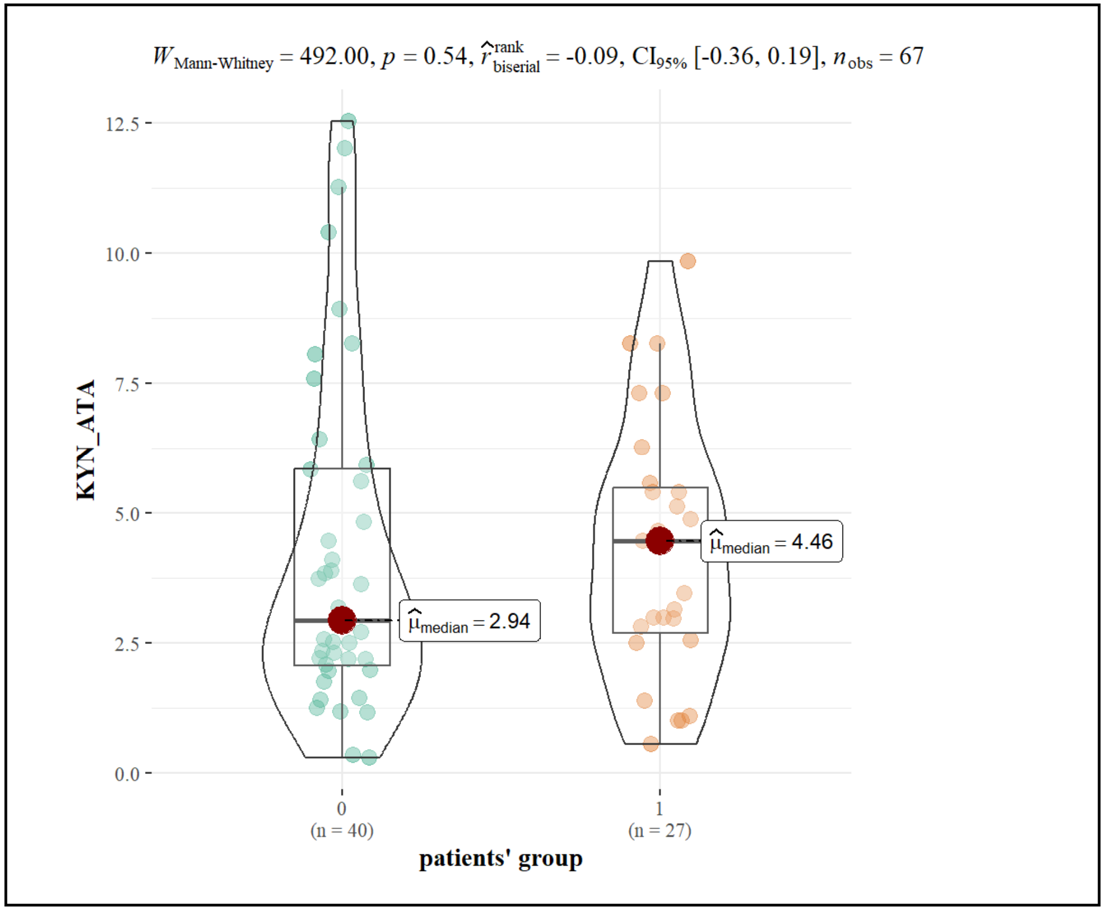
$W_{\text{Mann-Whitney}}$ = 492.00, p = 0.54, $\hat{r}^{\text{rank}}_{\text{biserial}}$ = -0.09, $CI_{95\%}$ [-0.36, 0.19], n_{obs} = 67
KYN_ATA
12.5
10.0
7.5
5.0
2.5
0.0
$\hat{\mu}_{\text{median}}$ = 2.94
$\hat{\mu}_{\text{median}}$ = 4.46
0
(n = 40)
1
(n = 27)
patients' group

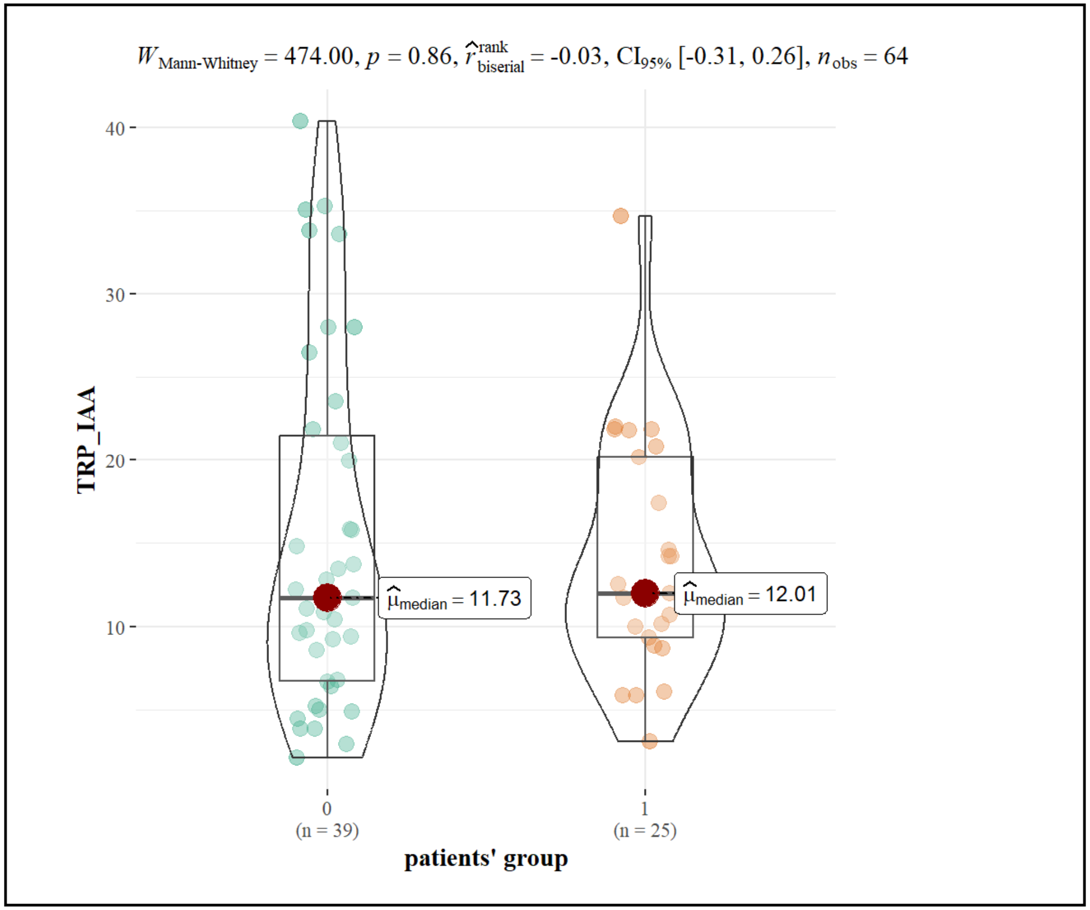
$W_{\text{Mann-Whitney}} = 474.00$, $p = 0.86$, $\widehat{r}_{\text{biserial}}^{\text{rank}} = -0.03$, $CI_{95\%}$ [-0.31, 0.26], $n_{\text{obs}} = 64$
TRP_IAA
40
30
20
10
$\widehat{\mu}_{\text{median}} = 11.73$
$\widehat{\mu}_{\text{median}} = 12.01$
0
(n = 39)
1
(n = 25)
patients' group

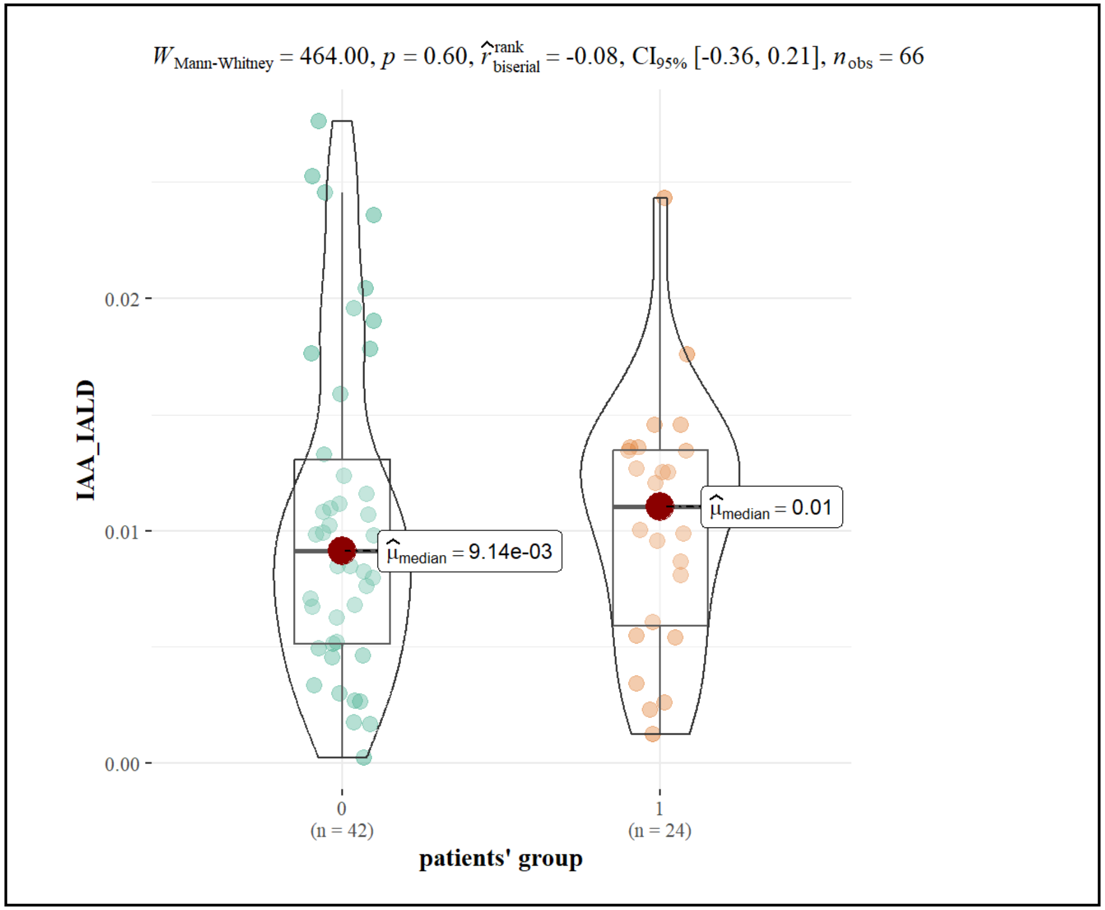

$W_{\text{Mann-Whitney}} = 464.00$, $p = 0.60$, $\hat{r}_{\text{biserial}}^{\text{rank}} = -0.08$, $\text{CI}_{95\%}$ [-0.36, 0.21], $n_{\text{obs}} = 66$
IAA_IALD
0.02
0.01
0.00
$\hat{\mu}_{\text{median}} = 9.14\text{e-}03$
$\hat{\mu}_{\text{median}} = 0.01$
0
(n = 42)
1
(n = 24)
patients' group

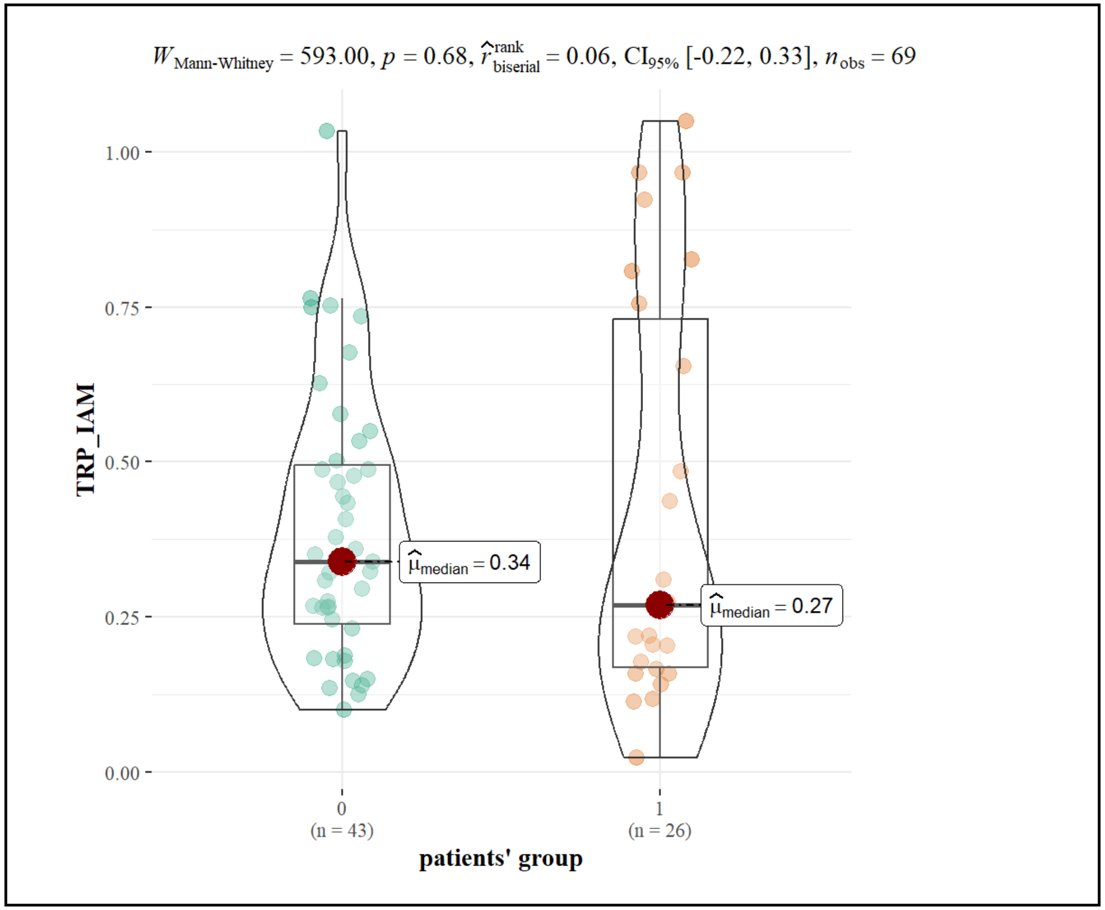

$W_{\text{Mann-Whitney}} = 593.00$, $p = 0.68$, $\hat{r}_{\text{biserial}}^{\text{rank}} = 0.06$, $\text{CI}_{95\%}$ [-0.22, 0.33], $n_{\text{obs}} = 69$
TRP_IAM
1.00
0.75
0.50
0.25
0.00
$\hat{\mu}_{\text{median}} = 0.34$
$\hat{\mu}_{\text{median}} = 0.27$
0
(n = 43)
1
(n = 26)
patients' group

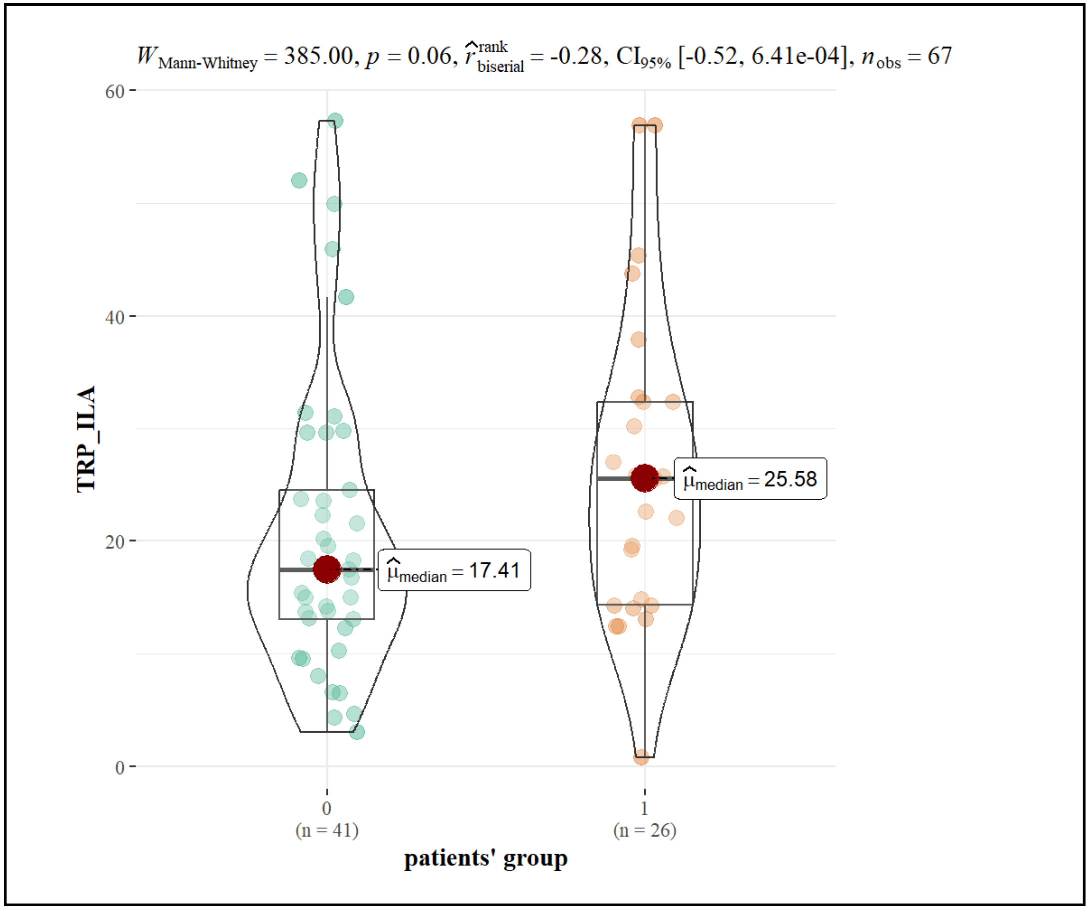

$W_{\text{Mann-Whitney}} = 385.00$, $p = 0.06$, $\hat{r}^{\text{rank}}_{\text{biserial}} = -0.28$, $\text{CI}_{95\%}$ [-0.52, 6.41e-04], $n_{\text{obs}} = 67$
TRP_ILA
60
40
20
0
$\hat{\mu}_{\text{median}} = 17.41$
$\hat{\mu}_{\text{median}} = 25.58$
0
(n = 41)
1
(n = 26)
patients' group

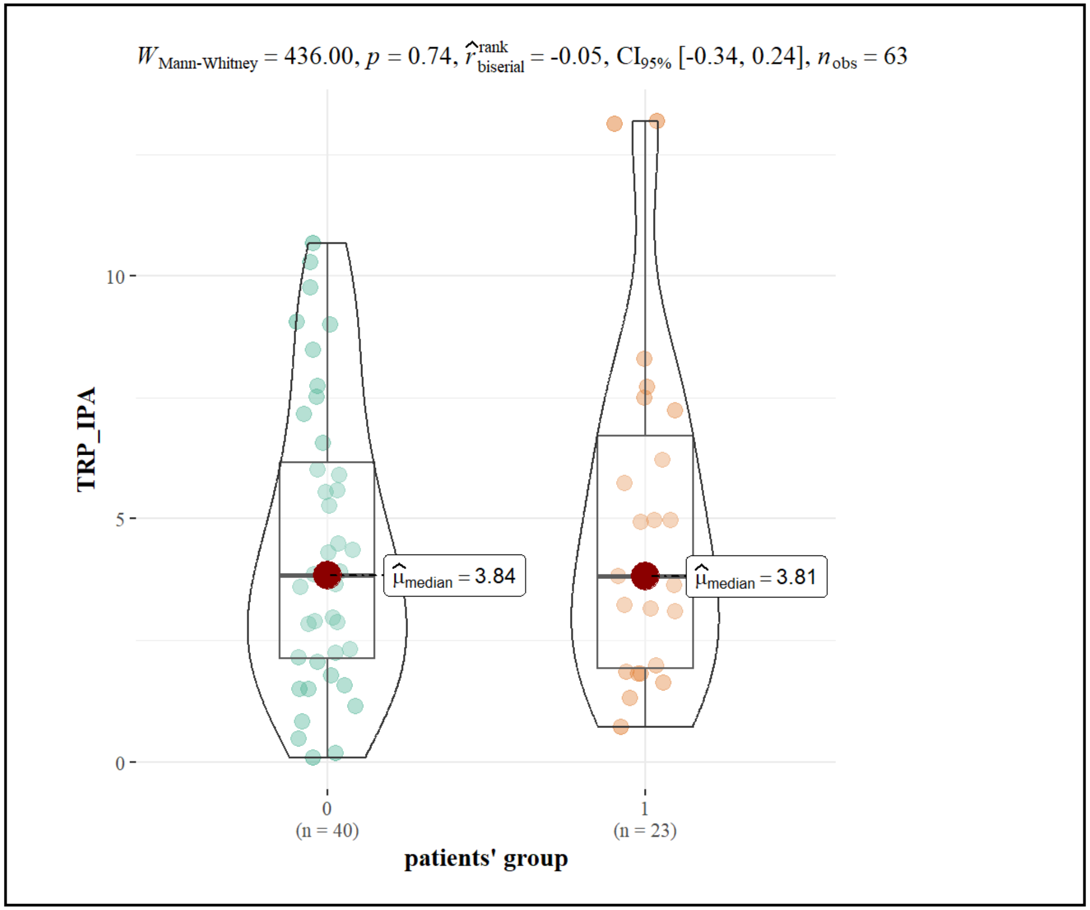
$W_{\text{Mann-Whitney}} = 436.00$, $p = 0.74$, $\hat{r}_{\text{biserial}}^{\text{rank}} = -0.05$, $\text{CI}_{95\%}$ [-0.34, 0.24], $n_{\text{obs}} = 63$
TRP_IPA
10
5
0
$\hat{\mu}_{\text{median}} = 3.84$
$\hat{\mu}_{\text{median}} = 3.81$
0
(n = 40)
1
(n = 23)
patients' group

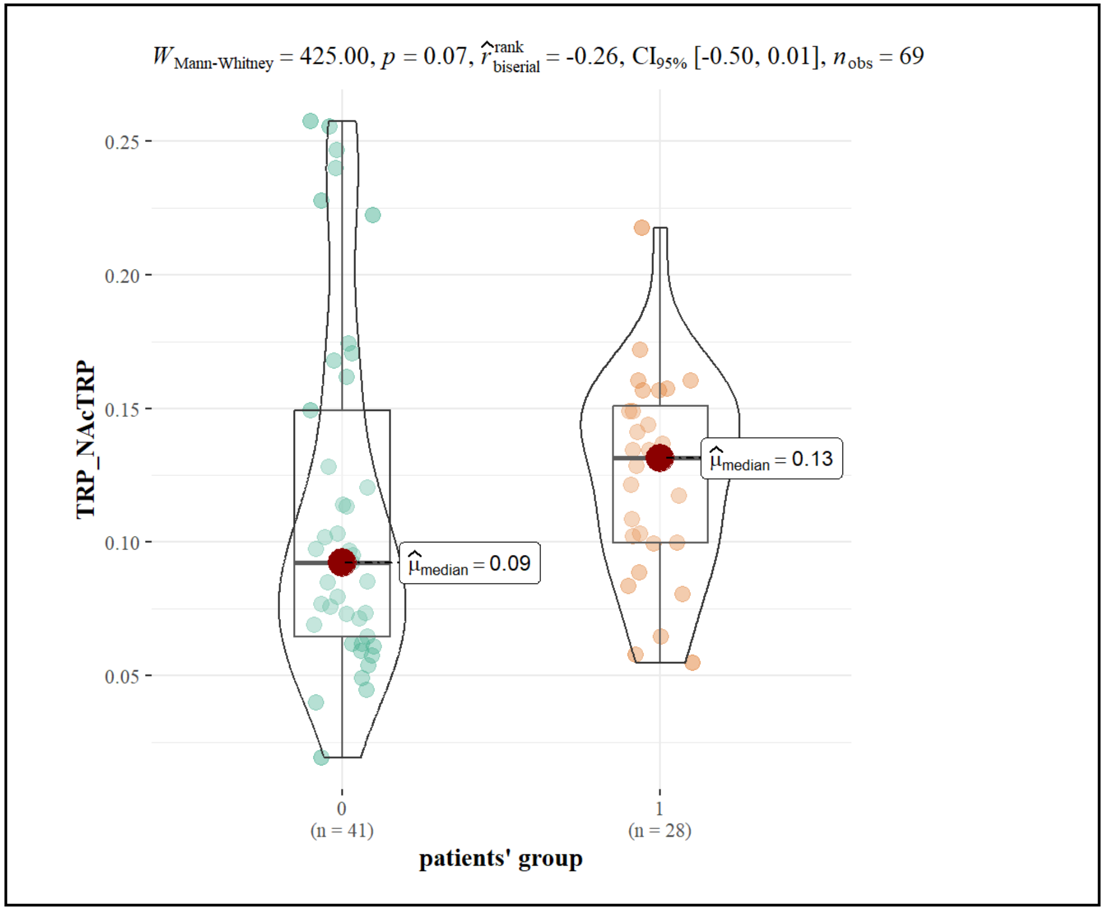

$W_{\text{Mann-Whitney}} = 425.00$, $p = 0.07$, $\hat{r}_{\text{biserial}}^{\text{rank}} = -0.26$, $\text{CI}_{95\%}$ [-0.50, 0.01], $n_{\text{obs}} = 69$
TRP_NAcTRP
0.25
0.20
0.15
0.10
0.05
$\hat{\mu}_{\text{median}} = 0.09$
$\hat{\mu}_{\text{median}} = 0.13$
0
(n = 41)
1
(n = 28)
patients' group

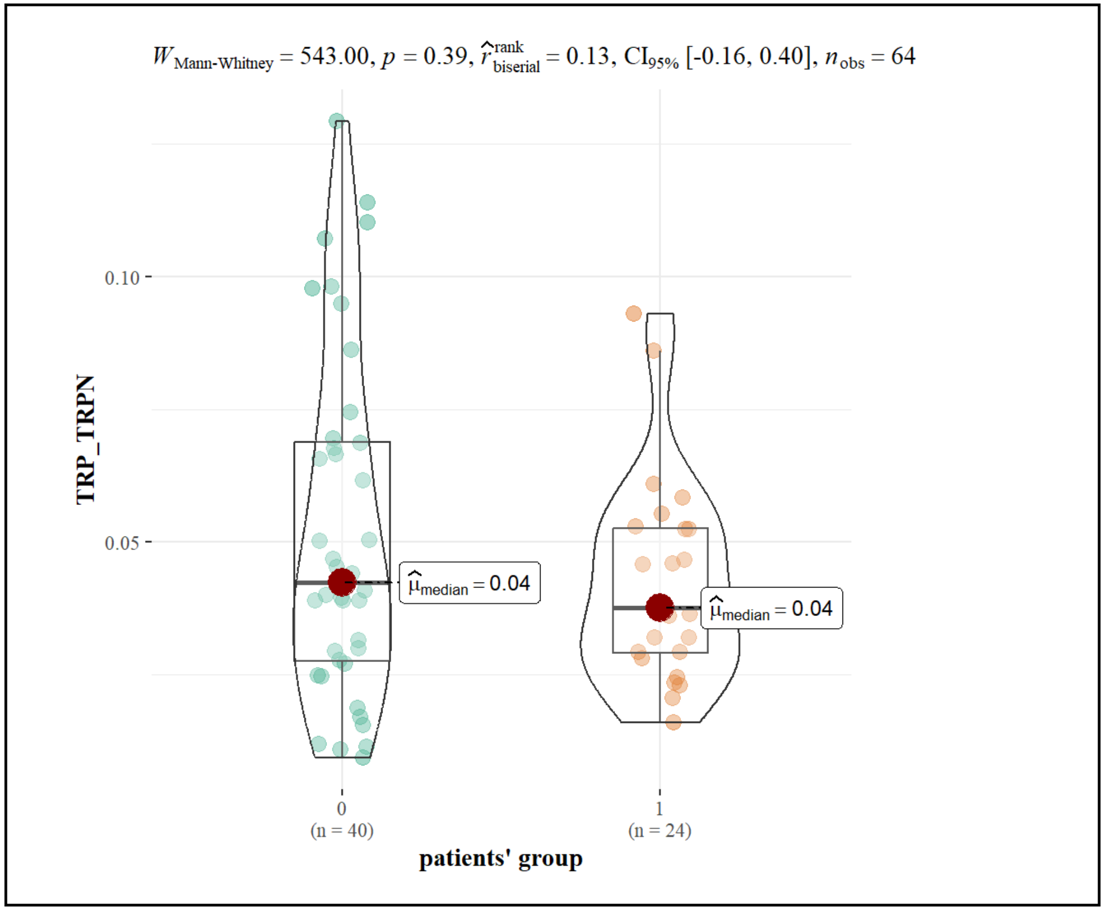
$W_{\text{Mann-Whitney}} = 543.00$, $p = 0.39$, $\hat{r}^{\text{rank}}_{\text{biserial}} = 0.13$, $\text{CI}_{95\%}$ [-0.16, 0.40], $n_{\text{obs}} = 64$
TRP_TRPN
0.10
0.05
$\hat{\mu}_{\text{median}} = 0.04$
$\hat{\mu}_{\text{median}} = 0.04$
0
(n = 40)
1
(n = 24)
patients' group

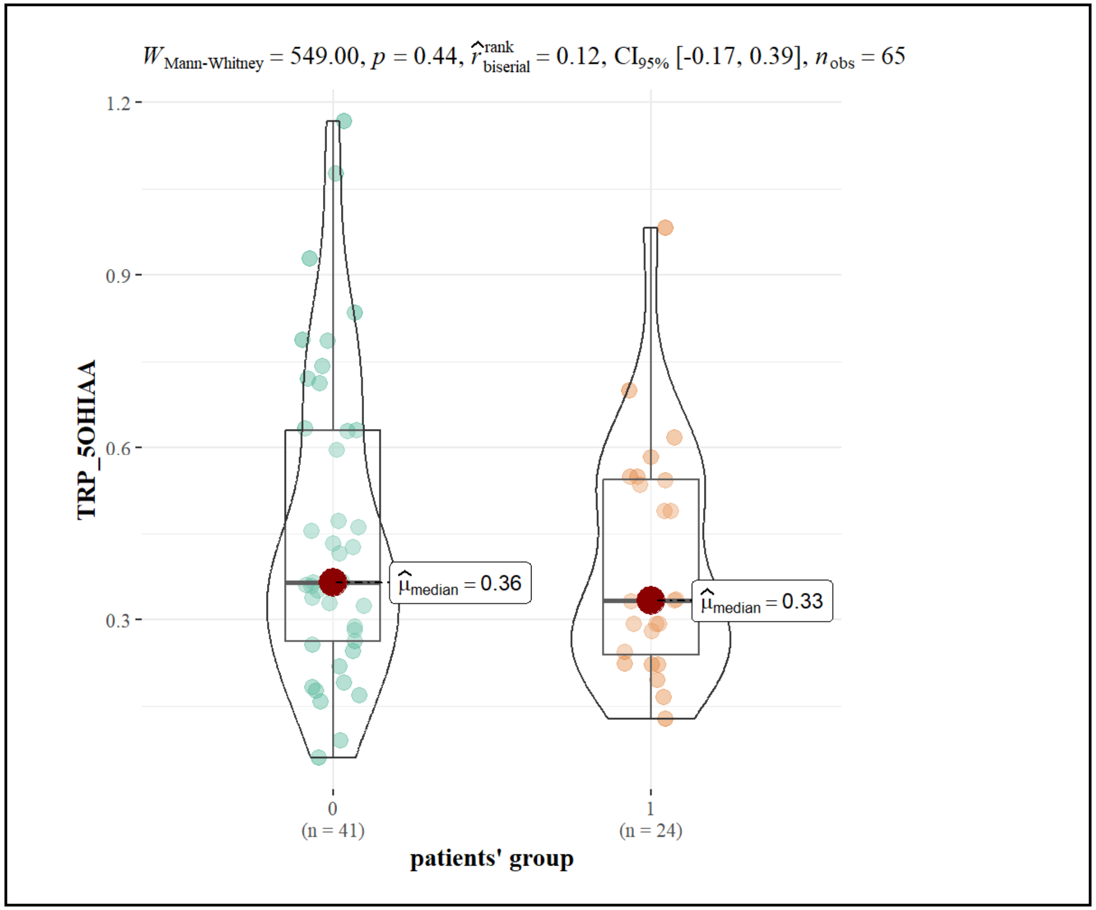

$W_{\text{Mann-Whitney}} = 549.00$, $p = 0.44$, $\widehat{r}_{\text{biserial}}^{\text{rank}} = 0.12$, $\text{CI}_{95\%}$ [-0.17, 0.39], $n_{\text{obs}} = 65$
TRP_5OHIAA
1.2
0.9
0.6
0.3
$\widehat{\mu}_{\text{median}} = 0.36$
$\widehat{\mu}_{\text{median}} = 0.33$
0
(n = 41)
1
(n = 24)
patients' group

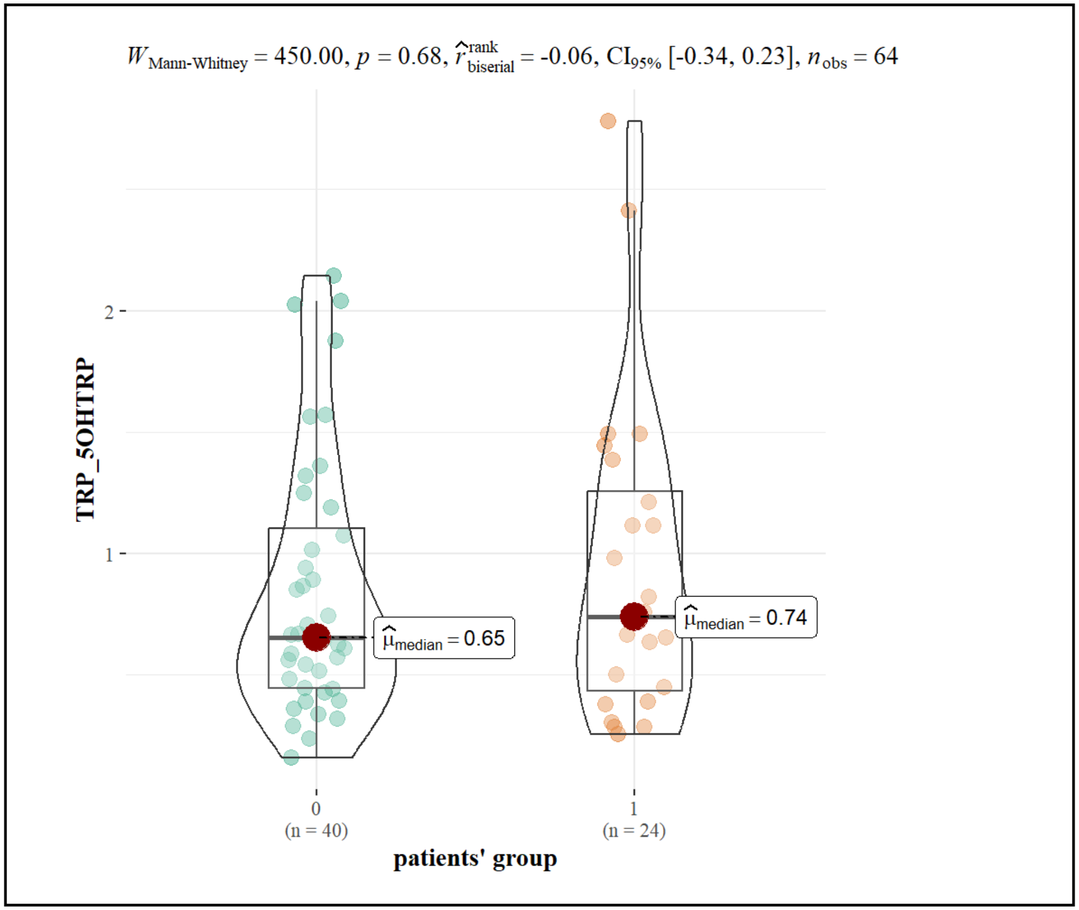

$W_{\text{Mann-Whitney}} = 450.00$, $p = 0.68$, $\hat{r}^{\text{rank}}_{\text{biserial}} = -0.06$, $\text{CI}_{95\%}$ [-0.34, 0.23], $n_{\text{obs}} = 64$
TRP_5OHTRP
2
1
$\hat{\mu}_{\text{median}} = 0.65$
$\hat{\mu}_{\text{median}} = 0.74$
0
(n = 40)
1
(n = 24)
patients' group

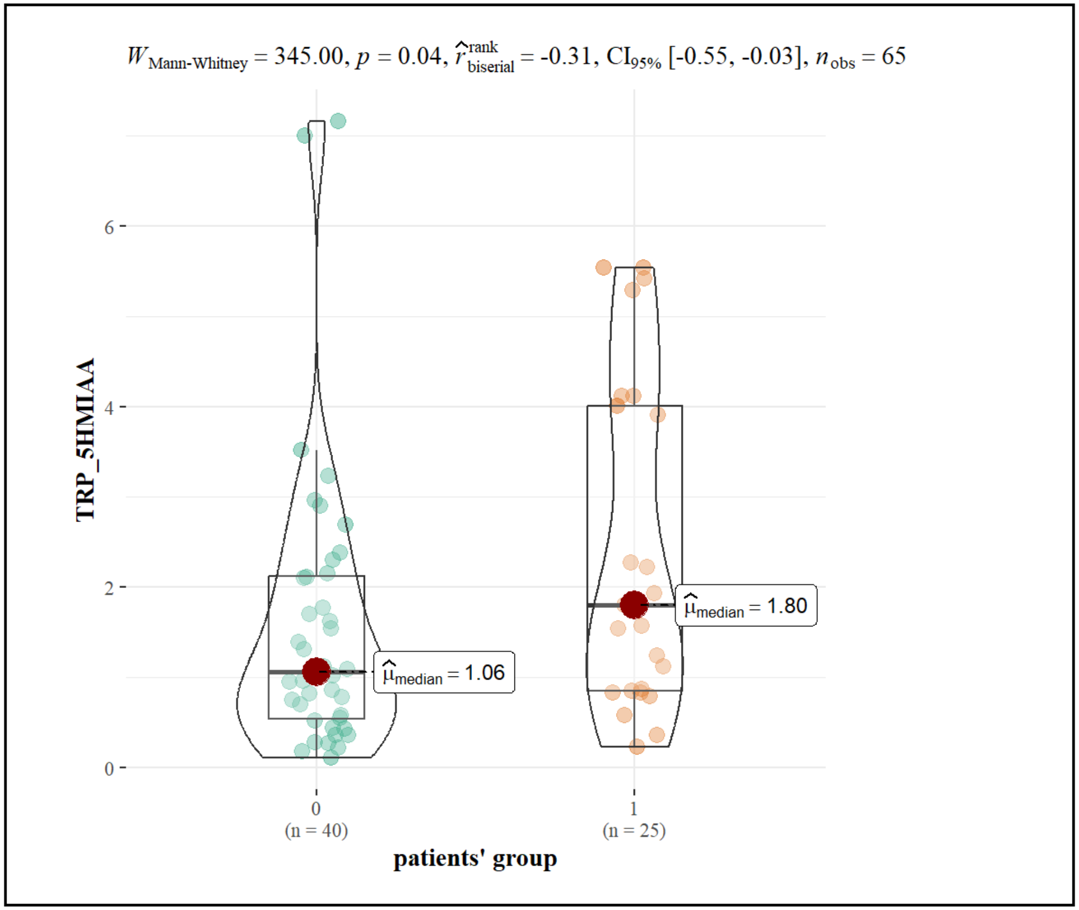
$W_{\text{Mann-Whitney}} = 345.00$, $p = 0.04$, $\hat{r}_{\text{biserial}}^{\text{rank}} = -0.31$, $\text{CI}_{95\%}$ [-0.55, -0.03], $n_{\text{obs}} = 65$
TRP_5HMIAA
$\hat{\mu}_{\text{median}} = 1.06$
$\hat{\mu}_{\text{median}} = 1.80$
0
2
4
6
0
(n = 40)
1
(n = 25)
patients' group

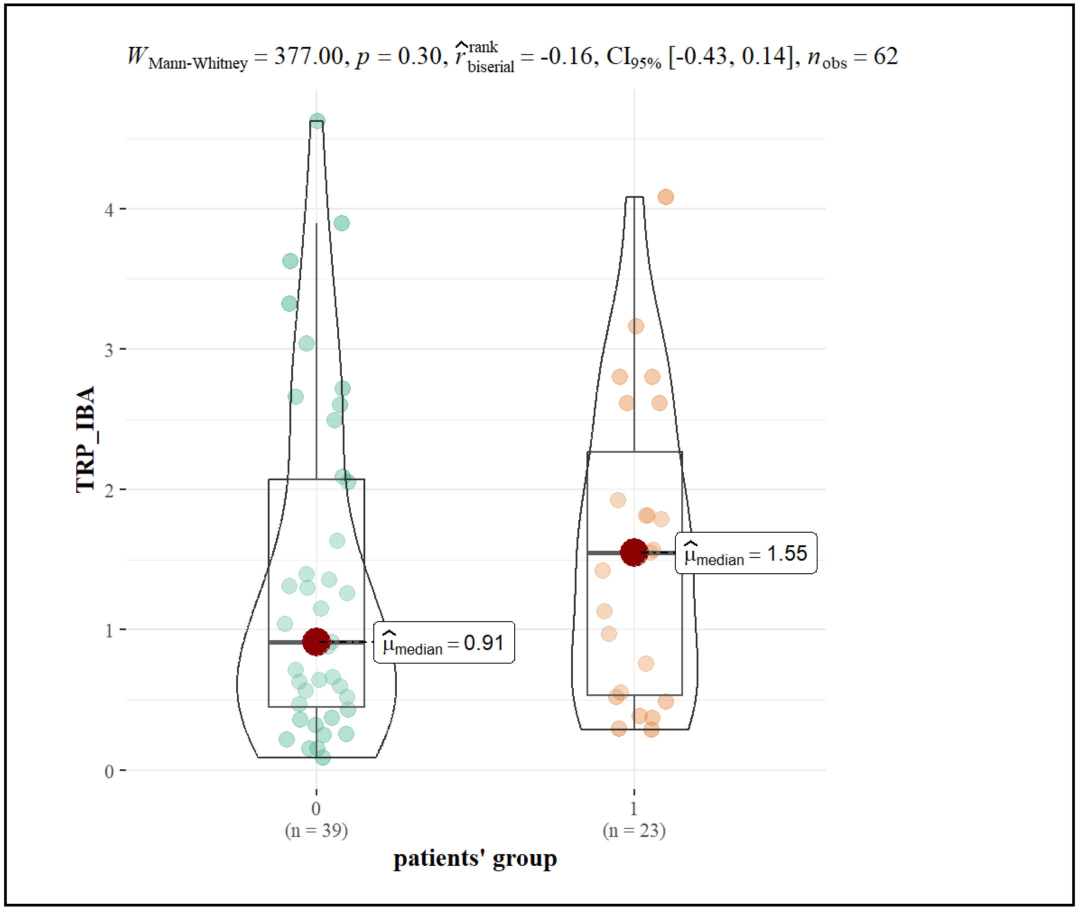

$W_{\text{Mann-Whitney}} = 377.00$, $p = 0.30$, $\hat{r}^{\text{rank}}_{\text{biserial}} = -0.16$, $\text{CI}_{95\%}$ [-0.43, 0.14], $n_{\text{obs}} = 62$
TRP_IBA
4
3
2
1
0
$\hat{\mu}_{\text{median}} = 0.91$
$\hat{\mu}_{\text{median}} = 1.55$
0
(n = 39)
1
(n = 23)
patients' group

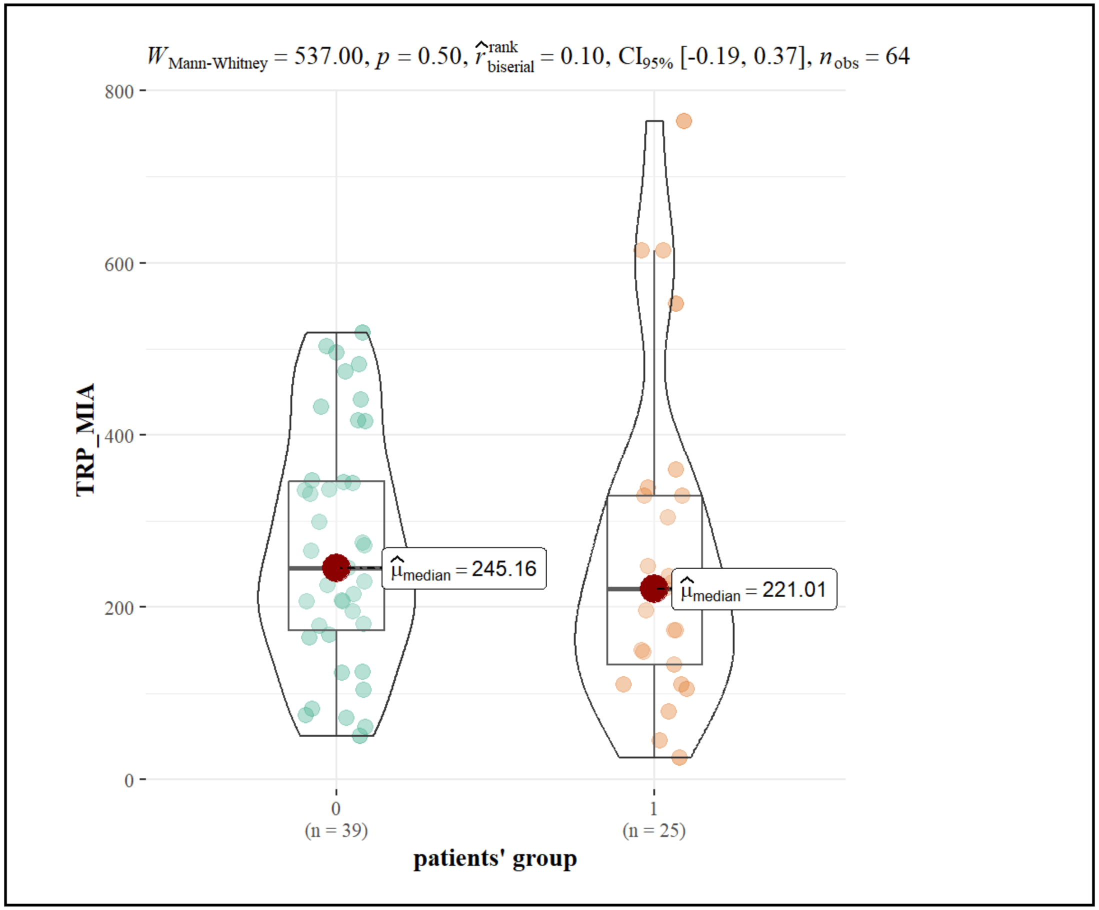

$W_{\text{Mann-Whitney}}$ = 537.00, p = 0.50, $\hat{r}_{\text{biserial}}^{\text{rank}}$ = 0.10, $CI_{95\%}$ [-0.19, 0.37], n_{obs} = 64
800
600
400
200
0
TRP_MIA
$\hat{\mu}_{\text{median}}$ = 245.16
$\hat{\mu}_{\text{median}}$ = 221.01
0
(n = 39)
1
(n = 25)
patients' group

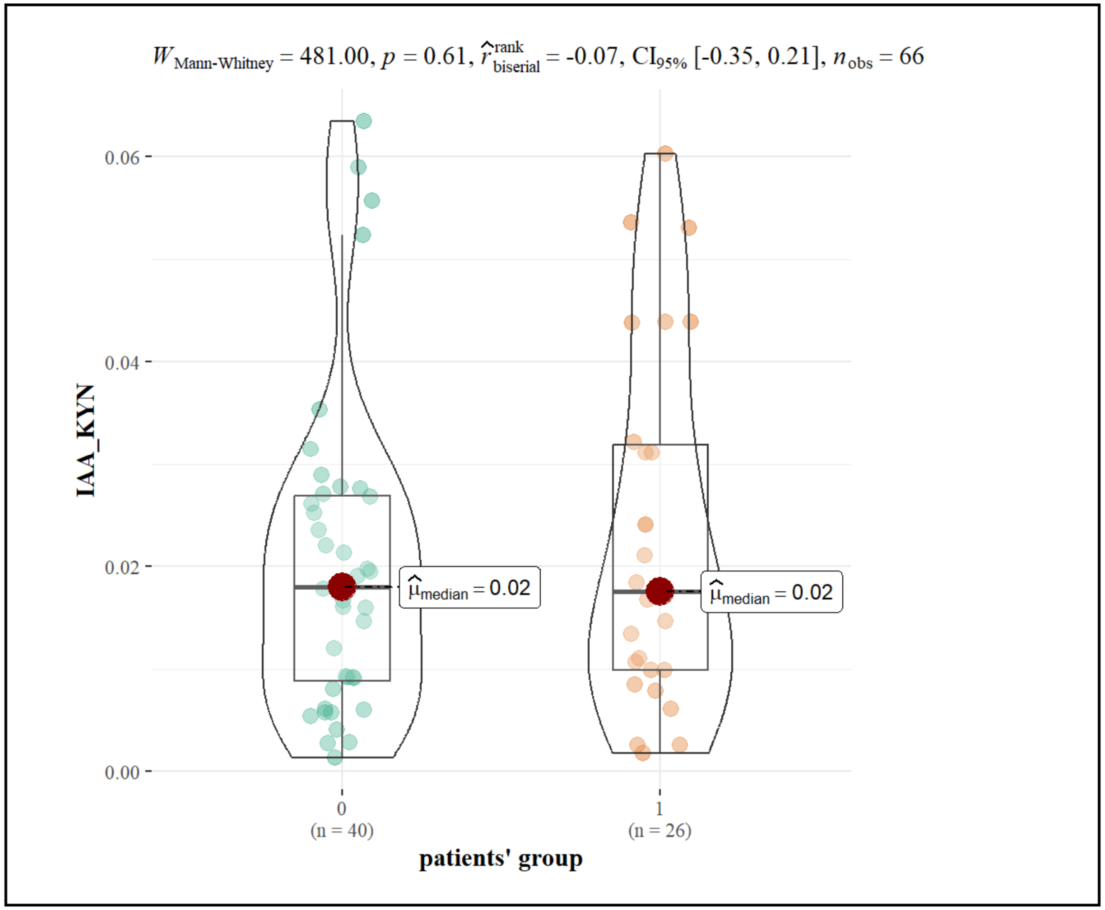

$W_{\text{Mann-Whitney}}$ = 481.00, p = 0.61, $\hat{r}^{\text{rank}}_{\text{biserial}}$ = -0.07, $\text{CI}_{95\%}$ [-0.35, 0.21], n_{obs} = 66
0.06
0.04
0.02
0.00
IAA_KYN
$\hat{\mu}_{\text{median}}$ = 0.02
$\hat{\mu}_{\text{median}}$ = 0.02
0
(n = 40)
1
(n = 26)
patients' group

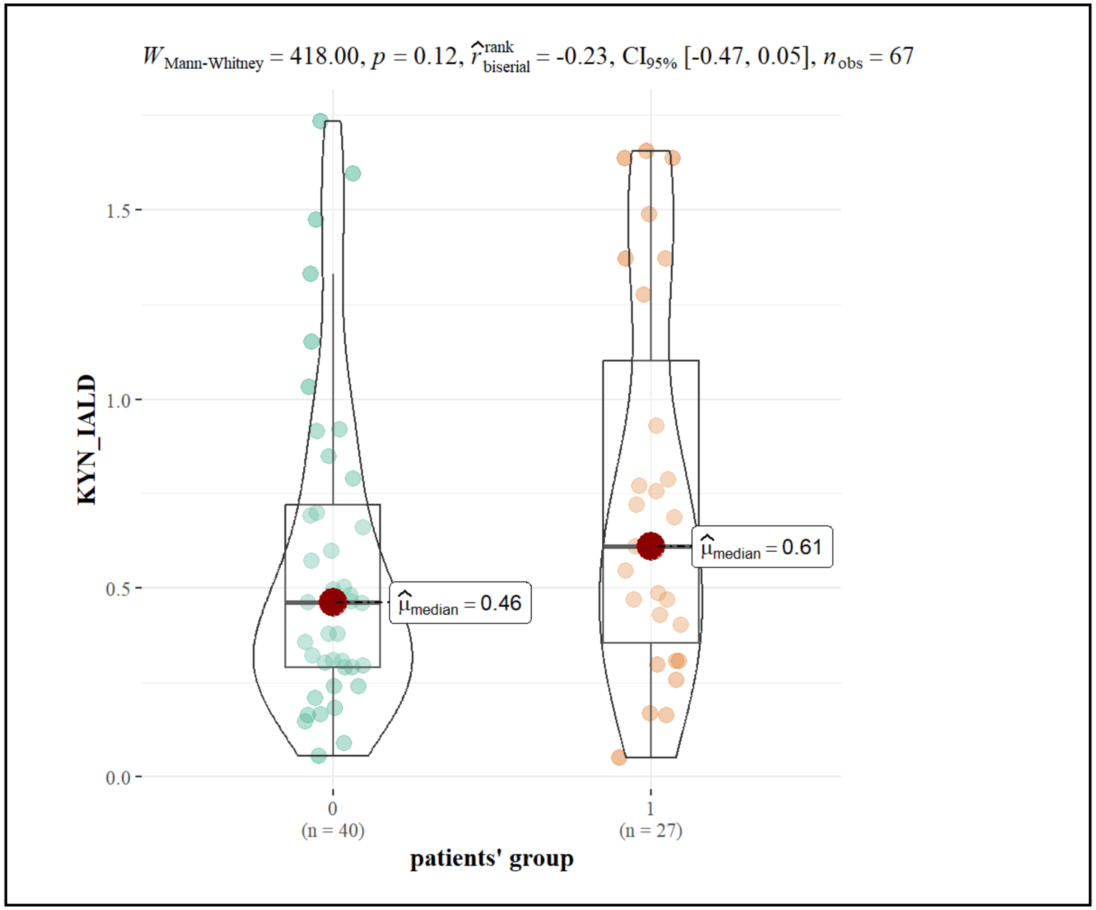

$W_{\text{Mann-Whitney}} = 418.00$, $p = 0.12$, $\hat{r}_{\text{biserial}}^{\text{rank}} = -0.23$, $\text{CI}_{95\%}$ [-0.47, 0.05], $n_{\text{obs}} = 67$
KYN_IALD
1.5
1.0
0.5
0.0
$\hat{\mu}_{\text{median}} = 0.46$
$\hat{\mu}_{\text{median}} = 0.61$
0
(n = 40)
1
(n = 27)
patients' group

1 – 4 ASSOCIATION

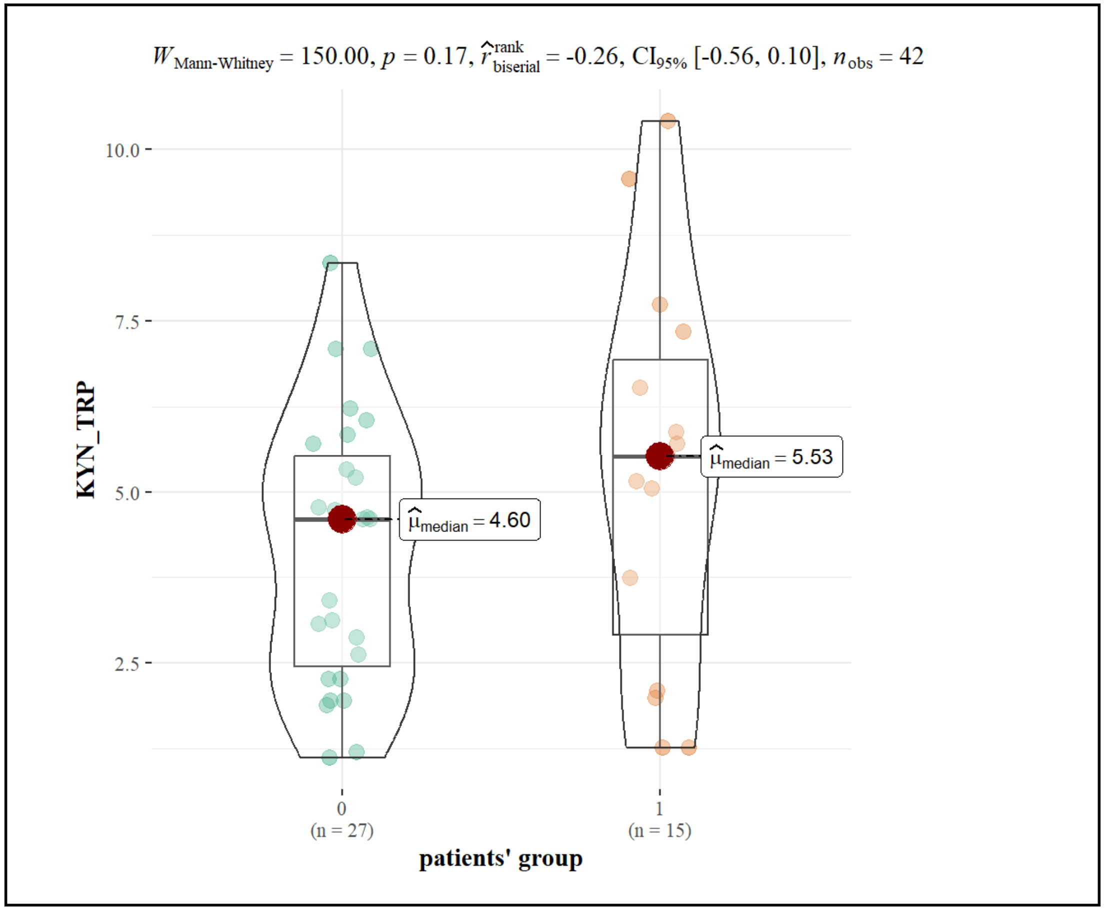

$W_{\text{Mann-Whitney}}$ = 150.00, p = 0.17, $\hat{r}^{\text{rank}}_{\text{biserial}}$ = -0.26, $\text{CI}_{95\%}$ [-0.56, 0.10], n_{obs} = 42
KYN_TRP
10.0
7.5
5.0
2.5
$\hat{\mu}_{\text{median}}$ = 4.60
$\hat{\mu}_{\text{median}}$ = 5.53
0
(n = 27)
1
(n = 15)
patients' group

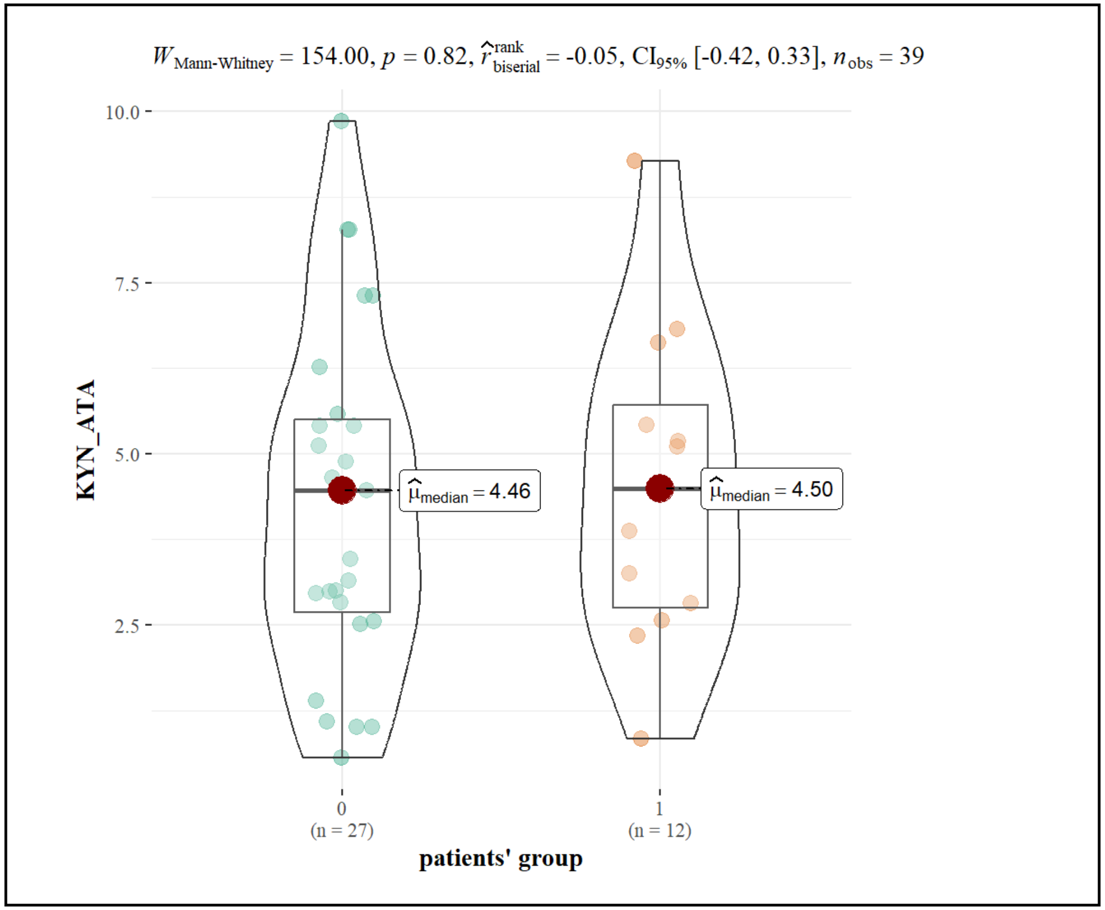

$W_{\text{Mann-Whitney}} = 154.00$, $p = 0.82$, $\hat{r}_{\text{biserial}}^{\text{rank}} = -0.05$, $\text{CI}_{95\%}$ [-0.42, 0.33], $n_{\text{obs}} = 39$
10.0
7.5
5.0
2.5
KYN_ATA
$\hat{\mu}_{\text{median}} = 4.46$
$\hat{\mu}_{\text{median}} = 4.50$
0
(n = 27)
1
(n = 12)
patients' group

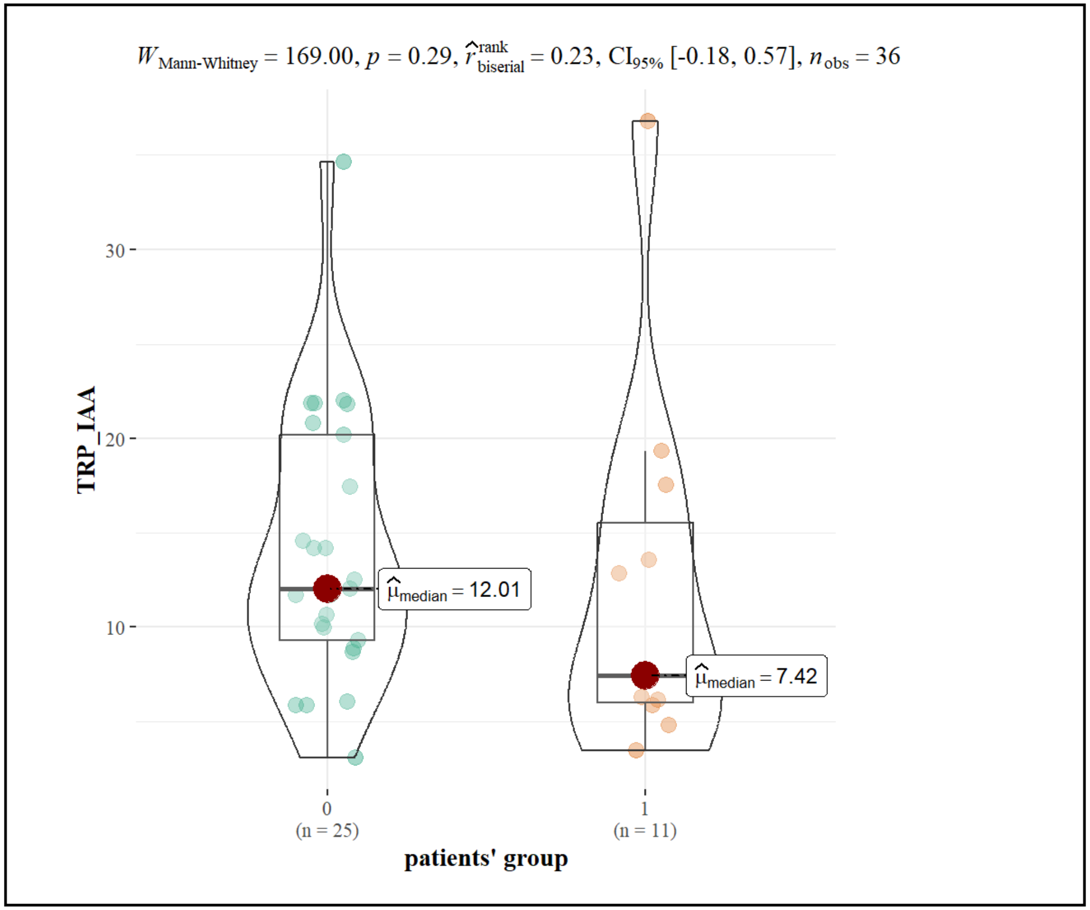
W Mann-Whitney = 169.00, p = 0.29, r̂ rank biserial = 0.23, CI95% [-0.18, 0.57], n obs = 36
TRP_IAA
30
20
10
μ̂ median = 12.01
μ̂ median = 7.42
0
(n = 25)
1
(n = 11)
patients' group

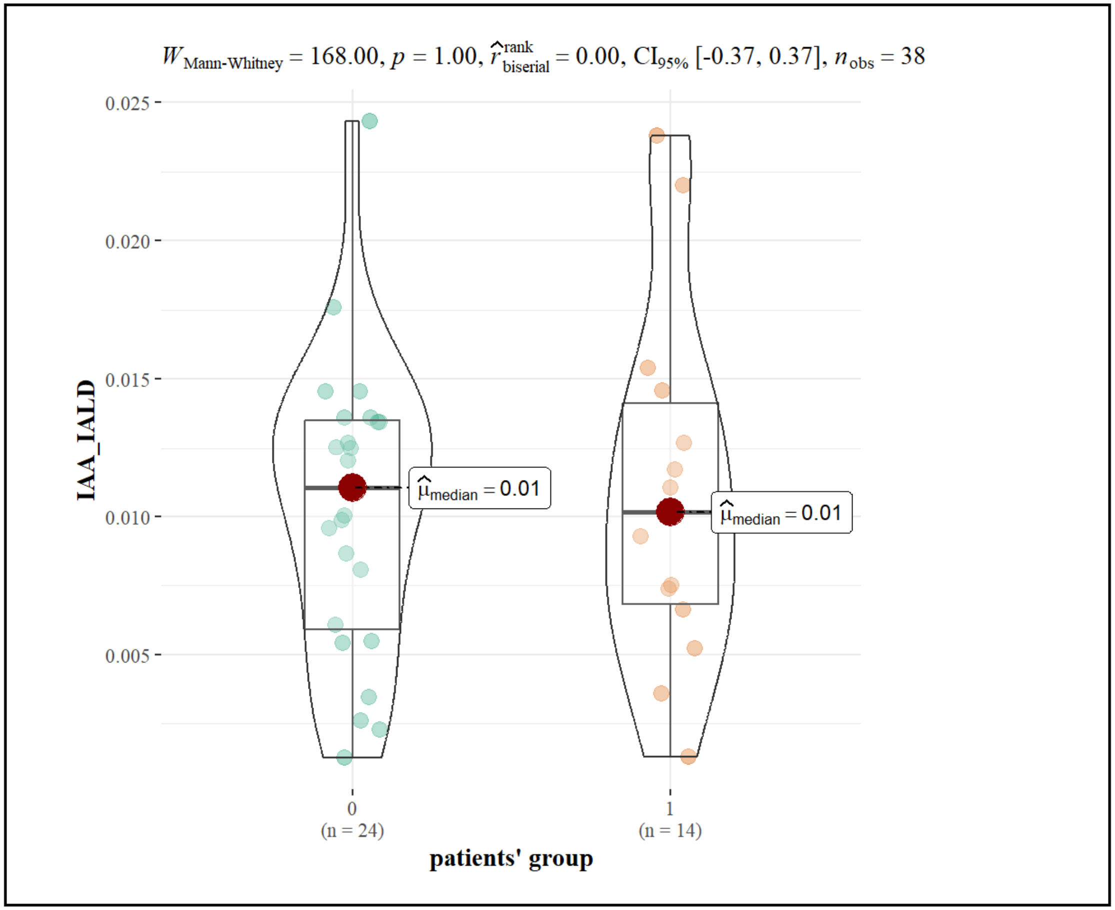

$W_{\text{Mann-Whitney}} = 168.00$, $p = 1.00$, $\widehat{r}_{\text{biserial}}^{\text{rank}} = 0.00$, $\text{CI}_{95\%}$ [-0.37, 0.37], $n_{\text{obs}} = 38$
0.025
0.020
0.015
0.010
0.005
IAA_IALD
$\widehat{\mu}_{\text{median}} = 0.01$
$\widehat{\mu}_{\text{median}} = 0.01$
0
(n = 24)
1
(n = 14)
patients' group

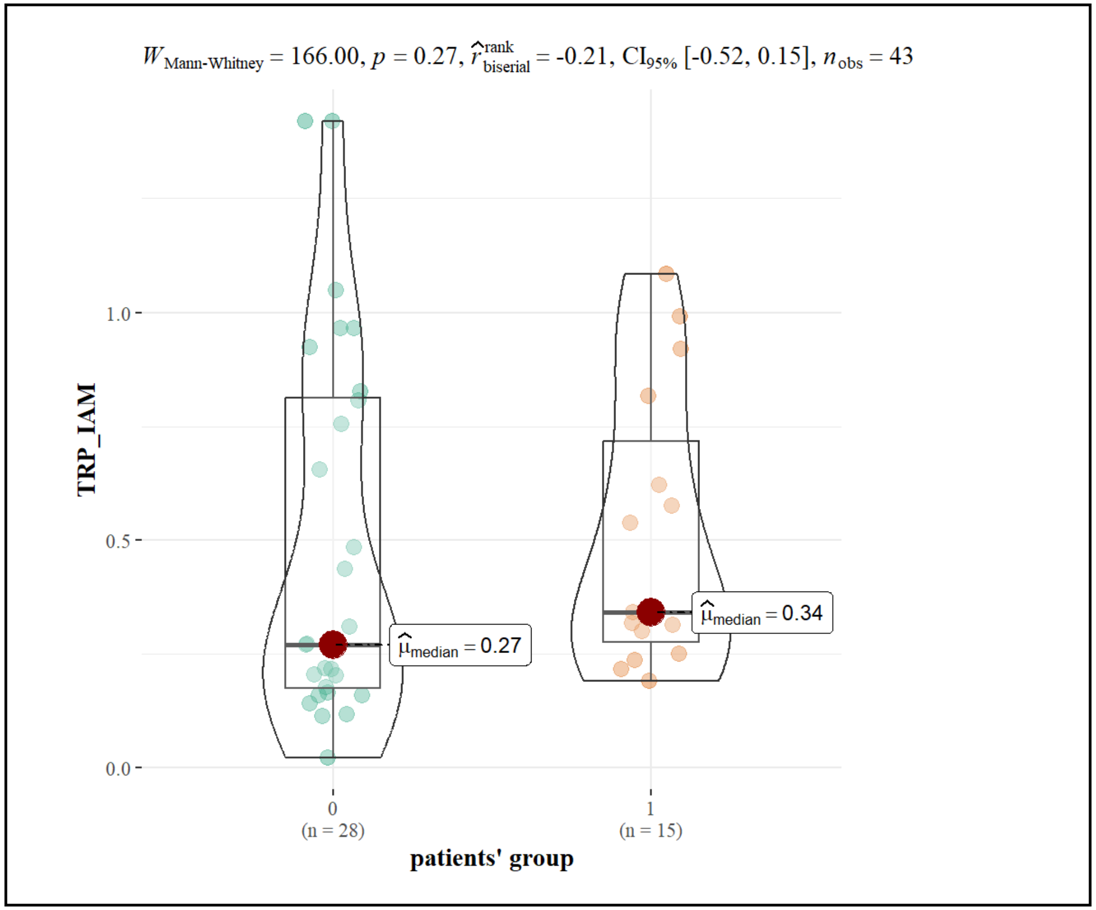

$W_{\text{Mann-Whitney}} = 166.00$, $p = 0.27$, $\hat{r}^{\text{rank}}_{\text{biserial}} = -0.21$, $\text{CI}_{95\%}$ [-0.52, 0.15], $n_{\text{obs}} = 43$
TRP_IAM
1.0
0.5
0.0
$\hat{\mu}_{\text{median}} = 0.27$
$\hat{\mu}_{\text{median}} = 0.34$
0
(n = 28)
1
(n = 15)
patients' group

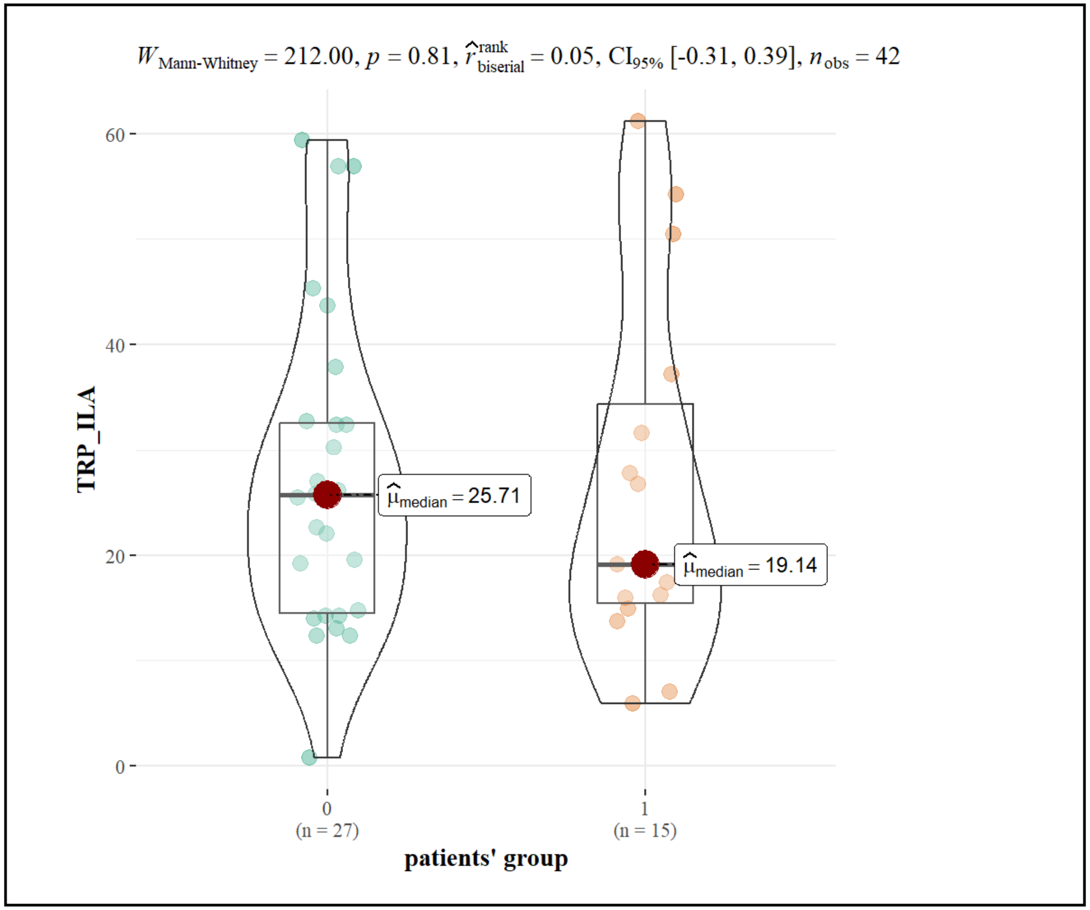

$W_{\text{Mann-Whitney}} = 212.00$, $p = 0.81$, $\hat{r}_{\text{biserial}}^{\text{rank}} = 0.05$, $\text{CI}_{95\%}$ [-0.31, 0.39], $n_{\text{obs}} = 42$
TRP_ILA
60
40
20
0
$\hat{\mu}_{\text{median}} = 25.71$
$\hat{\mu}_{\text{median}} = 19.14$
0
(n = 27)
1
(n = 15)
patients' group

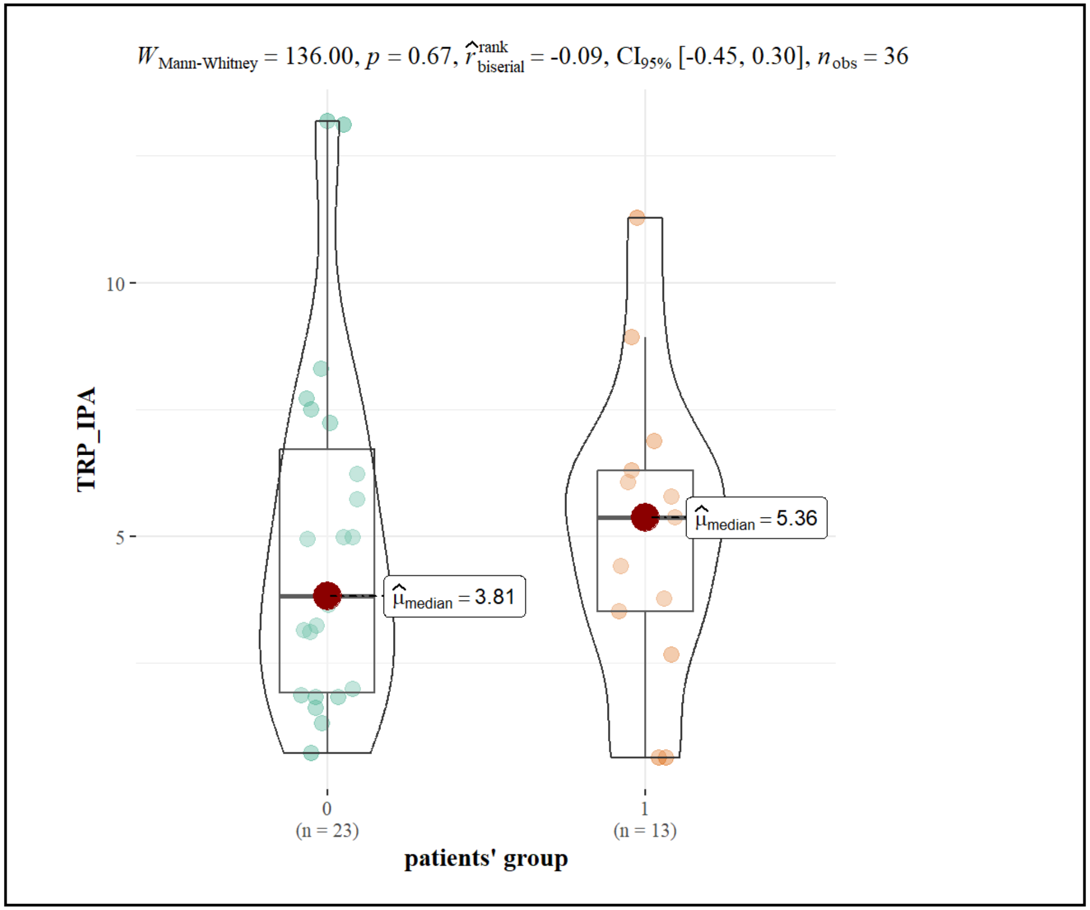

$W_{\text{Mann-Whitney}} = 136.00$, $p = 0.67$, $\hat{r}_{\text{biserial}}^{\text{rank}} = -0.09$, $\text{CI}_{95\%}$ [-0.45, 0.30], $n_{\text{obs}} = 36$
TRP_IPA
10
5
$\hat{\mu}_{\text{median}} = 3.81$
$\hat{\mu}_{\text{median}} = 5.36$
0
(n = 23)
1
(n = 13)
patients' group

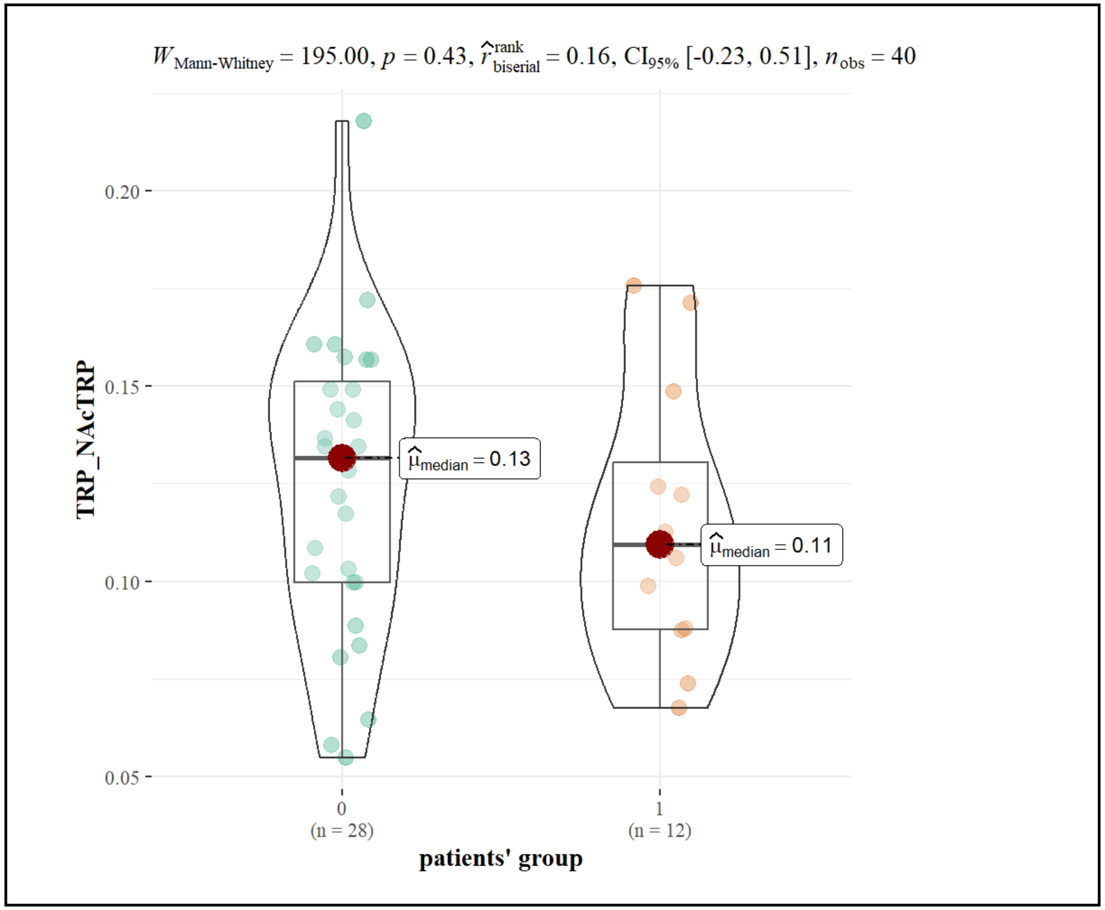

$W_{\text{Mann-Whitney}} = 195.00$, $p = 0.43$, $\hat{r}_{\text{biserial}}^{\text{rank}} = 0.16$, $\text{CI}_{95\%}$ [-0.23, 0.51], $n_{\text{obs}} = 40$
TRP_NAcTRP
0.20
0.15
0.10
0.05
$\hat{\mu}_{\text{median}} = 0.13$
$\hat{\mu}_{\text{median}} = 0.11$
0
(n = 28)
1
(n = 12)
patients' group

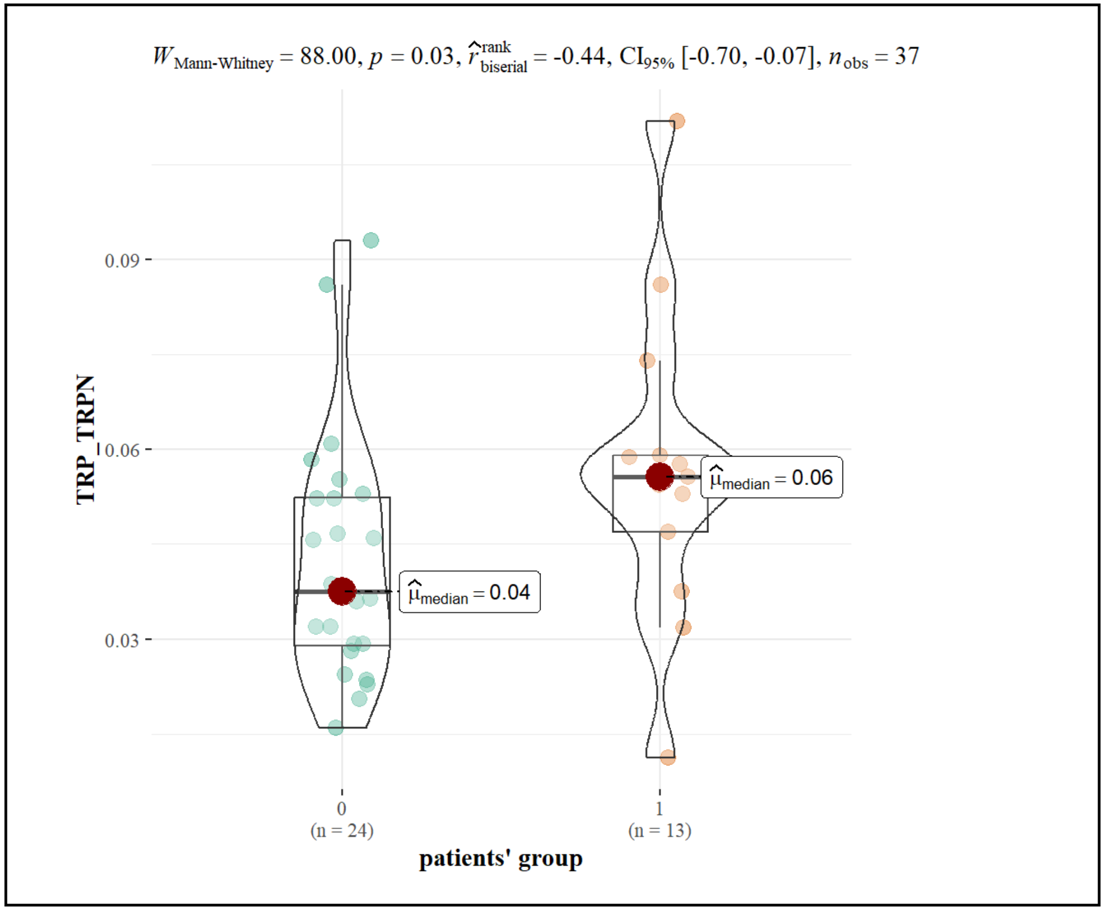

$W_{\text{Mann-Whitney}} = 88.00$, $p = 0.03$, $\widehat{r}_{\text{biserial}}^{\text{rank}} = -0.44$, $\text{CI}_{95\%}$ [-0.70, -0.07], $n_{\text{obs}} = 37$
TRP_TRPN
0.09
0.06
0.03
$\widehat{\mu}_{\text{median}} = 0.04$
$\widehat{\mu}_{\text{median}} = 0.06$
0
(n = 24)
1
(n = 13)
patients' group

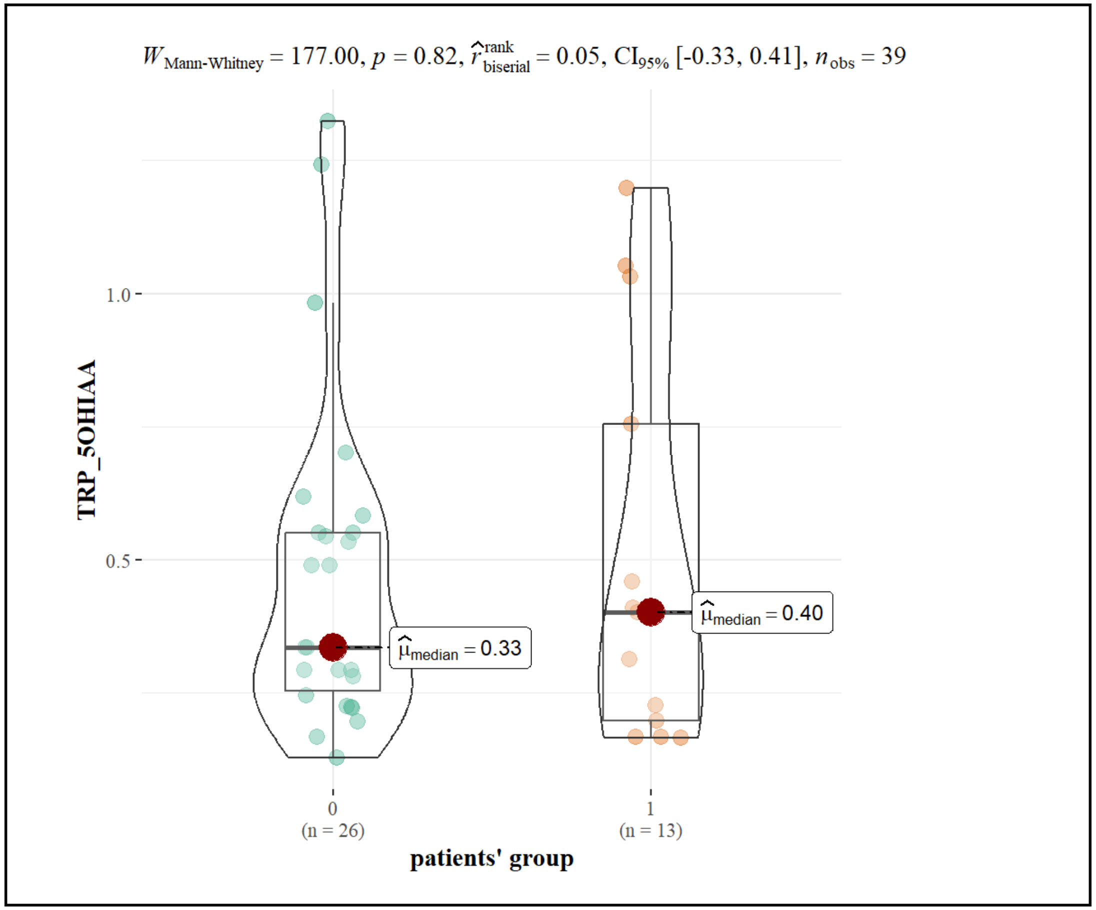

$W_{\text{Mann-Whitney}} = 177.00$, $p = 0.82$, $\hat{r}^{\text{rank}}_{\text{biserial}} = 0.05$, $\text{CI}_{95\%}$ [-0.33, 0.41], $n_{\text{obs}} = 39$
TRP_5OHIAA
1.0
0.5
$\hat{\mu}_{\text{median}} = 0.33$
$\hat{\mu}_{\text{median}} = 0.40$
0
(n = 26)
1
(n = 13)
patients' group

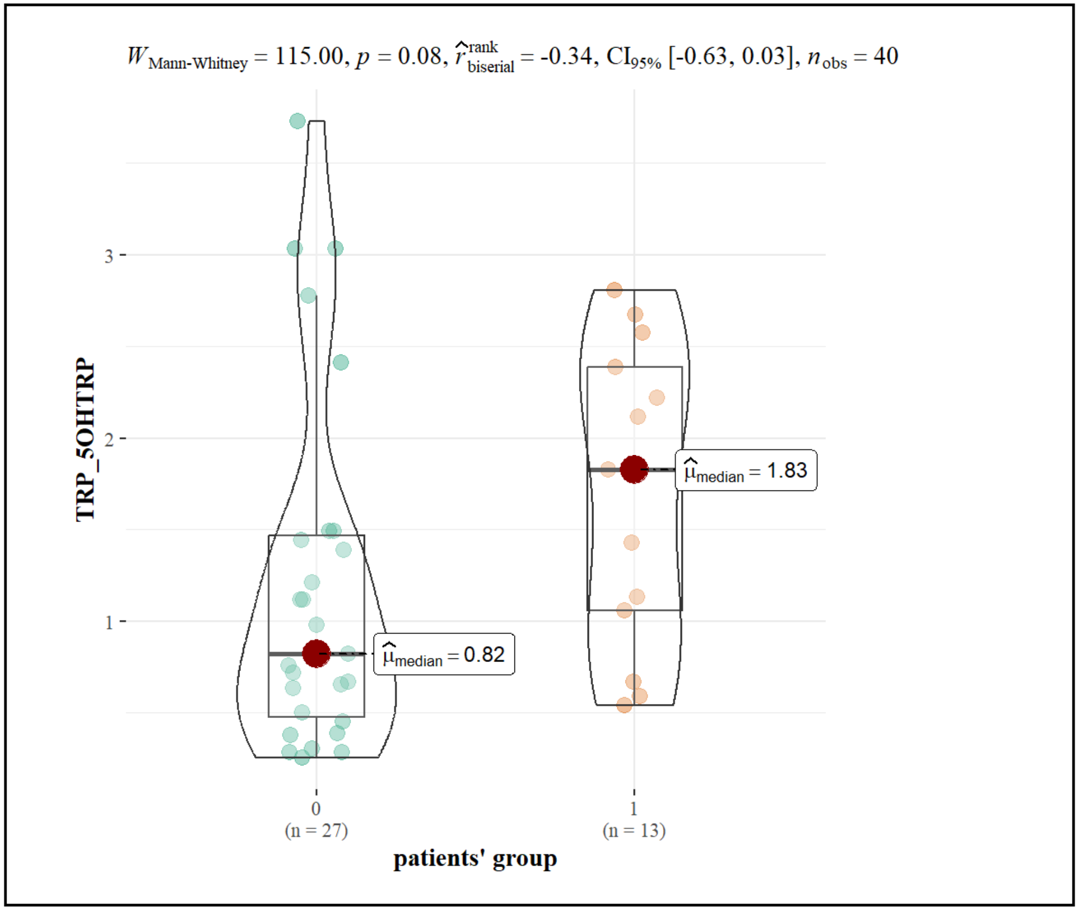

$W_{\text{Mann-Whitney}} = 115.00$, $p = 0.08$, $\hat{r}_{\text{biserial}}^{\text{rank}} = -0.34$, $\text{CI}_{95\%}$ [-0.63, 0.03], $n_{\text{obs}} = 40$
TRP_5OHTRP
3
2
1
$\hat{\mu}_{\text{median}} = 0.82$
$\hat{\mu}_{\text{median}} = 1.83$
0
(n = 27)
1
(n = 13)
patients' group

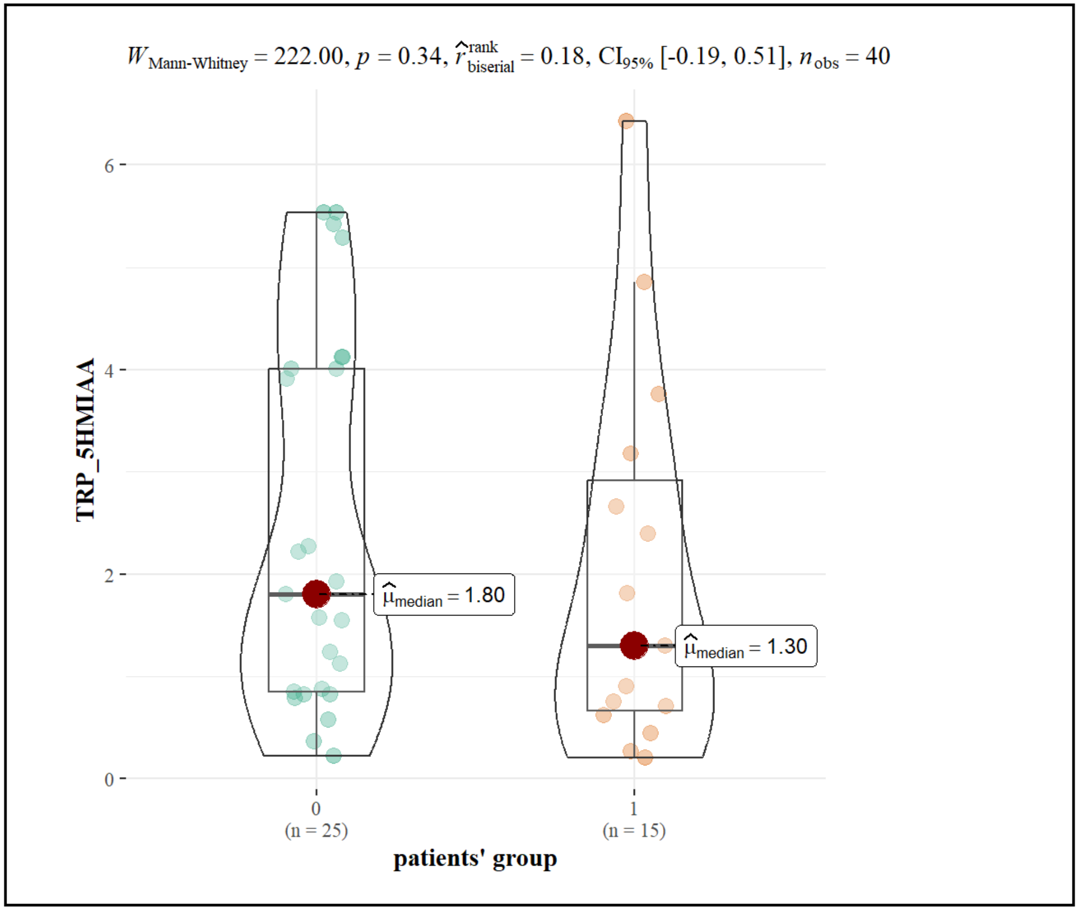

$W_{\text{Mann-Whitney}} = 222.00$, $p = 0.34$, $\hat{r}_{\text{biserial}}^{\text{rank}} = 0.18$, $\text{CI}_{95\%}$ [-0.19, 0.51], $n_{\text{obs}} = 40$
TRP_5HMIAA
$\hat{\mu}_{\text{median}} = 1.80$
$\hat{\mu}_{\text{median}} = 1.30$
0
(n = 25)
1
(n = 15)
patients' group

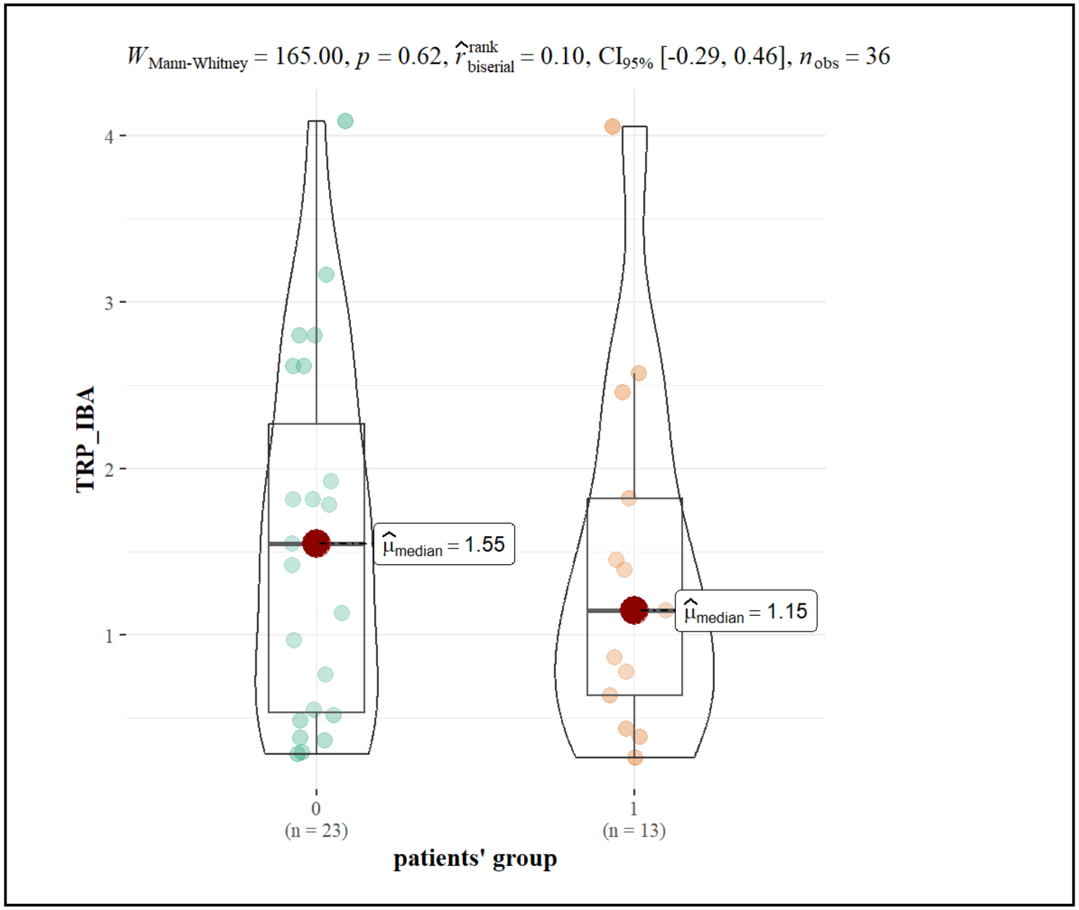

$W_{\text{Mann-Whitney}} = 165.00$, $p = 0.62$, $\hat{r}^{\text{rank}}_{\text{biserial}} = 0.10$, $\text{CI}_{95\%}$ [-0.29, 0.46], $n_{\text{obs}} = 36$
TRP_IBA
4
3
2
1
$\hat{\mu}_{\text{median}} = 1.55$
$\hat{\mu}_{\text{median}} = 1.15$
0
(n = 23)
1
(n = 13)
patients' group

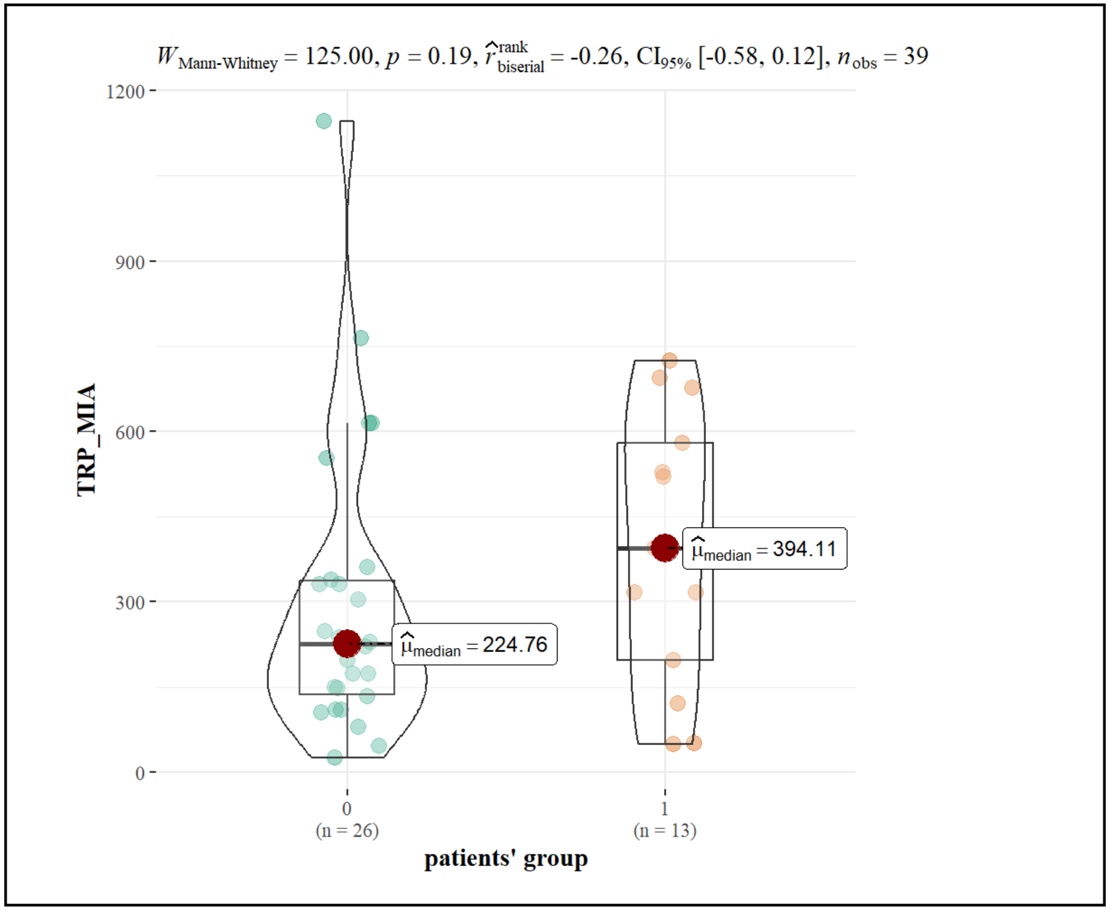
$W_{\text{Mann-Whitney}} = 125.00$, $p = 0.19$, $\hat{r}_{\text{biserial}}^{\text{rank}} = -0.26$, $\text{CI}_{95\%}$ [-0.58, 0.12], $n_{\text{obs}} = 39$
1200
900
600
300
0
TRP_MIA
$\hat{\mu}_{\text{median}} = 224.76$
$\hat{\mu}_{\text{median}} = 394.11$
0
(n = 26)
1
(n = 13)
patients' group

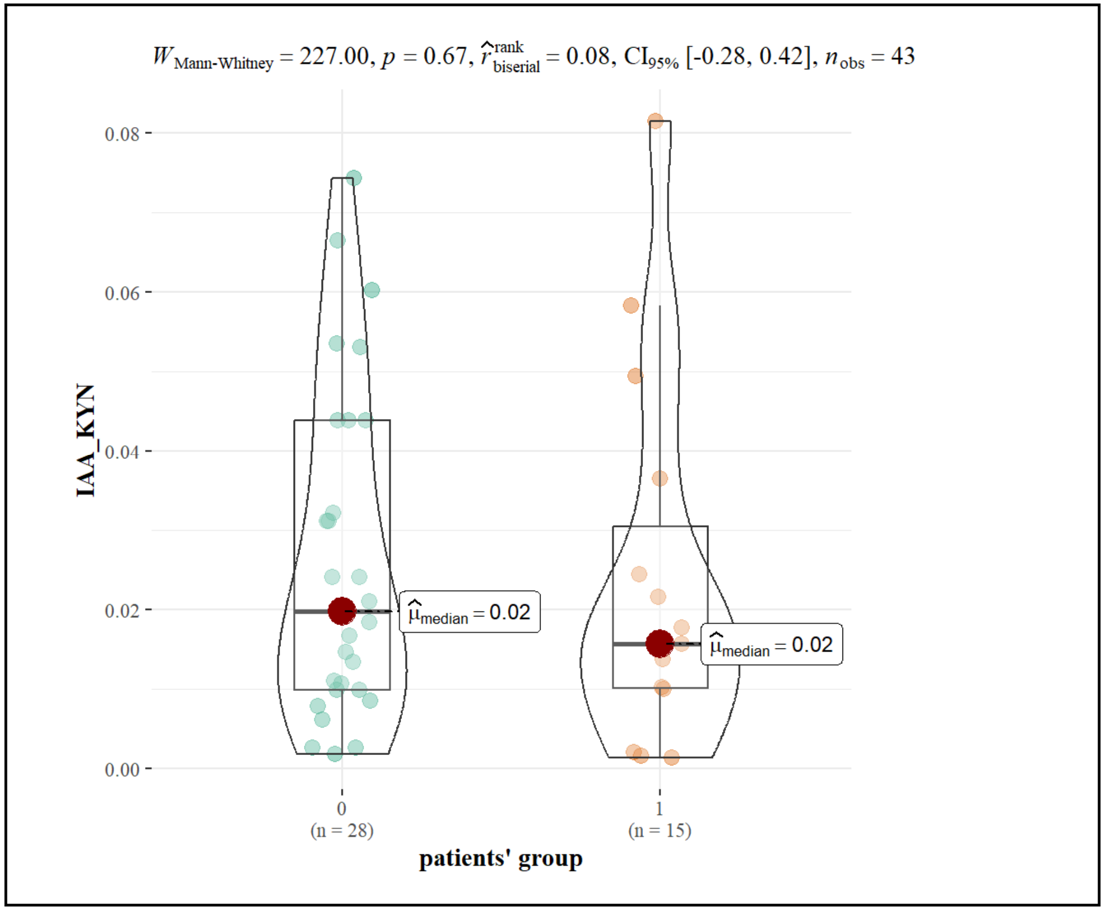
$W_{\text{Mann-Whitney}} = 227.00$, $p = 0.67$, $\hat{r}_{\text{biserial}}^{\text{rank}} = 0.08$, $\text{CI}_{95\%}$ [-0.28, 0.42], $n_{\text{obs}} = 43$
0.08
0.06
0.04
0.02
0.00
IAA_KYN
$\hat{\mu}_{\text{median}} = 0.02$
$\hat{\mu}_{\text{median}} = 0.02$
0
(n = 28)
1
(n = 15)
patients' group

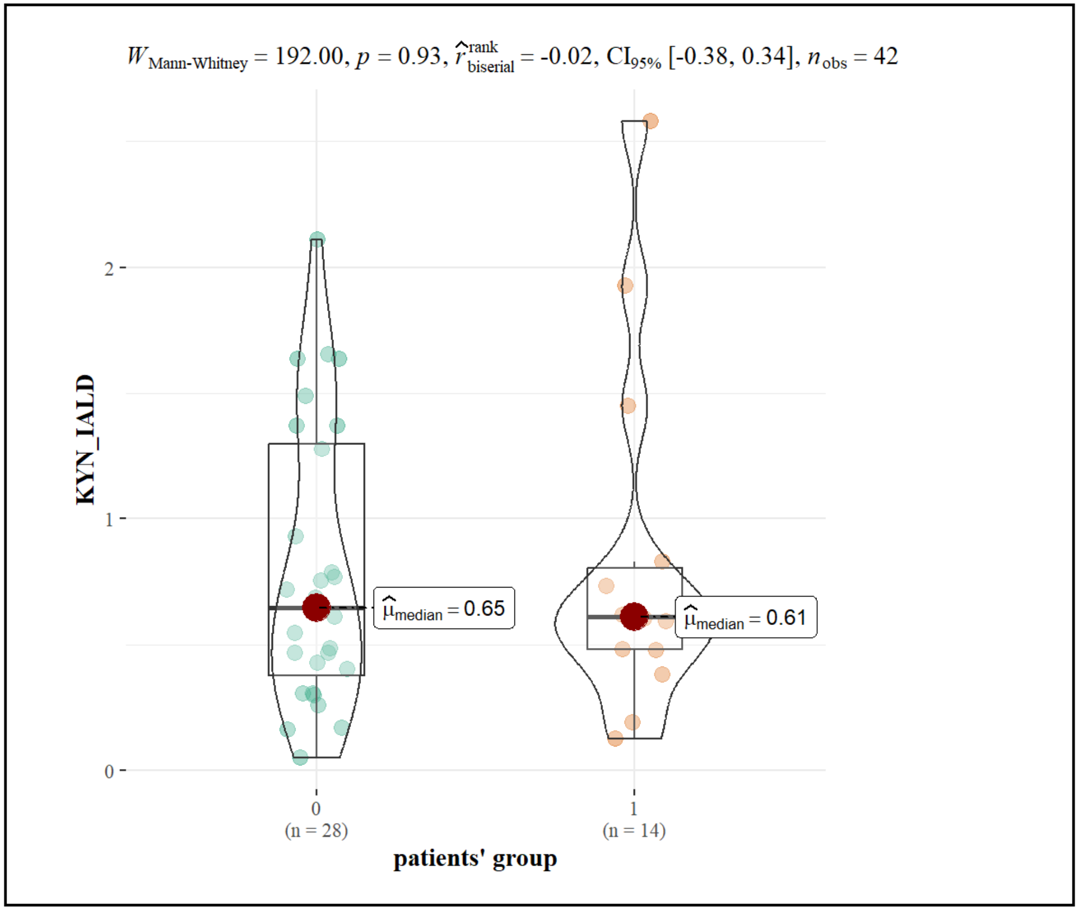

$W_{\text{Mann-Whitney}} = 192.00$, $p = 0.93$, $\hat{r}_{\text{biserial}}^{\text{rank}} = -0.02$, $\text{CI}_{95\%}$ [-0.38, 0.34], $n_{\text{obs}} = 42$
KYN_IALD
2
1
0
$\hat{\mu}_{\text{median}} = 0.65$
$\hat{\mu}_{\text{median}} = 0.61$
0
(n = 28)
1
(n = 14)
patients' group

3 – 4 ASSOCIATION

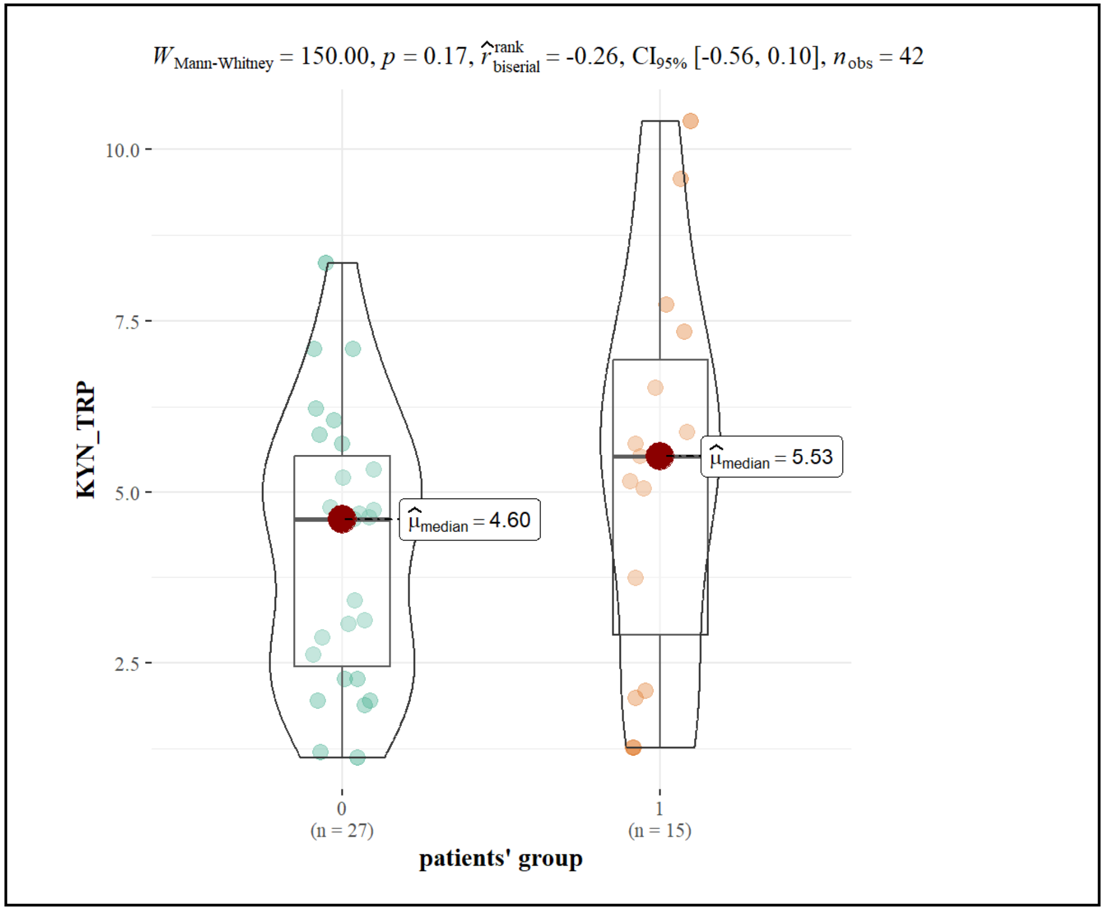

$W_{Mann-Whitney} = 150.00$, $p = 0.17$, $\hat{r}^{rank}_{biserial} = -0.26$, $CI_{95\%}$ [-0.56, 0.10], $n_{obs} = 42$
10.0
7.5
5.0
2.5
KYN_TRP
$\hat{\mu}_{median} = 4.60$
$\hat{\mu}_{median} = 5.53$
0
(n = 27)
1
(n = 15)
patients' group

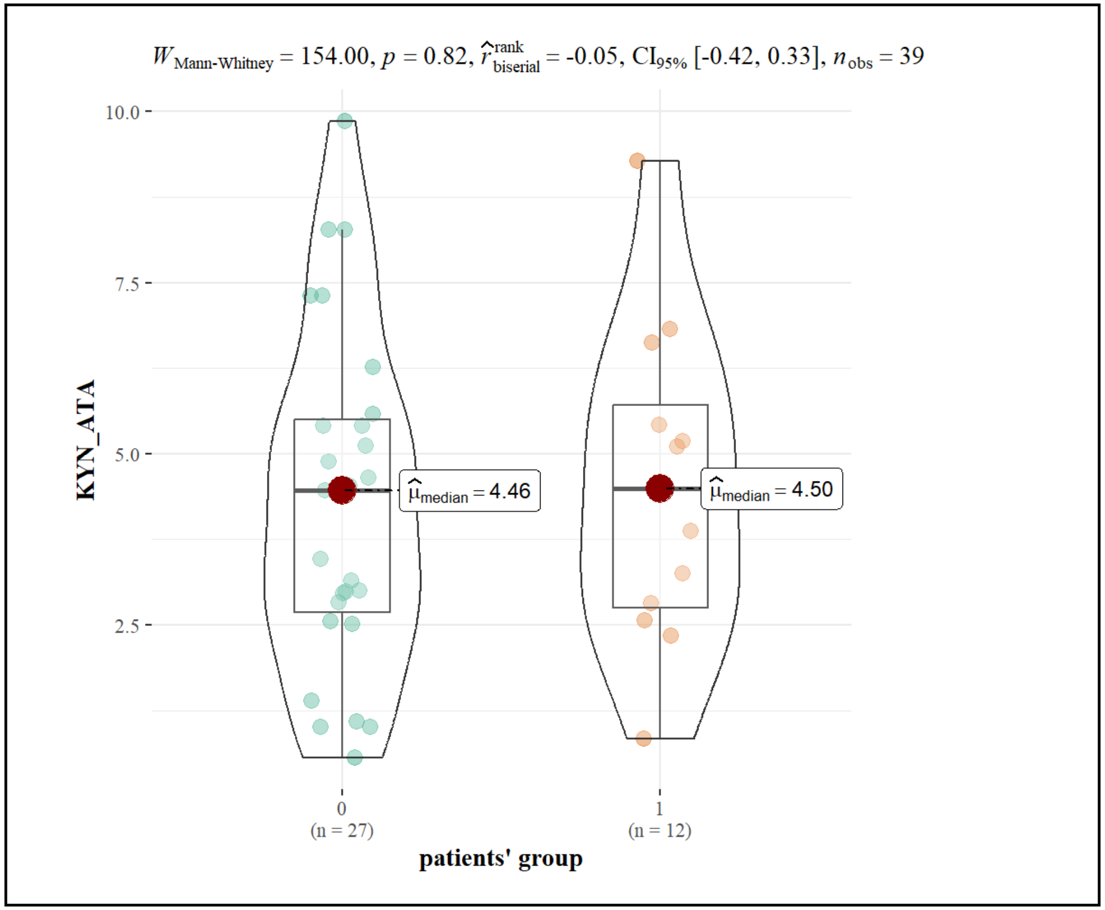
$W_{\text{Mann-Whitney}} = 154.00$, $p = 0.82$, $\hat{r}_{\text{biserial}}^{\text{rank}} = -0.05$, $\text{CI}_{95\%}$ [-0.42, 0.33], $n_{\text{obs}} = 39$
10.0
7.5
5.0
2.5
KYN_ATA
$\hat{\mu}_{\text{median}} = 4.46$
$\hat{\mu}_{\text{median}} = 4.50$
0
(n = 27)
1
(n = 12)
patients' group

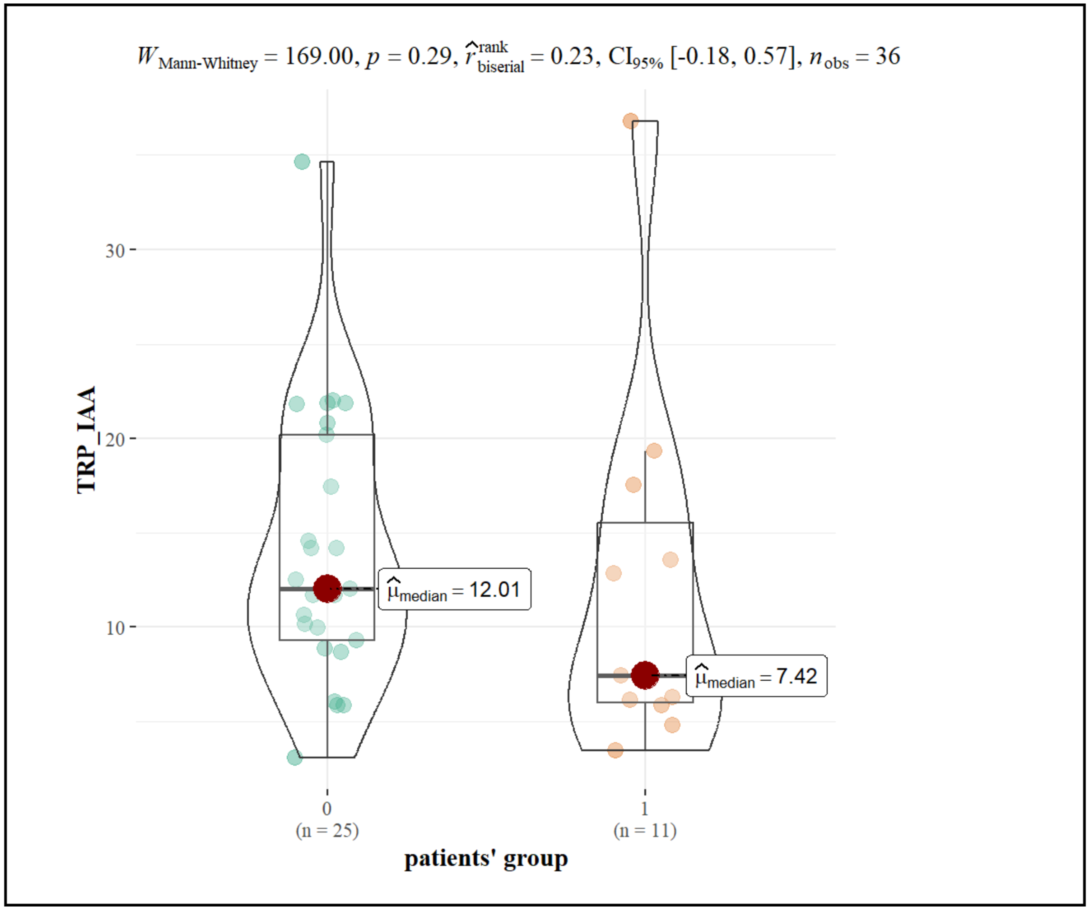

$W_{\text{Mann-Whitney}} = 169.00$, $p = 0.29$, $\hat{r}_{\text{biserial}}^{\text{rank}} = 0.23$, $\text{CI}_{95\%}$ [-0.18, 0.57], $n_{\text{obs}} = 36$
TRP_IAA
30
20
10
$\hat{\mu}_{\text{median}} = 12.01$
$\hat{\mu}_{\text{median}} = 7.42$
0
(n = 25)
1
(n = 11)
patients' group

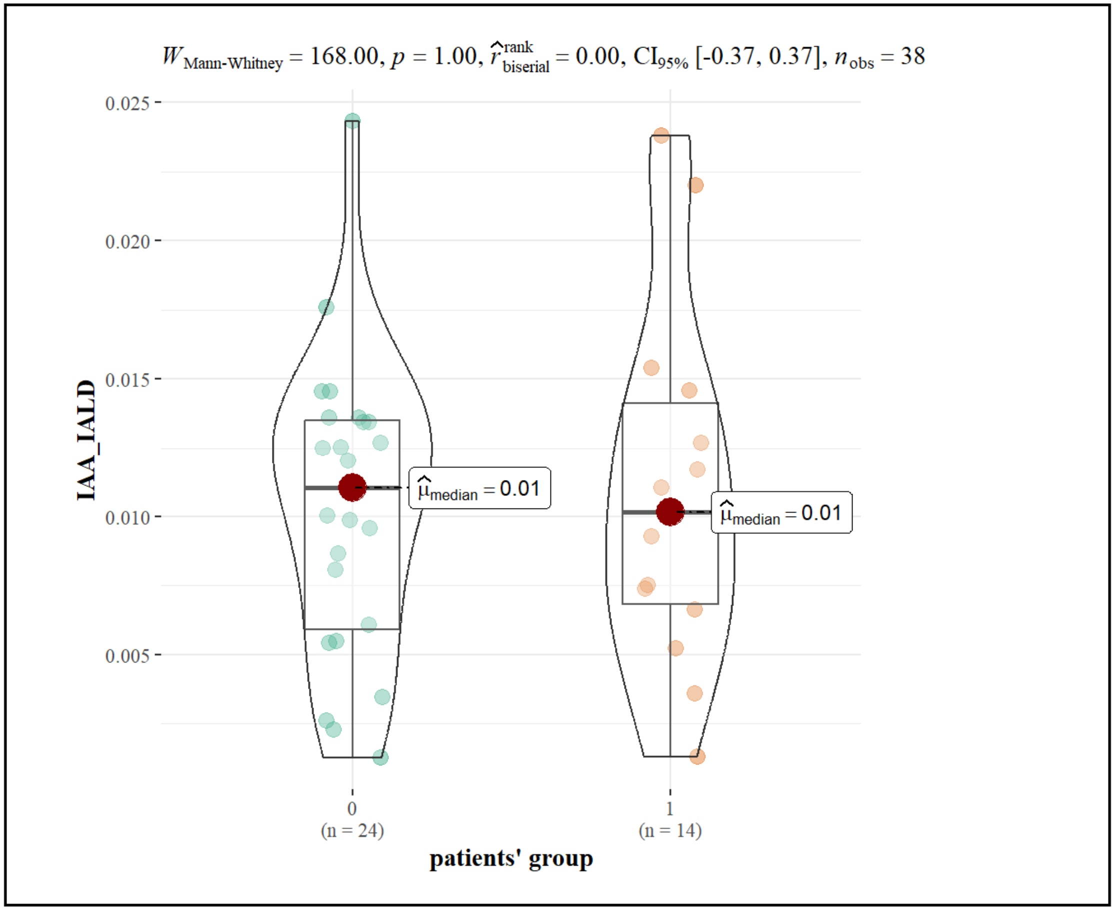

$W_{\text{Mann-Whitney}} = 168.00$, $p = 1.00$, $\hat{r}^{\text{rank}}_{\text{biserial}} = 0.00$, $\text{CI}_{95\%}$ [-0.37, 0.37], $n_{\text{obs}} = 38$
0.025
0.020
0.015
0.010
0.005
IAA_IALD
$\hat{\mu}_{\text{median}} = 0.01$
$\hat{\mu}_{\text{median}} = 0.01$
0
(n = 24)
1
(n = 14)
patients' group

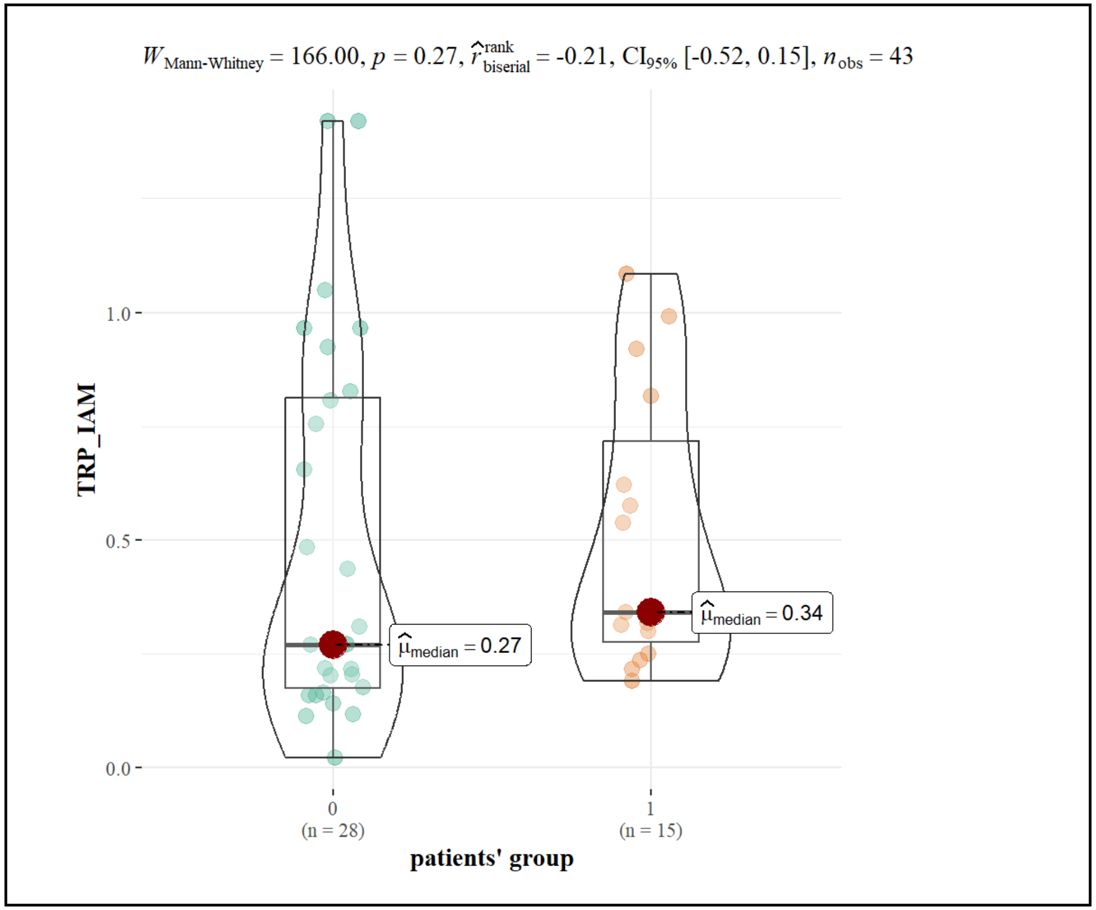
$W_{\text{Mann-Whitney}} = 166.00, p = 0.27, \hat{r}_{\text{biserial}}^{\text{rank}} = -0.21, \text{CI}_{95\%} [-0.52, 0.15], n_{\text{obs}} = 43$
TRP_IAM
1.0
0.5
0.0
$\hat{\mu}_{\text{median}} = 0.27$
$\hat{\mu}_{\text{median}} = 0.34$
0
(n = 28)
1
(n = 15)
patients' group

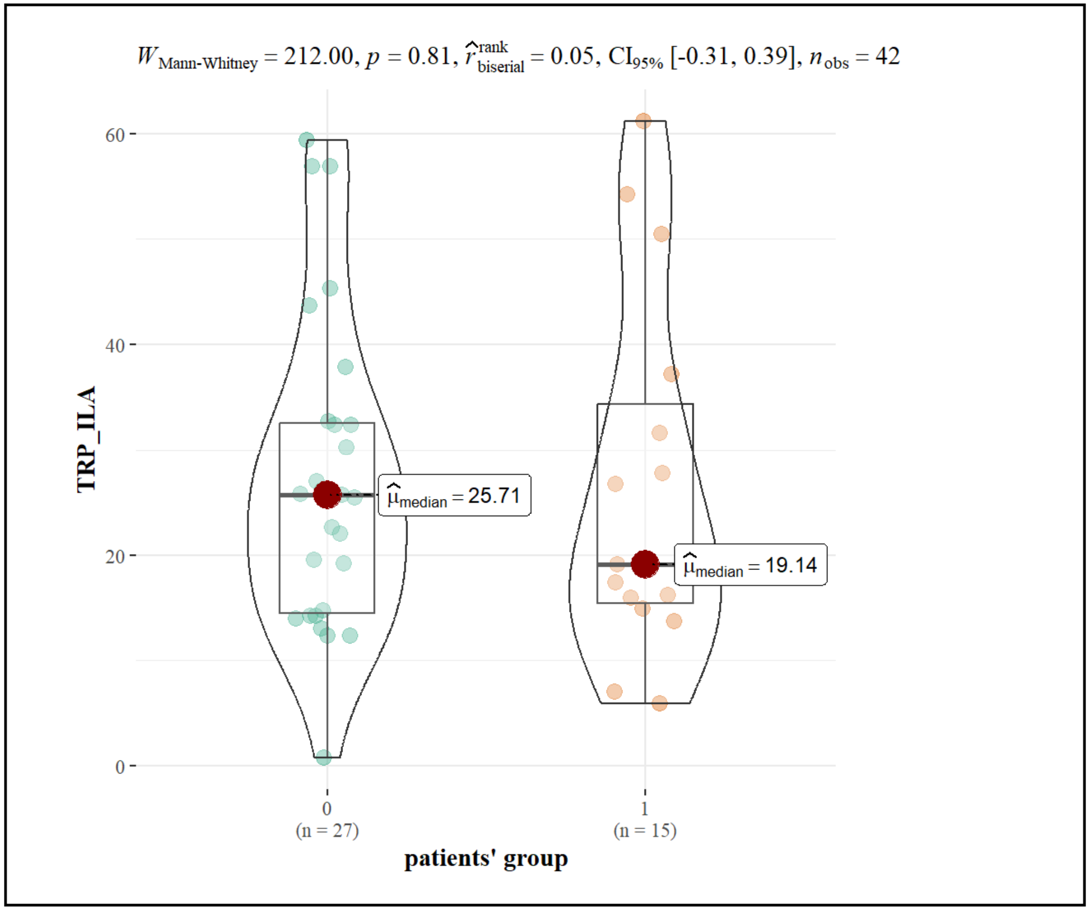

$W_{\text{Mann-Whitney}} = 212.00$, $p = 0.81$, $\widehat{r}_{\text{biserial}}^{\text{rank}} = 0.05$, $\text{CI}_{95\%}$ [-0.31, 0.39], $n_{\text{obs}} = 42$
60
40
20
0
TRP_ILA
$\widehat{\mu}_{\text{median}} = 25.71$
$\widehat{\mu}_{\text{median}} = 19.14$
0
(n = 27)
1
(n = 15)
patients' group

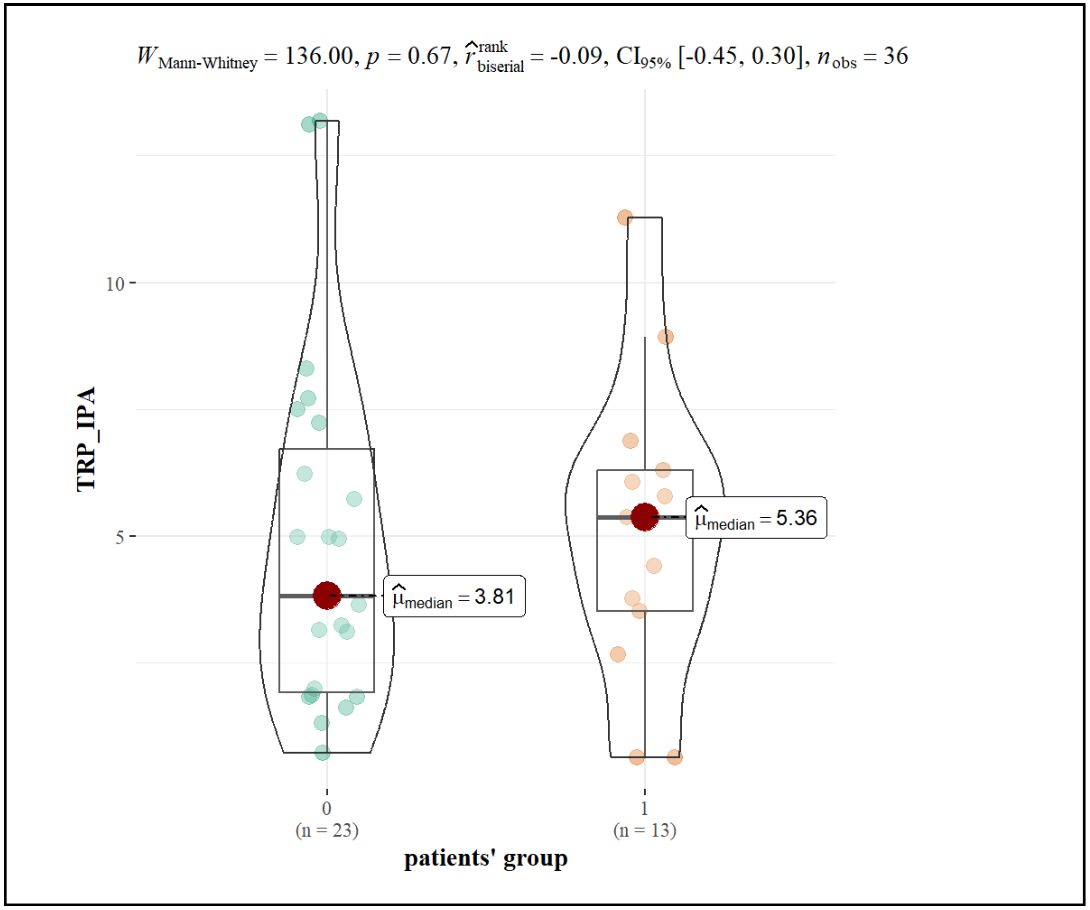

$W_{\text{Mann-Whitney}} = 136.00$, $p = 0.67$, $\hat{r}_{\text{biserial}}^{\text{rank}} = -0.09$, $\text{CI}_{95\%}$ [-0.45, 0.30], $n_{\text{obs}} = 36$
TRP_IPA
10
5
$\hat{\mu}_{\text{median}} = 3.81$
$\hat{\mu}_{\text{median}} = 5.36$
0
(n = 23)
1
(n = 13)
patients' group

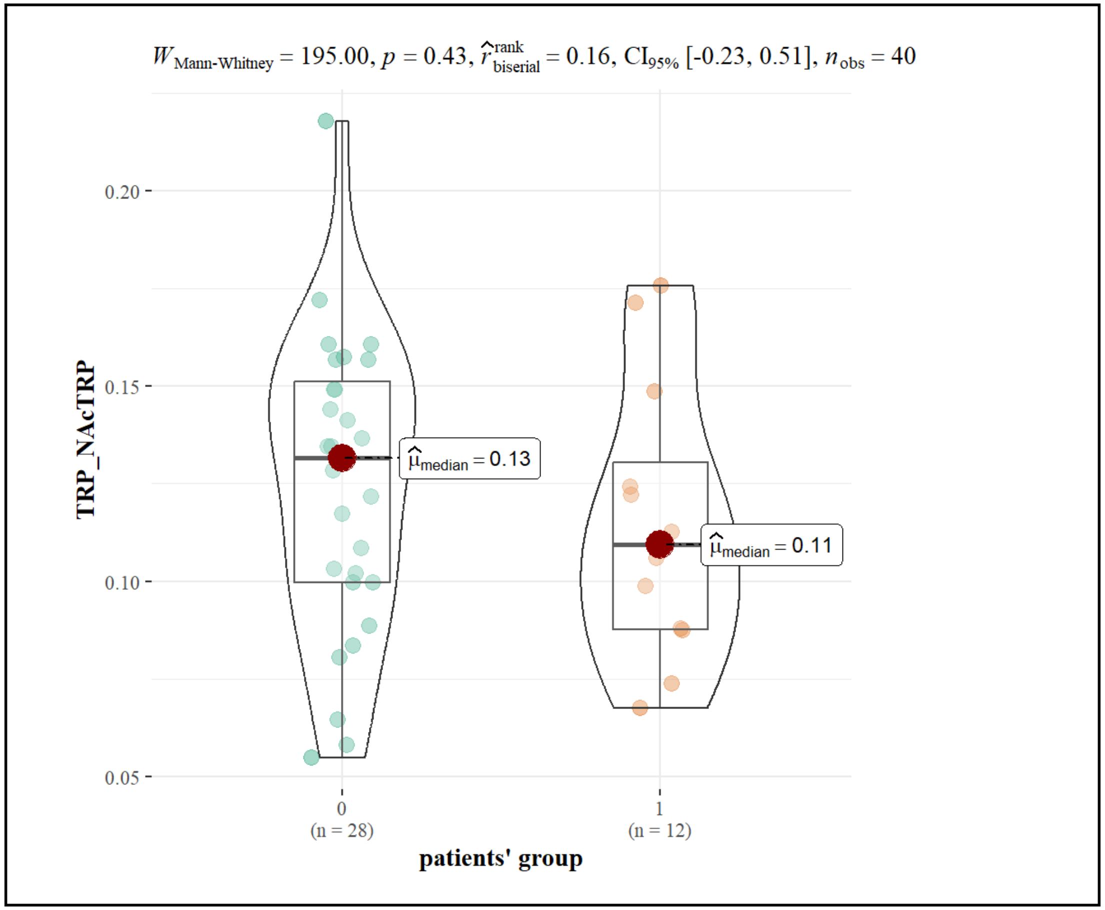

$W_{\text{Mann-Whitney}} = 195.00$, $p = 0.43$, $\hat{r}^{\text{rank}}_{\text{biserial}} = 0.16$, $\text{CI}_{95\%}$ [-0.23, 0.51], $n_{\text{obs}} = 40$
TRP_NAcTRP
0.20
0.15
0.10
0.05
$\hat{\mu}_{\text{median}} = 0.13$
$\hat{\mu}_{\text{median}} = 0.11$
0
(n = 28)
1
(n = 12)
patients' group

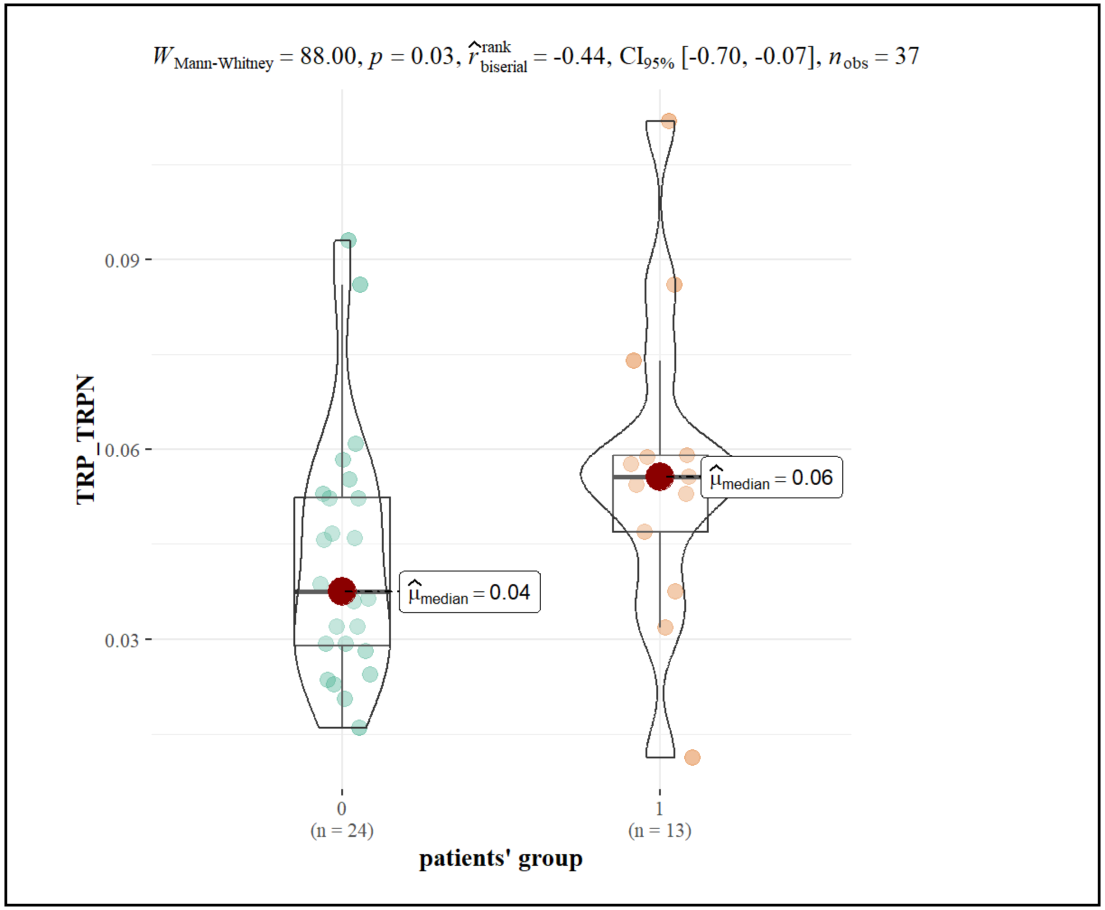

$W_{\text{Mann-Whitney}} = 88.00$, $p = 0.03$, $\hat{r}^{\text{rank}}_{\text{biserial}} = -0.44$, $\text{CI}_{95\%}$ [-0.70, -0.07], $n_{\text{obs}} = 37$
TRP_TRPN
0.09
0.06
0.03
$\hat{\mu}_{\text{median}} = 0.04$
$\hat{\mu}_{\text{median}} = 0.06$
0
(n = 24)
1
(n = 13)
patients' group

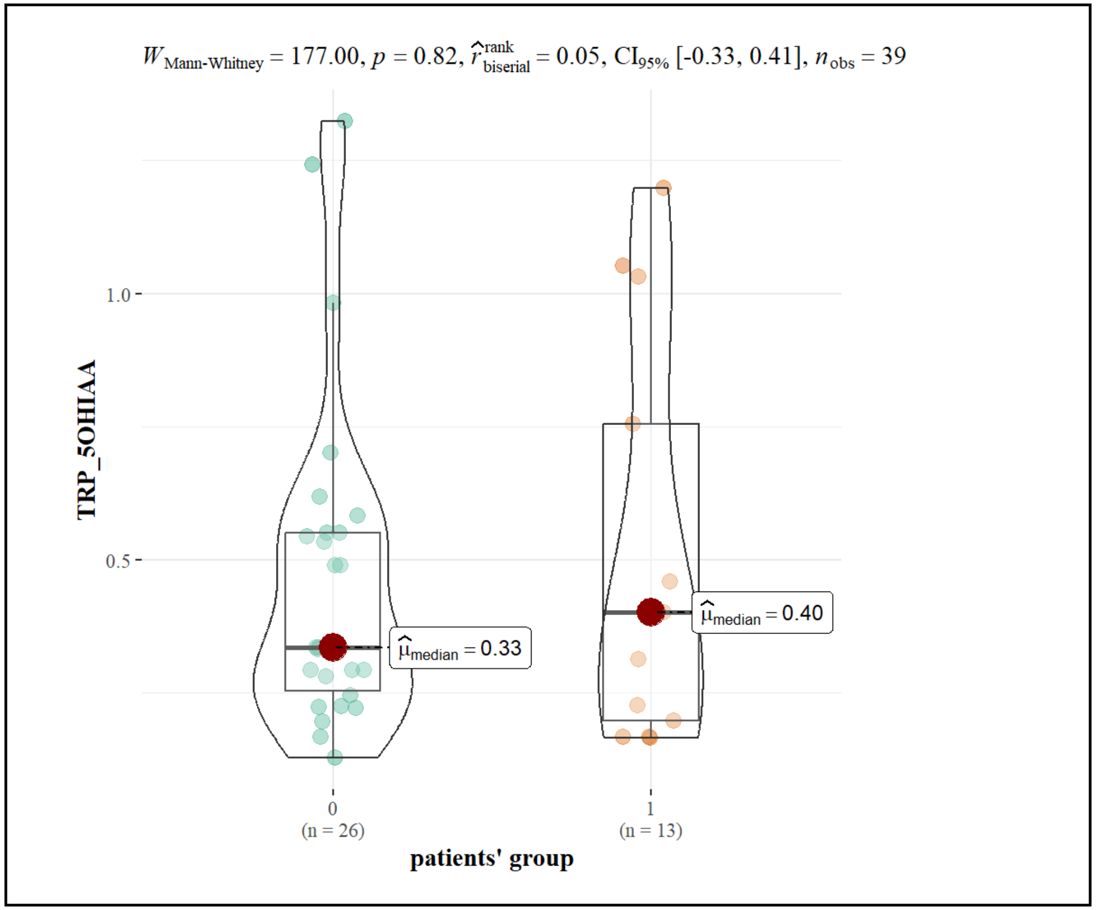

$W_{Mann-Whitney} = 177.00$, $p = 0.82$, $\hat{r}^{rank}_{biserial} = 0.05$, $CI_{95\%}$ [-0.33, 0.41], $n_{obs} = 39$
1.0
0.5
TRP_5OHIAA
$\hat{\mu}_{median} = 0.33$
$\hat{\mu}_{median} = 0.40$
0
(n = 26)
1
(n = 13)
patients' group

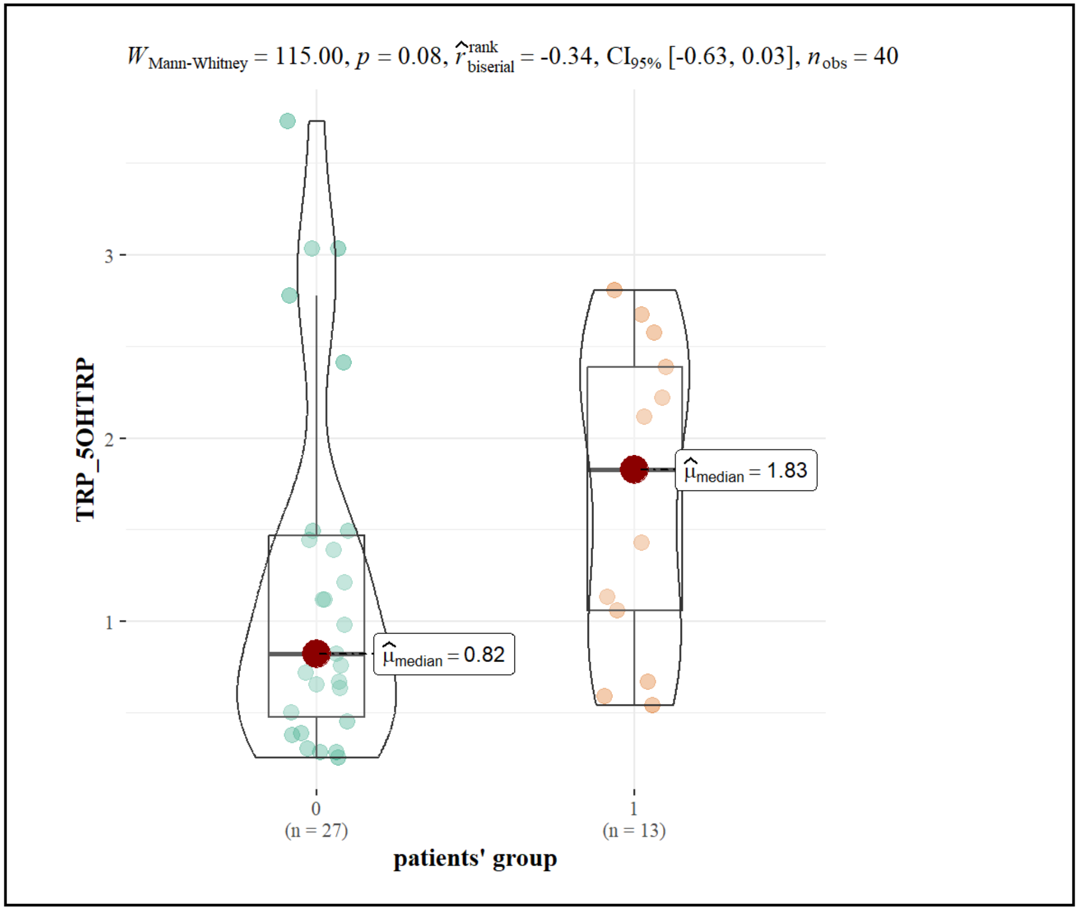

$W_{\text{Mann-Whitney}} = 115.00$, $p = 0.08$, $\hat{r}_{\text{biserial}}^{\text{rank}} = -0.34$, $\text{CI}_{95\%}$ [-0.63, 0.03], $n_{\text{obs}} = 40$
TRP_5OHTRP
3
2
1
$\hat{\mu}_{\text{median}} = 0.82$
$\hat{\mu}_{\text{median}} = 1.83$
0
(n = 27)
1
(n = 13)
patients' group

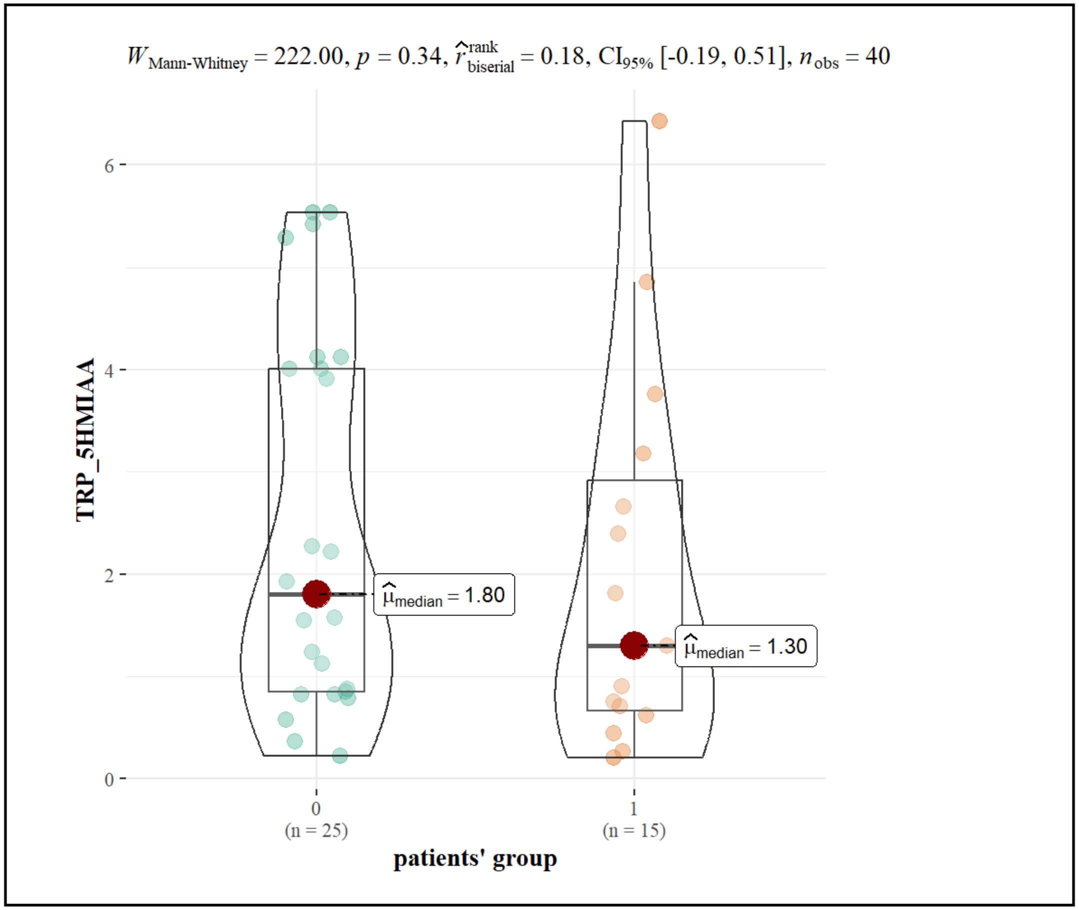

$W_{\text{Mann-Whitney}} = 222.00$, $p = 0.34$, $\hat{r}^{\text{rank}}_{\text{biserial}} = 0.18$, $\text{CI}_{95\%}$ [-0.19, 0.51], $n_{\text{obs}} = 40$
TRP_5HMIAA
6
4
2
0
$\hat{\mu}_{\text{median}} = 1.80$
$\hat{\mu}_{\text{median}} = 1.30$
0
(n = 25)
1
(n = 15)
patients' group

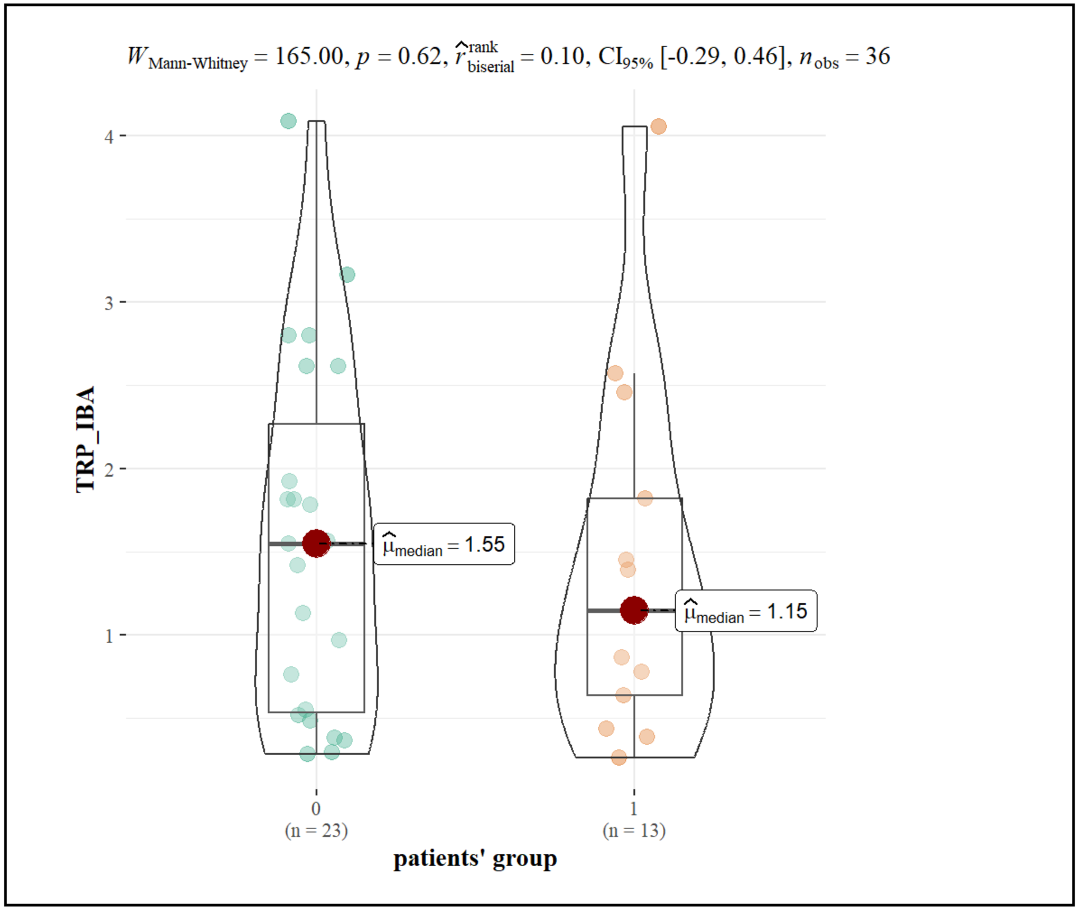
$W_{\text{Mann-Whitney}} = 165.00$, $p = 0.62$, $\hat{r}_{\text{biserial}}^{\text{rank}} = 0.10$, $\text{CI}_{95\%}$ [-0.29, 0.46], $n_{\text{obs}} = 36$
TRP_IBA
4
3
2
1
$\hat{\mu}_{\text{median}} = 1.55$
$\hat{\mu}_{\text{median}} = 1.15$
0
(n = 23)
1
(n = 13)
patients' group

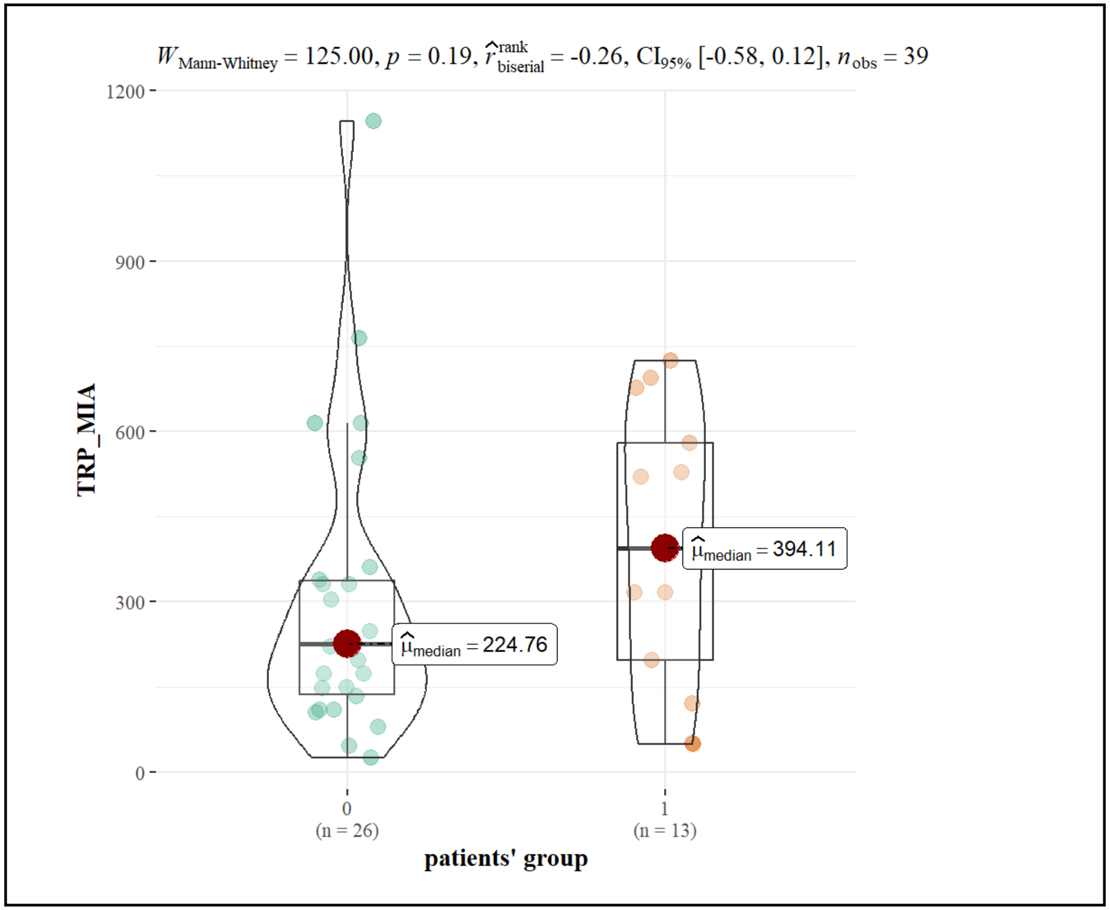
$W_{\text{Mann-Whitney}}$ = 125.00, p = 0.19, $\hat{r}^{\text{rank}}_{\text{biserial}}$ = -0.26, $\text{CI}_{95\%}$ [-0.58, 0.12], n_{obs} = 39
1200
900
600
300
0
TRP_MIA
$\hat{\mu}_{\text{median}}$ = 224.76
$\hat{\mu}_{\text{median}}$ = 394.11
0
(n = 26)
1
(n = 13)
patients' group

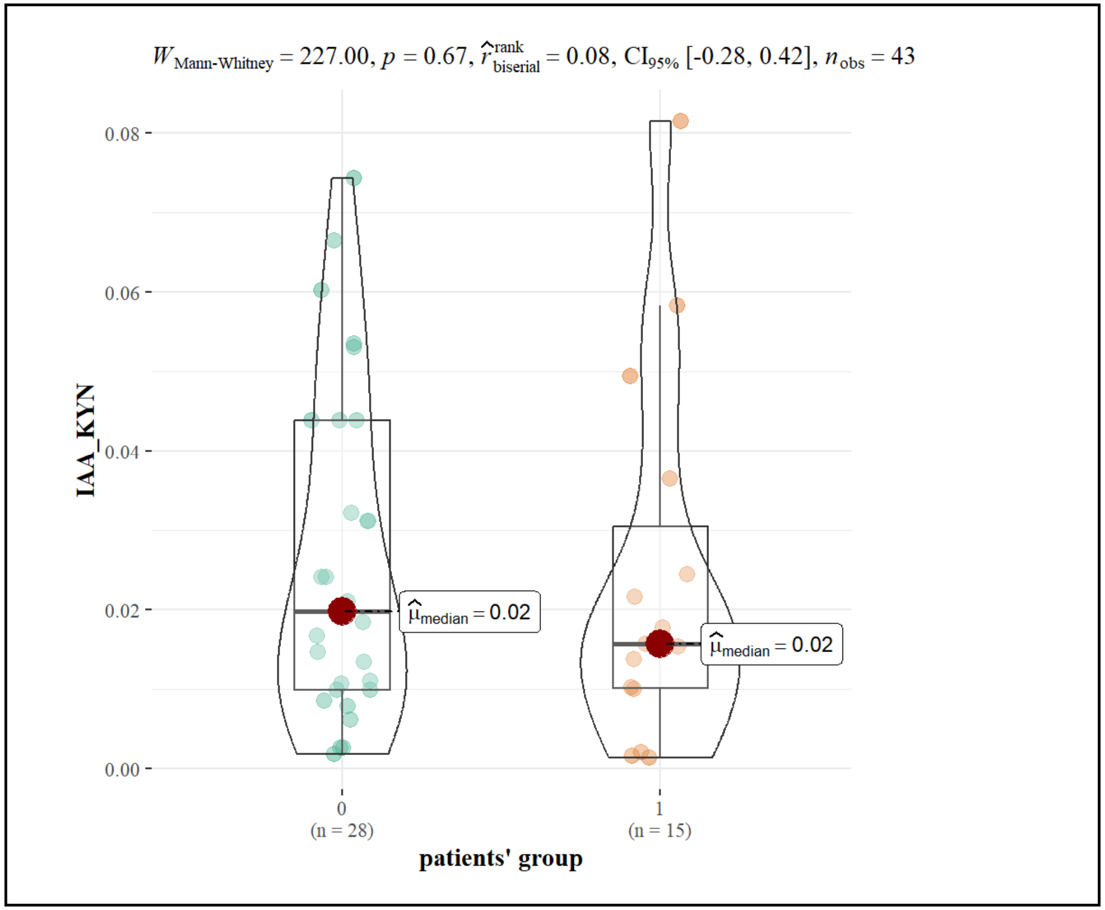

$W_{\text{Mann-Whitney}} = 227.00$, $p = 0.67$, $\widehat{r}_{\text{biserial}}^{\text{rank}} = 0.08$, $\text{CI}_{95\%}$ [-0.28, 0.42], $n_{\text{obs}} = 43$
IAA_KYN
0.08
0.06
0.04
0.02
0.00
$\widehat{\mu}_{\text{median}} = 0.02$
$\widehat{\mu}_{\text{median}} = 0.02$
0
(n = 28)
1
(n = 15)
patients' group

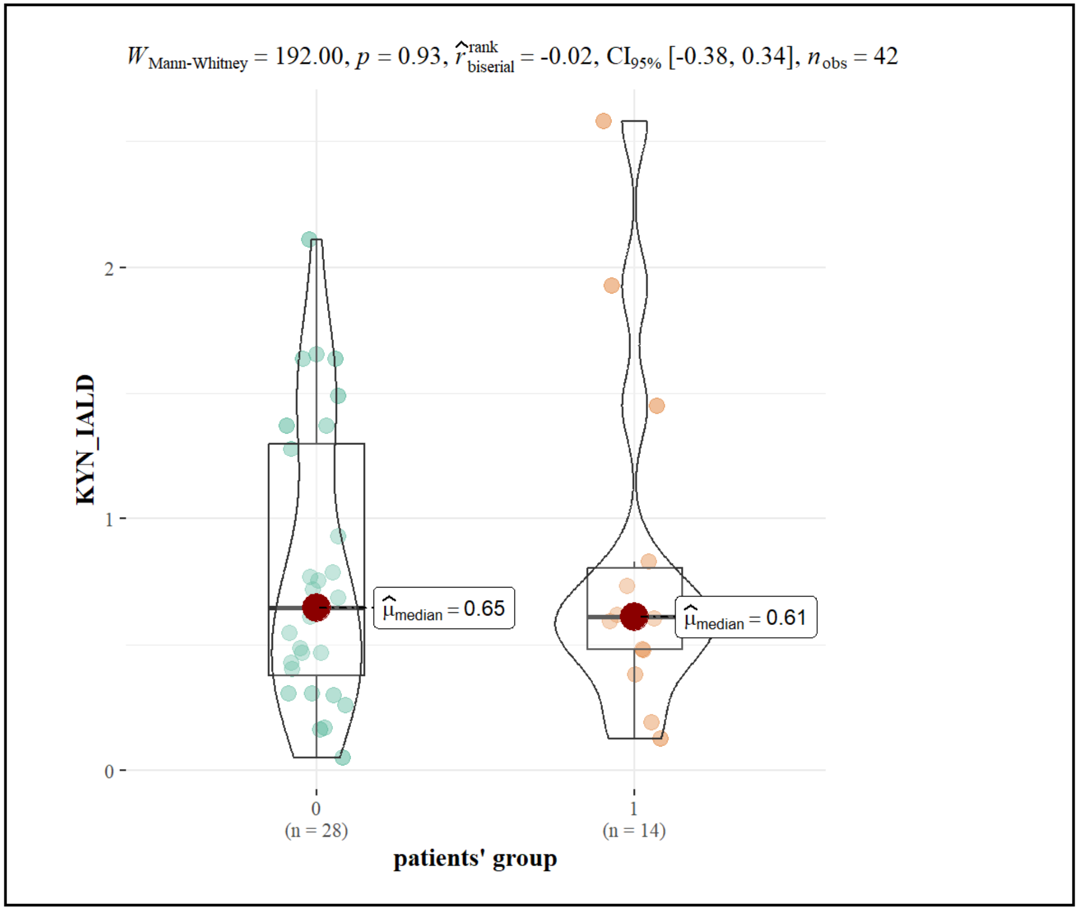

$W_{\text{Mann-Whitney}} = 192.00$, $p = 0.93$, $\hat{r}_{\text{biserial}}^{\text{rank}} = -0.02$, $\text{CI}_{95\%}$ [-0.38, 0.34], $n_{\text{obs}} = 42$
KYN_IALD
2
1
0
$\hat{\mu}_{\text{median}} = 0.65$
$\hat{\mu}_{\text{median}} = 0.61$
0
(n = 28)
1
(n = 14)
patients' group

Table S3: Tryptophan metabolites in children with moderate and severe ASD and their siblings; all values are in nM/mmol of creatinine

Metabolite	Sibling (N=28)	Moderate (N=28)	p value	Sibling (N=16)	Severe (N=16)	p value
TRP			0.04			0.57
Mean (SD)	17.17 (7.60)	22.62 (9.63)		19.95 (9.07)	21.74 (6.52)	
Median (Q1, Q3)	16.13 (13.00, 19.80)	19.32 (15.73, 31.79)		21.38 (12.16, 26.52)	21.37 (19.29, 25.67)	
Min - Max	6.59 - 38.83	9.07 - 39.65		6.03 - 34.94	7.38 - 31.30	
ATA			0.95			0.91
Mean (SD)	23.15 (10.08)	23.61 (11.68)		17.66 (7.17)	16.46 (7.23)	
Median (Q1, Q3)	22.39 (17.72, 26.50)	21.47 (14.26, 32.64)		15.43 (12.09, 22.57)	16.88 (10.21, 21.09)	
Min - Max	5.52 - 50.67	5.04 - 50.73		9.44 - 31.09	4.52 - 29.51	
IAA			0.54			0.71
Mean (SD)	1.85 (1.59)	2.02 (1.50)		1.67 (1.31)	1.57 (1.07)	
Median (Q1, Q3)	1.32 (0.83, 2.08)	1.68 (0.94, 2.85)		1.52 (0.42, 2.83)	1.18 (0.88, 2.37)	
Min - Max	0.11 - 5.84	0.20 - 5.80		0.19 - 3.93	0.22 - 3.53	
IALD			0.66			0.09
Mean (SD)	186.89 (110.21)	172.01 (88.14)		225.31 (135.07)	152.50 (76.64)	
Median (Q1, Q3)	173.05 (102.97, 265.91)	147.13 (111.80, 226.26)		251.39 (104.43, 319.38)	134.39 (91.37, 188.69)	
Min - Max	17.43 - 449.03	30.44 - 358.36		39.59 - 455.15	62.03 - 313.85	
IAM			0.98			0.66
Mean (SD)	61.99 (37.17)	65.64 (41.31)		61.37 (38.55)	54.88 (26.84)	
Median (Q1, Q3)	54.04 (34.55, 76.61)	57.14 (32.71, 85.72)		62.11 (26.57, 80.24)	56.01 (29.36, 77.79)	

Min - Max	15.84 - 160.04	18.11 - 160.99		10.43 - 132.42	12.85 - 100.63	
IBA			0.82			0.62
Mean (SD)	21.35 (19.70)	22.91 (20.66)		17.68 (11.84)	15.77 (13.97)	
Median (Q1, Q3)	17.22 (8.05, 24.24)	12.97 (8.11, 39.90)		17.07 (9.99, 19.99)	12.21 (8.17, 18.71)	
Min - Max	1.04 - 75.60	0.03 - 68.17		0.81 - 39.56	0.40 - 44.33	
ILA			0.49			0.94
Mean (SD)	1.15 (0.62)	1.01 (0.62)		0.93 (0.49)	0.94 (0.51)	
Median (Q1, Q3)	1.02 (0.64, 1.64)	0.74 (0.67, 1.08)		0.82 (0.61, 1.26)	0.83 (0.52, 1.40)	
Min - Max	0.30 - 2.41	0.28 - 2.67		0.18 - 1.97	0.27 - 1.80	
IPA			0.51			0.76
Mean (SD)	5.59 (3.29)	5.12 (4.76)		3.39 (1.68)	3.62 (1.40)	
Median (Q1, Q3)	5.09 (3.59, 7.44)	4.13 (1.82, 6.78)		3.61 (2.98, 4.52)	3.16 (2.65, 4.69)	
Min - Max	0.79 - 13.98	0.00 - 19.17		0.20 - 5.72	1.27 - 5.71	
KYN			0.86			0.45
Mean (SD)	79.77 (42.99)	73.59 (38.22)		87.47 (52.40)	113.77 (64.41)	
Median (Q1, Q3)	68.93 (52.14, 106.48)	73.98 (41.73, 99.02)		71.21 (48.16, 123.96)	103.99 (64.82, 139.26)	
Min - Max	28.46 - 211.85	11.62 - 178.18		25.41 - 185.54	29.30 - 270.96	
MIA			0.75			0.94
Mean (SD)	0.08 (0.05)	0.09 (0.07)		0.07 (0.04)	0.07 (0.04)	
Median (Q1, Q3)	0.06 (0.06, 0.10)	0.07 (0.04, 0.12)		0.06 (0.04, 0.10)	0.06 (0.04, 0.07)	
Min - Max	0.01 - 0.21	0.00 - 0.23		0.01 - 0.14	0.00 - 0.14	
NAcTRP			0.46			0.38
Mean (SD)	203.33 (117.72)	212.55 (87.76)		194.52 (109.36)	165.18 (55.57)	

Median (Q1, Q3)	181.26 (96.22, 276.54)	225.92 (159.52, 265.84)		195.26 (93.31, 237.95)	181.75 (112.48, 203.13)	
Min - Max	35.88 - 488.19	56.47 - 344.29		66.14 - 409.24	80.06 - 241.57	
TRPN			0.07			0.45
Mean (SD)	400.99 (281.31)	516.92 (281.85)		432.70 (193.42)	365.38 (173.84)	
Median (Q1, Q3)	385.01 (211.48, 451.06)	573.42 (267.86, 758.15)		436.53 (284.27, 572.56)	391.67 (238.62, 451.97)	
Min - Max	69.53 - 1255.39	63.19 - 1075.56		56.29 - 755.95	125.02 - 653.10	
5OHIAA			0.25			0.90
Mean (SD)	46.57 (24.55)	53.62 (23.03)		56.22 (34.24)	51.35 (36.95)	
Median (Q1, Q3)	39.46 (27.88, 57.57)	48.64 (33.60, 63.86)		40.00 (32.02, 88.16)	43.90 (25.99, 51.32)	
Min - Max	18.23 - 108.52	24.96 - 101.83		17.96 - 116.40	9.74 - 136.33	
5OHTRP			0.82			0.32
Mean (SD)	28.73 (16.04)	31.05 (24.70)		18.22 (10.47)	14.24 (7.92)	
Median (Q1, Q3)	28.14 (17.61, 32.65)	24.00 (13.38, 45.02)		16.98 (10.71, 25.55)	12.46 (9.79, 19.67)	
Min - Max	5.48 - 76.76	3.48 - 85.09		0.71 - 36.72	0.79 - 28.73	
5MIAA			0.34			0.67
Mean (SD)	15.16 (11.02)	12.17 (9.65)		18.90 (14.96)	20.60 (13.60)	
Median (Q1, Q3)	12.77 (6.09, 22.33)	9.62 (7.40, 12.78)		17.15 (12.49, 22.05)	21.68 (8.15, 33.47)	
Min - Max	0.79 - 45.27	1.12 - 38.93		1.83 - 61.28	4.06 - 44.47	
U-Creatinin			0.46			0.71
Mean (SD)	7.70 (3.88)	8.40 (5.06)		7.38 (5.02)	8.29 (4.33)	
Median (Q1, Q3)	6.70 (4.85, 9.85)	7.10 (4.40, 10.65)		6.10 (4.25, 9.60)	8.40 (5.25, 11.00)	
Min - Max	1.60 - 17.50	2.30 - 18.20		0.10 - 18.30	0.70 - 15.90	

Table S4: Tryptophan metabolite ratios in the urine of a group of children with moderate and severe ASD and their siblings

Metabolite ratios	Sibling (N=28)	Moderate (N=28)	p value	Sibling (N=16)	Severe (N=16)	p value
KYN_TRP			0.60			0.48
Mean (SD)	4.45 (1.73)	4.20 (1.93)		4.75 (2.29)	5.39 (2.81)	
Median (Q1, Q3)	4.03 (3.30, 5.27)	4.60 (2.53, 5.42)		4.37 (3.43, 5.55)	5.53 (3.57, 6.93)	
Min - Max	1.41 - 9.03	1.20 - 8.34		0.82 - 9.84	1.27 - 10.41	
KYN_ATA			0.37			0.15
Mean (SD)	3.73 (2.68)	4.34 (2.46)		5.14 (4.20)	6.93 (4.63)	
Median (Q1, Q3)	2.50 (2.10, 4.47)	4.46 (2.83, 5.41)		3.86 (2.12, 7.45)	5.30 (3.41, 8.66)	
Min - Max	0.30 - 10.41	0.57 - 9.85		0.36 - 12.54	2.34 - 17.01	
TRP_IAA			0.81			1.00
Mean (SD)	13.76 (9.57)	13.14 (5.82)		17.04 (12.28)	20.60 (19.27)	
Median (Q1, Q3)	10.34 (6.58, 16.86)	11.85 (9.19, 18.12)		12.27 (9.92, 25.31)	13.19 (6.16, 32.42)	
Min - Max	2.94 - 35.29	3.09 - 22.02		2.12 - 40.36	3.44 - 63.91	
IAA_IALD			0.74			0.13
Mean (SD)	0.01 (0.01)	0.01 (0.01)		0.01 (0.00)	0.01 (0.00)	
Median (Q1, Q3)	0.01 (0.01, 0.02)	0.01 (0.01, 0.01)		0.01 (0.00, 0.01)	0.01 (0.01, 0.01)	
Min - Max	0.00 - 0.03	0.00 - 0.03		0.00 - 0.01	0.00 - 0.02	
TRP_IAM			0.86			0.16
Mean (SD)	0.38 (0.23)	0.42 (0.33)		0.38 (0.21)	0.53 (0.30)	
Median (Q1, Q3)	0.34 (0.18, 0.50)	0.27 (0.17, 0.73)		0.31 (0.26, 0.50)	0.44 (0.29, 0.79)	
Min - Max	0.10 - 1.03	0.02 - 1.05		0.12 - 0.76	0.19 - 1.08	
TRP_ILA			0.06			0.98

Mean (SD)	18.97 (10.71)	26.08 (14.03)		23.50 (17.11)	23.81 (14.36)	
Median (Q1, Q3)	15.34 (13.11, 22.27)	25.71 (14.21, 32.35)		21.55 (10.51, 29.64)	19.01 (15.41, 29.69)	
Min - Max	4.63 - 49.93	0.79 - 56.88		1.67 - 57.27	5.88 - 54.20	
TRP_NAcTRP			0.14			1.00
Mean (SD)	0.11 (0.06)	0.12 (0.04)		0.12 (0.08)	0.13 (0.07)	
Median (Q1, Q3)	0.09 (0.07, 0.13)	0.13 (0.10, 0.15)		0.10 (0.07, 0.15)	0.11 (0.09, 0.16)	
Min - Max	0.04 - 0.24	0.05 - 0.22		0.02 - 0.26	0.06 - 0.31	
TRP_TRPN			0.54			0.78
Mean (SD)	0.05 (0.03)	0.04 (0.02)		0.06 (0.04)	0.05 (0.02)	
Median (Q1, Q3)	0.04 (0.03, 0.07)	0.04 (0.03, 0.05)		0.05 (0.03, 0.10)	0.06 (0.05, 0.06)	
Min - Max	0.01 - 0.11	0.02 - 0.09		0.01 - 0.11	0.01 - 0.09	
TRP_5OHIAA			0.59			0.84
Mean (SD)	0.46 (0.25)	0.39 (0.21)		0.59 (0.43)	0.62 (0.48)	
Median (Q1, Q3)	0.36 (0.30, 0.63)	0.33 (0.23, 0.54)		0.46 (0.31, 0.75)	0.41 (0.21, 1.01)	
Min - Max	0.09 - 1.08	0.13 - 0.98		0.06 - 1.72	0.17 - 1.72	
TRP_5OHTRP			0.15			0.09
Mean (SD)	0.65 (0.45)	0.84 (0.54)		1.01 (0.61)	1.69 (0.83)	
Median (Q1, Q3)	0.50 (0.39, 0.67)	0.69 (0.40, 1.12)		0.85 (0.59, 1.32)	1.83 (1.06, 2.39)	
Min - Max	0.16 - 2.04	0.26 - 2.41		0.29 - 2.14	0.54 - 2.81	
TRP_5MIAA			0.60			0.09
Mean (SD)	2.09 (1.90)	2.32 (1.76)		0.84 (0.54)	1.45 (1.12)	
Median (Q1, Q3)	1.94 (0.67, 2.75)	1.69 (0.85, 4.01)		0.82 (0.40, 1.17)	1.09 (0.69, 1.95)	
Min - Max	0.18 - 7.36	0.23 - 5.54		0.11 - 1.70	0.27 - 3.76	
TRP_IPA			0.50			0.89
Mean (SD)	4.05 (2.83)	4.81 (3.50)		1.34 (0.97)	1.41 (1.09)	
Median (Q1, Q3)	3.24 (2.08, 5.28)	3.73 (1.89, 6.86)		1.04 (0.60, 2.05)	1.15 (0.64, 1.82)	

Min - Max	0.47 - 10.29	0.72 - 13.18		0.15 - 3.33	0.27 - 4.05	
TRP_IBA			0.12			0.62
Mean (SD)	1.34 (1.35)	2.00 (1.67)		4.49 (3.05)	4.96 (3.28)	
Median (Q1, Q3)	0.72 (0.34, 1.94)	1.78 (0.65, 2.71)		3.91 (2.58, 6.76)	4.41 (3.09, 6.48)	
Min - Max	0.09 - 4.62	0.29 - 6.17		0.09 - 9.00	0.64 - 11.28	
TRP_MIA			0.66			0.72
Mean (SD)	236.27 (126.73)	248.60 (165.80)		377.15 (283.27)	404.21 (252.53)	
Median (Q1, Q3)	215.29 (145.09, 317.42)	221.01 (140.82, 329.70)		337.72 (207.41, 435.25)	456.88 (178.25, 604.11)	
Min - Max	50.51 - 518.99	25.69 - 614.63		74.47 - 1182.53	49.48 - 723.93	
IAA_KYN			0.83			1.00
Mean (SD)	0.02 (0.02)	0.02 (0.02)		0.02 (0.01)	0.02 (0.01)	
Median (Q1, Q3)	0.02 (0.01, 0.03)	0.02 (0.01, 0.03)		0.02 (0.01, 0.02)	0.02 (0.00, 0.02)	
Min - Max	0.00 - 0.06	0.00 - 0.06		0.00 - 0.04	0.00 - 0.05	
KYN_IALD			0.18			0.17
Mean (SD)	0.55 (0.41)	0.74 (0.49)		0.57 (0.51)	0.68 (0.51)	
Median (Q1, Q3)	0.38 (0.30, 0.70)	0.61 (0.40, 0.93)		0.38 (0.23, 0.74)	0.60 (0.45, 0.65)	
Min - Max	0.15 - 1.73	0.05 - 1.66		0.06 - 1.60	0.13 - 1.93	
U-Creatinine			0.46			0.71
Mean (SD)	7.70 (3.88)	8.40 (5.06)		7.38 (5.02)	8.29 (4.33)	
Median (Q1, Q3)	6.70 (4.85, 9.85)	7.10 (4.40, 10.65)		6.10 (4.25, 9.60)	8.40 (5.25, 11.00)	
Min - Max	1.60 - 17.50	2.30 - 18.20		0.10 - 18.30	0.70 - 15.90	
